# Light, Switch, Action! The Influence of Geometrical Photoisomerization in an Adaptive Self-Assembled System

**DOI:** 10.1021/jacs.4c11206

**Published:** 2024-11-05

**Authors:** Marco Ovalle, Charlotte N. Stindt, Ben L. Feringa

**Affiliations:** Stratingh Institute for Chemistry, University of Groningen, Nijenborgh 3, Groningen 9747 AG, The Netherlands

## Abstract

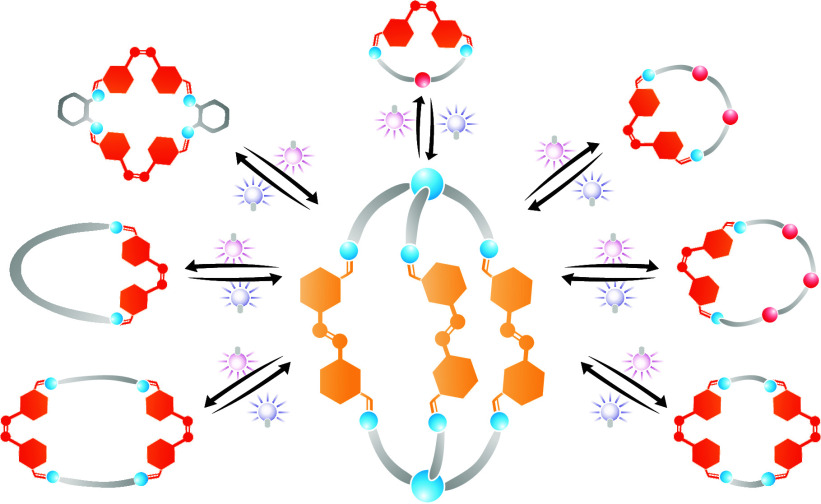

The ubiquitous ability of natural dynamic nanostructures to adapt to environmental changes is a highly desirable property for chemical systems, particularly in the development of complex matter, molecular machines, and life-like materials. Designing such systems is challenging due to the generation of complex mixtures with responses that are difficult to predict, characterize, and diversify. Here, we navigate between self-assembled architectures using light by operating an intrinsic photoswitchable building block that governs the state of the system. When complementary units are present, the photoswitch determines the predominant architecture, reversibly adapting between the cage and macrocycles, including (otherwise inaccessible) higher-energy assemblies. Our study showcases this concept with seven different transformations, offering an unprecedented degree of control, diversification, and adaptation by self-selecting complementary units. These findings could enable applications of on-demand dissipative macrocycles based on dynamic bonds. We also envision different transient nanostructures, e.g., reticular and polymeric materials, being explored by fine-tuning the nature of the complementary unit.

## Introduction

Living organisms represent the most complex form of known matter. Our abilities to adapt and maintain out-of-equilibrium states are key characteristics that distinguish us from inanimate objects.^1^ These properties contribute to the high level of complexity observed in biological systems. Understanding the principles behind these characteristics presents a fundamental challenge and holds promise for applying them to generate new life-like materials, molecular machines, and synthetic chemical systems.^2−8^

A powerful tool in the development of adaptable materials is the reversible formation of a chemical interaction. Dynamic covalent bonds, in particular, have proven to be an excellent motif for the design of building blocks that undergo spontaneous self-assembly into a myriad of molecular architectures.^9,10^ Examples include polymers,^11^ reticular materials,^12,13^ and discrete molecular architectures, such as macrocycles,^14−16^ cages,^17−20^ and mechanically interlocked molecules.^21−23^ Regardless of the nature of the self-assembled architecture, the dynamic behavior of its reversible bonds makes it susceptible to structural modifications in response to an external input.^16,24−26^ In particular, introducing energy into the dynamic system, either by a chemical fuel^27−32^ or in the form of light,^33−36^ allows the obtention of out-of-equilibrium states. This enables reversible changes in the assembly to occur on-demand with little to no modification of the overall chemical composition of the system.

Molecular switching^37^ of one of the components integrated into a molecular architecture can greatly influence its shape and function.^38−49^ The outcome in dynamic systems, however, is difficult to predict, often leading to rearrangement of its components into a new architecture^36,49−53^ or oligomerization.^26,54^ Pioneering work by the Lehn group^53^ showed how geometrical isomerization via metalloswitching can promote the reversible rearrangement of the components in an imine-based [2 + 2]macrocycle-to-[1 + 1]macrocycle transformation (the numbers inside the bracket denote the amount of self-assembled complementary units in the nanostructure), as well as macrocycle-to-polymer transformations. Similar behavior has been observed using light in metalorganic cage assemblies^50−52^ and other imine-based macrocycles.^36^ To the best of our knowledge, a general approach to navigate between a diverse variety of dynamic covalent nanostructures in a precisely controlled and reversible manner has not been reported. Instead, examples in the literature tend to undergo a rearrangement of their components, which prevents further transformation with an external unit, limiting the accessible assemblies.

**Figure 1:**
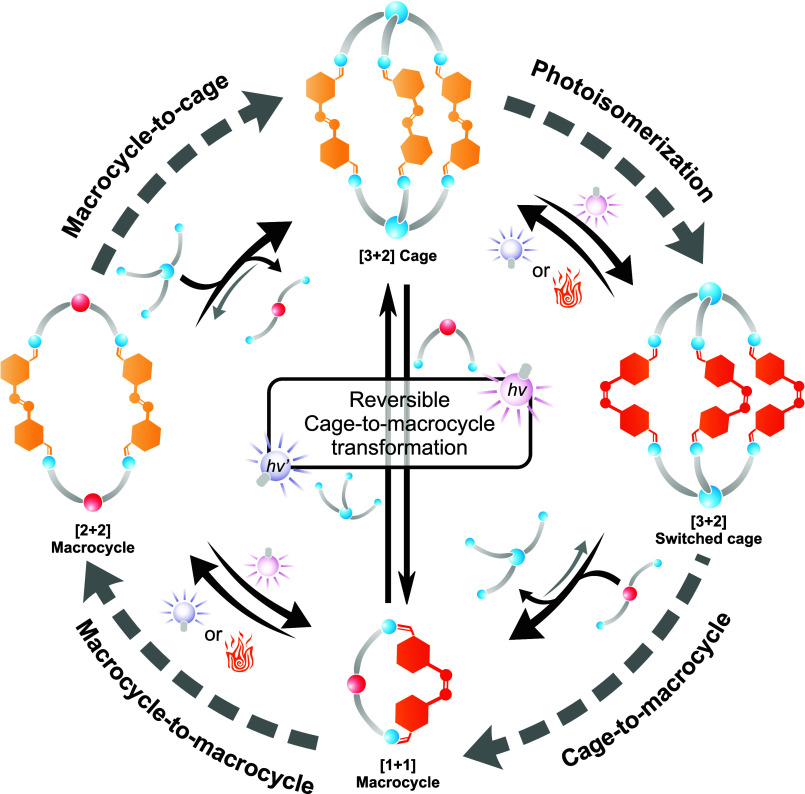
Different self-assemblies governed by a photoswitchable building block. Photoisomerization of the [3 + 2]cage **1** occurs reversibly upon the application of external photochemical stimuli. When a ditopic complementary building block is present, the strained cage undergoes a **[3 + 2]cage-to-[1 + 1]macrocycle** transformation. When isolated, the **[1 + 1]macrocycle-to-[2 + 2]macrocycle** transformation can be controlled with photochemical stimuli. In the presence of a competing tritopic complementary building block, the **[2 + 2]macrocycle-to-[3 + 2]cage** transformation occurs. When all the components coexist in solution, a **reversible [3 + 2]cage-to-[1 + 1]macrocycle transformation** is controlled with light.

Our group recently reported^33^ a system in which a dynamic imine [3 + 2]cage (**1**) composed of a ditopic azobenzene trimer is able to undergo geometric *E* → *Z* isomerization without rearrangement of its components. By doing so, the dynamic assembly becomes strained and high in energy. Reaching this state is key to further transform the metastable architecture via constitutional exchange with other components in solution. We hypothesized that by introducing an additional competing complementary unit with the potential to generate a different architecture, we would be able to navigate between different self-assemblies using light as an external stimulus and energy source in a controlled and reversible fashion (Figure 1).

In this study, we explore ditopic amines as a competing complementary unit in the system. This allowed us to develop seven different light-fueled reversible [3 + 2]cage-to-[*n* + *n*]macrocycle transformations (Figure 1). When the ditopic azobenzene (**2**) building block is in the presence of a tritopic and a ditopic amine, the state of the photoswitch determines the preference to form either the [3 + 2] cage or an [*n* + *n*] macrocycle. The thermally stable *E* form favors the kinetic and thermodynamic formation of cage *EEE*-**1**. Upon irradiation to the *Z* form, a near-quantitative and fast reaction with the complementary unit is observed, leading to the formation of the macrocycles.

The influence of photoswitching on isolated macrocycles was also studied. Depending on the ditopic amine used, the *E* → *Z* isomerization was followed by a ring contraction in a [2 + 2]macrocycle-to-[1 + 1]macrocycle transformation or switching without rearrangement of the components in a [2 + 2]macrocycle-to-switched[2 + 2]macrocycle transformation.

We anticipate that the principle of providing strain to a dynamic molecular architecture, followed by the constitutional transformation to a new nanostructure, will serve as a useful tool for the development of new responsive and adaptive dissipative systems. Moreover, the remarkable diversification capability in our system offers an ideal platform to explore other assemblies generated on-demand, for example, different macrocycles and cages with an engineered associated function, as well as mechanically interlocked molecules, molecular machines, polymers, and reticular materials.

## Results and Discussion

### Cage **1** Dynamic Behavior

We previously discovered that cage **1** can undergo stepwise isomerization from its most stable form following the sequence *EEE***–1***→ZEE***–1** → *ZZE***–1** → *ZZZ***–1** without rearrangement of its components and with negligible hydrolysis in benzene (Figure 1).^33^ By doing this, strain is introduced into the imine bonds, which favors the reactions of the photoisomers of **1** when a competing nucleophile is present, in contrast to the stable and less reactive *EEE***–1** isomer. In this work, we first evaluated the dynamic behavior of **1** in chloroform (1 mM) in the presence of a catalytic amount of trifluoroacetic acid (TFA, 5 μM) to accelerate the kinetics of the transformations and improve the solubility of the system. Under these conditions, only 15% of cage *EEE***–1** is hydrolyzed (Figure S1) into its components *E-***2** and tris(2-aminoethyl)amine (**TREN**). The switching process proceeds cleanly under these conditions (Figures S2 and S3, Videos S1 and S2).

### Macrocycle *EE*-**3** Self-Assembly and Switching

**Figure 2:**
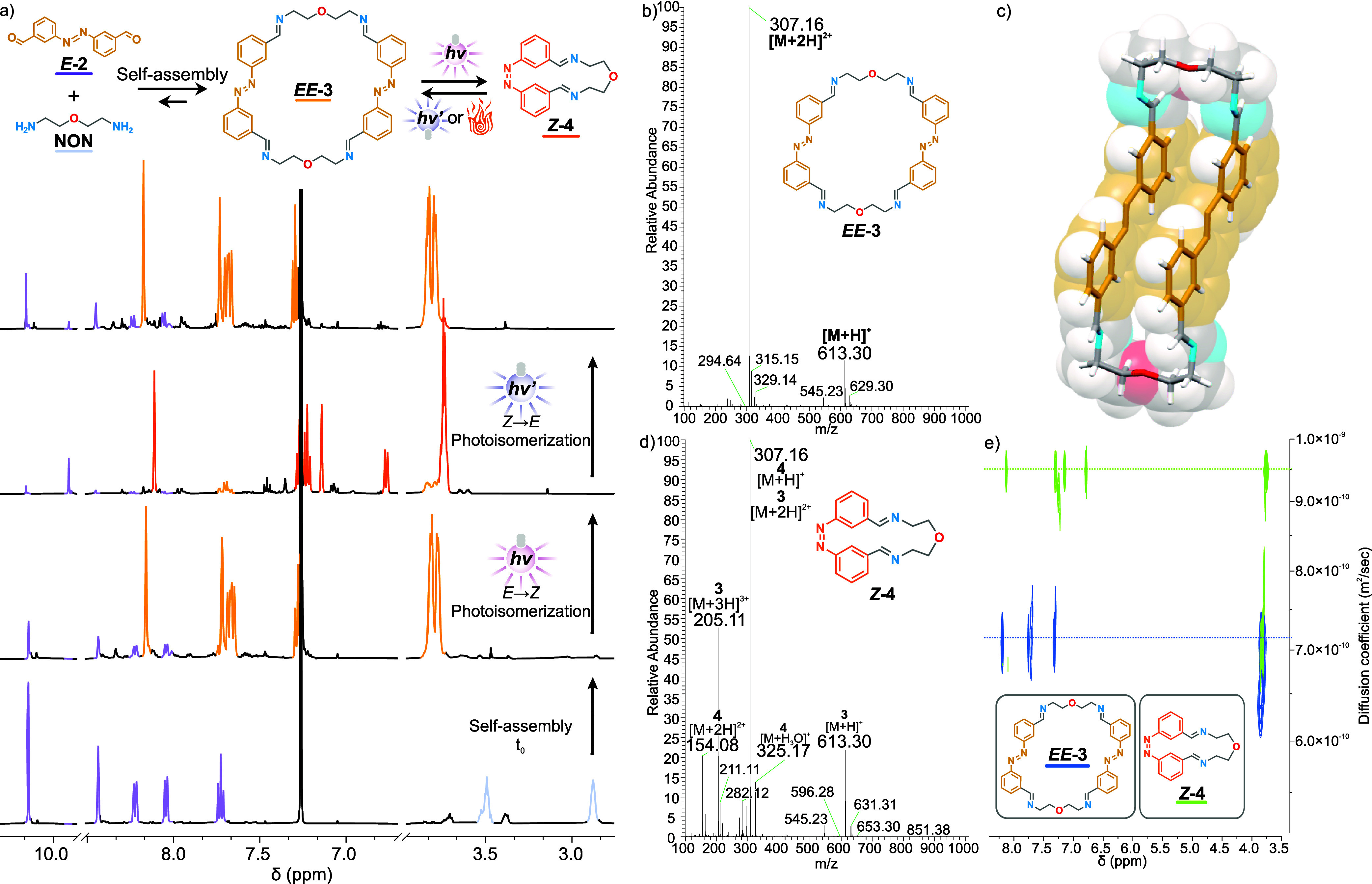
Self-assembly, characterization, and switching behavior of macrocycles 3 and 4. (a) Stacked ^1^H NMR spectra (500 MHz, CDCl_3_, TFA, 5 μM, 25 °C) of the self-assembly of *E-***2** (purple) and **NON** (blue) into *EE-***3** (yellow) and its photoswitching. From bottom to top: Immediately after mixing the components (*t*_0_, bottom), 3 h after the reaction, switching by UV irradiation (λ_irr_ = 340 nm, 1.8 h) generating the [1 + 1] macrocycle *Z-***4** (orange), switching by visible light irradiation (λ_irr_ = 415 nm, top) promoting the regeneration of the [2 + 2] macrocycle *EE-***3** (yellow). (b) ESI-HRMS spectra of *EE-***3** and (c) crystal structure of *EE***-3**. (d) ESI-HRMS spectra of *Z-***4** and (e) DOSY NMR overlay spectra (500 MHz, CDCl_3_ 5 μM TFA, 25 °C) of the sample before (blue) and after (green) UV irradiation (λ_irr_ = 340 nm).

The new macrocycles emerging from the ditopic amine 2-(2-aminoethoxy)ethanamine **NON** (3 mM) and the photoswitchable building block *E-***2** (3.3 mM) were studied by analyzing their self-assembly and switching behavior (Figure 2) in CDCl_3_ in the presence of a catalytic amount of TFA (5 μM). The process was followed by time-dependent ^1^H NMR spectroscopy (Figures 2a and S4 and S5). The reaction was completed within 3 h, as indicated by the full consumption of **NON** and the clean appearance of a new set of signals corresponding to the [2 + 2] macrocycle *EE***–3**. The structure was confirmed by ESI-HRMS (Figures 2b and S75) and X-ray crystallography (Figure 2c).

The switching behavior of *EE-***3** was studied by *in situ*^1^H NMR irradiation (λ_irr_ = 340 and 415 nm, Figures 2a and S6 and S9). The shielded signals in the aromatic region are characteristic of the *Z* isomer of the azobenzene moiety, indicating a successful switching process with a 94% yield. Further analysis by ESI-HRMS (Figures 2d, S6 and S7) and Diffusion-Ordered NMR spectroscopy (DOSY NMR, Figures 2e and S52 and S53) confirmed that the switching is associated with a [2 + 2]macrocycle-to-[1 + 1]macrocycle transformation from *EE-***3** to *Z-***4**.

The ESI-HRMS spectra for *EE-***3** (Figures 2b and S75) show the [**3** + H]^+^ species at *m*/*z* = 613.30 and the doubly charged species [**3** + 2H]^2**+**^ at *m*/*z* = 307.16. The doubly charged [**3** + 2H]^2+^ and singly charged [**4** + H]^+^ species are isobaric. However, after irradiation, a characteristic signal corresponding to the doubly charged species [**4** + 2H]^2+^ can be observed at *m*/*z* = 154.08 (Figures 2d and S76).

In addition, DOSY NMR spectra (Figures 2e, S52 and S54) of the irradiated and nonirradiated samples were recorded. After irradiation, a significant increase in the diffusion coefficient (9.5 × 10^–10^ m^2^/s for *Z-***4**) was observed compared to before irradiation (7.1 × 10^–10^ m^2^/s for *EE-***2**). This difference can be explained by the reduction of the molecular weight as a consequence of the [2 + 2]macrocycle-to-[1 + 1]macrocycle transformation.

The macrocycle *EE-***3** is regenerated from *Z-***4** in a [1 + 1]macrocycle-to-[2 + 2]macrocycle transformation upon visible light irradiation (λ_irr_ = 415 nm, Figures 2a, S8 and S9) with a yield of 88%. The thermal *Z* → *E* isomerization was also studied (Figures S10 and S11; it is noteworthy that under these conditions, heating the sample leads to significant hydrolysis, forming *Z-***2**). The newly formed *Z-***4** showed higher thermal stability than similar compounds, such as dialdehyde precursor *Z-***2**, cage *ZZE-***1**, and macrocycle *ZZ-***3** (*vide infra*). The [1 + 1] macrocycle *Z-***4** shows an increase in the thermal half-life of approximately 2 orders of magnitude (Figures S10–S12). Similar examples of increased thermal half-lives upon rearrangement of dynamic azobenzene-based assemblies have been reported.^50^

In summary, *E-***2** and **NON** self-assemble into the [2 + 2] macrocycle *EE-***3**. Upon irradiation, *EE-***3** is reversibly converted into the [1 + 1] macrocycle *Z-***4**, which shows enhanced thermal stability compared to the photoisomers depicted in Figure S12.

### Cage-To-Macrocycle Transformation

**Figure 3:**
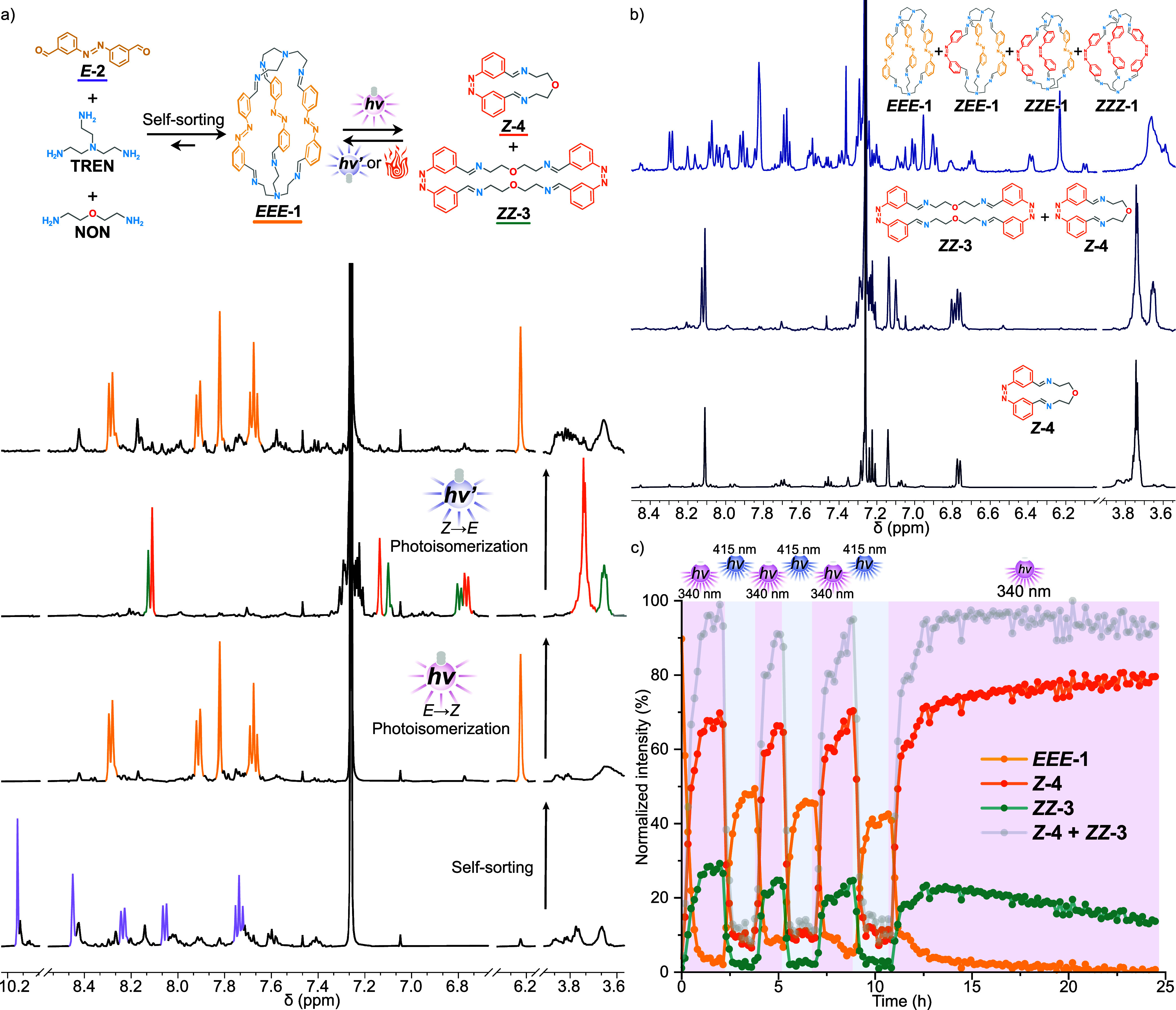
Reversible light-fueled cage-to-macrocycle transformation. (a) ^1^H NMR spectra (500 MHz, CDCl_3_ 5 μM TFA, 25 °C) of the self-assembly and switching (from bottom to top) of *E-***2** (purple), **NON**, and **TREN**. After 3 h of reaction, *EEE-***1** (yellow) is formed. The subsequent spectrum shows the result of the light-fueled cage-to-macrocycle transformation *EEE-***1** (yellow) → *Z-***4** (orange) + *ZZ-***3** (teal) after UV irradiation (λ_irr_ = 340 nm, 1.8 h). Finally, visible light irradiation (λ_irr_ = 415 nm, 1 h, top) promotes the light-fueled macrocycle-to-cage transformation *Z-***4** (orange) + *ZZ-***3** (teal) → *EEE-***1** (yellow). (b) Stacked ^1^H NMR spectra (500 MHz, CDCl_3_, 5 μM TFA, 25 °C) of (bottom) *Z-***4** obtained from the macrocycle-to-macrocycle transformation *EE-***3** → *Z-***4**. In the middle, macrocycles *Z-***4** and *ZZ-***3** obtained from the macrocycle-to-macrocycle transformation *EEE-***1** → **Z-4** + *ZZ-***3** are shown. Finally, at the top, the spectra of the mixture of isomers of **1** obtained from cage switching *EEE-***1** → *EEZ-***1** + *EZZ-***1** + *ZZZ-***1** are shown for comparison. (c) Fatigue study over 3.5 cycles of the light-fueled cage-to-macrocycle reversible transformation *EEE-***1** ↔ *Z-***4** + *ZZ-***3** obtained by *in situ*^1^H NMR irradiation (500 MHz, CDCl_3_, 5 μM TFA, 25 °C).

Having characterized the macrocycle behavior, the self-assembly of *E-***2** (three equiv), **TREN** (two equiv), and **NON** (three equiv) was then studied. The formation of cage **1** was kinetically favored over macrocycle **3** (Figures 3a, S13 and S14 and Video S3), as the latter was initially not observed. We then evaluated the thermodynamic preference (Figure S15) by adding **NON** (three equiv) to *EEE-***1** (one equiv). Around 15% of macrocycle **3** and 85% of cage **1** remained unreacted after 24 h at 25 °C in the dark. These experiments (Figures S13–S15) indicate that the cage is both thermodynamically and kinetically favored in the dark.

The photoisomerization process was followed by *in situ*^1^H NMR irradiation (λ_irr_ = 340 nm, Figures 3a, S16 and S17 and Video S3). After 1.8 h, the signals of cage *EEE-***1** disappeared as expected, but the signals for the different photoisomers of cage **1** (Figure 3b) were not observed. Instead, the signals corresponding to the [1 + 1] macrocycle *Z-***4** appeared, which confirms the successful light-fueled cage-to-macrocycle transformation. Additionally, in another experiment, the cage was photoisomerized before the addition of **NON** to follow the kinetics of this transformation. However, immediately after the addition, most of the reaction had already taken place (Video S4), indicating that the reaction is too fast to follow by NMR spectroscopy at 25 °C. Therefore, in our setup, the cage-to-macrocycle transformation is limited to the time it takes to reach the photostationary state. Thus, this reaction rate can be controlled by the intensity of light irradiation.

In comparison with the macrocycle-to-macrocycle transformation, a new set of signals appeared in the cage-to-macrocycle transformation (Figure 3b). These signals do not correspond to any of the isomers of cage **1** but are very similar to the signals of the [1 + 1] macrocycle *Z-***4**. DOSY ^1^H NMR spectra show a lower diffusion coefficient (Figure S55) of the new species. It was also observed that under prolonged irradiation (λ_irr_ = 340 nm, Figure 3c), the new set of peaks is converted into *Z-***4**. This led us to conclude that the new species is the [2 + 2] macrocycle *ZZ-***3**, which is otherwise inaccessible from the macrocycle-to-macrocycle transformation. When the cage-to-macrocycle transformation occurs, due to the increase in the local concentration of azobenzenes, the higher energy compound *ZZ-***3** can be observed. This represents an emergent property that can drive the system further away from equilibrium.

Subsequent irradiation with visible light (λ_irr_ = 415 nm, Figures 3a, S18 and S19) promotes the *Z* → *E* isomerization and the macrocycle-to-cage transformation. *EEE-***1** is regenerated to 60% of its initial intensity. This can be explained by the generation of a photostationary state distribution in which no cage isomer other than *EEE-***1** is able to exist in the presence of a competing amine, but a plethora of other kinetic products is generated.

Alternatively, thermal *Z* → *E* isomerization and macrocycle-to-cage transformation can be performed at higher temperatures (Figures S20 and S21). In comparison with the photochemical pathway that generates additional photoisomers and kinetic species (Figures S18 and S20), the thermal pathway proceeds cleanly, as evidenced by the absence of additional signals in the ^1^H NMR spectra (Figure S20). The enhanced thermal stability for *Z-***4** in comparison with *ZZ-***3** is evident as the latter disappears faster. This is expected, as the thermal stabilization arising from the ring contraction is not present in *ZZ-***3**.

Finally, the reversible cage-to-macrocycle transformation was subjected to a fatigue analysis (3.5 cycles over 28 h) and monitored by ^1^H NMR spectroscopy (Figure 3c and Video S5). No fatigue was observed.

### Macrocycle Self-Assembly between *E*-2 and Various Ditopic Amines

**Figure 4:**
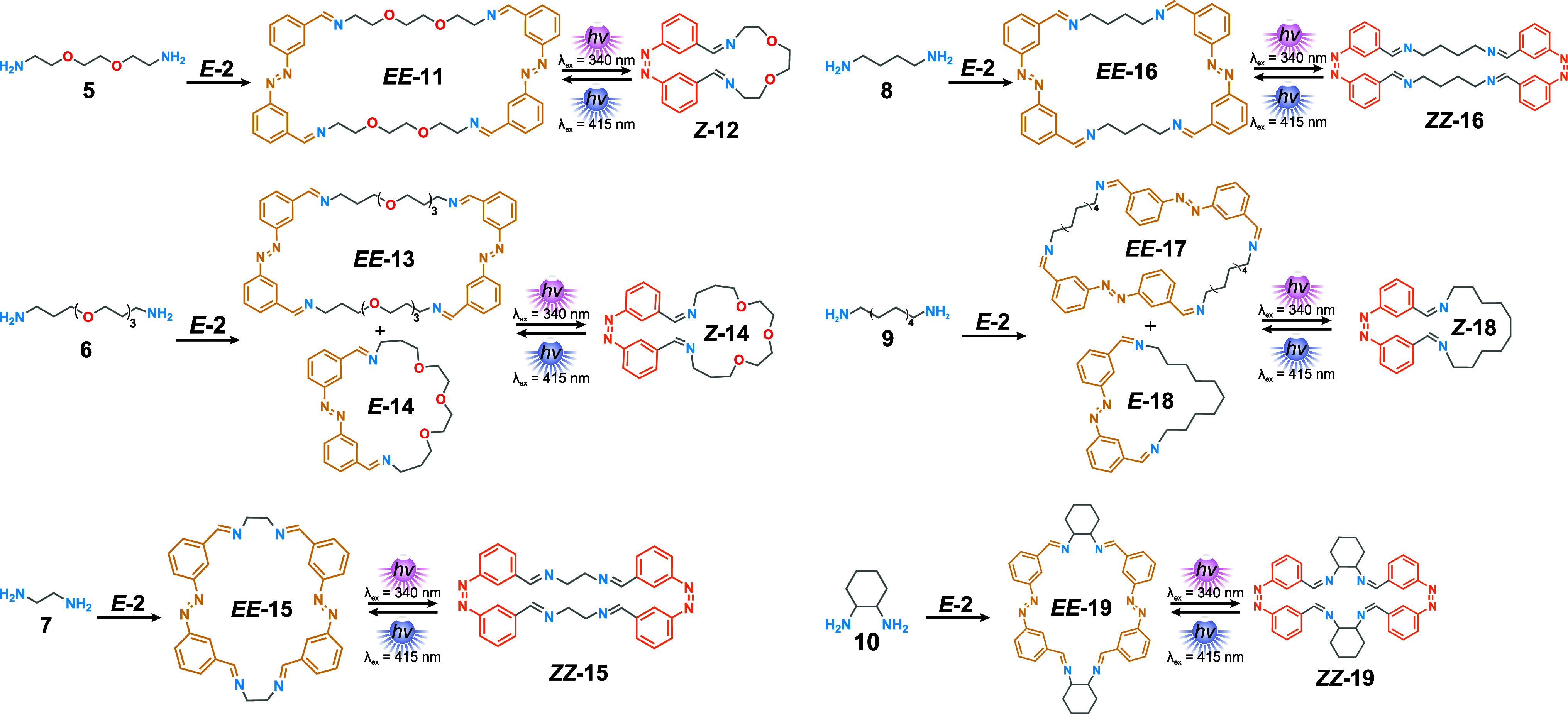
Summary of all the macrocyclic architectures formed in the self-assembly between amines **5–10** (1 equiv, 3 mM) and azobenzene *E-***2** (1.1 equiv, 3.3 mM) and the result of the *E* → *Z* isomerization upon UV (λ_irr_ = 340 nm) or visible (λ_irr_ = 415 mn) light irradiation. Conditions: CDCl_3_ in the presence of a catalytic amount of TFA (5 μM). Ditopic amine **10** was used as a racemic mixture of the *trans* isomers.

Following the same procedure used in the analysis of the system with **NON**, we employed a similar strategy to systematically study the behavior of cage **1** with ditopic amines **5**–**10** (Figure 4, where ditopic amine **10** was used as a racemic mixture of *trans* isomers) to further test the adaptability of our system. First, ^1^H NMR was used to follow the self-assembly of *E-***2** with ditopic amines **5**–**10** into macrocycles **11**–**19** (Figures 4, S21 and S27). For each case, 1.1 equiv of *E-***2** (3.3 mM) and 1 equiv of the corresponding ditopic amine (3 mM) were reacted in CDCl_3_ in the presence of a catalytic amount of TFA (5 μM). For amines **5**, **7**, and **10**, the reaction proceeds readily at room temperature after 16 h. For amines **6**, **8**, and **9**, it is necessary to perform the reaction at 50 °C over 16 h. For every reaction, new sets of peaks corresponding to the formation of imines can be observed. In most cases, a single major species can be observed, except for the reaction with amines **6** and **9** (Figures S23 and S26), in which case two different species can be observed that coexist in solution.

The macrocyclic order was determined by ESI-HRMS (Figures S75–S106). Similar to macrocyclic *EE-***3**, macrocycles **11**–**19** (Figure 4) can undergo multiple ionizations during the analysis, with the singly and doubly charged species giving the most intense signals. The [2 + 2] macrocycles were observed in high abundance in all cases. For the self-assembly of *E-***2** with the longer amines **6** and **9**, the [1 + 1] macrocycles *E-***14** and *E-***18** were also formed, in addition to the formation of the [2 + 2] macrocycles *EE-***13** and *EE-***17**, respectively (Figures S82–S83 and S96–97). This was also observed for amine **5**, forming mainly *EE-***11** and less *E-***12** (Figures S77–S78). It is noteworthy that low-intensity signals corresponding to higher-order [3 + 3] and even [4 + 4] macrocycles **20**–**28** were also observed by HRMS.

The difference in the macrocyclic order can be observed by DOSY NMR. For macrocycles **13**–**14** (Figures S59–S60) and **17**–**18** (Figure S69) that coexist in solution, two different diffusion coefficients are observed for the different sets of peaks. In both cases, we assigned the lower diffusion profile to the [2 + 2] macrocycle and the higher diffusion rate to the [1 + 1] macrocycle, as we expect the smaller compound to diffuse faster, in analogy to the previously characterized ***EE***-**3 ↔ *Z***-**4** system.

Based on these results, we conclude that *E-***2** forms predominantly [2 + 2] macrocycles in the presence of ditopic amines. If the ditopic amine is large enough, a significant amount of the [1 + 1] macrocycle is also generated (Figure 4). Additionally, a variety of higher order macrocycles can be formed, albeit at much lower abundance.

### Photoswitching of Various Macrocycles

**Figure 5:**
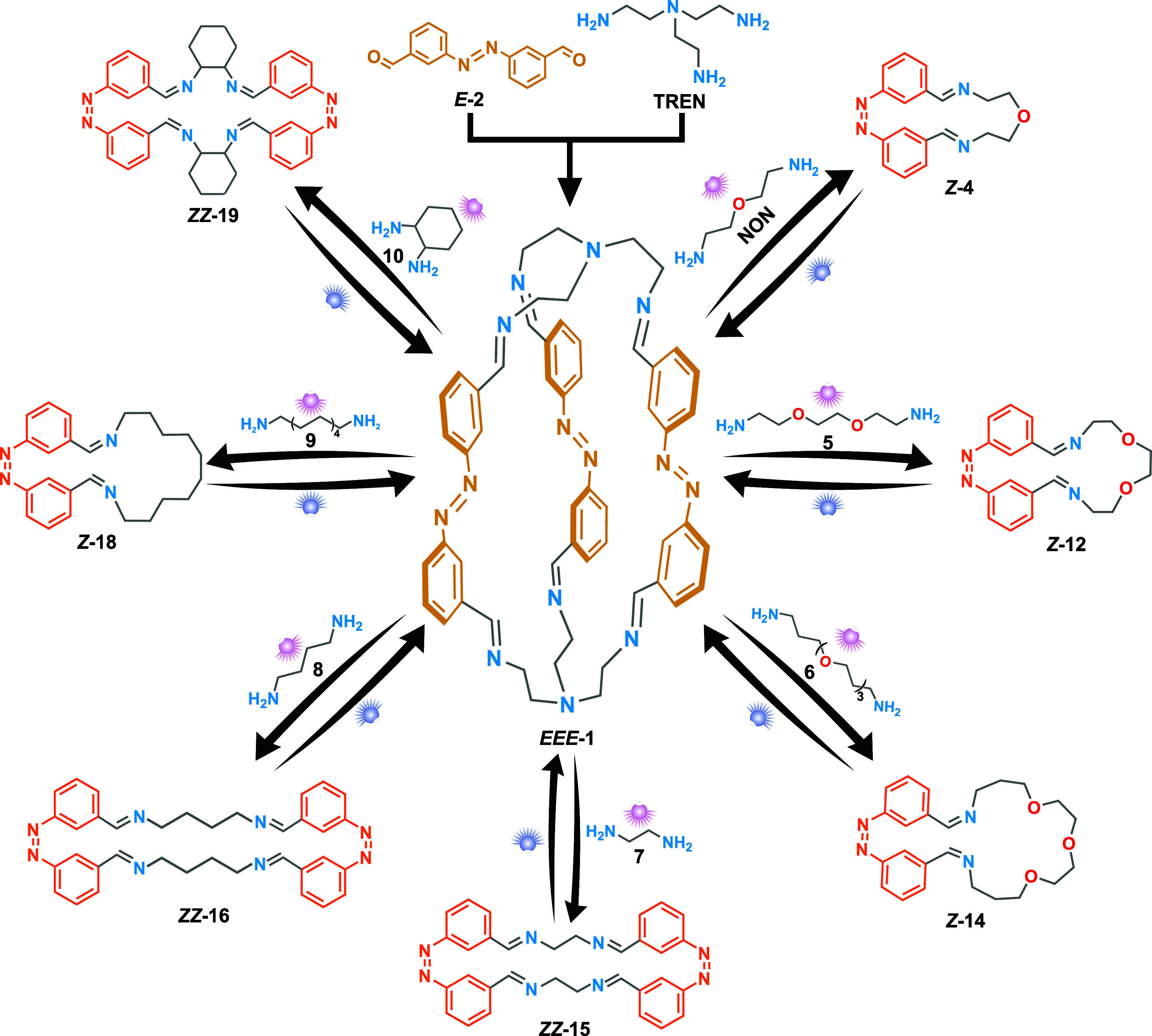
Summary of all reversible cage-to-macrocycle transformations with **1** and amines **5–10.** Ditopic amine **10** was used as a racemic mixture of *trans* isomers.

The switching behavior of macrocycles **11**–**19** was studied by *in situ*^1^H NMR irradiation with UV light (λ_irr_ = 340 nm, 3h), followed by another period of visible light irradiation (λ_irr_ = 415 nm, 1 h). All macrocycles displayed excellent photoswitching properties, having a PSS_340_ ratio above *E*:*Z* = 5:95 for the *E* → *Z* isomerization as well as a PSS_415_ ratio above *E*:*Z* = 80:20 for the *Z* → *E* process (Figures 5 and S21–S27). Remarkably, the mixtures containing different coexisting macrocycles **13**–**14** and **17**–**18** gave rise to a single compound after switching (Figures S23 and S26). Analyzing the DOSY NMR spectra before and after irradiation allowed us to determine the macrocycle order after irradiation (Figure 5, Table 1, and Figures S56–S74). Macrocycles *EE-***15**, *EE-***16**, and *EE-***19** were able to switch while maintaining the same [2 + 2] macrocyclic order, as indicated by the retention of a similar diffusion coefficient. In contrast, macrocycles *EE-***11**, *EE-***13**, and *EE-***16** showed an increase in the diffusion coefficient upon irradiation, suggesting that a [2 + 2]macrocycle-to-[1 + 1]macrocycle transformation occurs in these assemblies, as was also observed for *EE*-**3**.

**Table 1:**
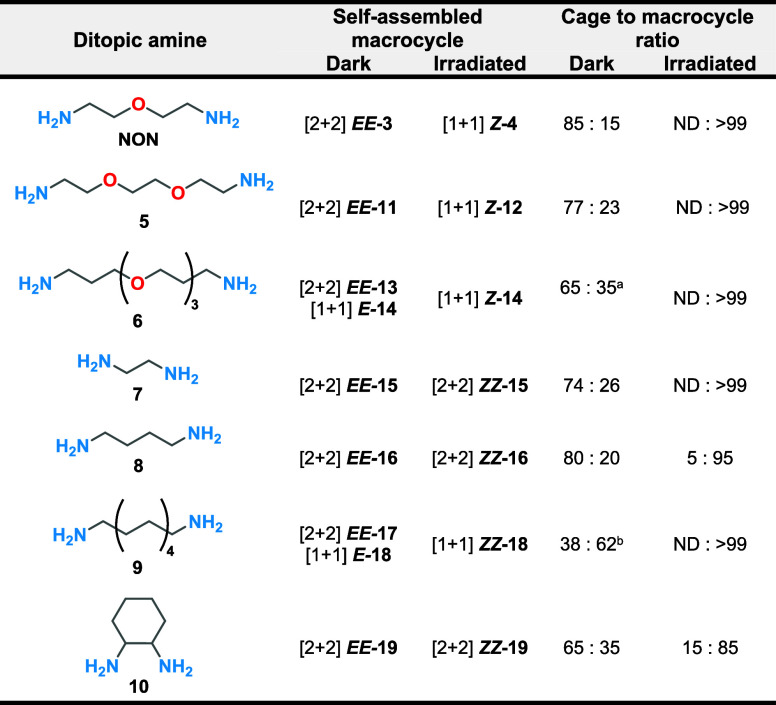
Summary of the Macrocycle Order upon Self-Assembly between *E***-2** with Ditopic Amines **NON** and **5–10** in the Dark and upon Irradiation as well as the Ratios of the Cage and Macrocycle after Mixing Amines *E***-2**, **TREN**, and Ditopic Amines **NON** and **5**–**10**^a^^b^^c^

a The macrocyclic distribution includes *EE-***13** and *E-***14** in 13% and 21%, respectively, of the total mixture.

b The macrocyclic distribution includes *EE-***17** and *E-***18** in 36% and 26%, respectively, of the total mixture.

c Ditopic amine 10 was used as a racemic mixture of trans isomers.

### Cage-to-Macrocycle Transformations

We evaluated the assembly of 3 equiv of *E-***2** (3 mM), 3 equiv of the corresponding ditopic amines **5**–**10** (3 mM), and 2 equiv of tritopic amine **TREN** (2 mM) in CDCl_3_ in the presence of a catalytic amount of TFA (5 μM). *In situ*^1^H NMR spectroscopy was used to follow the process (Figures S28–S51 and Videos S6–S11). Comparing the signals of the previous macrocycles allowed us to determine the ratio of cage/macrocycle present in both the dark and the irradiated state. It is important to note that additional signals not ascribable to the cage or macrocycle are present at lower intensity. The cage-to-macrocycle behavior is summarized in Figure 5.

For all cases except for amine **9**, we observe that the cage was the most abundant species in the dark (Table 1). As observed with **NON**, the cage-to-macrocycle transformation in which a [1 + 1] macrocycle is expected (amines **5**, **6**, and **9**) occurred cleanly without detectable NMR signals for the isomers of cage **1** after irradiation. Interestingly, for the amines in which no [2 + 2]macrocycle-to-[1 + 1]macrocycle transformation takes place (**7**, **8**, and **10**), the cage-to-macrocycle transformation is also favored but occurs along with the generation of more species detected by ^1^H NMR. This suggests that the additional contraction of the ring observed in the [2 + 2]macrocycle-to-[1 + 1] transformation further stabilizes and favors the macrocycle formation in the cage-to-macrocycle transformation. In contrast, macrocycles **15**, **16**, and **19**, similarly to cage **1**, can undergo photoswitching without rearrangement of their components, potentially generating strain and favoring the reaction with competing nucleophiles. This property makes them suitable candidates to be studied for new dissipative transformations, taking advantage of the same principle of the introduction of strain in the self-assembly.

## Conclusions

In summary, we were able to control the constitutional self-assembly of a dynamic covalent nanostructure by the geometric isomerization of a building block embedded into it. The strain introduced in the assembly allowed us to promote a constitutional exchange with nucleophiles of our choice. The introduction of ditopic complementary units forces the system to adapt to an external stimulus by forming either a cage or one of the many macrocycles explored in this work. In total, we performed seven light-fueled and reversible cage-to-macrocycle transformations. The systematic study of the dynamic behavior for the different dynamic components in the systems allowed us to understand the complex transformations taking place in the different steps of the process, including the formation of otherwise inaccessible and higher energy species. This novel approach to precisely control molecular architectures at the nanometric scale opens the door to a new generation of materials with the ability to be generated and dissembled on-demand. Several imine-based macrocycles with an associated potential function such as molecular recognition in host–guest systems, molecular machinery, particularly in the formation of interlocked structures, etc., could be explored following the procedures detailed in this work. Furthermore, this concept could be extended to different architectures by fine-tuning the nature of the complementary unit (e.g., topicity and geometry) to obtain a diverse collection of responsive and adaptive reticular and polymeric materials.

## Supporting Information Available

General information;^55−60^ synthesis and characterization; cage 1 dynamic behavior in CDCl_3_; macrocycle EE-**3** self-assembly and switching; cage-to-macrocycle transformation; self-assembly and switching of macrocycles **11**–**19**; cage-to-macrocycle transformations with ditopic amines 5–10; DOSY NMR spectra of macrocycles 3–4 and 11–19; ESI-HRMS of self-assembled macrocycles; NMR spectra (PDF)
Videos of the ^1^H NMR spectra evolution for selected kinetic experiments (MP4, MP4, MP4, MP4, MP4, MP4, MP4, MP4, MP4, MP4, MP4)
