# Supplementary material for: Light, Switch, Action! The Influence of Geometrical Photoisomerization in an Adaptive Self-Assembled System
Source: J Am Chem Soc. 2024 Nov 5;146(46):31892–900. doi: 10.1021/jacs.4c11206 (PMC11583216; doi:10.1021/jacs.4c11206)
Supplement: Supplementary file 1 — ja4c11206_si_001.pdf [file ja4c11206_si_001.pdf]

**Supporting Information**

# Light, switch, action! The influence of geometrical photoisomerization in an adaptive self-assembled system.

Marco Ovalle[1], Charlotte N. Stindt[1], Ben L. Feringa[1]*

1. Stratingh Institute for Chemistry
University of Groningen
Nijenborgh 3, 9747AG Groningen (The Netherlands)
E-mail: b.l.feringa@rug.nl

## Table of contents

General information ....... 2
Synthesis and characterization ....... 3
Cage 1 dynamic behavior in $CDCl_3$ ....... 7
Macrocycle *EE*-3 self-assembly and switching ....... 9
Cage-to-macrocycle transformation ....... 18
Self-assembly and switching of macrocycles 11 – 19. ....... 27
Cage-to-macrocycle transformations with ditopic amines 5-10 ....... 33
DOSY NMR spectra of macrocycles 3-4 and 11-19 ....... 57
ESI-HRMS of self-assembled macrocycles ....... 69
Apendix ....... 88
NMR spectra ....... 88
References ....... 92

## General information

Chemicals were purchased from commercial sources and used without purification. NMR spectra were recorded on a Varian Mercury Plus ($^1$H 400 MHz, $^{13}$C 100 MHz), or a Varian Unity Plus ($^1$H 500 MHz, $^{13}$C 125 MHz). Chemical shifts are in parts per million (ppm) relative to TMS, using the $CDCl_3$ solvent residual peak as internal standard (δ = 7.26 for $^1$H, δ = 77.16 for $^{13}$C). Data is reported as chemical shifts (δ) in ppm, multiplicity (s = singlet, d = doublet, dd = doublet of doublets, ddd = doublet of doublets of doublets, td = triplet of doublets, t = triplet, q = quartet, br. = broad, m = multiplet), coupling constants *J* (Hz), and integration. Variable temperature NMR, *in-situ* irradiation and DOSY NMR experiments were performed using a Varian Inova 500 (500 MHz) spectrometer. NMR irradiation experiments were performed at 25 °C with a fiber-coupled LED and a 1000 μm optical fiber (FP1000URT) modified to fit into the NMR tube, as described in the literature[1] or *ex-situ* with the same LEDs positioned at a distance of 3 cm from the samples. Irradiation experiments were performed using Thorlabs LEDs (M340F3, M415L4). DOSY NMR experiments were run in 5 mm precision tubes (Wilmad® Z272027). Diffusion Bipolar Pulse Pair Stimulated Echo (Dbppste) pulse sequence was used with a diffusion gradient length (δ) of 2.0 ms and a diffusion delay (Δ) of 130.0 ms for all cases. Raw data was analyzed using the peak fit DOSY transform function integrated in MestReNova v15. High Resolution Mass spectra (HRMS) were recorded on a LTQ Orbitrap XL (ESI+, ESI-, APCI+).

Compounds (*E*)-3,3'-(diazene-1,2-diyl)dibenzaldehyde (***E*-2**) and cage **1** were prepared according to literature procedures.[2,3]

Supplementary videos of the $^1$H NMR spectra evolution are provided for selected kinetic experiments and indicated in the corresponding figure caption.

### General procedure for the cage-to-macrocycle transformations

A stock solution of ***E*-2** (0.34 mg, 1.4 μmol, 1.1 eq.) in 0.2 mL of $CDCl_3$ acidified with trifluoroacetic acid (TFA, 5 μM) was mixed in the dark with acidified stock solutions ($CDCl_3$, TFA, 5 μM) of tris(2-aminoethyl)amine (**TREN**, 0.13 mg, 0.87 μmol, 0.67 eq in 0.2 mL) and the corresponding ditopic amine (**NON**, **5-10**, 1.3 μmol, 1 eq in 0.2 mL). Immediately after mixing, the reaction was monitored by *in-situ* $^1$H NMR for 8 h. The mixture was then allowed to relax for at least 8 more h before performing the $^1$H NMR *in-situ* irradiation experiments.

### DOSY NMR sample preparation and irradiation

For macrocycle ***EE*-3**, the DOSY NMR sample was prepared by dissolving the isolated powder in $CDCl_3$ in a concentration of 1.5 mM. This sample was irradiated for 2 h ($\lambda_{ex}$ = 340 nm) to obtain the spectra of ***Z*-4**. Macrocycles **11**-**19** were prepared following the same procedure as the one described for the self-assembly of macrocycles. The NMR samples were *ex-situ* irradiated ($\lambda_{ex}$ = 340 nm) by placing the LED next to the NMR tube for 4 h in order to measure the photoisomerized macrocycles.

### High Resolution Mass spectra (HRMS) Sample preparation

For macrocycle ***EE*-3**, the HRMS sample was prepared from the isolated powder dissolved in $CHCl_3$ in a concentration of 1.5 mM. This sample was irradiated for 2 h ($\lambda_{ex}$ = 340 nm) to obtain the spectra of ***Z*-4**. Macrocycles **11**-**19** were prepared following the same procedure as the one described for the self-assembly of macrocycles using $CHCl_3$ instead of $CDCl_3$.

## Synthesis and characterization

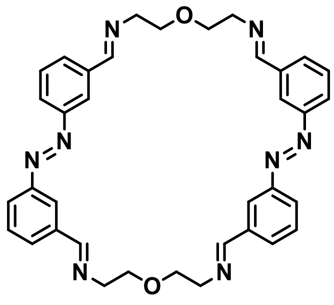

Synthesis of macrocycle ***EE*-3**

To a solution of ***E*-2** (50 mg, 0.21 mmol) dissolved in 5 mL of acetonitrile, a solution of 2-(2-aminoethoxy)ethanamine (**NON**, 23 mg, 0.22 mmol) in 5 mL was added dropwise and stirred for 16 h at room temperature in the dark. After the reaction was completed, the solvent was reduced by a constant flow of dry $N_2$, yielding ***EE*-3** a light orange powder (64 mg, 0.10 mmol, 98%). $^1$H NMR (400 MHz, $CDCl_3$) δ 8.17 (s, 1H), 7.73 (t, *J* = 1.8 Hz, 1H), 7.69 (d, *J* = 7.6 Hz, 1H), 7.67 (d, *J* = 7.6 Hz, 1H), 7.29 (t, *J* = 7.7 Hz, 1H), 4.37 – 3.18 (m, 4H). $^{13}$C NMR (101 MHz, $cdcl_3$) δ 162.29, 152.18, 136.97, 129.46, 129.08, 124.77, 123.31, 69.22, 60.93. HRMS, m/z $[M+H]^+$ calculated for $C_{36}H_{36}N_8O_2$: 613.3034; found: 613.3034.

General procedure for the self-assembly of macrocycles **3**, **11-19**

A stock solution of ***E*-2** (0.34 mg, 1.4 µmol, 1.1 eq.) in 0.25 mL of $CDCl_3$ acidified with trifluoroacetic acid (TFA, 5 µM) was mixed in the dark with an acidified stock solution ($CDCl_3$, TFA, 5 µM) of the corresponding ditopic amine (**NON**, **5-10**, 1.3 µmol, 1 eq in 0.25 mL). The self-assembly was performed in the dark for at least 16 h at room temperature or 50 °C and analyzed without further purification. The self-assembly process was followed by $^1$H NMR. The macrocycle formation was evaluated by integration of the imine signal with respect to the aldehyde. Typically, the self-assembly proceeds in 90->99%.

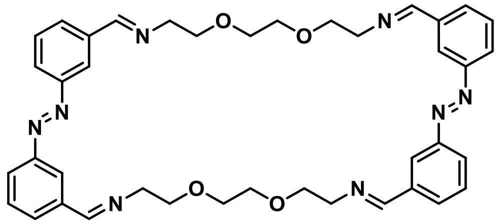

***EE*-11**. Following the general procedure for the self-assembly of macrocycles, ***E*-2** was reacted with 2,2'-(ethane-1,2-diylbis(oxy))bis(ethan-1-amine) (**5**) to form ***EE*-11** (98%). $^1$H NMR (500 MHz, $cdcl_3$) δ 8.27 (s, 1H), 8.12 (t, *J* = 1.9 Hz, 1H), 7.90 (d, *J* = 8.0 Hz, 1H), 7.84 (d, *J* = 7.6 Hz, 1H), 7.49 (t, *J* = 7.7 Hz, 1H), 3.78 (d, *J* = 4.8 Hz, 2H), 3.75 (d, *J* = 5.1 Hz, 2H), 3.65 (s, 2H). HRMS, m/z $[M+H]^+$ calculated for $C_{40}H_{44}N_8O_4$: 701.3558; found: 701.3553.

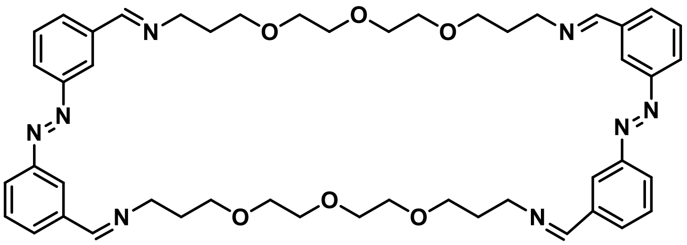


***EE*-13**. Following the general procedure for the self-assembly of macrocycles, ***E*-2** was reacted with 3,3'-((oxybis(ethane-2,1-diyl))bis(oxy))bis(propan-1-amine) (**6**) to form a mixture of ***EE*-13** (51% ) and ***E*-14** (45%). $^1$H NMR (500 MHz, $CDCl_3$) δ 8.35 (s, 1H), 8.18 (t, *J* = 1.9 Hz, 1H), 7.92 (d, *J* = 8.2 Hz, 1H), 7.83 (d, *J* = 7.6 Hz, 1H), 7.50 (t, *J* = 7.7 Hz, 1H), 3.68 – 3.52 (m, 8H), 2.05 – 1.9 (m, 2H). HRMS, m/z $[M+H]^+$ calculated for $C_{48}H_{60}N_8O_6$: 845.4709; found: 845.4704.

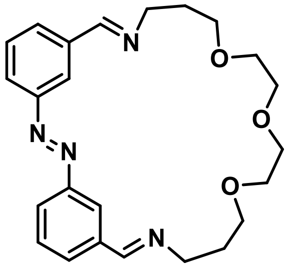


***E*-14**. Following the general procedure for the self-assembly of macrocycles, ***E*-2** was reacted with 3,3'-((oxybis(ethane-2,1-diyl))bis(oxy))bis(propan-1-amine) (**6**) to form a mixture of ***EE*-13** (51%) and ***E*-14** (45%). $^1$H NMR (500 MHz, $CDCl_3$) δ 8.55 (t, *J* = 1.9 Hz, 1H), 8.41 (s, 1H), 8.00 (dt, *J* = 8.1, 1.5 Hz, 1H), 7.65 (d, *J* = 7.5 Hz, 1H), 7.56 (t, *J* = 7.7 Hz, 1H), 3.78 – 3.70 (m, 8H), 2.05 – 1.9 (m, 2H). HRMS, m/z $[M+H]^+$ calculated for $C_{24}H_{30}N_4O_3$: 423.2391; found: 423.2388.

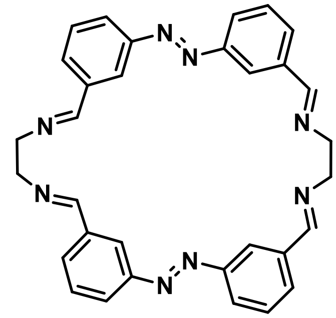


***EE*-15**. Following the general procedure for the self-assembly of macrocycles, ***E*-2** was reacted with ethane-1,2-diamine (**7**) to form ***EE*-15** quantitively. $^1$H NMR (500 MHz, $CDCl_3$) δ 8.29 (s, 1H), 8.07 (d, *J* = 1.8 Hz, 1H), 7.88 (d, *J* = 7.8 Hz, 1H), 7.79 (d, *J* = 7.7 Hz, 1H), 7.50 (t, *J* = 7.8 Hz, 1H), 4.09 (s, 2H). HRMS, m/z $[M+H]^+$ calculated for $C_{32}H_{28}N_8$: 525.2510; found: 525.2510.

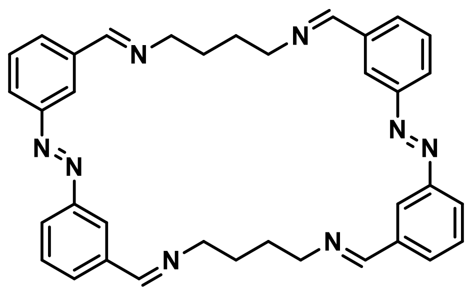

***EE*-16**. Following the general procedure for the self-assembly of macrocycles, ***E*-2** was reacted with butane-1,4-diamine (**8**) to form ***EE*-16** (90%). $^1$H NMR (500 MHz, $CDCl_3$) δ 8.36 (s, 1H), 8.28 (s, 1H), 7.93 (d, *J* = 8.1 Hz, 1H), 7.79 (d, J = 7.6 Hz, 1H)., 7.53 (t, *J* = 7.6 Hz, 1H), 3.76 – 3.71 (m, 2H), 1.85 – 1.77 (m, 2H). HRMS, m/z $[M+H]^+$ calculated for $C_{36}H_{36}N_8$: 581.3136; found: 581.3134.

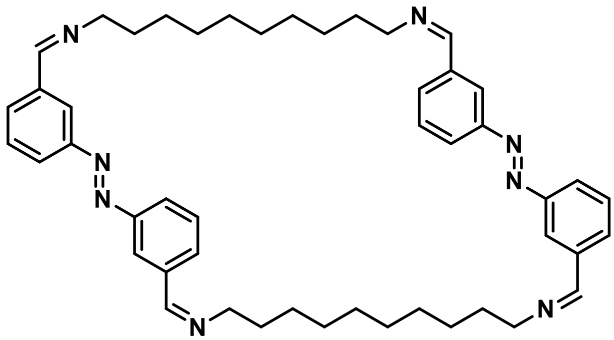

***EE*-17**. Following the general procedure for the self-assembly of macrocycles, ***E*-2** was reacted with decane-1,10-diamine (**9**) to form a mixture of ***EE*-17** (62%) and ***E*-18** (38%). $^1$H NMR (500 MHz, $CDCl_3$) δ 8.35 (s, 1H), 8.26 (t, J = 1.8 Hz, 1H), 7.99 – 7.94 (m, 1H), 7.85 (d, J = 7.6 Hz, 1H), 7.60 – 7.48 (m, 1H), 3.64 (t, J = 6.7 Hz, 2H), 1.76 – 1.66 (m, 2H), 1.48 – 1.13 (m, 6H). HRMS, m/z $[M+H]^+$ calculated for $C_{48}H_{60}N_8$: 749.5014; found: 749.5010.

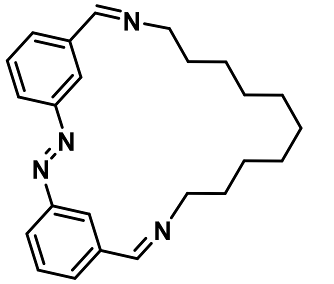

***E*-18**. Following the general procedure for the self-assembly of macrocycles, ***E*-2** was reacted with decane-1,10-diamine (**9**) to form a mixture of ***EE*-17** (62%) and ***E*-18** (38%). $^1$H NMR (500 MHz, $CDCl_3$) δ 8.70 (t, *J* = 1.9 Hz, 1H), 8.42 (s, 1H), 7.96 (d, *J* = 7.7 Hz, 1H), 7.63 – 7.41 (m, 2H), 3.68 (s, 2H), 1.77 – 1.66 (m, 2H), 1.51 – 1.14 (m, 8H). HRMS, m/z $[M+H]^+$ calculated for $C_{24}H_{30}N_4$: 375.2543; found: 375.2541.

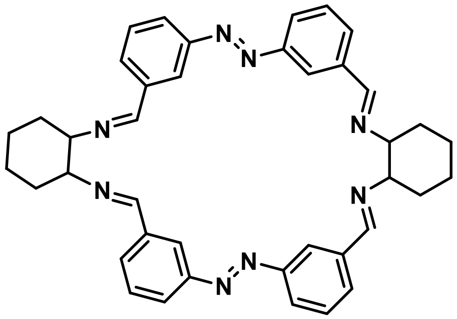

***EE*-19**. Following the general procedure for the self-assembly of macrocycles, ***E*-2** was reacted with (±)-*trans*-ciclohexane-1,2-diamine (**10**) to form ***EE*-19** quantitively. $^{1}$H NMR (500 MHz, $CDCl_3$) δ 8.36 (s, 1H), 8.13 (t, *J* = 1.9 Hz, 1H), 7.85 (dt, *J* = 7.8, 1.4 Hz, 1H), 7.76 (d, *J* = 7.6 Hz, 1H), 7.44 (t, *J* = 7.7 Hz, 1H), 3.62 – 3.38 (m, 1H), 1.98 – 1.91 (m, 2H), 1.59 – 1.55 (m, 2H). HRMS, m/z $[M+H]^+$ calculated for $C_{40}H_{40}N_8$: 633.3449; found: 633.3446.

# Cage 1 dynamic behavior in $CDCl_3$.

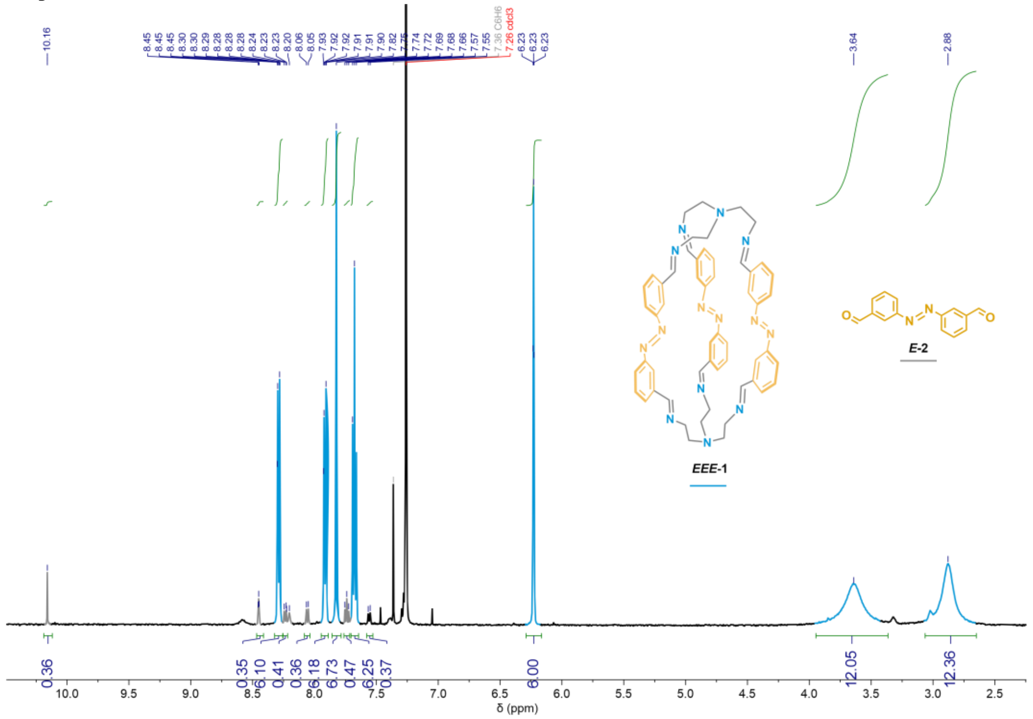


S1. $^{1}$H NMR of ***EEE*-1** in $CDCl_3$ with TFA 5 µM after 24 h equilibration at 25 °C.

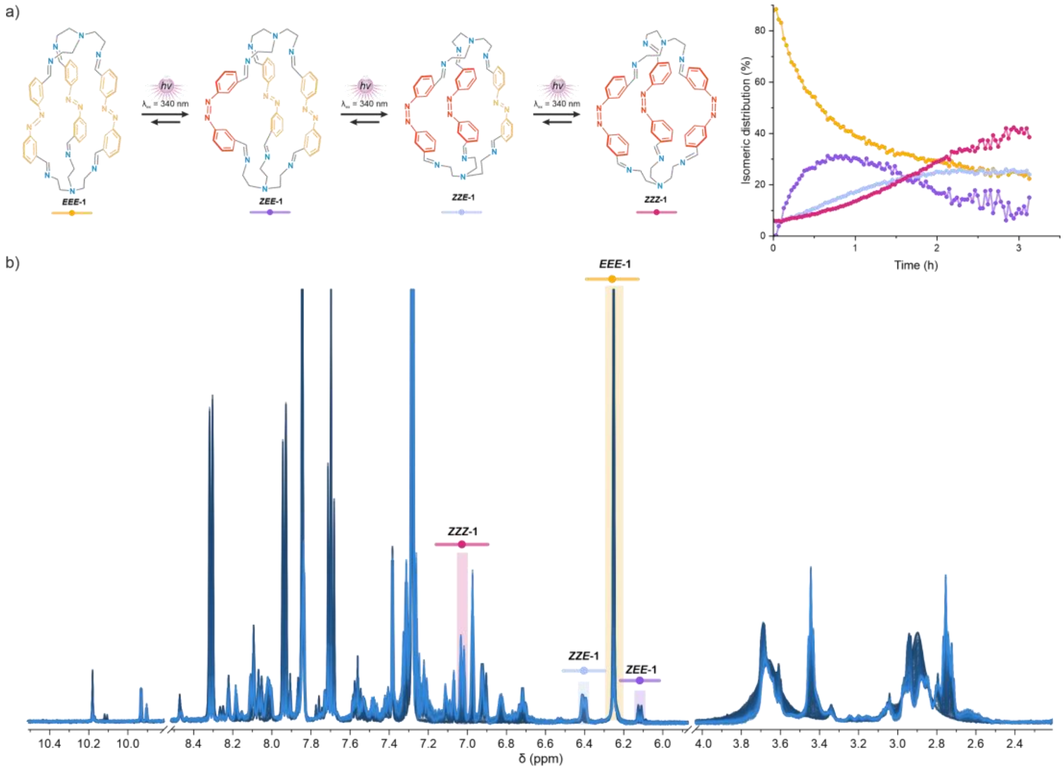


S2. Photoisomerization process ***EEE*-1** ⟶ ***ZZZ*-1** (total concentration of **1 =** 1 mM) under UV light irradiation ($\lambda_{irr}$ = 340 nm, 25 °C) over 3.2 h in $CDCl_3$ with TFA 5 µM. **a**, Kinetic traces of the evolutions of the different isomers of **1**. **b**, Evolution of the $^{1}$H NMR (500 MHz, $CDCl_3$, TFA 5 µM, 25 °C) spectra (from black to blue) during *in-situ* irradiation of **1**. The signals used to follow the kinetic traces are displayed with their respective color code. Supporting video 1 illustrates this process.

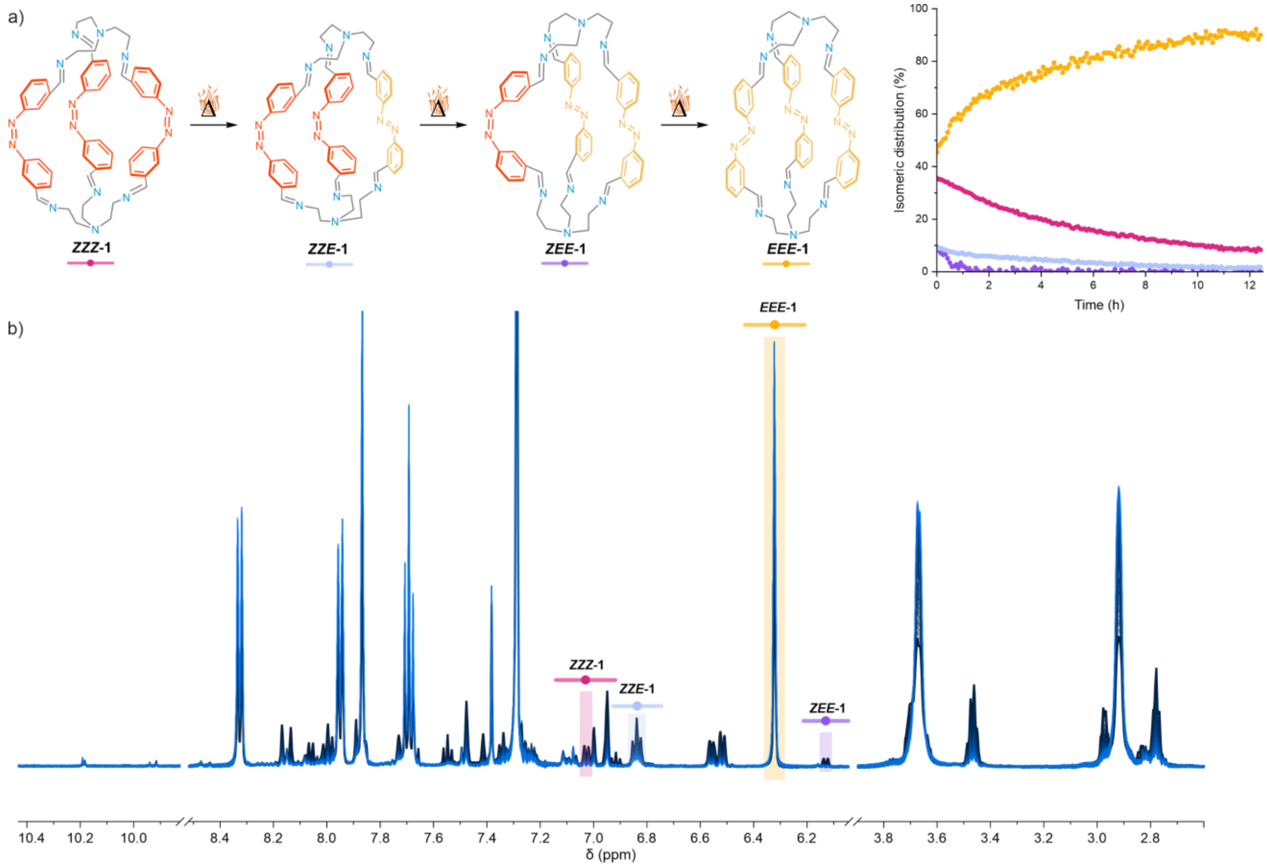


S3. Thermal isomerization process ***ZZZ*-1** ⟶ ***EEE*-1** (total concentration of **1 =** 1 mM) at 55 °C over 12.5 h. **a**, Kinetic traces of the evolutions of the different isomers of **1**. **b**, Evolution of the $^{1}$H NMR (500 MHz, $CDCl_3$, TFA 5 µM, 25 °C) spectra (from black to blue) during *in-situ* thermal isomerization of **1**. The signals used to follow the kinetic traces are displayed with their respective color code. Supporting video 2 illustrates this process.

## Macrocycle *EE*-3 self-assembly and switching

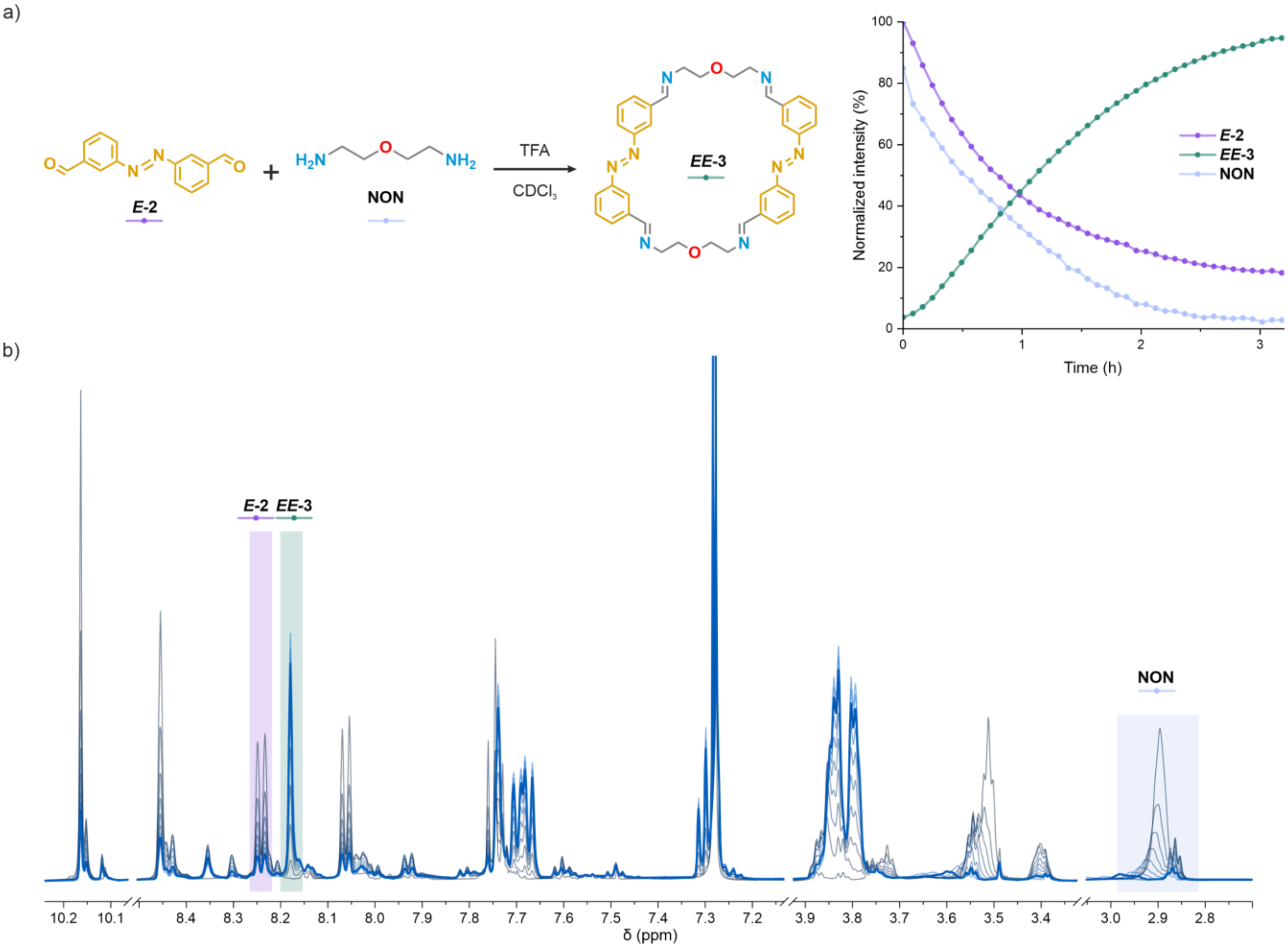


S4. Self-assembly process of ***E*-2** (3.3 mM) and **NON** (3.0 mM) into ***EE*-3** at 25 °C over 3.5 h. **a**, Kinetic traces of ***E*-2**, **NON** and ***EE-3*** during the self-assembly process. **b**, Evolution of the $^{1}$H NMR (500 MHz, $CDCl_3$, TFA 5 µM, 25 °C) spectra (from black to blue) during *in-situ* self-assembly of ***EE-3***. The signals used to follow the kinetic traces are displayed with their respective color code.

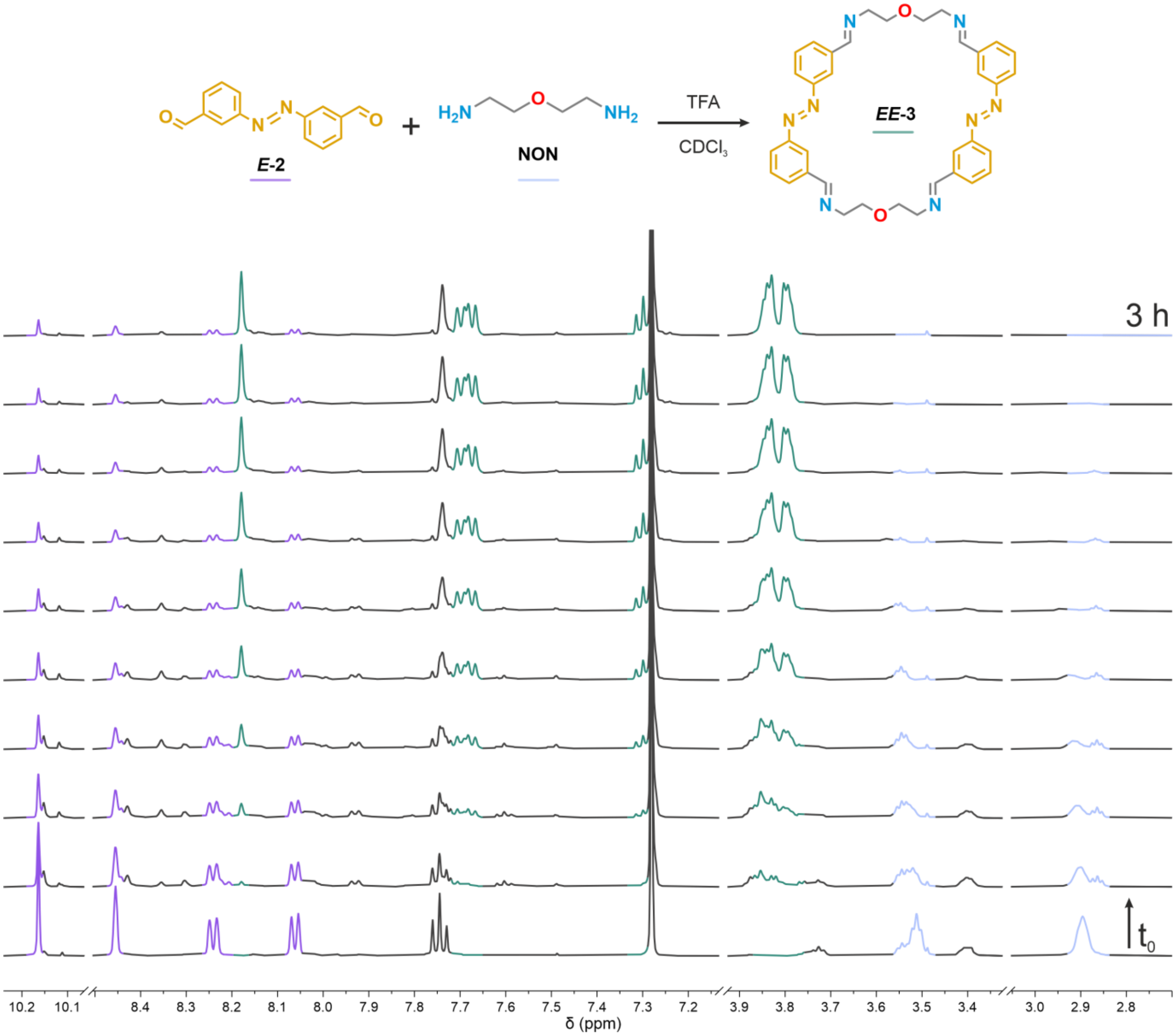


S5. [1]H NMR (500 MHz, $CDCl_3$, TFA 5 µM, 25 °C) stacked spectra of the self-assembly process ***E*-2** (3.3 mM) and **NON** (3.0 mM) into ***EE*-3** at 25 °C 3.5 h. The spectra evolution is shown from bottom to top.

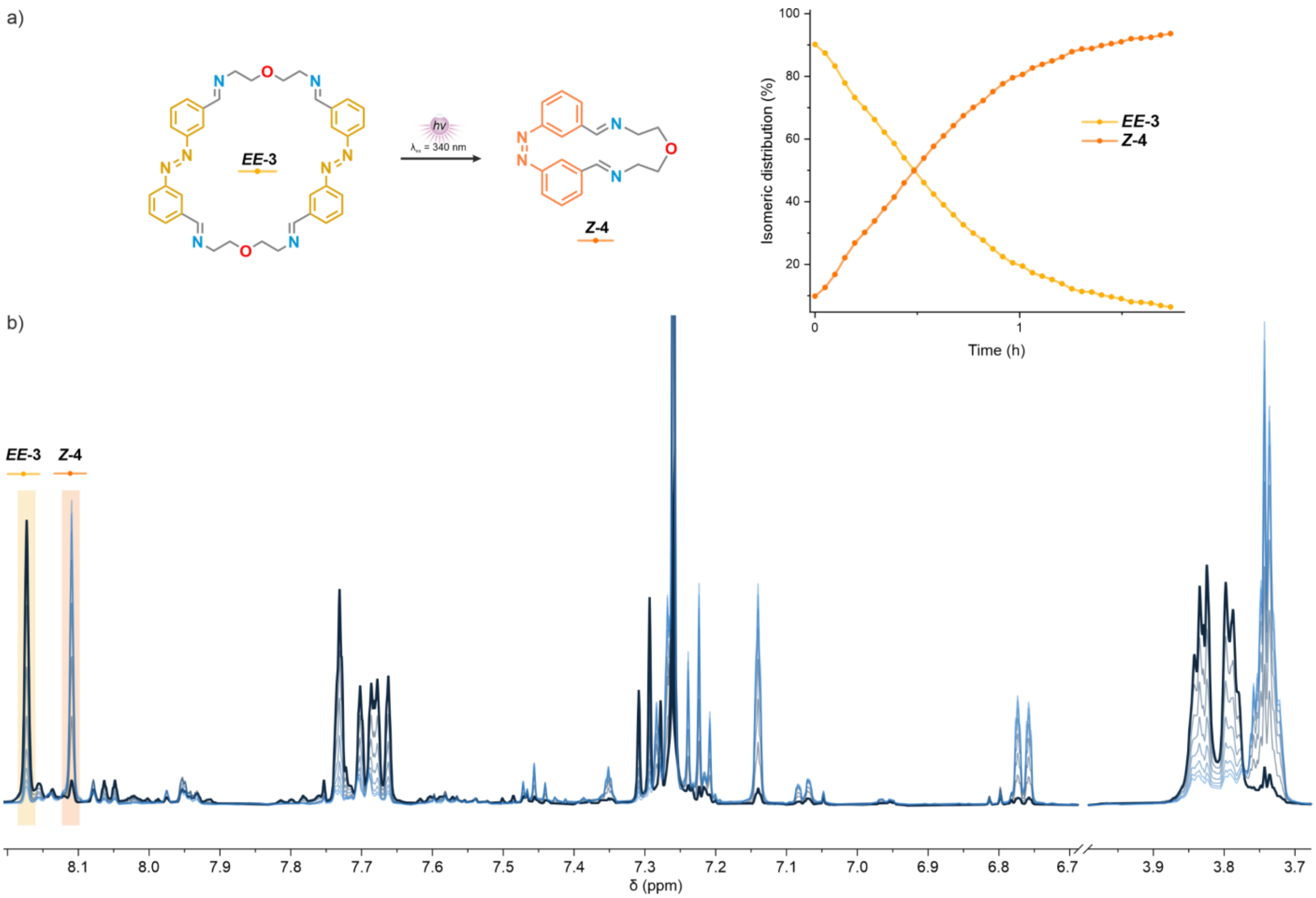


S6. Photoisomerization and ring contraction process ***EE*-3** ⟶ ***Z*-4** (1.5 mM) under UV light irradiation ($\lambda_{irr}$ = 340 nm) over 1.8 h. **a**, Kinetic traces of the evolutions of ***EE*-3** and ***Z*-4**. **b**, Evolution of the $^{1}$H NMR (500 MHz, $CDCl_3$, TFA 5 µM, 25 °C) spectra (from black to blue) during *in-situ* irradiation of ***EE*-3** ⟶ ***Z*-4**. The signals used to follow the kinetic traces are displayed with their respective color code.

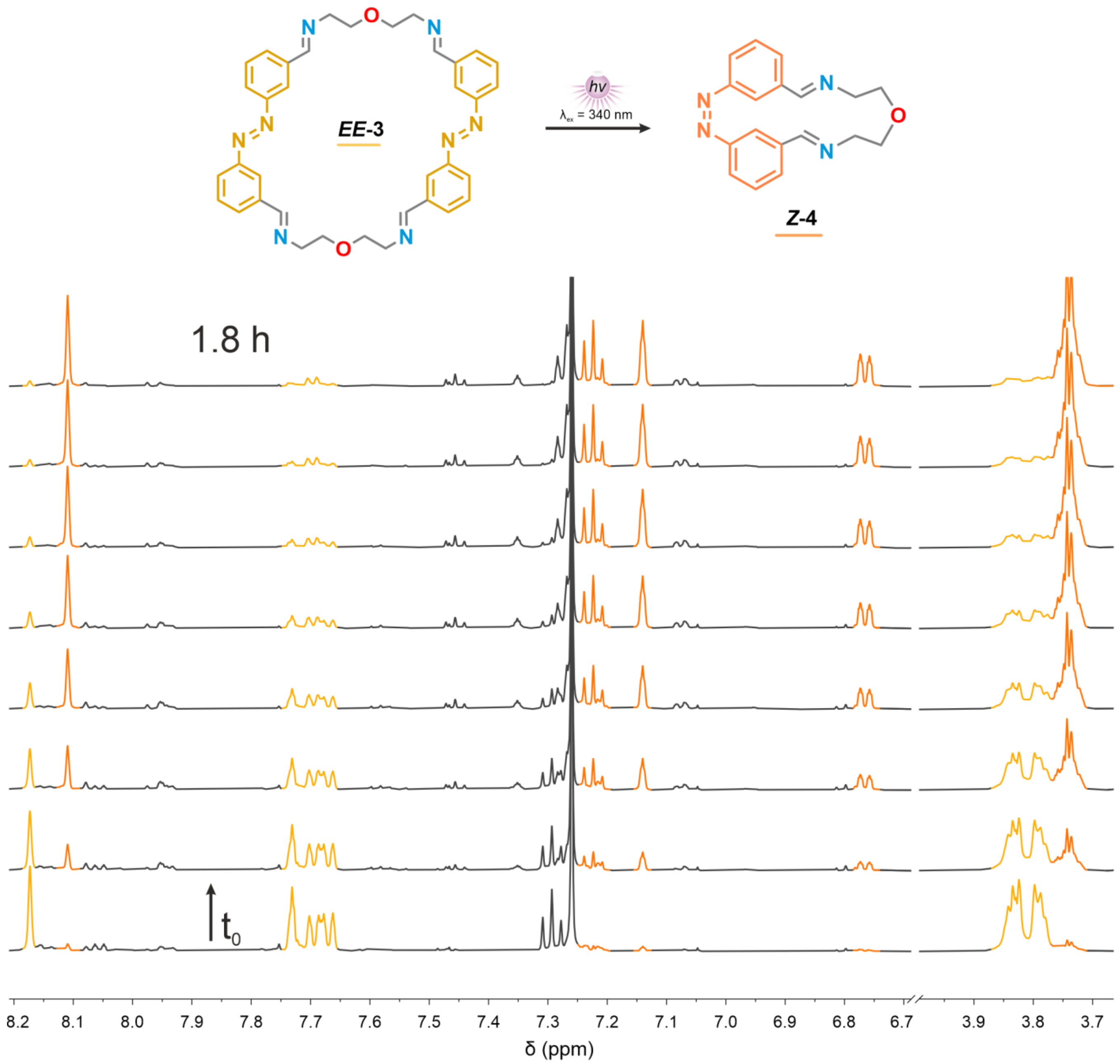


S7. $^{1}$H NMR (500 MHz, $CDCl_3$, TFA 5 µM, 25 °C) stacked spectra of the photoisomerization and ring contraction process ***EE*-3** ⟶ ***Z*-4** (1.5 mM) over 1.8 h. The spectra evolution is shown from bottom to top.

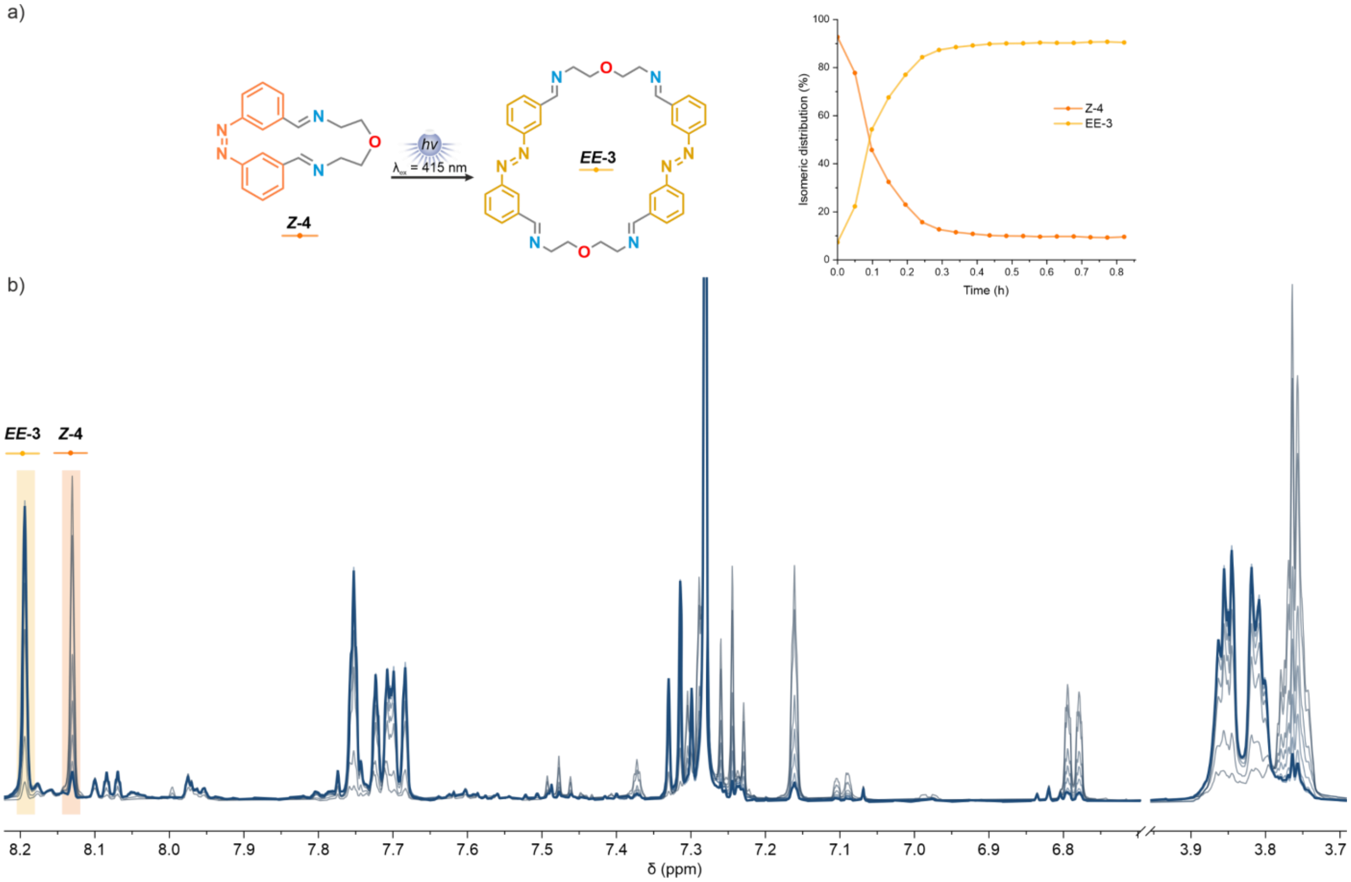


S8. Photoisomerization and ring expansion process ***Z*-4** ⟶ ***EE*-3** (1.5 mM) under visible light irradiation ($\lambda_{irr}$ = 420 nm) over 50 min. **a**, Kinetic traces of the evolutions of ***Z*-4** and ***EE*-3**. **b**, Evolution of the $^1$H NMR (500 MHz, $CDCl_3$, TFA 5 µM, 25 °C) spectra (from black to blue) during *in-situ* irradiation of ***Z*-4** ⟶ ***EE*-3**. The signals used to follow the kinetic traces are displayed with their respective color code.

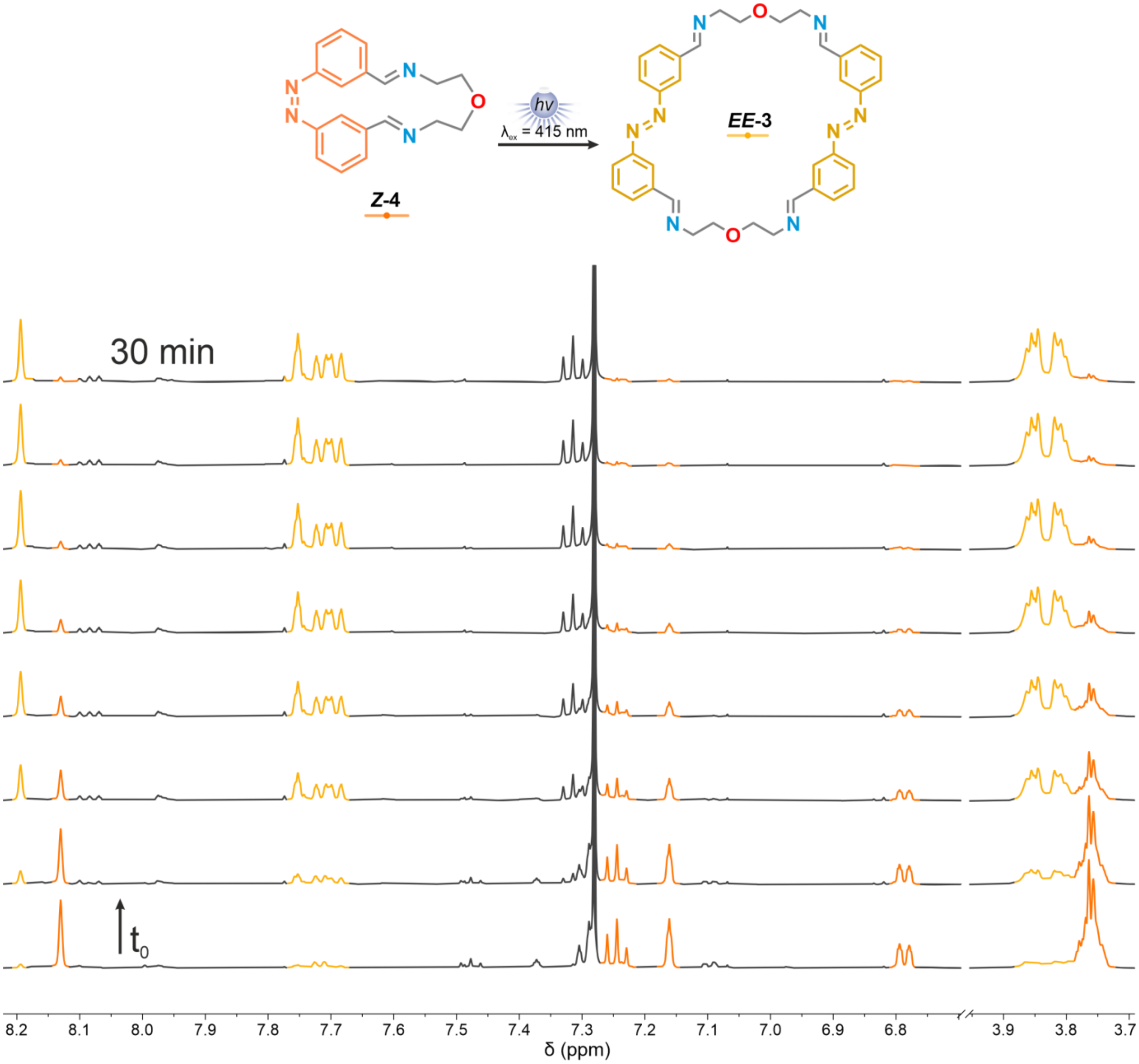


S9. $^1$H NMR (500 MHz, $CDCl_3$, TFA 5 µM, 25 °C) stacked spectra of the photoisomerization and ring contraction process ***Z*-4** ⟶ ***EE*-3** (1.5 mM) over 50 min. The spectra evolution is shown from bottom to top.

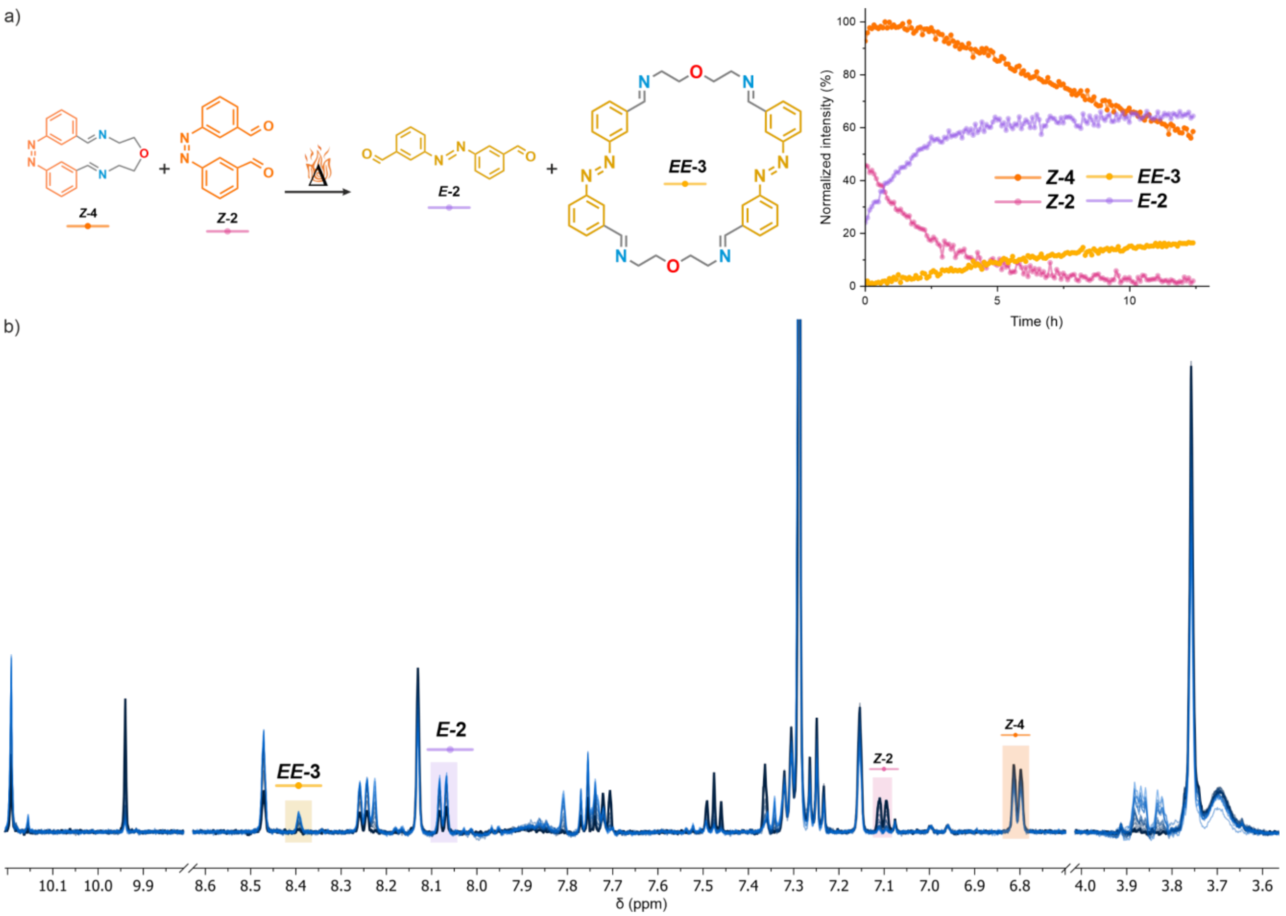


S10. Evolution of the kinetics of the thermal isomerization and ring expansion process ***Z*-4** ⟶ ***EE*-3** (1.5 mM) over 15 h. **a**, Kinetic traces of the evolutions of ***Z*-4** and ***EE*-3**. **b**, Evolution of the $^{1}$H NMR (500 MHz, $CDCl_3$, TFA 5 µM, 55 °C) spectra (from black to blue) during the thermal isomerization of ***Z*-4** ⟶ ***EE*-3**. The signals used to follow the kinetic traces are displayed with their respective color code. Notes: During the heating process, ***Z*-2** is generated as a result of the hydrolysis of ***Z*-4** at elevated temperatures. The same process is depicted in figure S11 in a stacked spectra presentation.

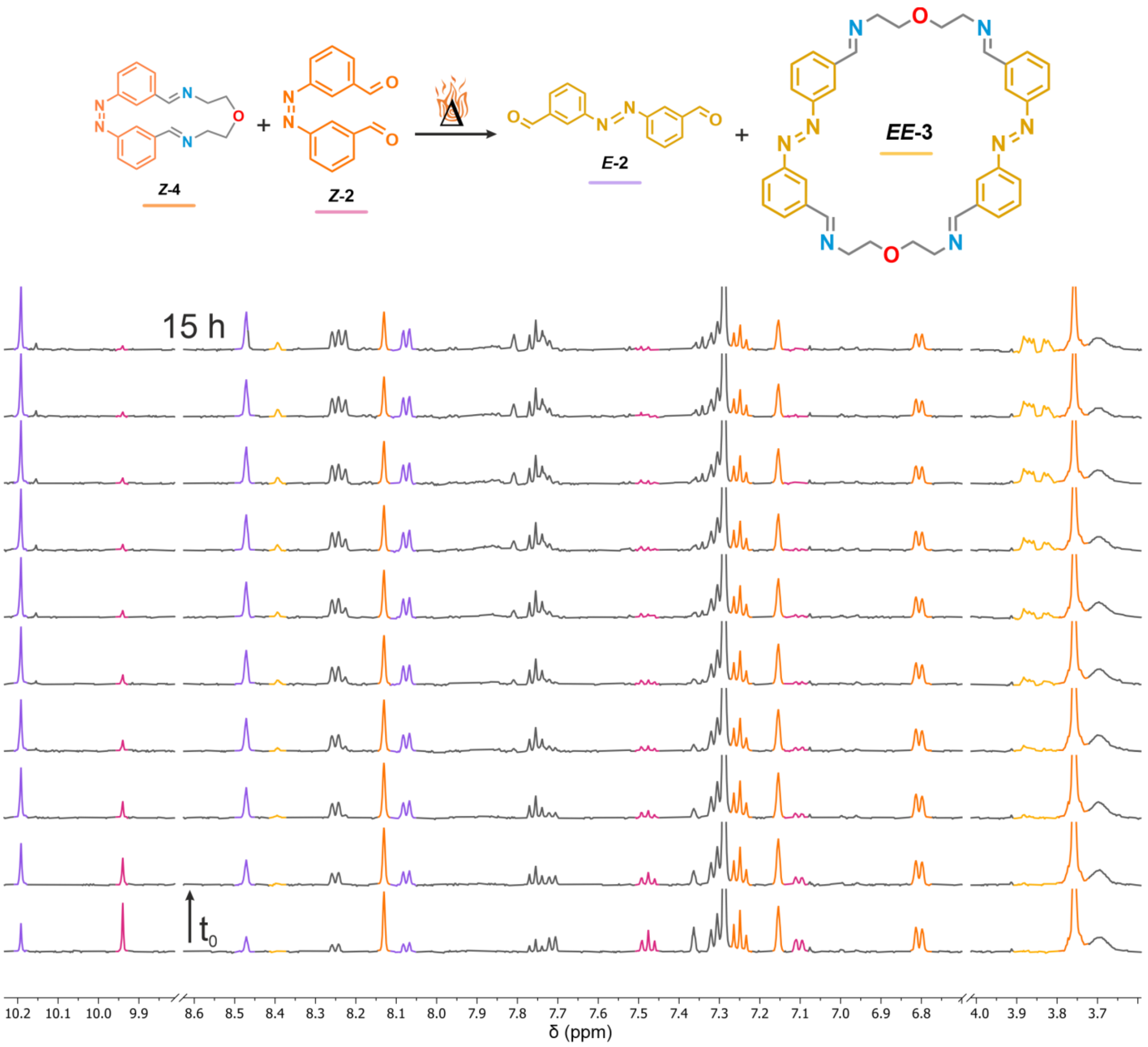


S11. $^{1}H$ NMR (500 MHz, $CDCl_3$, TFA 5 µM, 55 °C) stacked spectra of the thermal isomerization and ring expansion process ***Z*-4** ⟶ ***EE*-3** (1.5 mM) over 15 h. Hydrolysis is observed at this temperature, thus the ***Z*-2** → ***E*-2** isomerization is also observed. The spectra evolution is shown from bottom to top. Notes: During the heating process, ***Z*-2** is generated as a result of the hydrolysis of ***Z*-4** at elevated temperatures. The same process is depicted in figure S10 in an overlay spectra presentation.

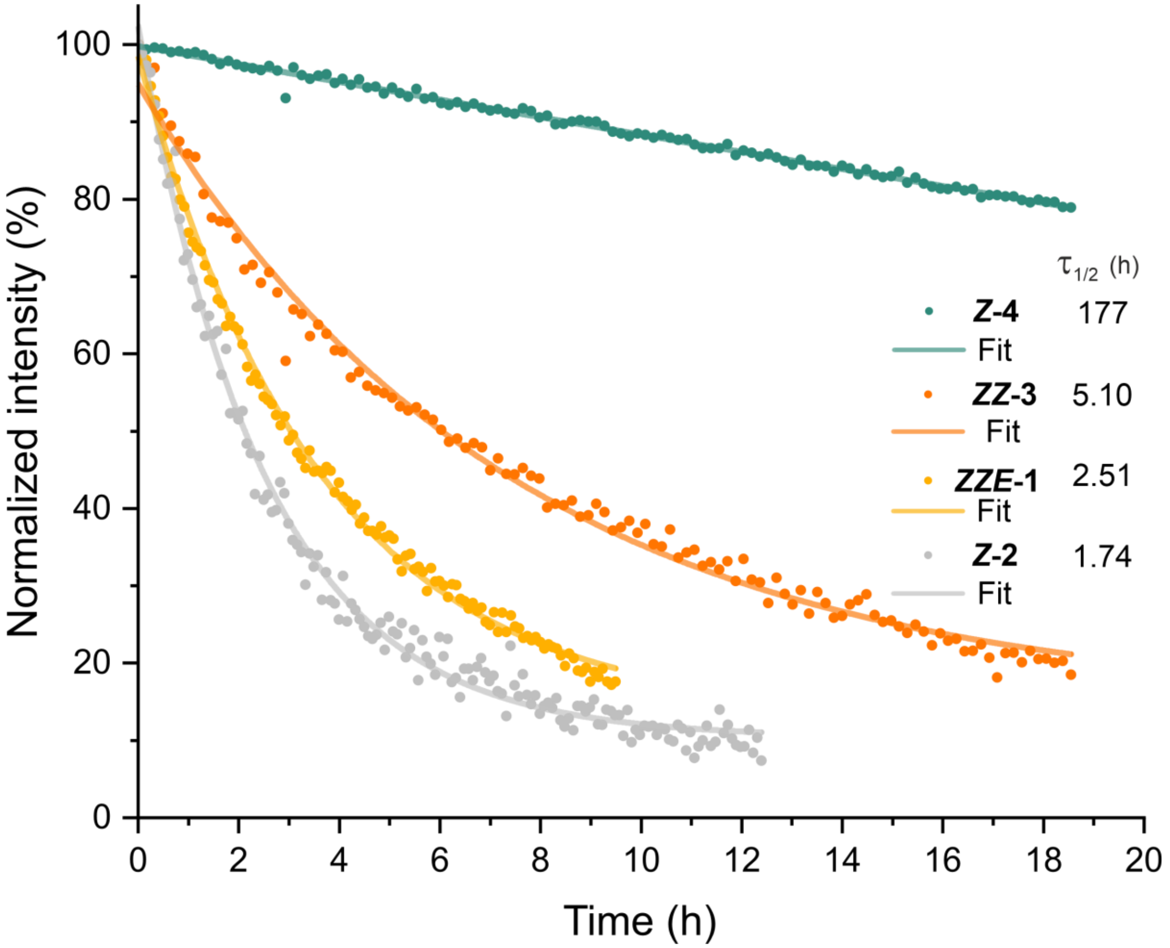


S12. Thermal decays at 55 °C on $CDCl_3$ of the different ***Z*** isomers of macrocycles **4** (blue) and **3** (orange), cage **1** (yellow, this isomer corresponds to ***ZZE*-1**) and aldehyde **2.** The data shown in this picture was taken from the data shown in figure S3 for cage ***ZZE*-1**, figure S11 for ***E*-2** and ***Z*-4** and figure S20 for ***ZZ*-3**.

## Cage-to-macrocycle transformation

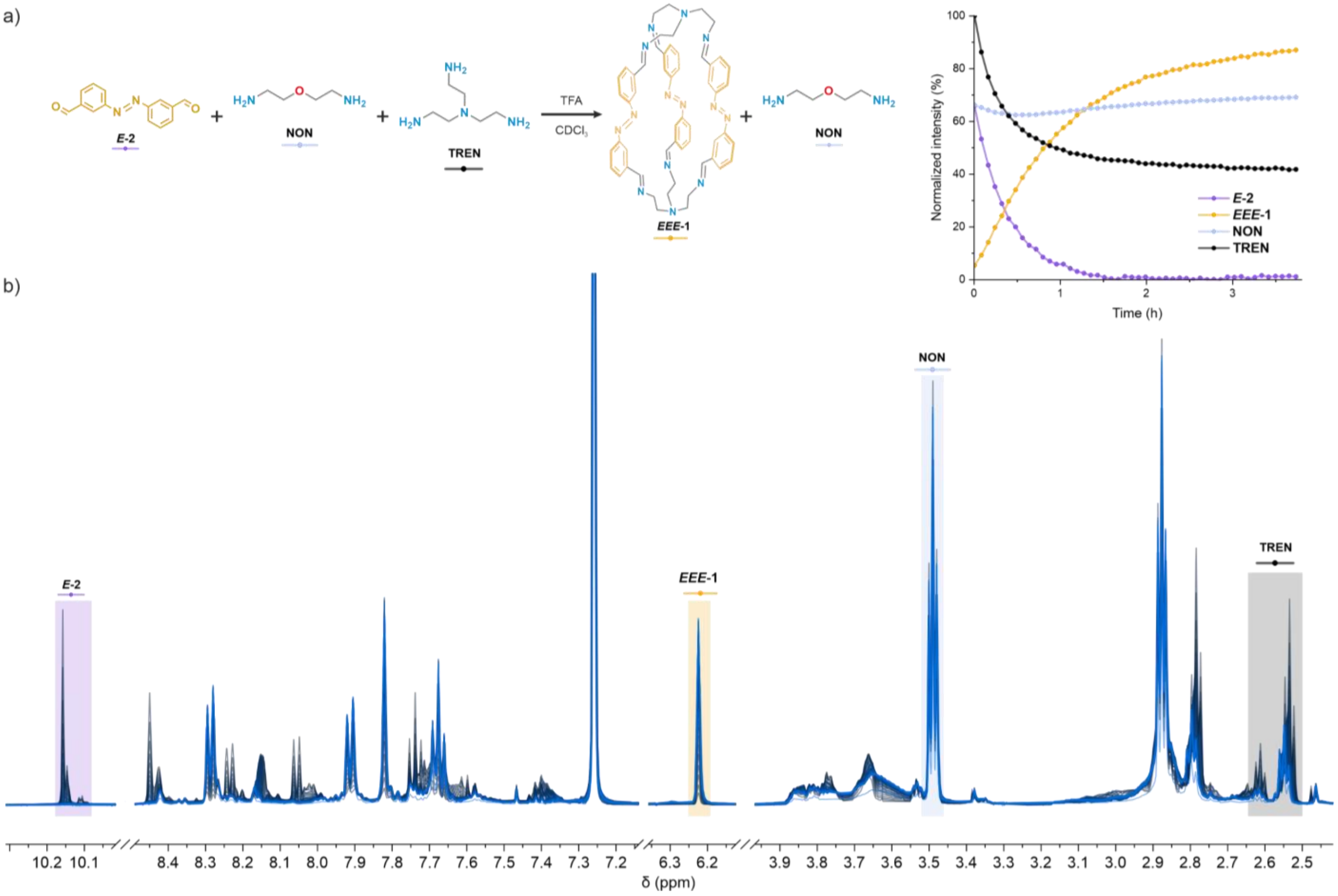


S13. Self-assembly process between ***E*-2** (3 mM), **TREN** (2 mM), and **NON** (3 mM) over 4 h. **a**, Kinetic traces of the evolutions of the self-sorting process. **b**, Evolution of the $^{1}$H NMR (500 MHz, $CDCl_3$, TFA 5 µM, 25 °C) spectra (from black to blue) during the self-sorting process. The signals used to follow the kinetic traces are displayed with their respective color code. Supporting video 4 illustrates this process. Note: The same process is depicted in figure S14 in a stacked spectra presentation.

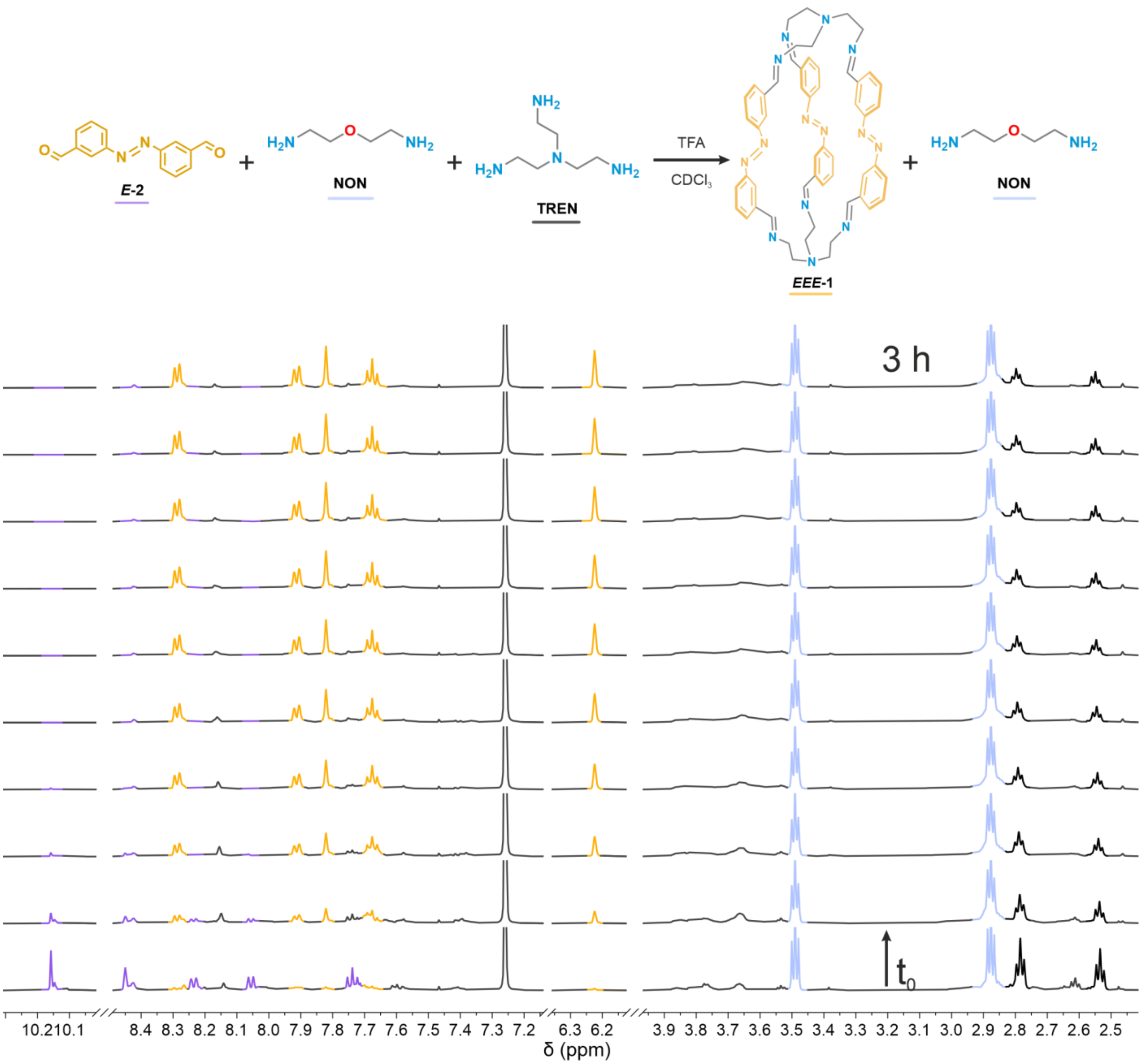


S14. [1]H NMR (500 MHz, $CDCl_3$, TFA 5 µM, 25 °C) stacked spectra of the self-sorting process between ***E*-2** (3 mM), **TREN** (2 mM), and **NON** (3 mM) over 4 h. The spectra evolution is shown from bottom to top. Supporting video 3 illustrates this process. Note: The same process is depicted in figure S13 in an overlayed spectra presentation.

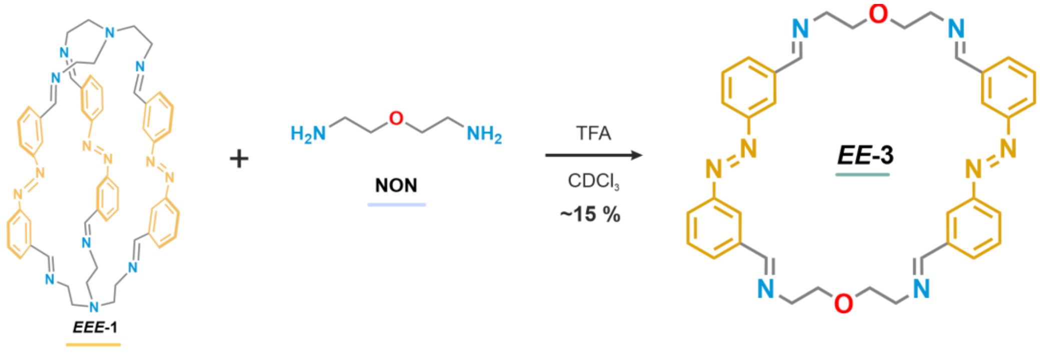


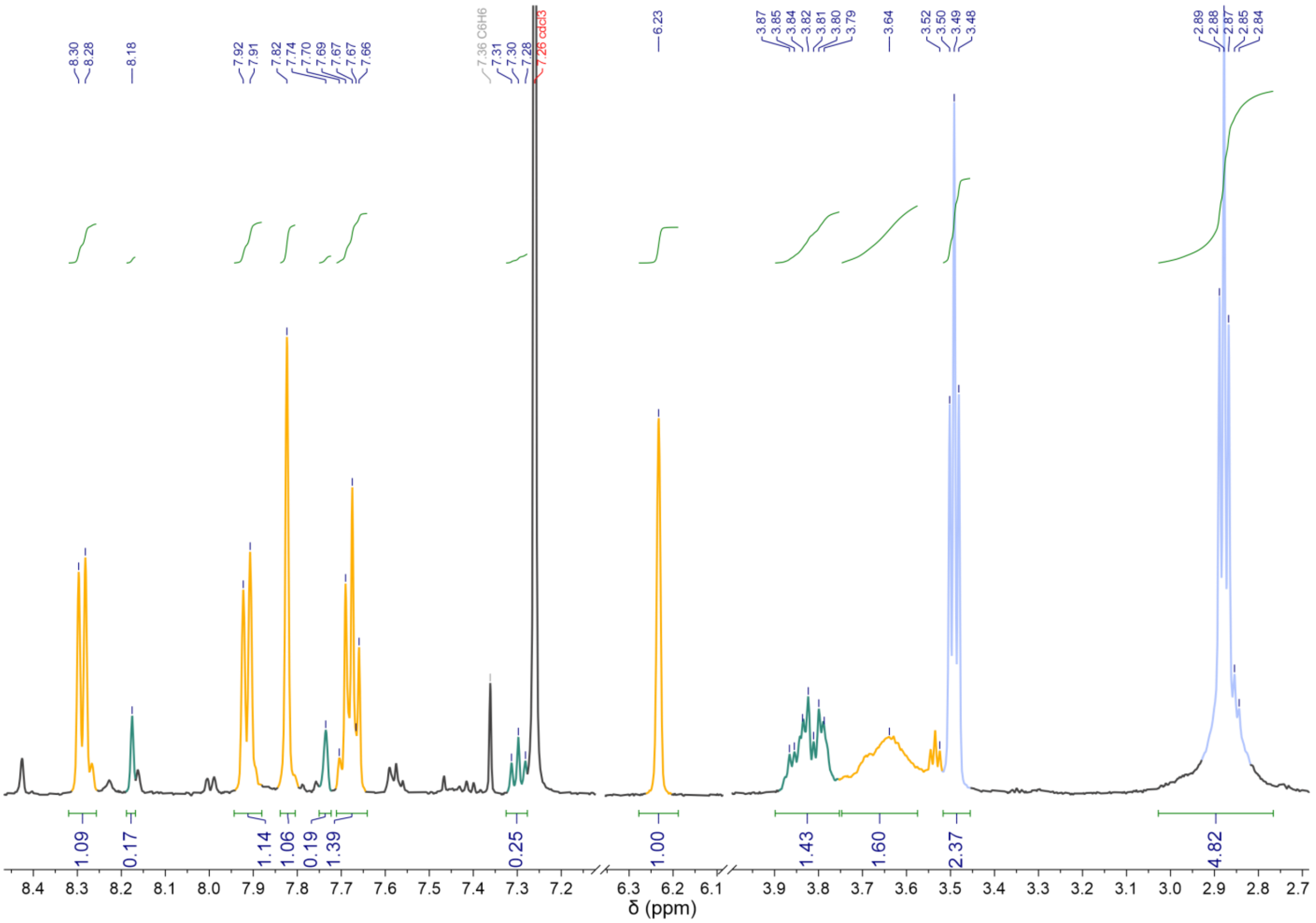


S15. [1]H NMR of ***EEE*-1** (3 mM) in $CDCl_3$ with TFA 5 µM after 24 h equilibration with **NON** (3 mM) at 25 °C.

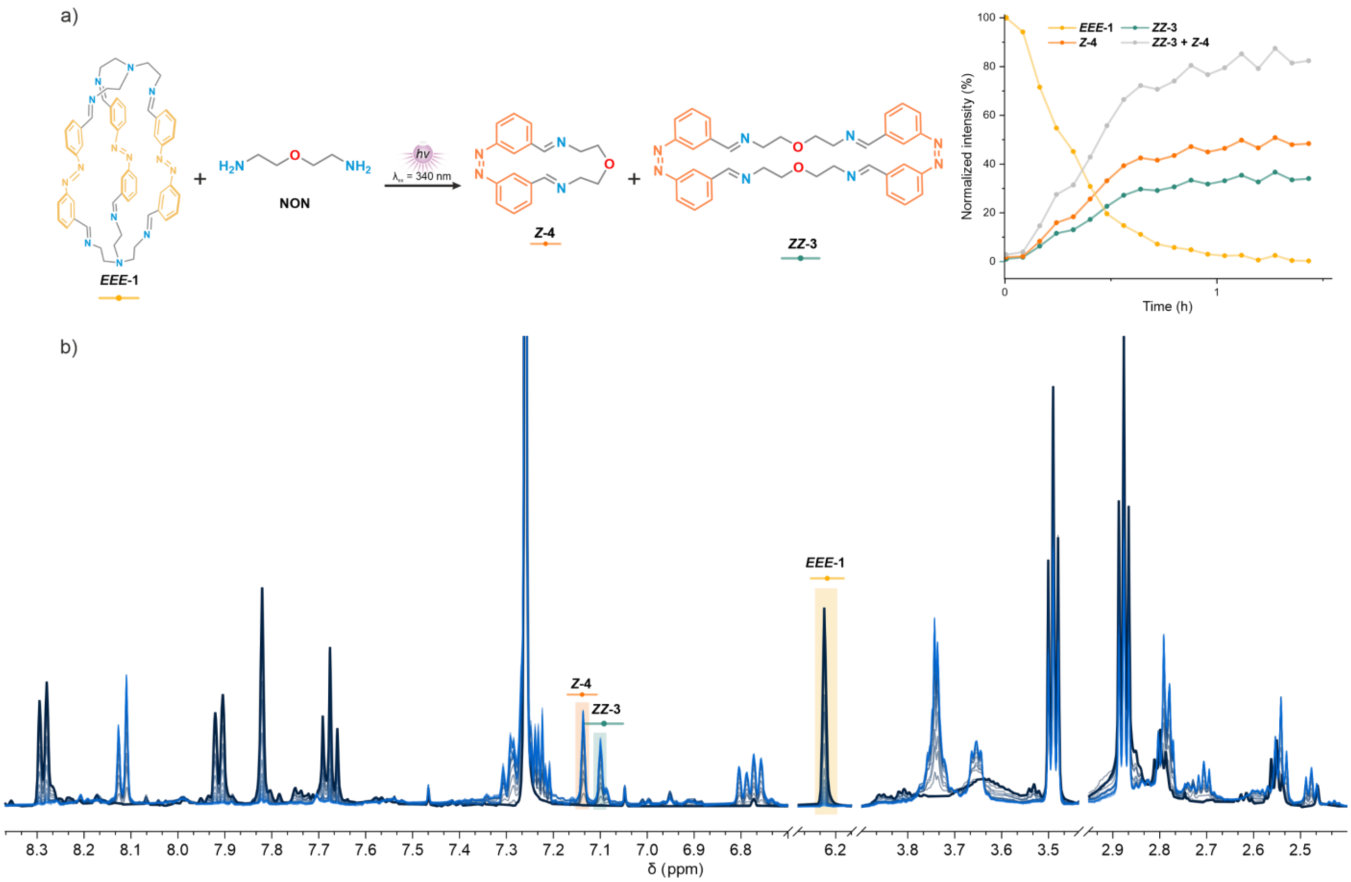


S16. Light-fueled cage-to-macrocycle transformation ***EEE*-1** + **NON** → ***Z*-3** + ***ZZ*-4** over 1.8 h. **a**, Kinetic traces of the process. **b**, Evolution of the $^{1}$H NMR (500 MHz, $CDCl_3$, TFA 5 µM, 25 °C) spectra (from black to blue) during the cage-to-macrocycle transformation. Supporting video 3 illustrates this process. Note: The same process is depicted in figure S17 in a stacked spectra presentation.

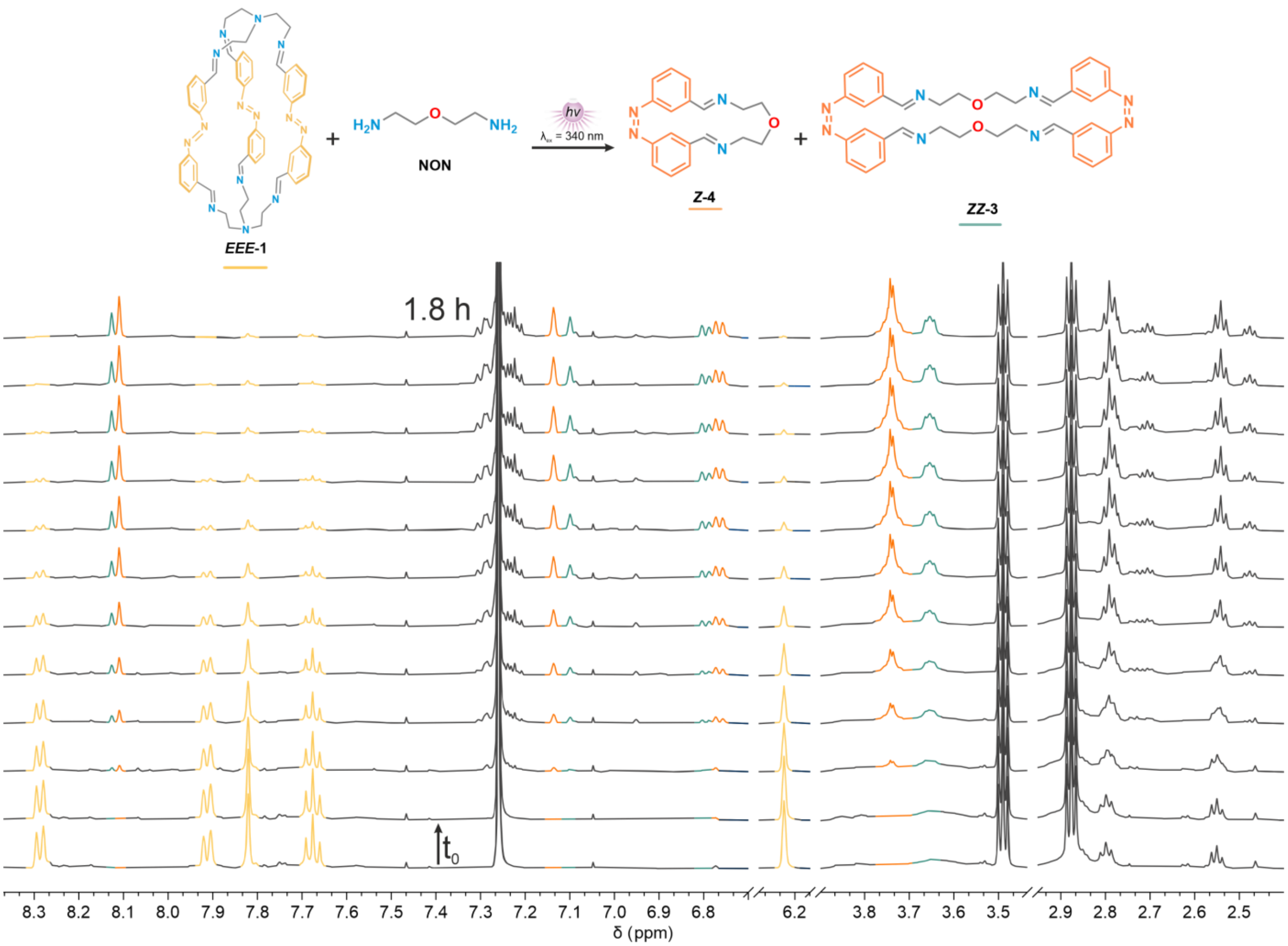


S17. $^1$H NMR (500 MHz, $CDCl_3$, TFA 5 µM, 25 °C) stacked spectra of the light-fueled cage-to-macrocycle transformation ***EEE*-1** + **NON** → ***Z*-3** + ***ZZ*-4** over 1.8 h. The spectra evolution is shown from bottom to top. Supporting video 3 illustrates this process. Note: The same process is depicted in figure S16 in an overlayed spectra presentation.

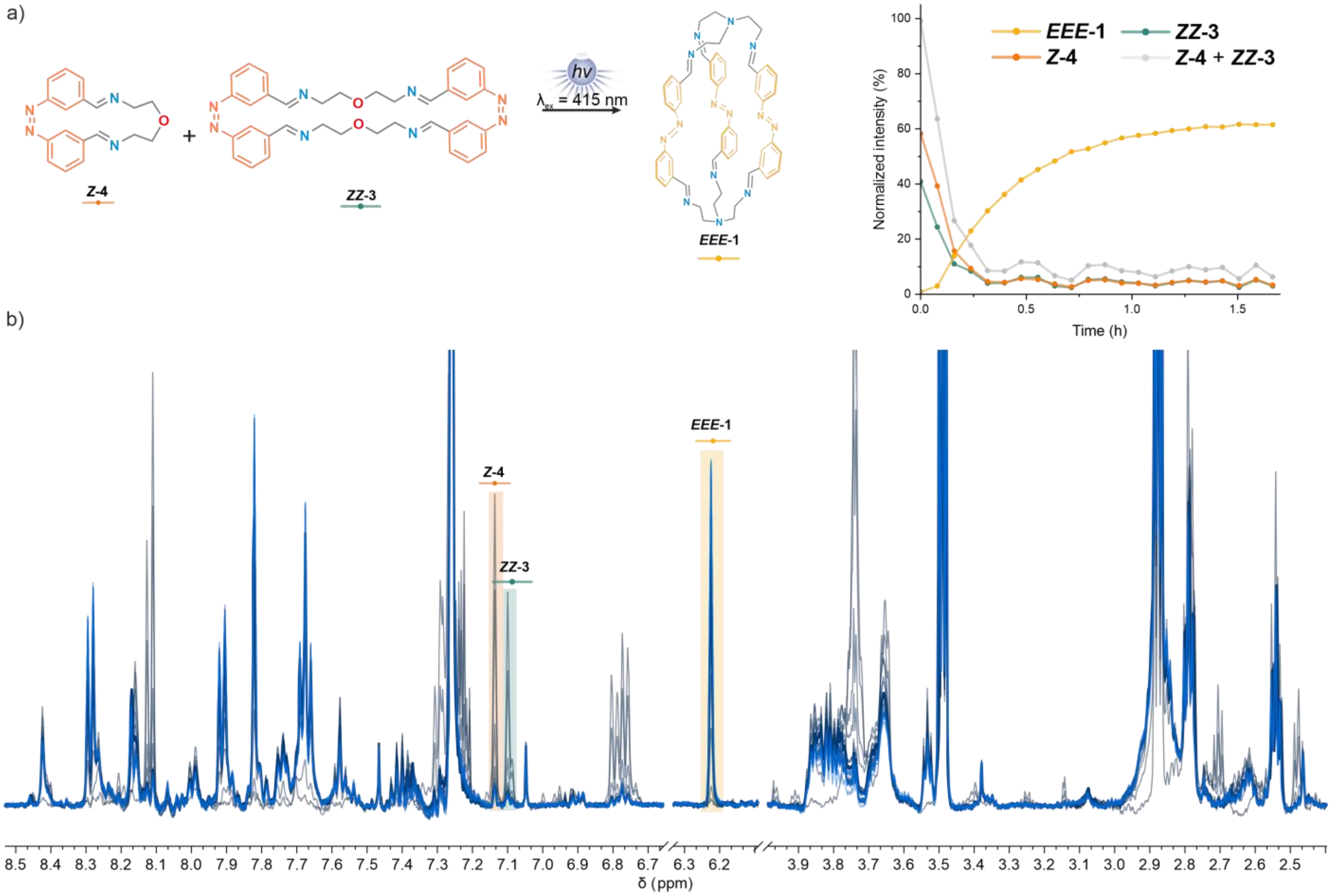


S18. Macrocycle-to-cage transformation ***Z*-3** + ***ZZ*-4** → ***EEE*-1** over 1.8 h. **a**, Kinetic traces of the process. **b**, Evolution of the [1]H NMR (500 MHz, $CDCl_3$, TFA 5 µM, 25 °C) spectra (from black to blue) during the cage-to-macrocycle transformation. Supporting video 3 illustrates this process. Note: The same process is depicted in figure S19 in a stacked spectra presentation.

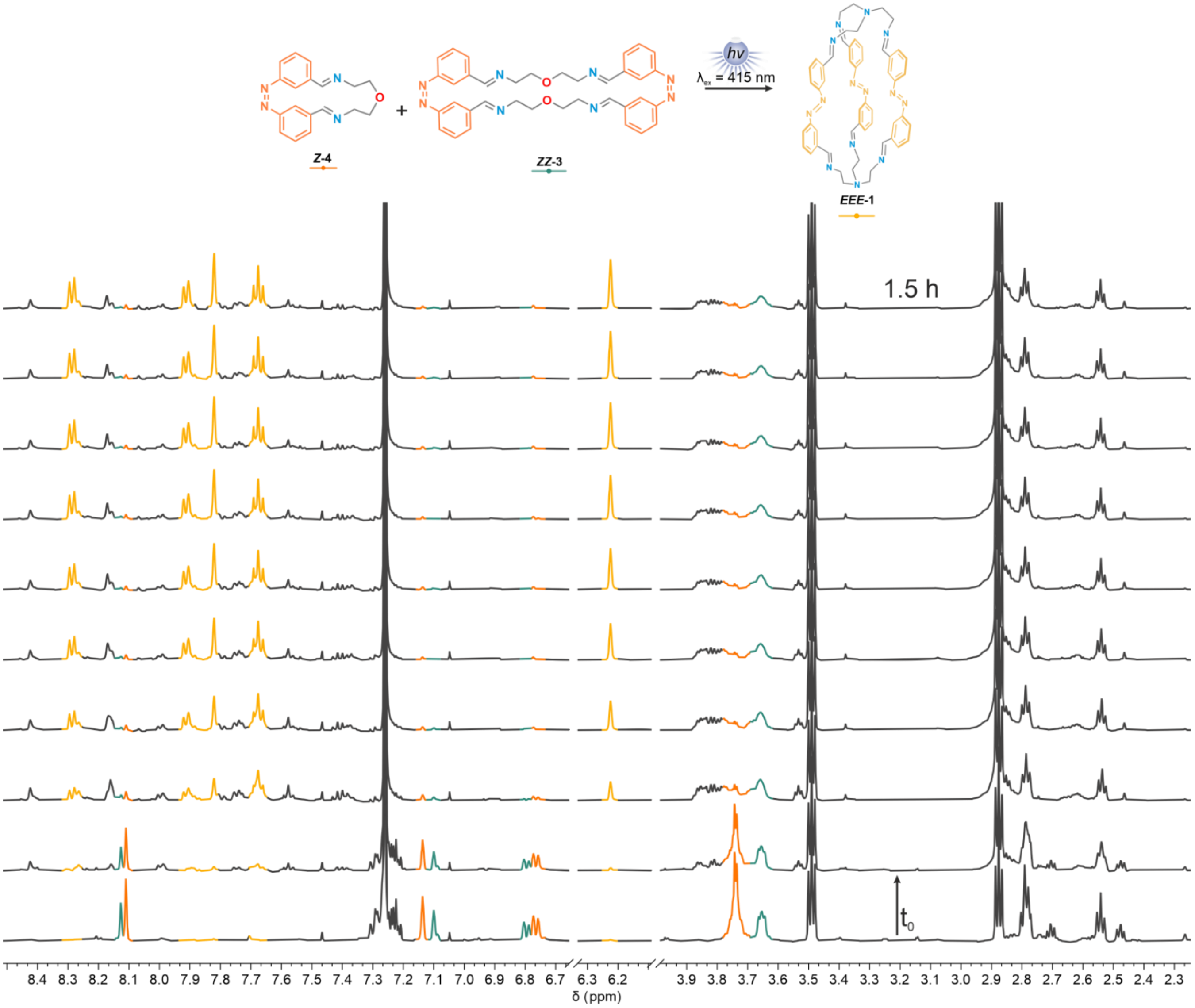


S19. $^{1}H$ NMR (500 MHz, $CDCl_3$, TFA 5 µM, 25 °C) stacked spectra of the macrocycle-to-cage transformation ***Z*-3** + ***ZZ*-4** → ***EEE*-1** over 1.8 h. The spectra evolution is shown from bottom to top. Supporting video 3 illustrates this process. Note: The same process is depicted in figure S18 in an overlayed spectra presentation.

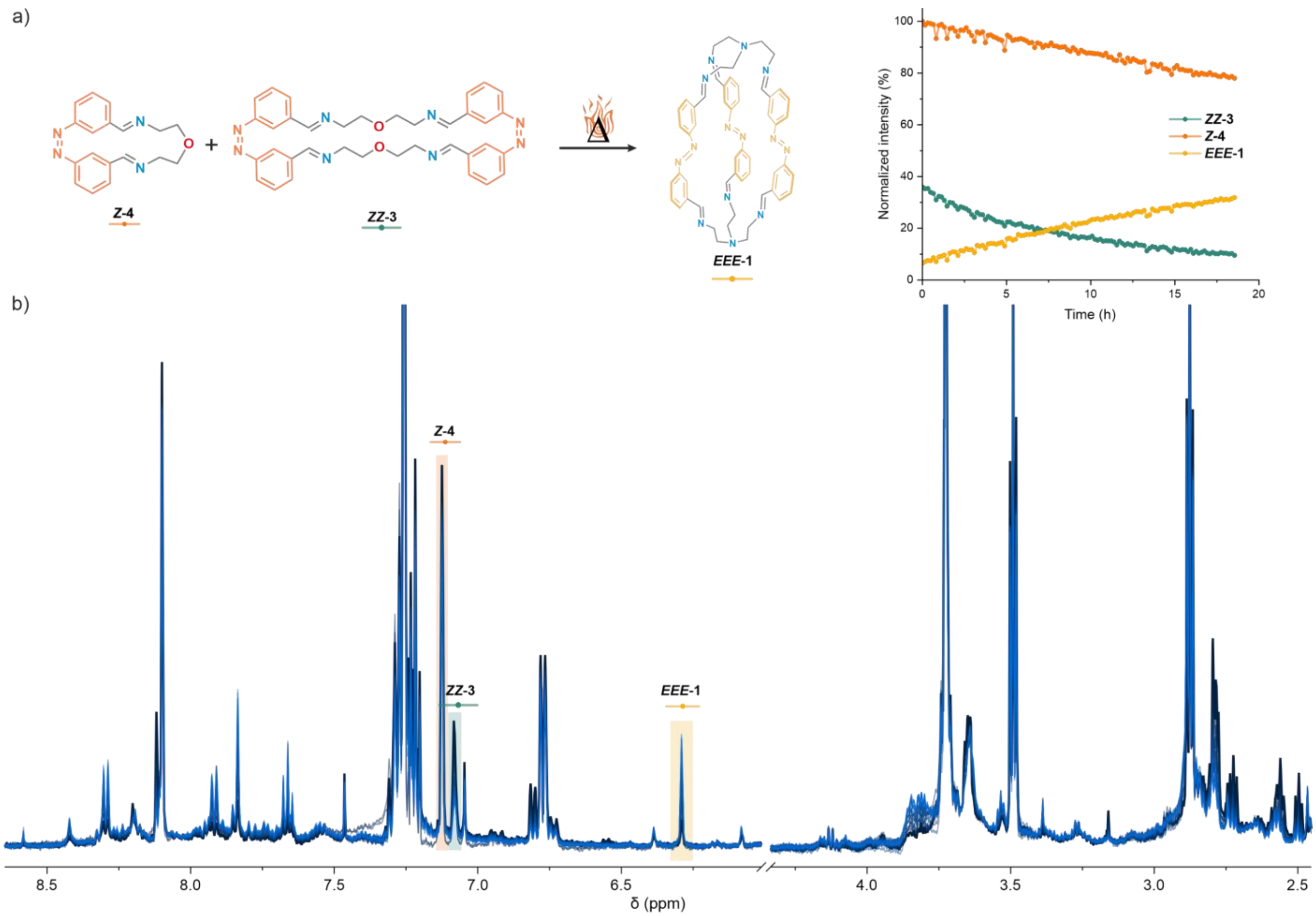


S20. Thermal macrocycle-to-cage transformation ***Z*-3** + ***ZZ*-4** → ***EEE*-1** + **NON** over 18h. **a**, Kinetic traces of the thermal process. **b**, Evolution of the $^{1}H$ NMR (500 MHz, $CDCl_3$, TFA 5 µM, 55 °C) spectra (from black to blue) during thermal process. The signals used to follow the kinetic traces are displayed with their respective color code. Note: The same process is depicted in figure S21 in a stacked spectra presentation.

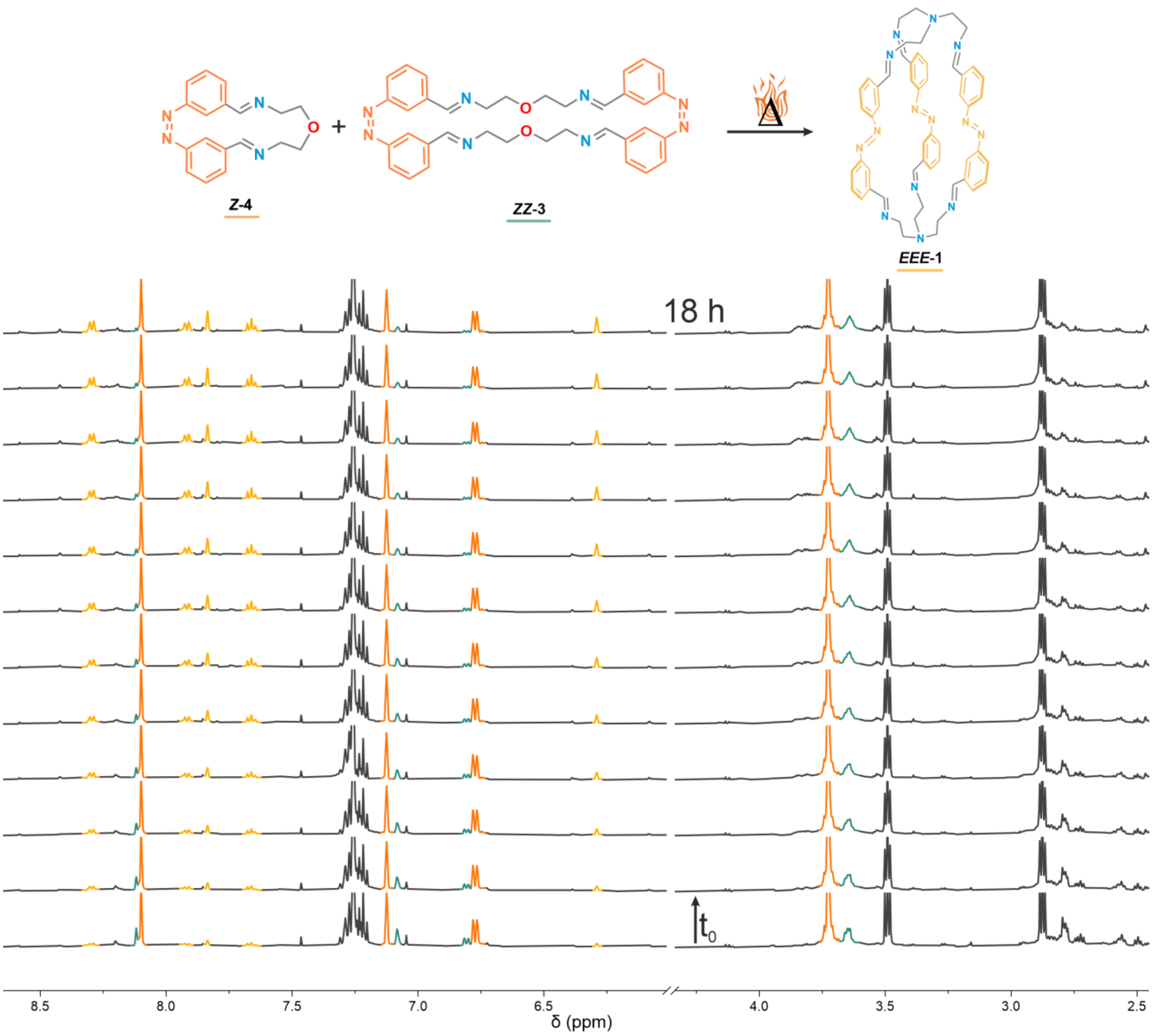


S21. $^{1}H$ NMR (500 MHz, $CDCl_3$, TFA 5 µM, 25 °C) stacked spectra of the thermal macrocycle-to-cage transformation ***Z*-3** + ***ZZ*-4** → ***EEE*-1** + **NON** over 18h. The spectra evolution is shown from bottom to top. Note: The same process is depicted in figure S20 in an overlayed spectra presentation.

## Self-assembly and switching of macrocycles 11 – 19.

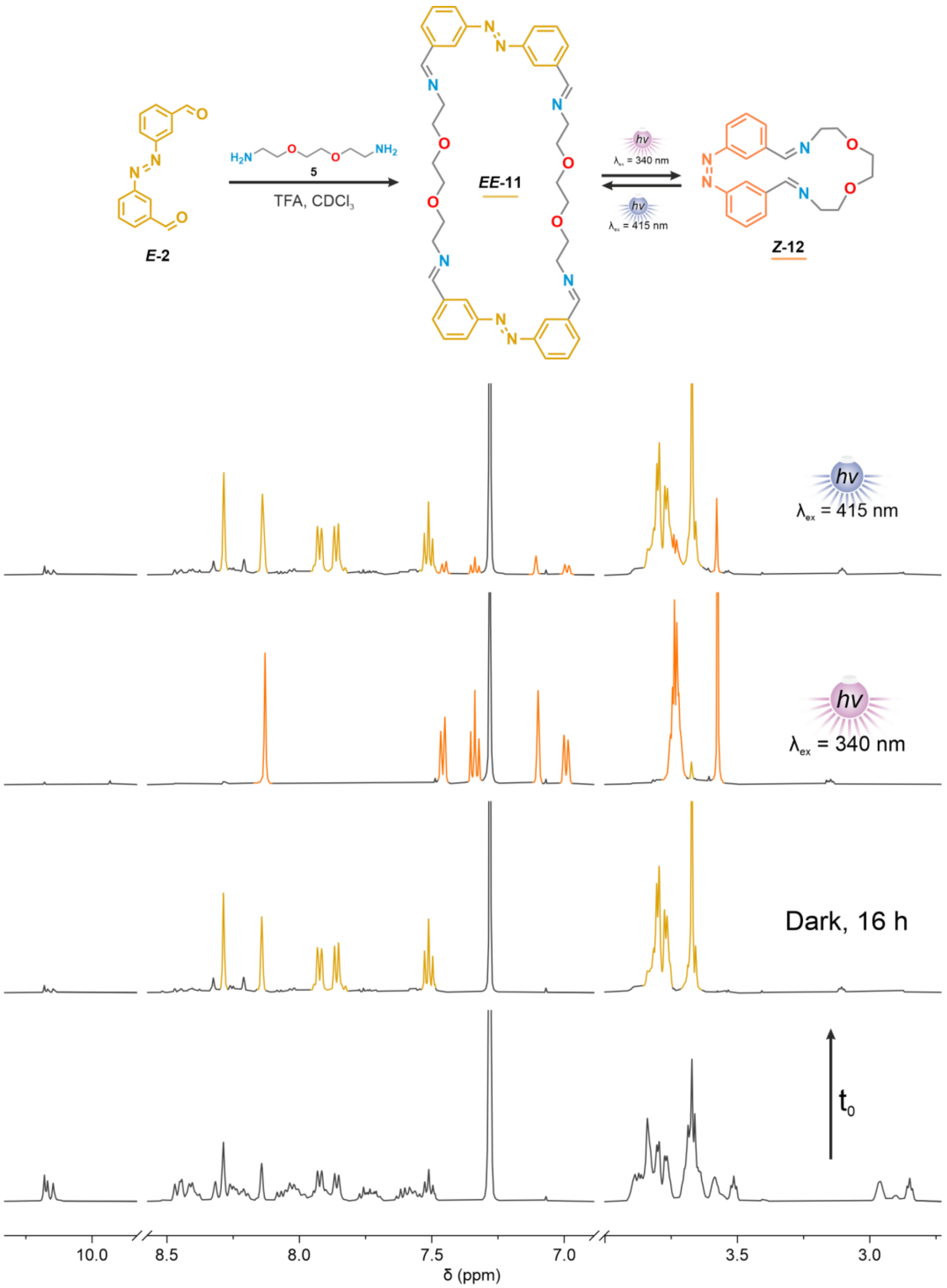


S22. [1]H NMR (500 MHz, $CDCl_3$, TFA 5 µM, 25 °C) stacked (from bottom to top) of the self-assembly of ***E*-2** (3 mM) + **5** (3mM) → ***EE*-11** immediately after mixing the components ($t_0$), followed by the self-assembled state in a after a period of 16 h in the dark. Next spectra show the photoisomerization and macrocycle-to-macrocycle transformation ***EE*-11** → ***Z*-12** of the self-assembled system by UV irradiation ($\lambda_{irr}$ = 340 nm). On the top, the ***Z*-12**→ ***EE*-11** photoisomerization and macrocycle-to-macrocycle transformation of the self-assembled system after visible irradiation ($\lambda_{irr}$ = 415 nm). Supporting video 6 illustrates this process. Supporting video 6 illustrates this process.

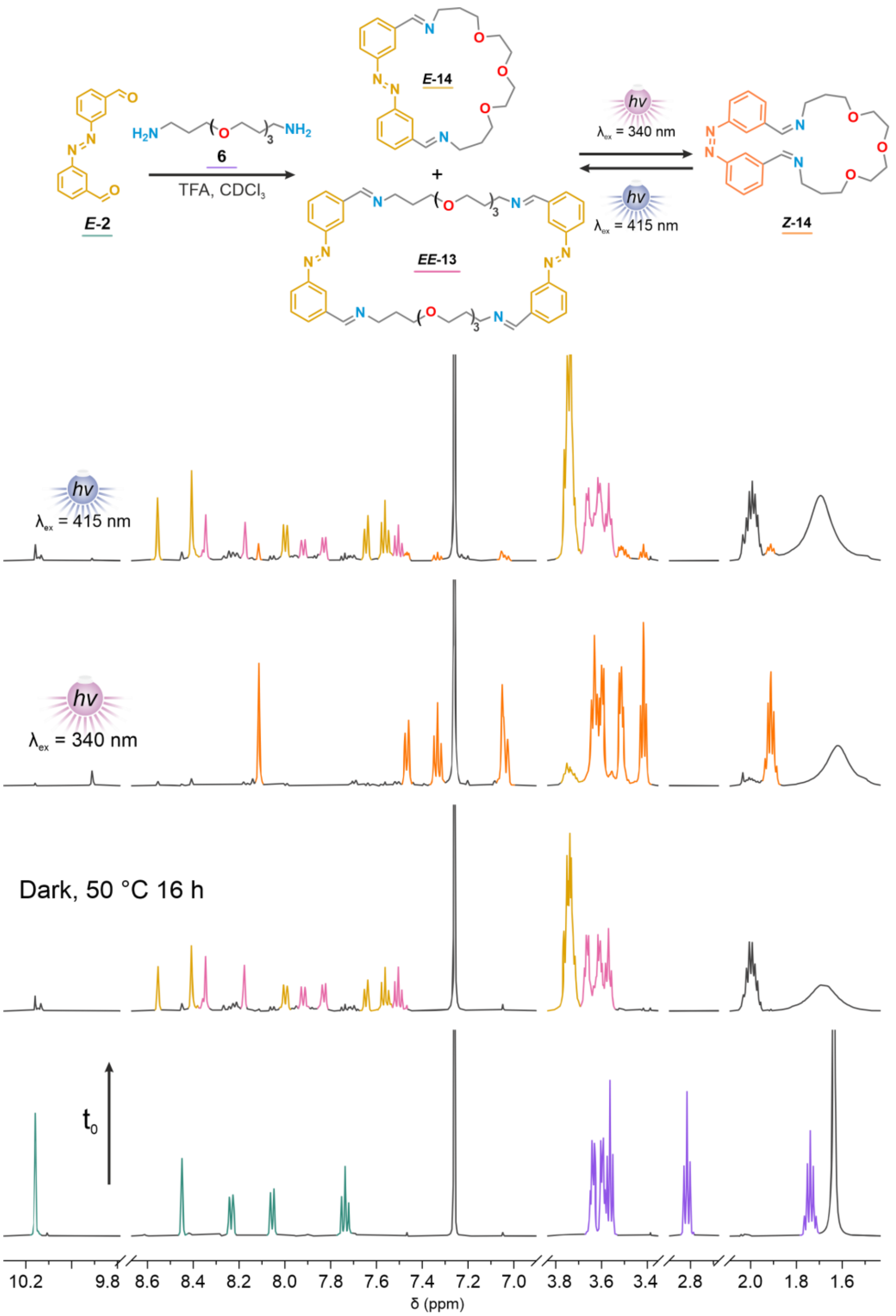


S23. $^{1}$H NMR (500 MHz, $CDCl_3$, TFA 5 µM, 25 °C) stacked (from bottom to top) of the self-assembly of ***E*-2** (3 mM) + **6** (3mM) → ***EE*-13** + ***E*-14** immediately after mixing the components ($t_0$), followed by the self-assembled state in a after a period of 16 h in the dark at 50 °C. Next spectra show the photoisomerization and macrocycle-to-macrocycle transformation ***EE*-13** + ***E*-14** → ***Z*-14** of the self-assembled system by UV irradiation ($\lambda_{irr}$ = 340 nm). On the top, the ***Z*-14**→ ***EE*-13** + ***E*-14** photoisomerization and macrocycle-to-macrocycle transformation of the self-assembled system after visible irradiation ($\lambda_{irr}$ = 415 nm) is shown. Supporting video 7 illustrates this process.

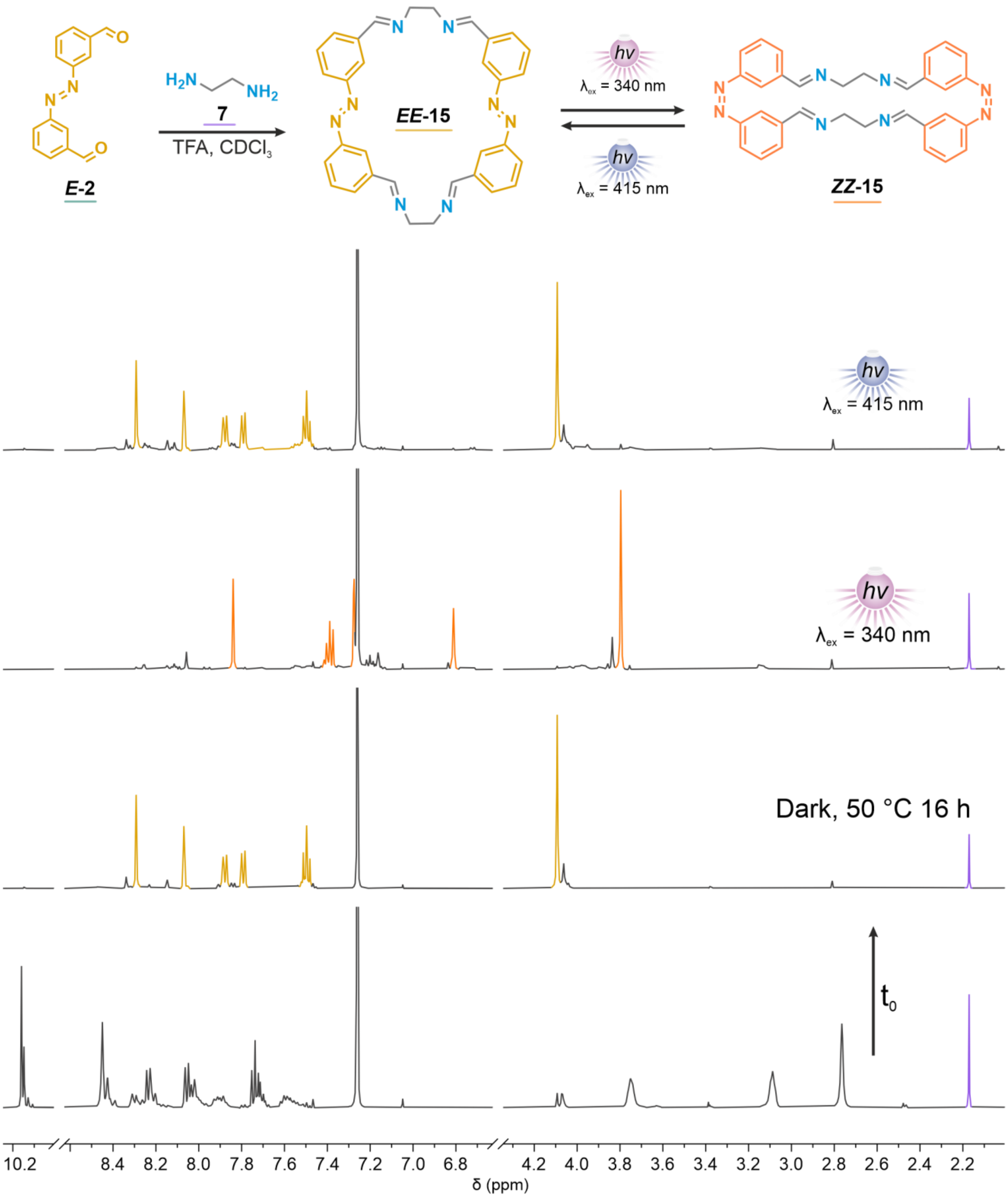


S24. $^{1}$H NMR (500 MHz, $CDCl_3$, TFA 5 µM, 25 °C) stacked (from bottom to top) of the self-assembly of ***E*-2** (3 mM) + **7** (3mM) → ***EE*-15** immediately after mixing the components ($t_0$), followed by the self-assembled state in a after a period of 16 h in the dark at 50 °C. Next spectra show the photoisomerization ***EE*-15** → ***ZZ*-15** of the self-assembled system by UV irradiation ($\lambda_{irr}$ = 340 nm). On the top, the ***ZZ*-15**→ ***EE*-15** photoisomerization of the self-assembled system after visible irradiation ($\lambda_{irr}$ = 415 nm) is shown. Supporting video 8 illustrates this process.

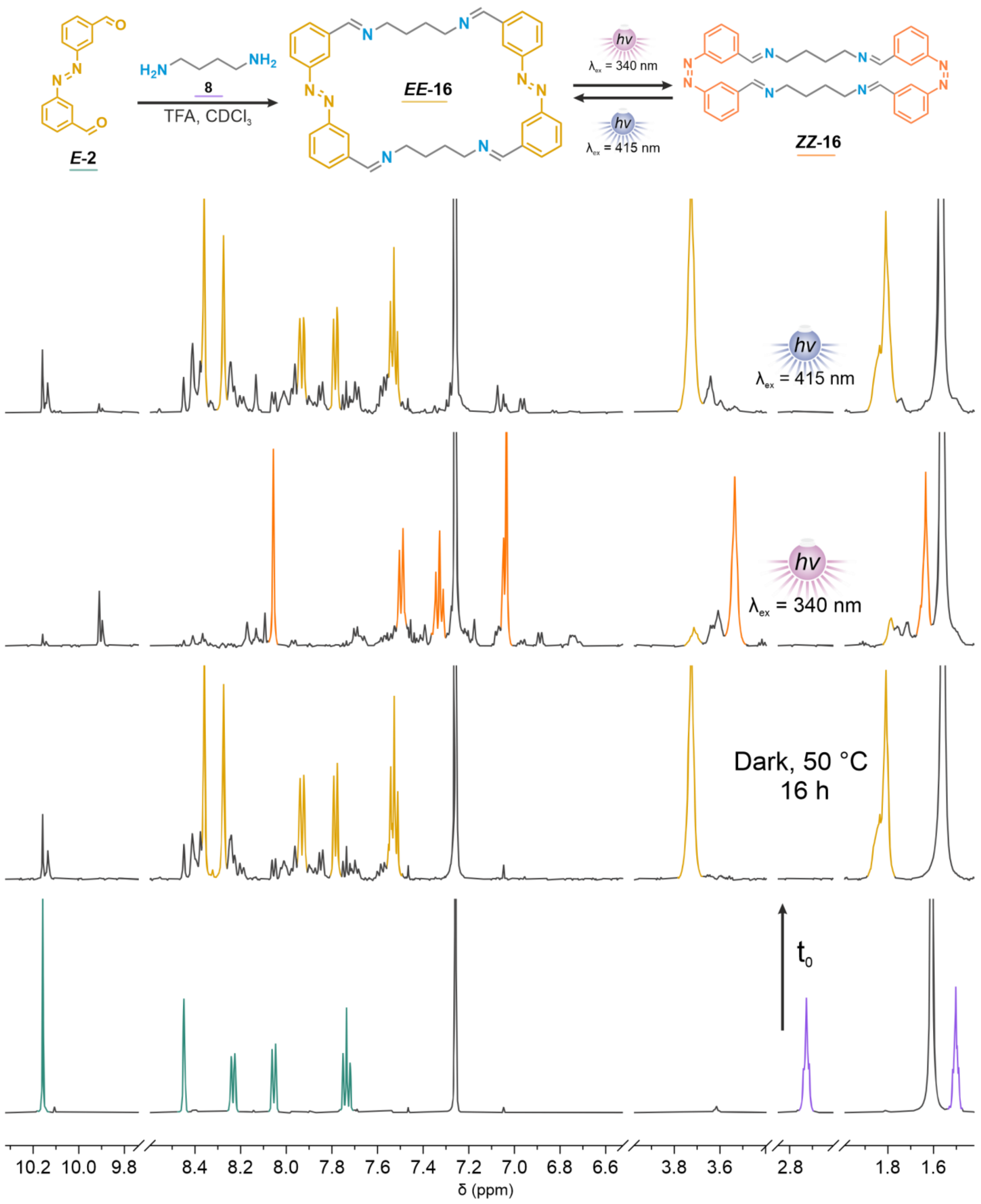


S25. [1]H NMR (500 MHz, $CDCl_3$, TFA 5 µM, 25 °C) stacked (from bottom to top) of the self-assembly of ***E*-2** (3 mM) + **8** (3mM) → ***EE*-16** immediately after mixing the components ($t_0$), followed by the self-assembled state in a after a period of 16 h in the dark at 50 °C. Next spectra show the photoisomerization ***EE*-16** → ***ZZ*-16** of the self-assembled system by UV irradiation ($\lambda_{irr}$ = 340 nm). On the top, the ***ZZ*-16**→ ***EE*-16** photoisomerization of the self-assembled system after visible irradiation ($\lambda_{irr}$ = 415 nm) is shown. Supporting video 9 illustrates this process.

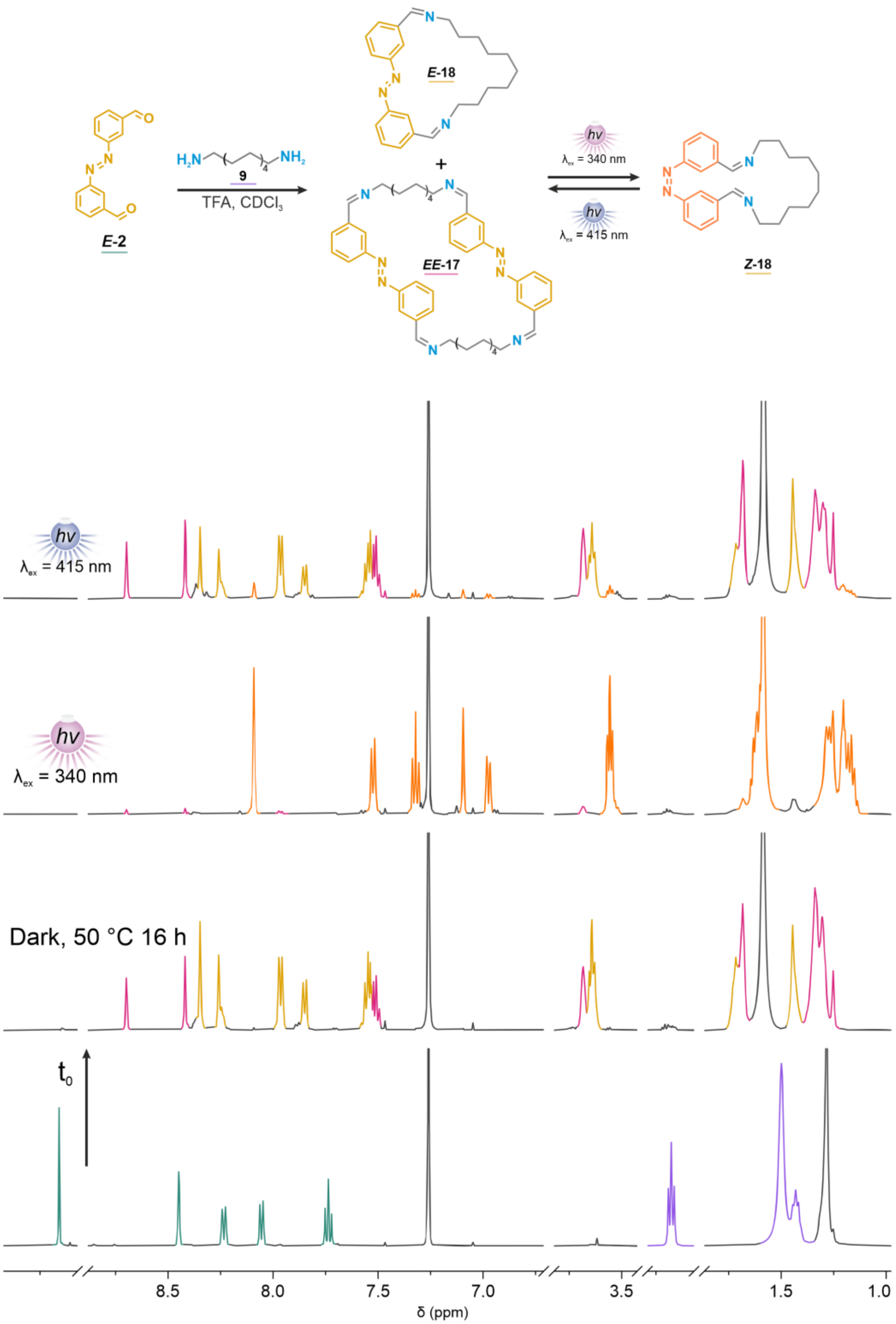


S26. $^1$H NMR (500 MHz, $CDCl_3$, TFA 5 µM, 25 °C) stacked (from bottom to top) of the self-assembly of ***E*-2** (3 mM) + **9** (3mM) → ***EE*-18** + ***E*-19** immediately after mixing the components ($t_0$), followed by the self-assembled state in a after a period of 16 h in the dark at 50 °C. Next spectra show the photoisomerization and macrocycle-to-macrocycle transformation ***EE*-18** + ***E*-19** → ***Z*-19** of the self-assembled system by UV irradiation ($\lambda_{irr}$ = 340 nm). On the top, the ***Z*-19**→ ***EE*-18** + ***E*-19** photoisomerization and macrocycle-to-macrocycle transformation of the self-assembled system after visible irradiation ($\lambda_{irr}$ = 415 nm) is shown. Supporting video 10 illustrates this process.

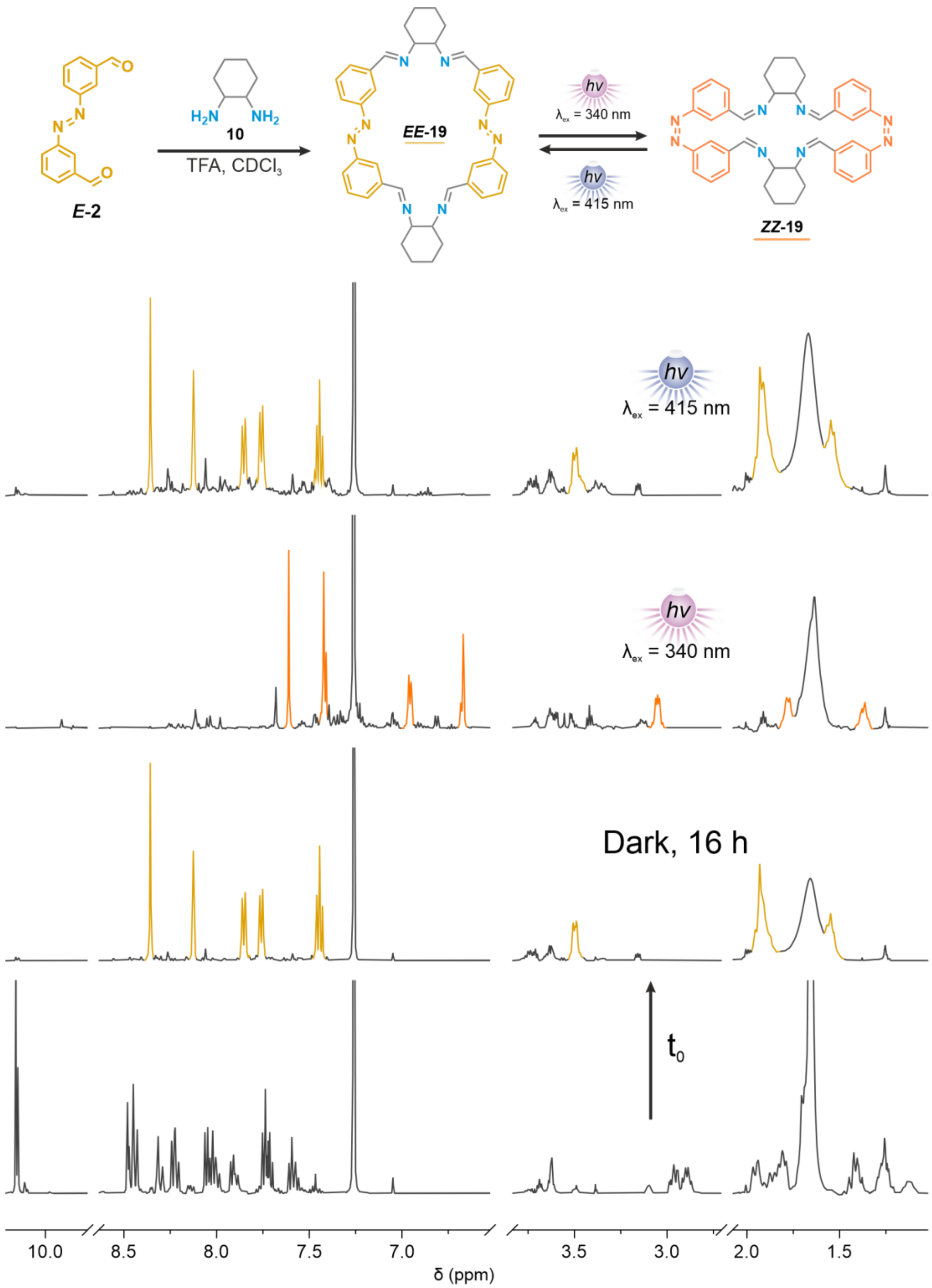


S27. [1]H NMR (500 MHz, $CDCl_3$, TFA 5 µM, 25 °C) stacked (from bottom to top) of the self-assembly of ***E*-2** (3 mM) + **10** (3mM) → ***EE*-19** immediately after mixing the components ($t_0$), followed by the self-assembled state in a after a period of 16 h in the dark. Next spectra show the photoisomerization ***EE*-19** → ***ZZ*-19** of the self-assembled system by UV irradiation ($\lambda_{irr}$ = 340 nm). On the top, the ***ZZ*-19**→ ***EE*-19** photoisomerization of the self-assembled system after visible irradiation ($\lambda_{irr}$ = 415 nm) is shown. Supporting video 11 illustrates this process.

## Cage-to-macrocycle transformations with ditopic amines 5-10.

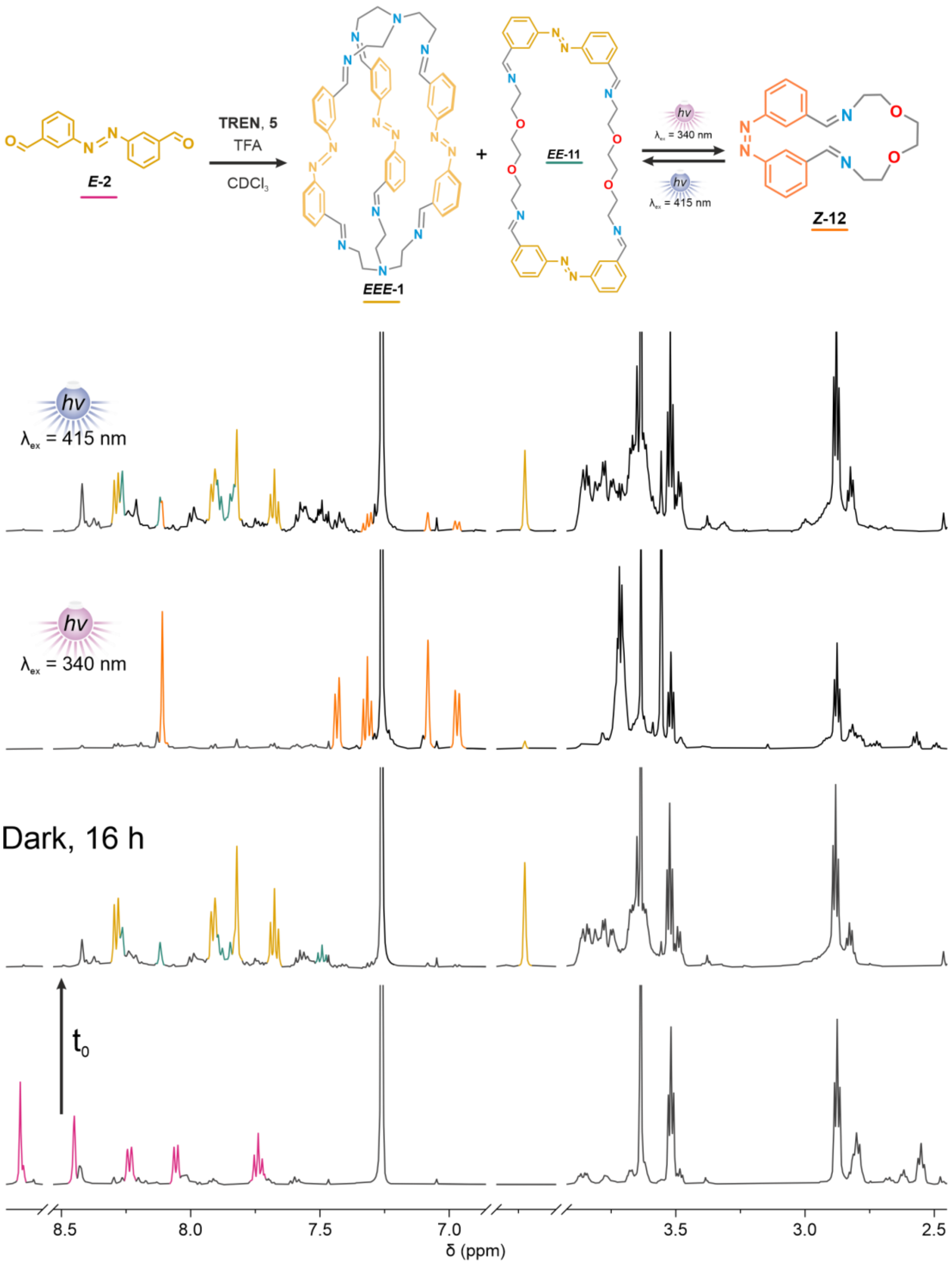


S28. [1]H NMR (500 MHz, $CDCl_3$, TFA 5 µM, 25 °C) stacked (from bottom to top) of the self-sorting and reversible light-fueled cage-to-macrocycle transformations for **5**. In the bottom, compounds ***E*-2** (3 mM) + **TREN** (2mM) + **5** (3mM) → ***EEE*-1** + ***EE*-11** immediately after mixing the components ($t_0$), followed by the self-sorting in the dark for 16 h. Next spectra show the cage-to-macrocycle transformation ***EEE*-1 + 5** → ***Z*-12** + **TREN** of the self-sorted system by UV irradiation ($\lambda_{irr}$ = 340 nm). On the top, the ***Z*-12** + **TREN** → ***EEE*-1 + 5** macrocycle to cage transformation of the self-assembled system after visible irradiation ($\lambda_{irr}$ = 415 nm) is shown.

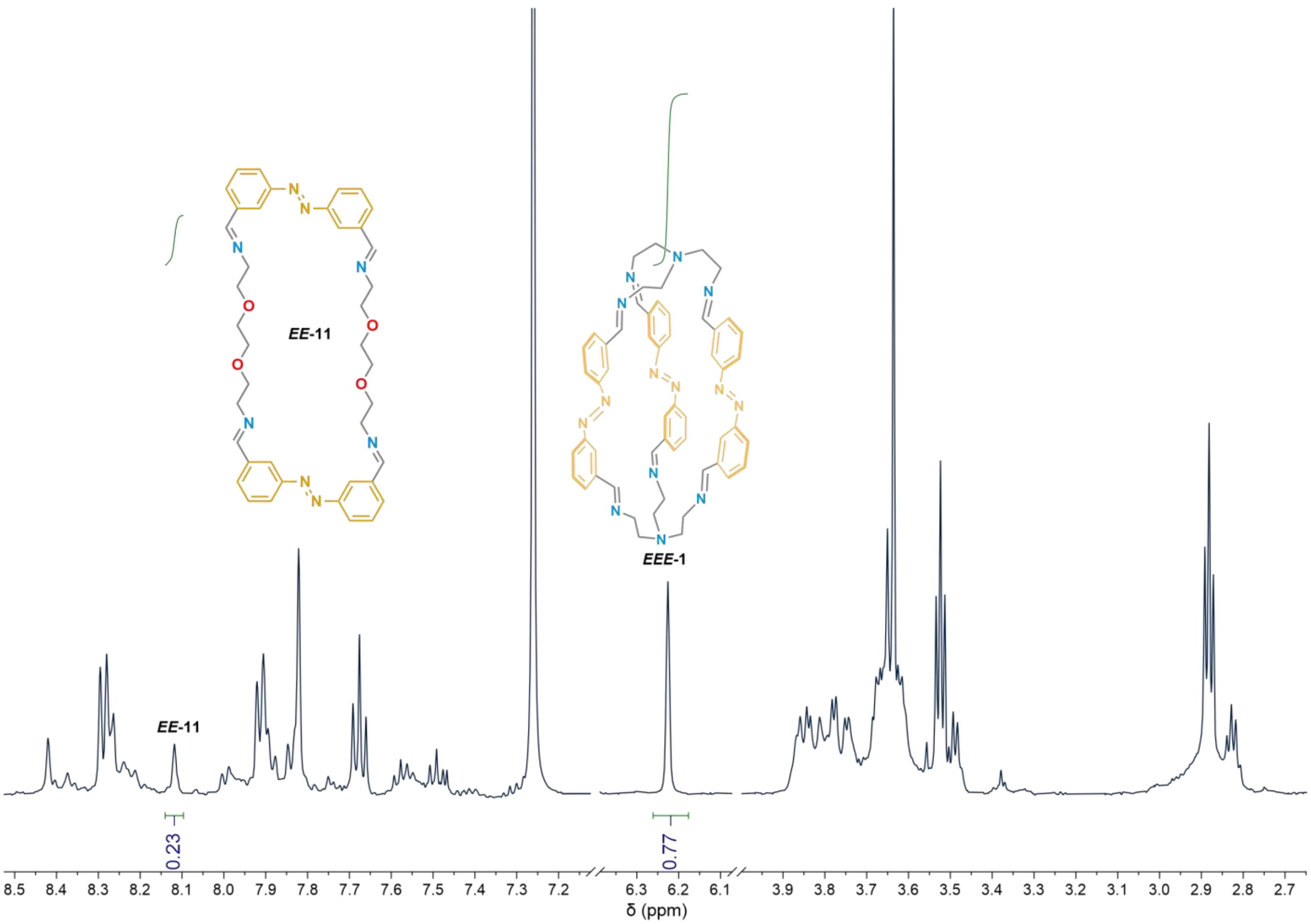


S29. $^{1}$H NMR (500 MHz, $CDCl_3$, TFA 5 µM, 25 °C) spectra of the self-sorted system for **5** after 16 h in the dark. The shown integration was used to determine the ratios after the self-sorting process in the dark.

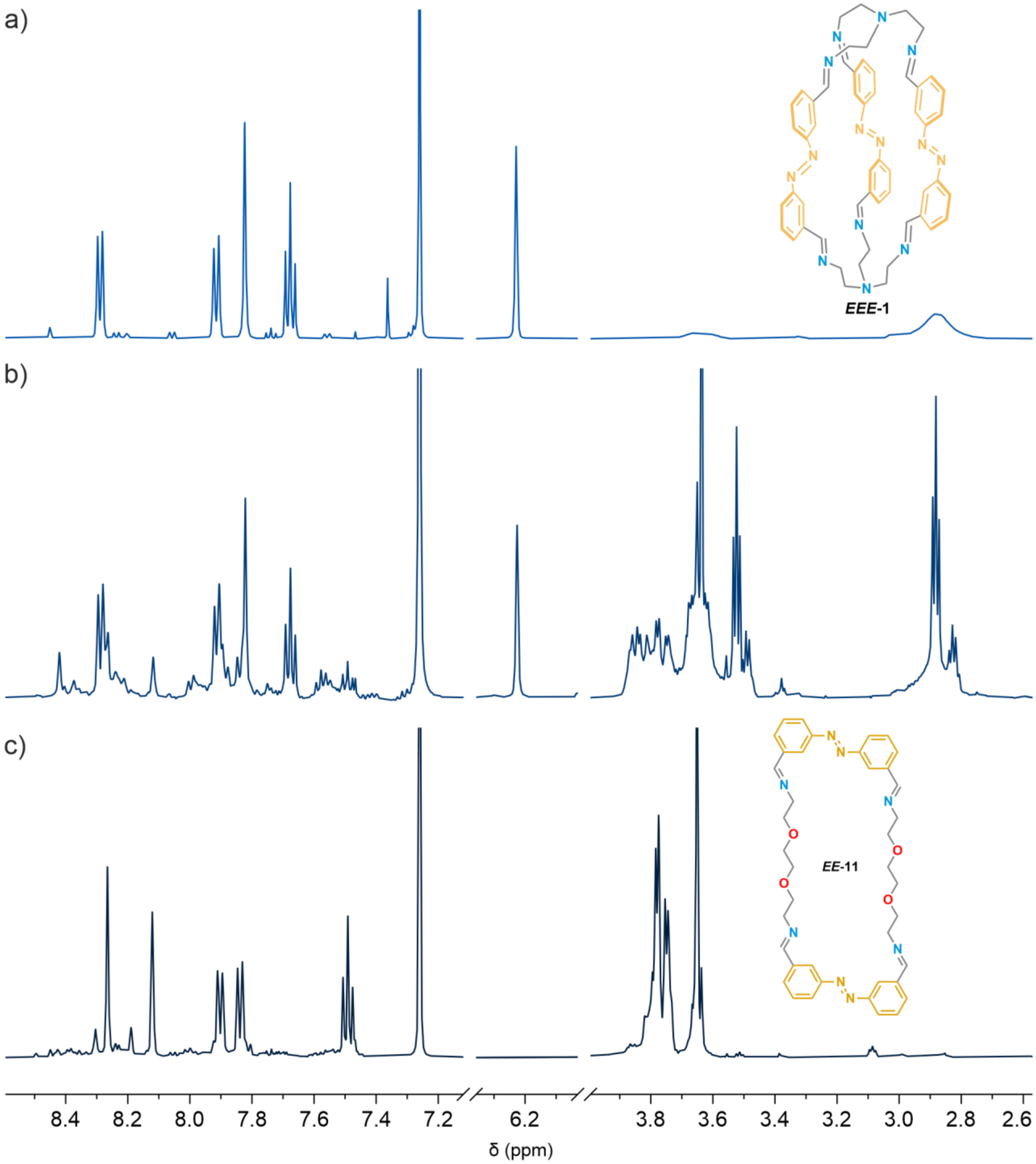


S30. [1]H NMR (500 MHz, $CDCl_3$, TFA 5 µM, 25 °C) stacked spectra comparing **a**, cage ***EEE*-1** equilibrated in the acidic solution, **b**, the self-sorted system of compounds ***E*-2** (3 mM) + **TREN** (2mM) + **5** (3mM) → ***EEE*-1** + ***EE*-11** after 16 h in the dark and **c**, self-assembled macrocycle ***EE*-11.**

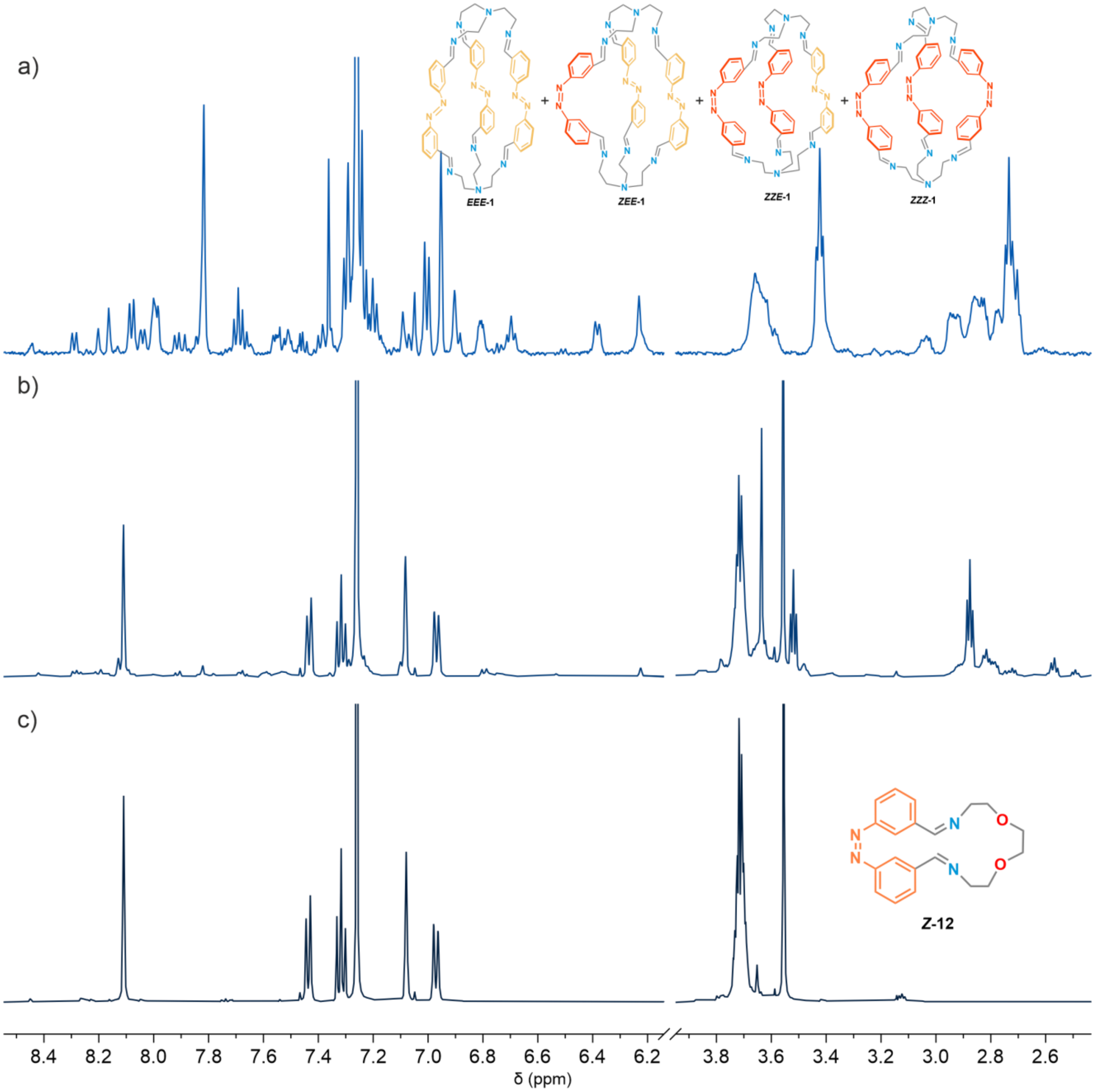


S31. $^{1}H$ NMR (500 MHz, $CDCl_3$, TFA 5 µM, 25 °C) stacked spectra comparing **a**, cage **1** irradiated with UV light ($\lambda_{irr}$ = 340 nm) generating a mixture of isomers of **1** (***EEE*-1**, ***ZEE*-1**, ***ZZE*-1** and ***ZZZ*-1**), **b**, the self-sorted system of compounds ***E*-2** (3 mM) + **TREN** (2mM) + **5** (3mM) after the light-fueled cage-to-macrocycle transformation ***EEE*-1** + ***EE*-11** → ***Z*-12** and **c**, irradiated sample of self-assembled system containing macrocycle ***Z*-12**.

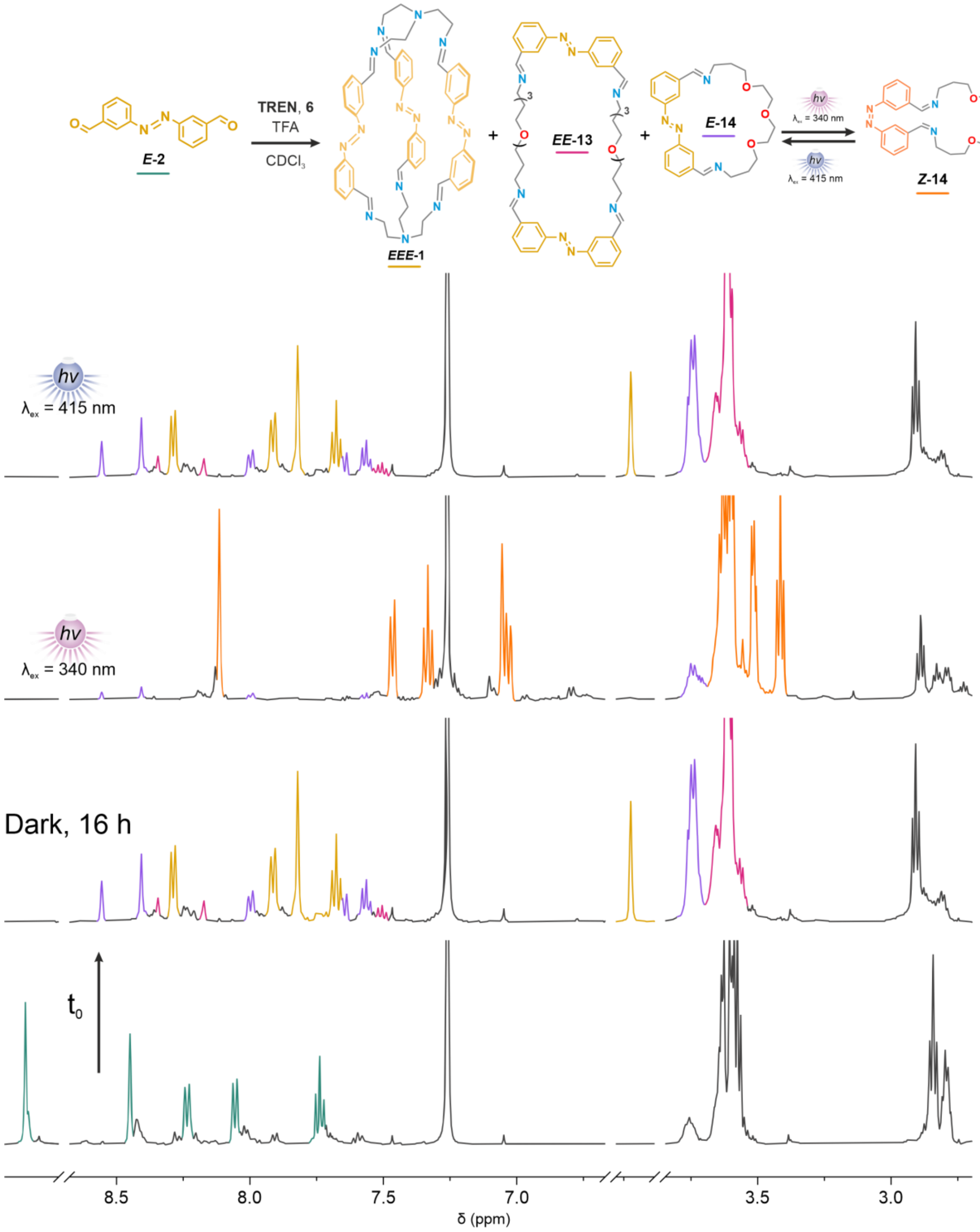


S32. [1]H NMR (500 MHz, $CDCl_3$, TFA 5 µM, 25 °C) stacked (from bottom to top) of the self-sorting and reversible light-fueled cage-to-macrocycle transformations for **6**. In the bottom, compounds ***E*-2** (3 mM) + **TREN** (2mM) + **6** (3mM) → ***EEE*-1** + ***EE*-13** + ***E*-14** immediately after mixing the components ($t_0$), followed by the self-sorting in the dark for 16 h. Next spectra show the cage-to-macrocycle transformation ***EEE*-1 + 6** → ***Z*-14** + **TREN** of the self-sorted system by UV irradiation ($\lambda_{irr}$ = 340 nm). On the top, the ***Z*-14** + **TREN** → ***EEE*-1 + 6** macrocycle to cage transformation of the self-assembled system after visible irradiation ($\lambda_{irr}$ = 415 nm) is shown.

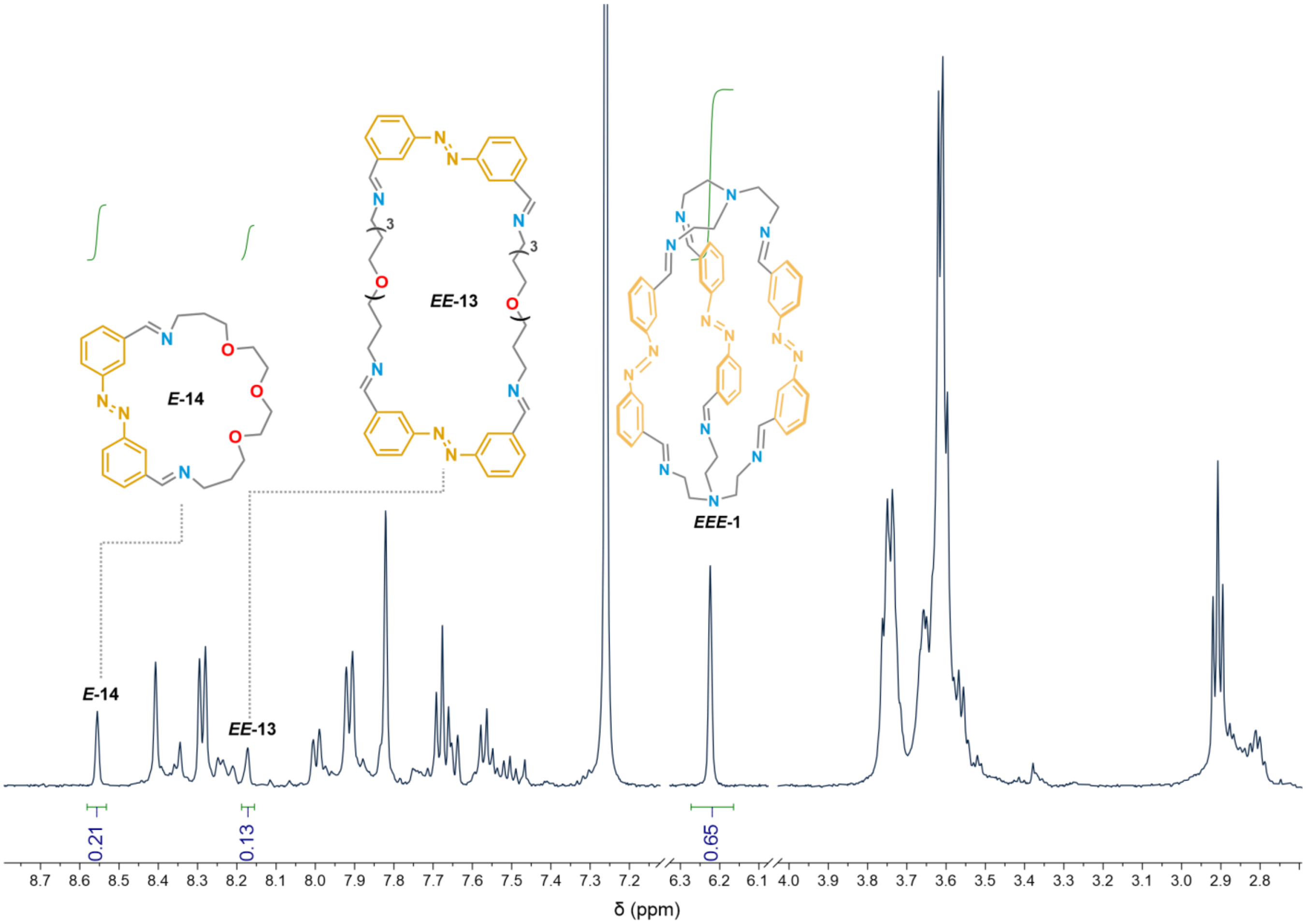


S33. $^{1}H$ NMR (500 MHz, $CDCl_3$, TFA 5 µM, 25 °C) spectra of the self-sorted system for **6** after 16 h in the dark. The shown integration was used to determine the ratios after the self-sorting process in the dark.

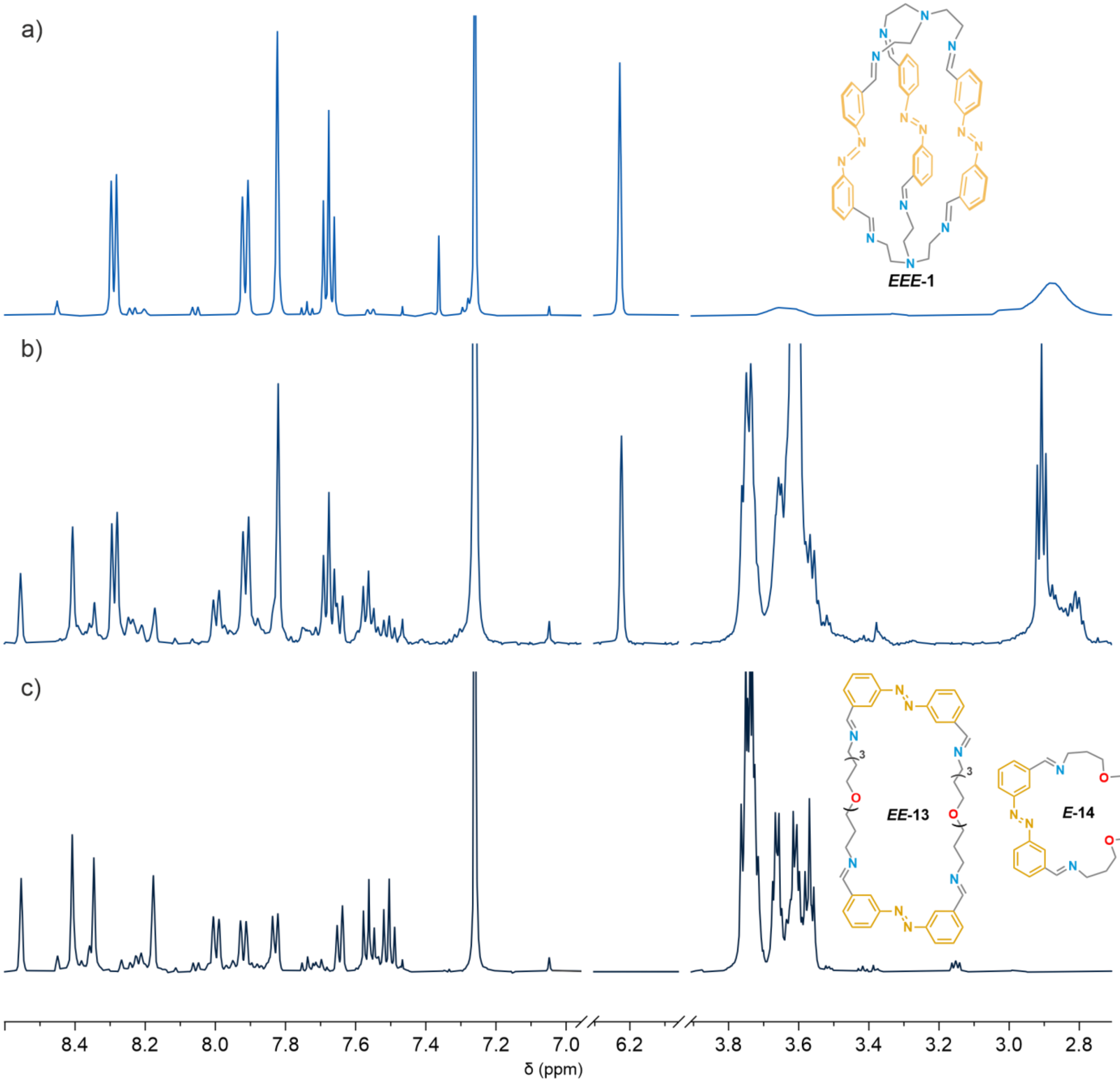


S34. $^1$H NMR (500 MHz, $CDCl_3$, TFA 5 µM, 25 °C) stacked spectra comparing **a**, cage ***EEE*-1** equilibrated in the acidic solution, **b**, the self-sorted system of compounds ***E*-2** (3 mM) + **TREN** (2mM) + **6** (3mM) → ***EEE*-1** + ***EE*-13** + ***E*-14** after 16 h in the dark and **c**, self-assembled macrocycles ***EE*-14** + ***E*-13**.

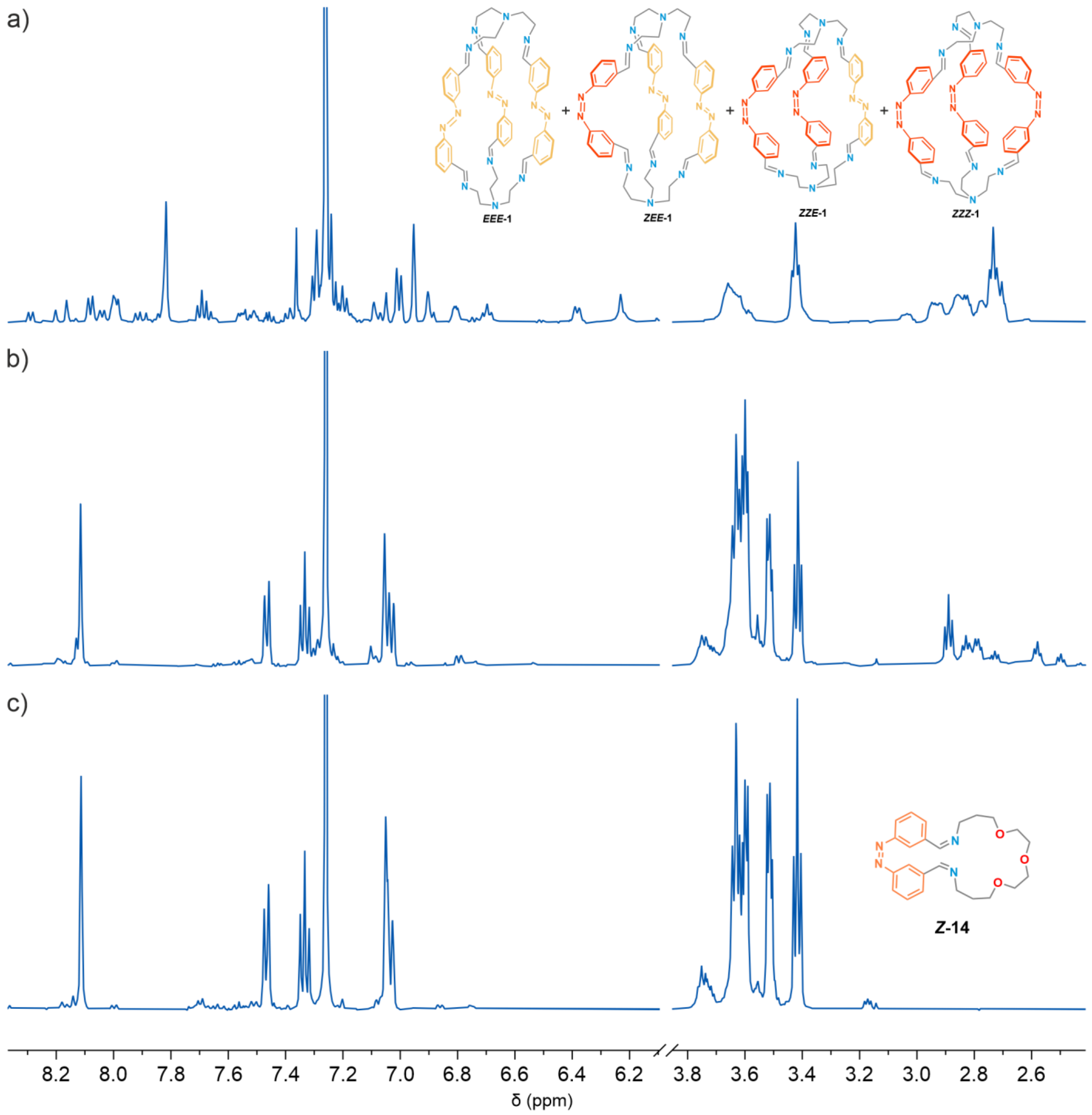


S35. [1]H NMR (500 MHz, $CDCl_3$, TFA 5 µM, 25 °C) stacked spectra comparing **a**, cage **1** irradiated with UV light ($\lambda_{irr}$ = 340 nm) generating a mixture of isomers of **1** (***EEE*-1**, ***ZEE*-1**, ***ZZE*-1** and ***ZZZ*-1**), **b**, the self-sorted system of compounds ***E*-2** (3 mM) + **TREN** (2mM) + **6** (3mM) after the light-fueled cage-to-macrocycle transformation ***EEE*-1** + ***EE*-11** → ***Z*-14** and **c**, irradiated sample of self-assembled system containing macrocycle ***Z*-14**.

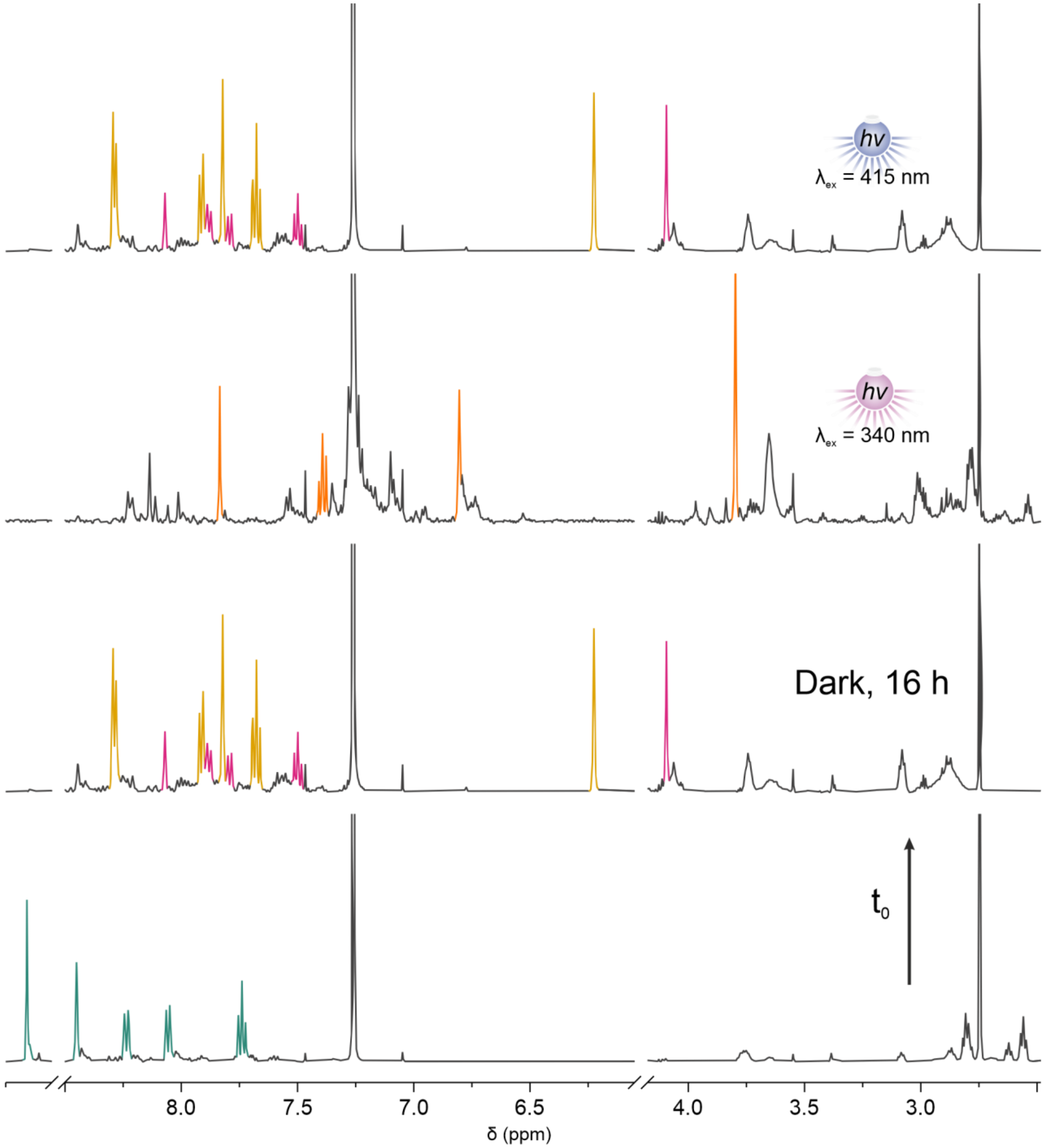


S36. [1]H NMR (500 MHz, $CDCl_3$, TFA 5 µM, 25 °C) stacked (from bottom to top) of the self-sorting and reversible light-fueled cage-to-macrocycle transformations for **7**. In the bottom, compounds ***E*-2** (3 mM) + **TREN** (2mM) + **7** (3mM) → ***EEE*-1** + ***EE*-15** immediately after mixing the components ($t_0$), followed by the self-sorting in the dark for 16 h. Next spectra show the cage-to-macrocycle transformation ***EEE*-1 + 6** → ***ZZ*-15** + **TREN** of the self-sorted system by UV irradiation ($\lambda_{irr}$ = 340 nm). On the top, the ***ZZ*-15** + **TREN** → ***EEE*-1 + 7** macrocycle to cage transformation of the self-assembled system after visible irradiation ($\lambda_{irr}$ = 415 nm) is shown.

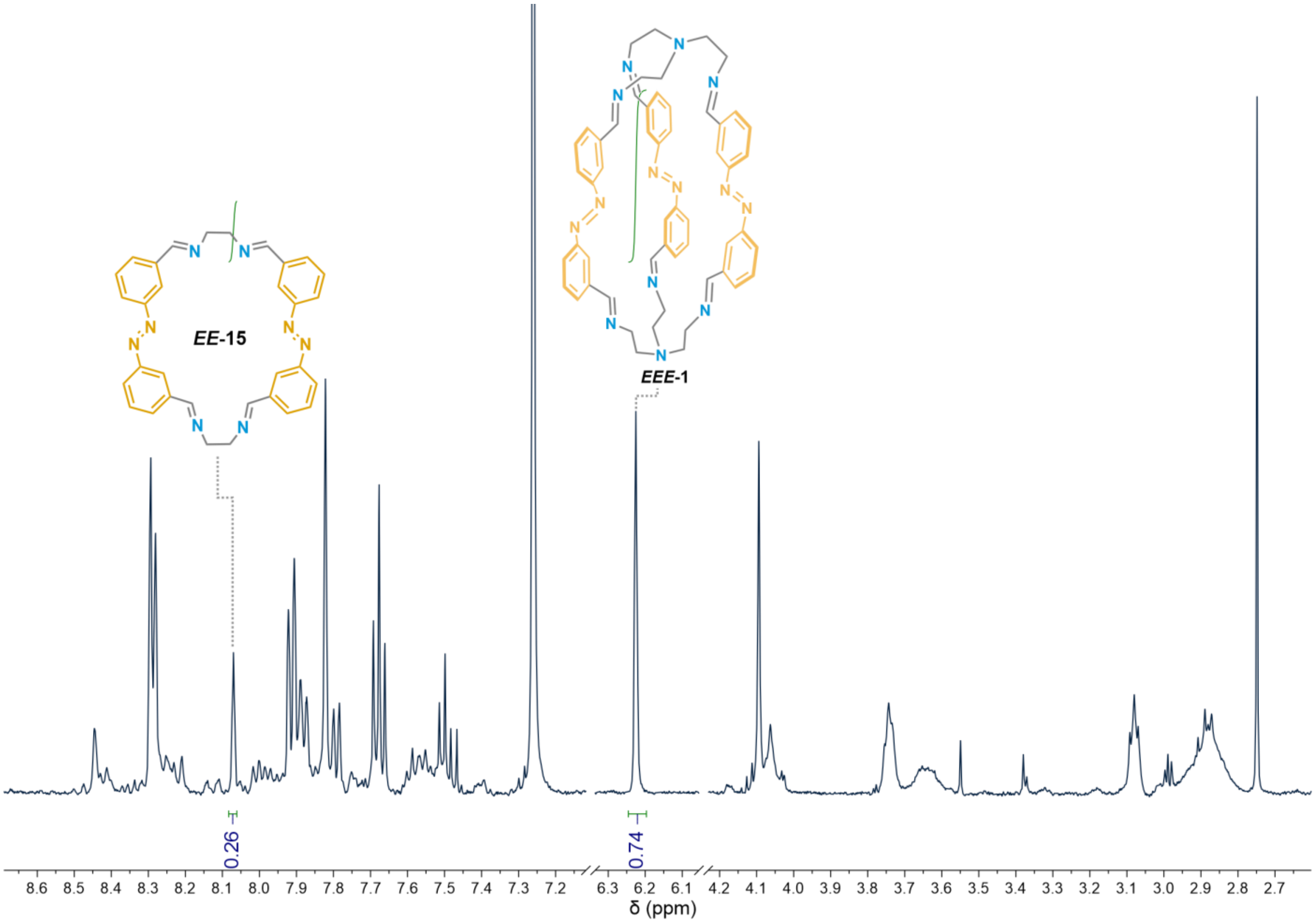


S37. [1]H NMR (500 MHz, $CDCl_3$, TFA 5 µM, 25 °C) spectra of the self-sorted system for **7** after 16 h in the dark. The shown integration was used to determine the ratios after the self-sorting process in the dark.

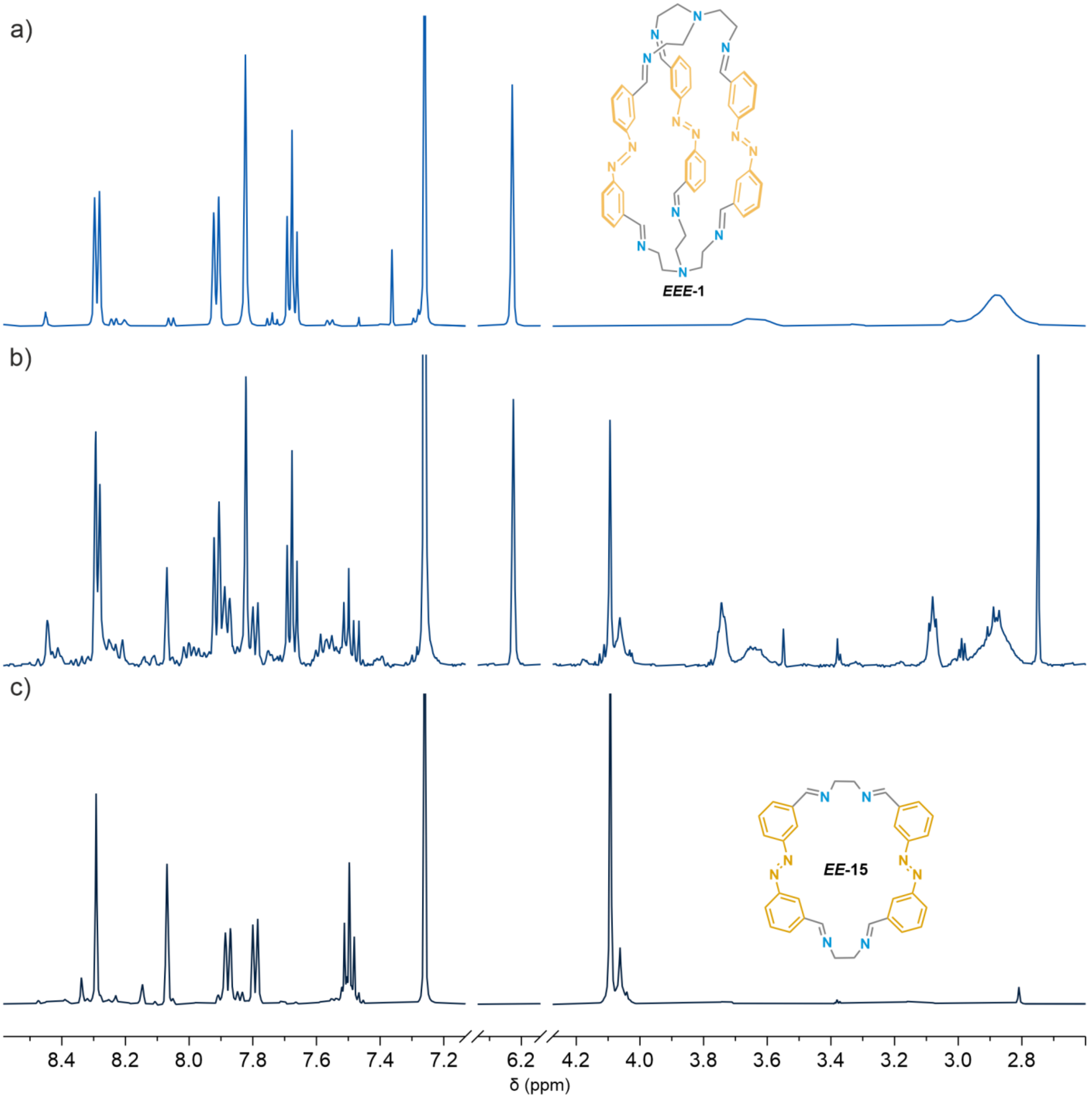


S38. $^{1}H$ NMR (500 MHz, $CDCl_3$, TFA 5 µM, 25 °C) stacked spectra comparing **a**, cage ***EEE*-1** equilibrated in the acidic solution, **b**, the self-sorted system of compounds ***E*-2** (3 mM) + **TREN** (2mM) + **7** (3mM) → ***EEE*-1** + ***EE*-15** + after 16 h in the dark and **c**, self-assembled macrocycle ***EE*-15**.

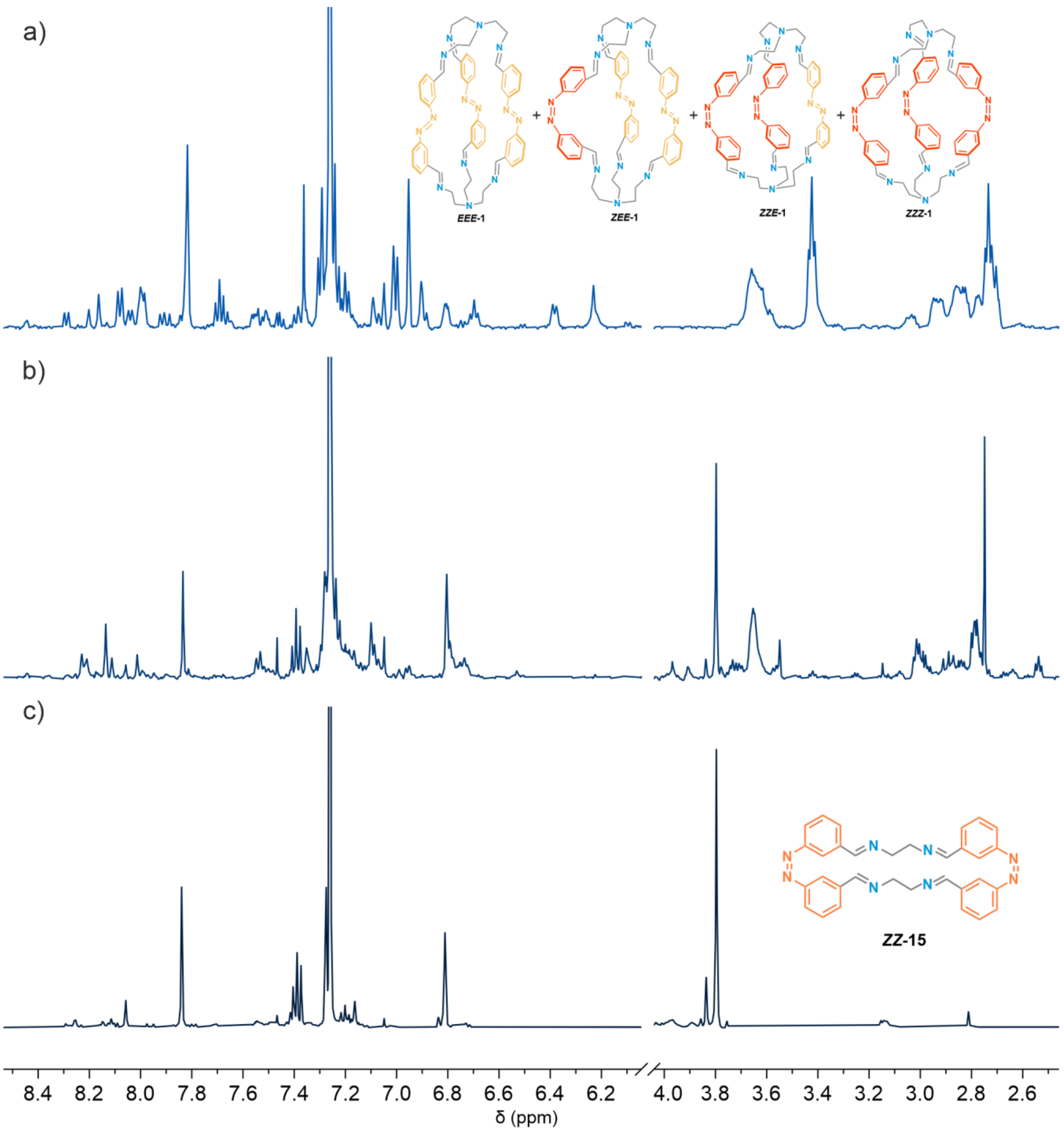


S39. [1]H NMR (500 MHz, $CDCl_3$, TFA 5 µM, 25 °C) stacked spectra comparing **a**, cage **1** irradiated with UV light ($\lambda_{irr}$ = 340 nm) generating a mixture of isomers of **1** (***EEE*-1**, ***ZEE*-1**, ***ZZE*-1** and ***ZZZ*-1**), **b**, the self-sorted system of compounds ***E*-2** (3 mM) + **TREN** (2mM) + **7** (3mM) after the light-fueled cage-to-macrocycle transformation ***EEE*-1** + ***EE*-15** → ***ZZ*-15** and **c**, irradiated sample of self-assembled system containing macrocycle ***ZZ*-15**.

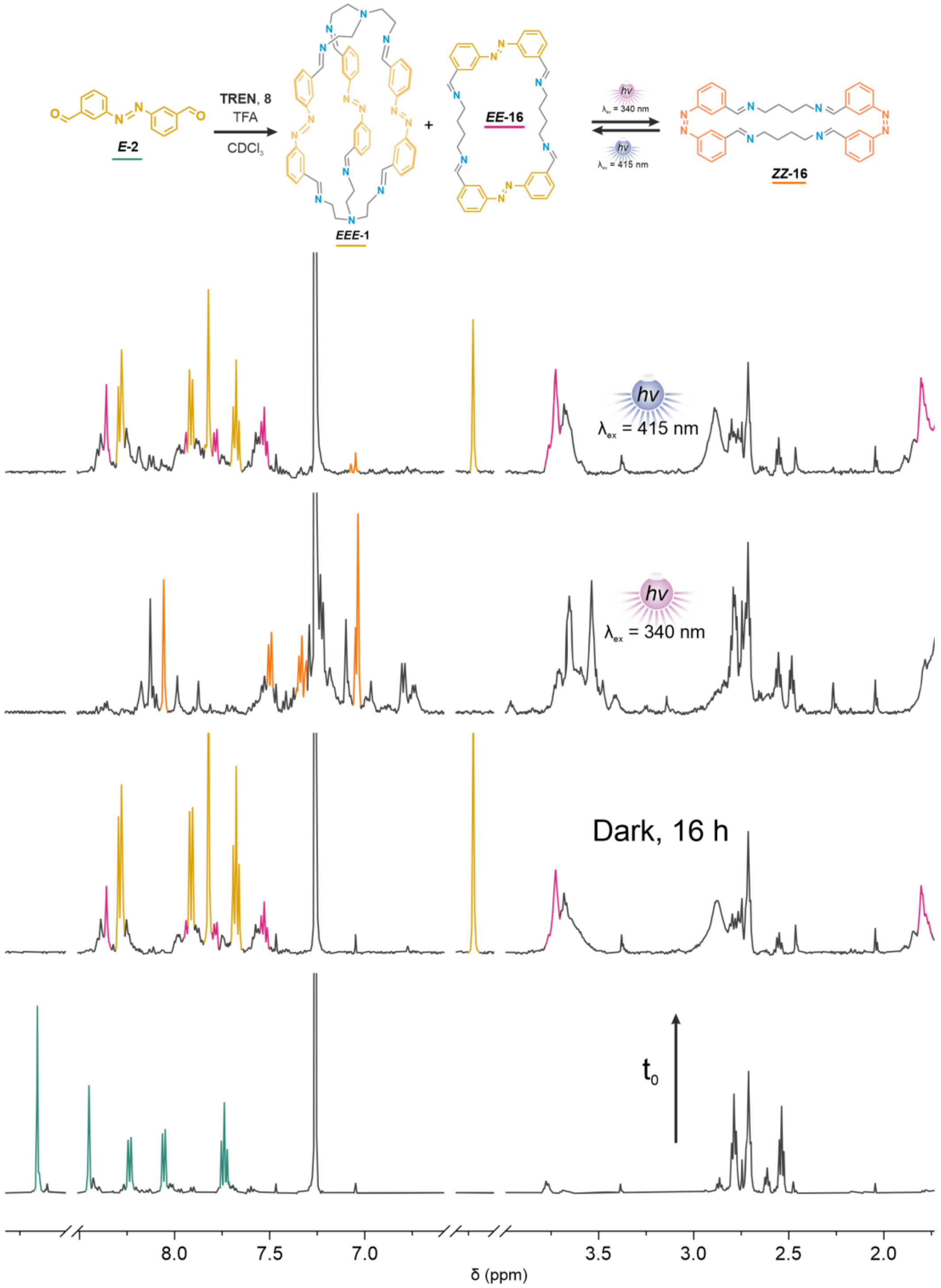


S40. [1]H NMR (500 MHz, $CDCl_3$, TFA 5 µM, 25 °C) stacked (from bottom to top) of the self-sorting and reversible light-fueled cage-to-macrocycle transformations for **8**. In the bottom, compounds ***E*-2** (3 mM) + **TREN** (2mM) + **8** (3mM) → ***EEE*-1** + ***EE*-16** immediately after mixing the components ($t_0$), followed by the self-sorting in the dark for 16 h. Next spectra show the cage-to-macrocycle transformation ***EEE*-1 + 8** → ***ZZ*-16** + **TREN** of the self-sorted system by UV irradiation ($\lambda_{irr}$ = 340 nm). On the top, the ***ZZ*-16** + **TREN** → ***EEE*-1 + 8** macrocycle to cage transformation of the self-assembled system after visible irradiation ($\lambda_{irr}$ = 415 nm) is shown.

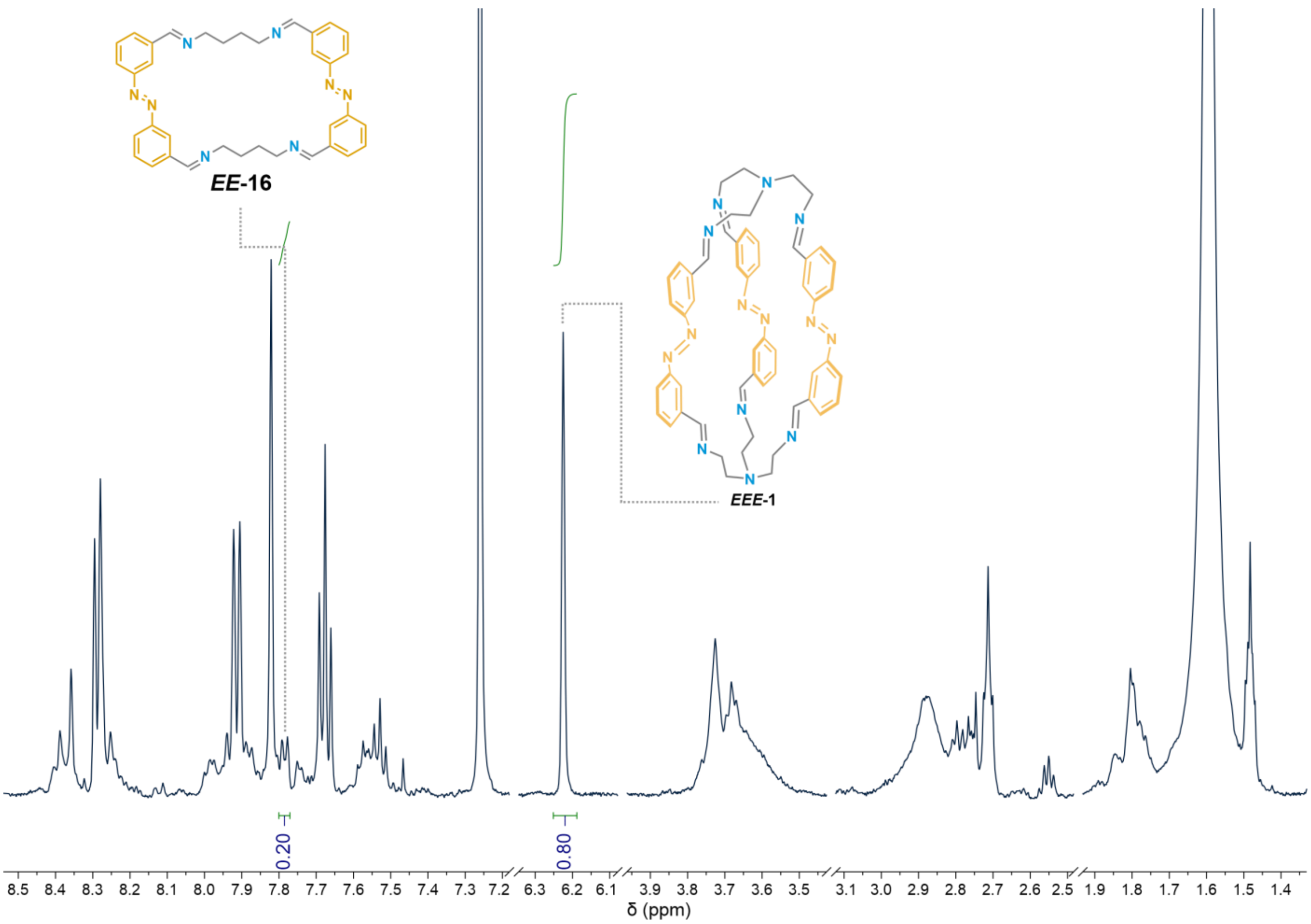


S41. $^1$H NMR (500 MHz, $CDCl_3$, TFA 5 µM, 25 °C) spectra of the self-sorted system for **8** after 16 h in the dark. The shown integration was used to determine the ratios after the self-sorting process in the dark.

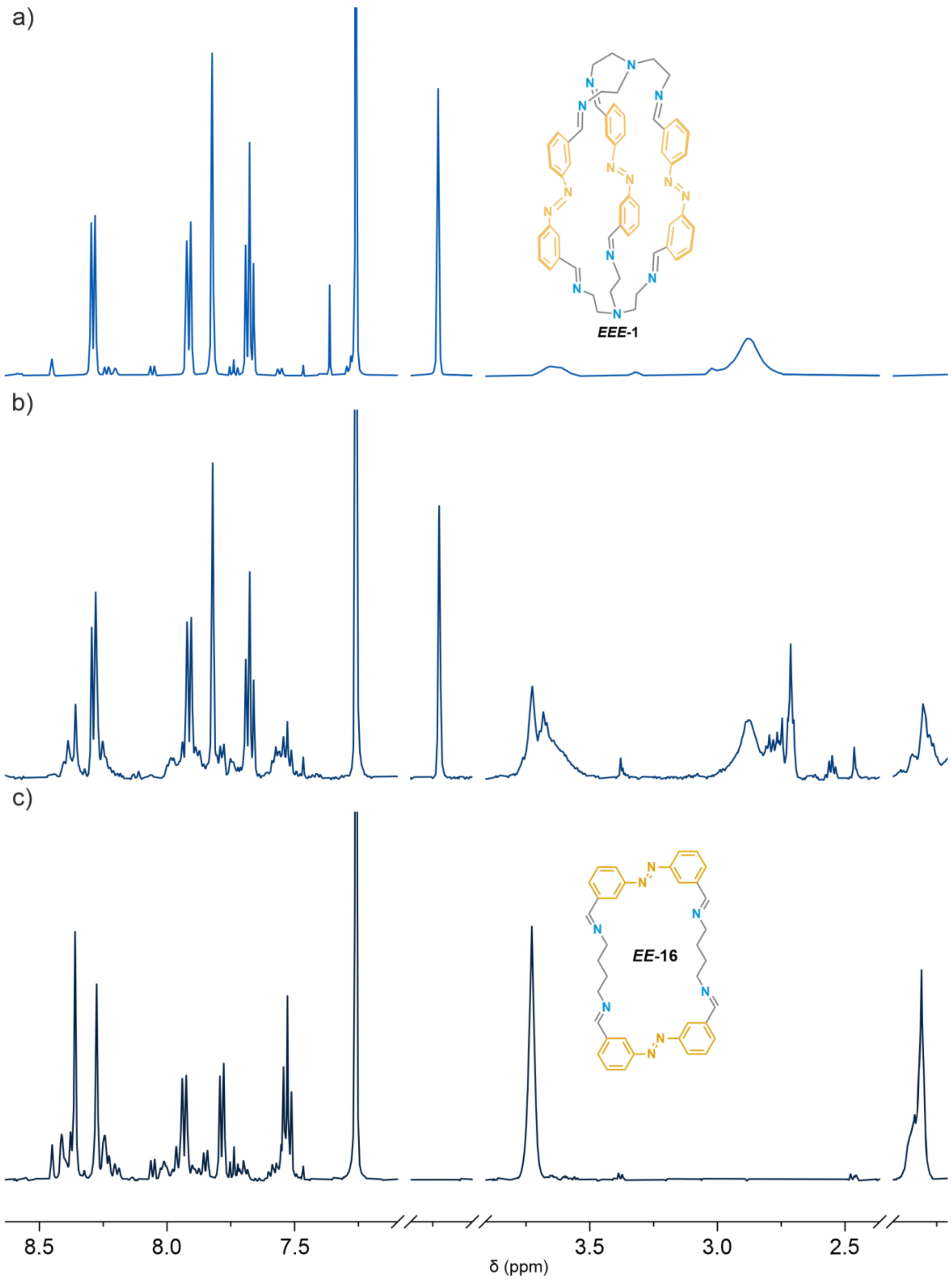


S42. $^{1}$H NMR (500 MHz, $CDCl_3$, TFA 5 µM, 25 °C) stacked spectra comparing **a**, cage ***EEE*-1** equilibrated in the acidic solution, **b**, the self-sorted system of compounds ***E*-2** (3 mM) + **TREN** (2mM) + **8** (3mM) → ***EEE*-1** + ***EE*-16** + after 16 h in the dark and **c**, self-assembled macrocycle ***EE*-16**.

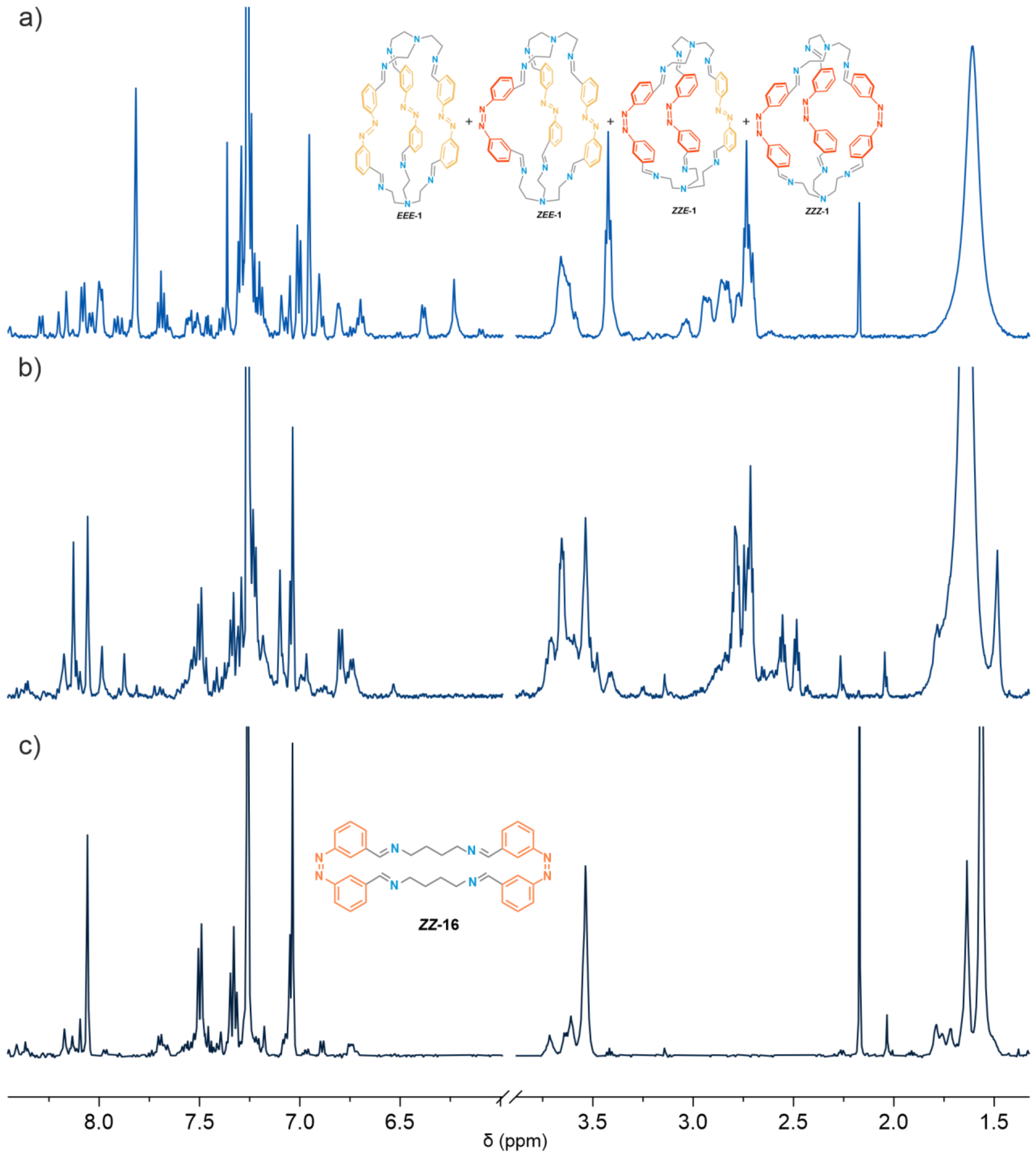


S43. $^1$H NMR (500 MHz, $CDCl_3$, TFA 5 µM, 25 °C) stacked spectra comparing **a**, cage **1** irradiated with UV light ($\lambda_{irr}$ = 340 nm) generating a mixture of isomers of **1** (***EEE*-1**, ***ZEE*-1**, ***ZZE*-1** and ***ZZZ*-1**), **b**, the self-sorted system of compounds ***E*-2** (3 mM) + **TREN** (2mM) + **8** (3mM) after the light-fueled cage-to-macrocycle transformation ***EEE*-1** + ***EE*-16** → ***ZZ*-16** and **c**, irradiated sample of self-assembled system containing macrocycle ***ZZ*-16**.

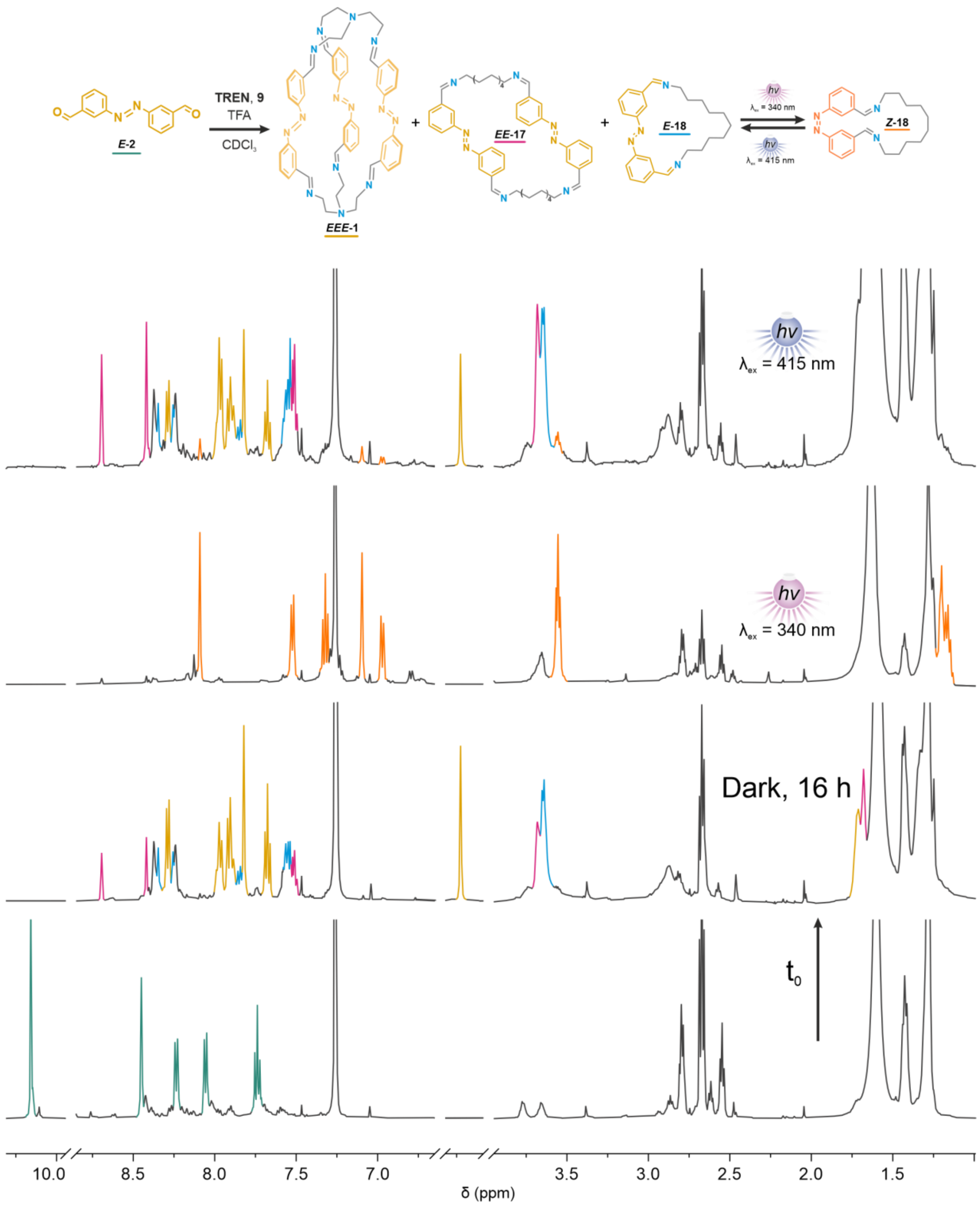


S44. [1]H NMR (500 MHz, $CDCl_3$, TFA 5 µM, 25 °C) stacked (from bottom to top) of the self-sorting and reversible light-fueled cage-to-macrocycle transformations for **9**. In the bottom, compounds ***E*-2** (3 mM) + **TREN** (2mM) + **9** (3mM) → ***EEE*-1** + ***EE*-17** + ***E*-18** immediately after mixing the components ($t_0$), followed by the self-sorting in the dark for 16 h. Next spectra show the cage-to-macrocycle transformation ***EEE*-1 + 8** → ***Z*-18** + **TREN** of the self-sorted system by UV irradiation ($\lambda_{irr}$ = 340 nm). On the top, the ***Z*-18** + **TREN** → ***EEE*-1 + 9** macrocycle to cage transformation of the self-assembled system after visible irradiation ($\lambda_{irr}$ = 415 nm) is shown.

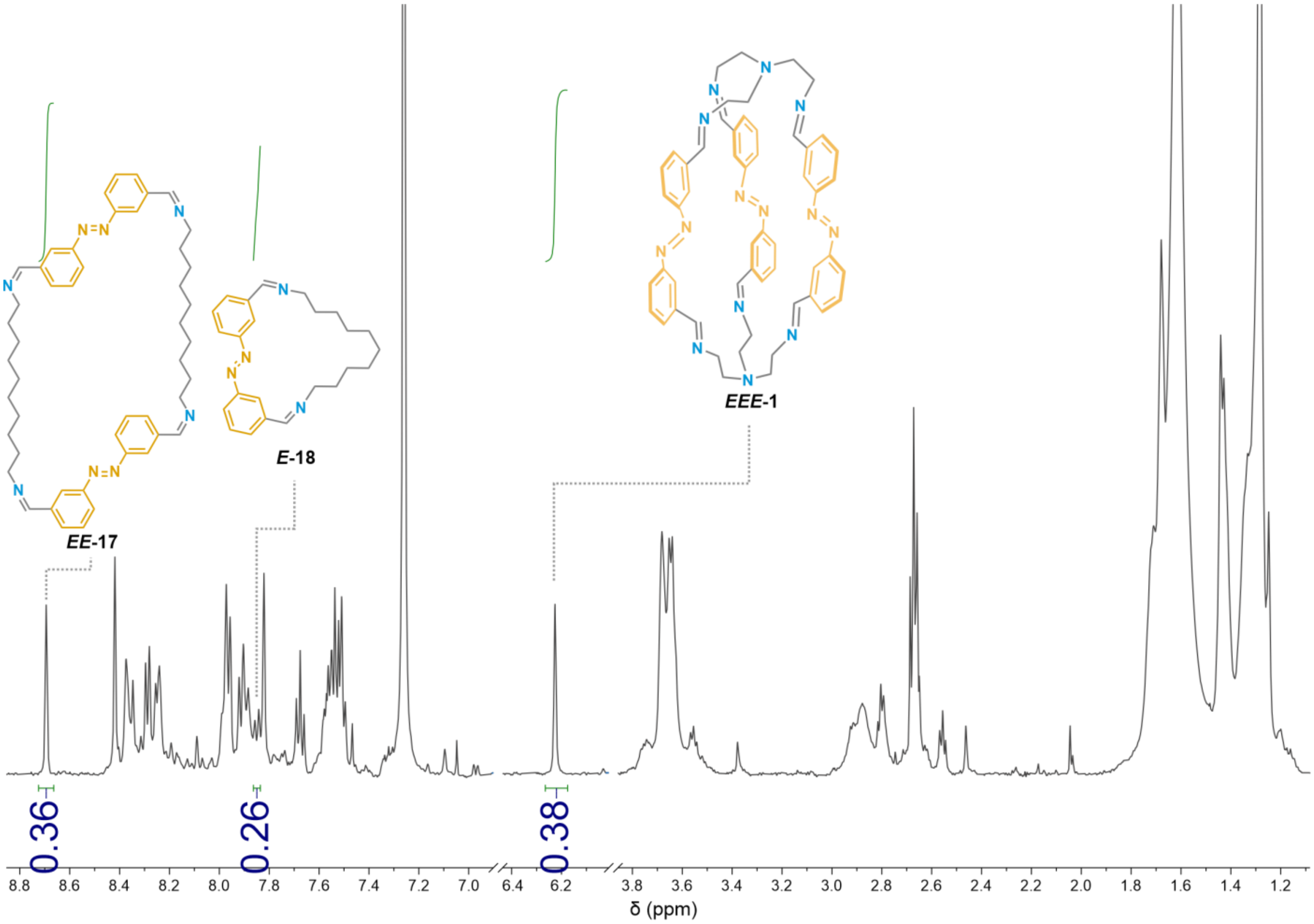


S45. [1]H NMR (500 MHz, $CDCl_3$, TFA 5 µM, 25 °C) spectra of the self-sorted system for **9** after 16 h in the dark. The shown integration was used to determine the ratios after the self-sorting process in the dark.

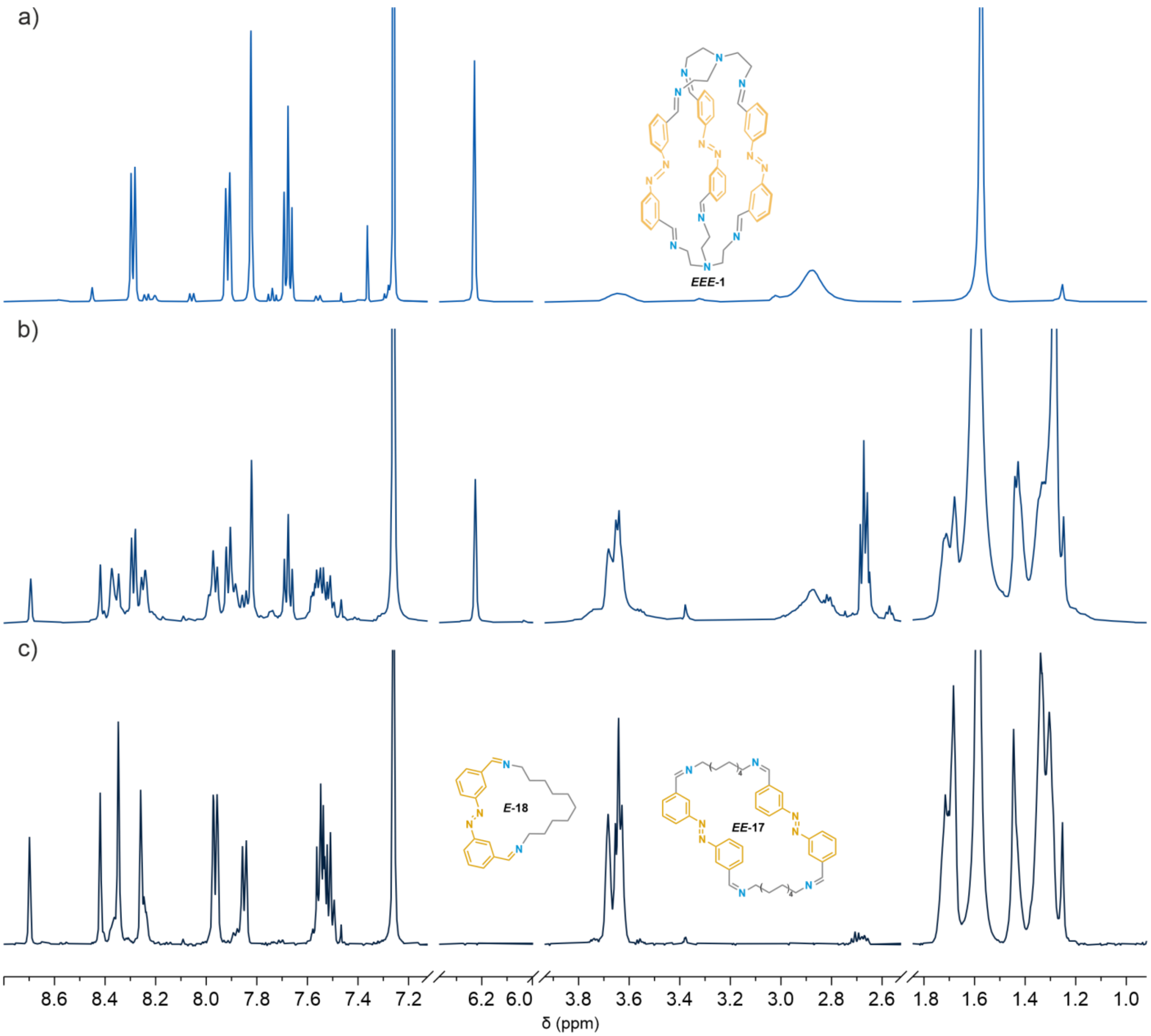


S46. [1]H NMR (500 MHz, $CDCl_3$, TFA 5 µM, 25 °C) stacked spectra comparing **a**, cage ***EEE*-1** equilibrated in the acidic solution, **b**, the self-sorted system of compounds ***E*-2** (3 mM) + **TREN** (2mM) + **9** (3mM) → ***EEE*-1** + ***EE*-17** + ***E*-18** after 16 h in the dark and **c**, self-assembled macrocycle ***EE*-17** + ***E*-18**.

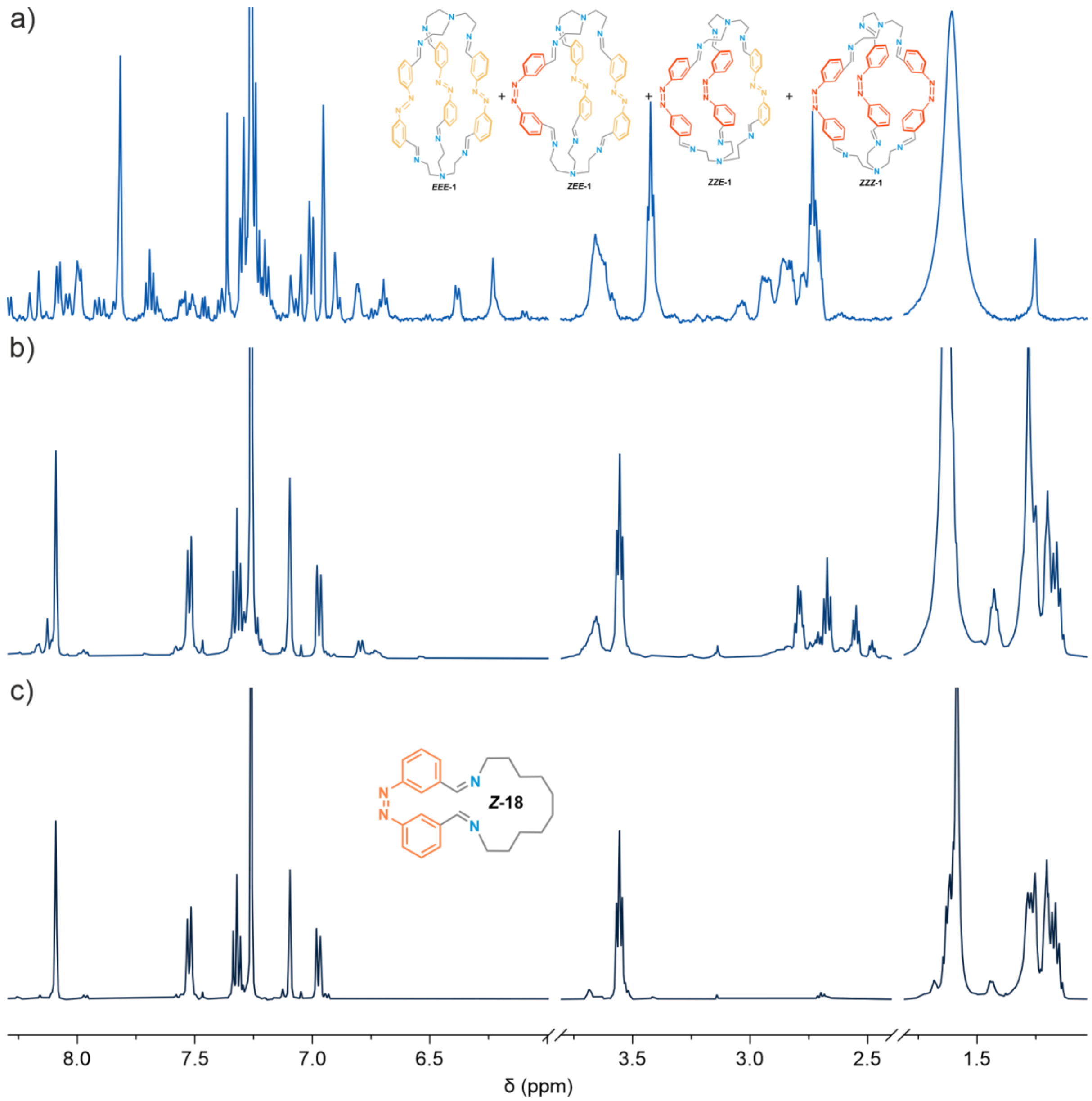


S47. $^{1}$H NMR (500 MHz, $CDCl_3$, TFA 5 µM, 25 °C) stacked spectra comparing **a**, cage **1** irradiated with UV light ($\lambda_{irr}$ = 340 nm) generating a mixture of isomers of **1** (***EEE*-1**, ***ZEE*-1**, ***ZZE*-1** and ***ZZZ*-1**), **b**, the self-sorted system of compounds ***E*-2** (3 mM) + **TREN** (2mM) + **9** (3mM) after the light-fueled cage-to-macrocycle transformation ***EEE*-1** + ***EE*-17** + ***E*-18** → ***Z*-18** and **c**, irradiated sample of self-assembled system containing macrocycle ***Z*-18**.

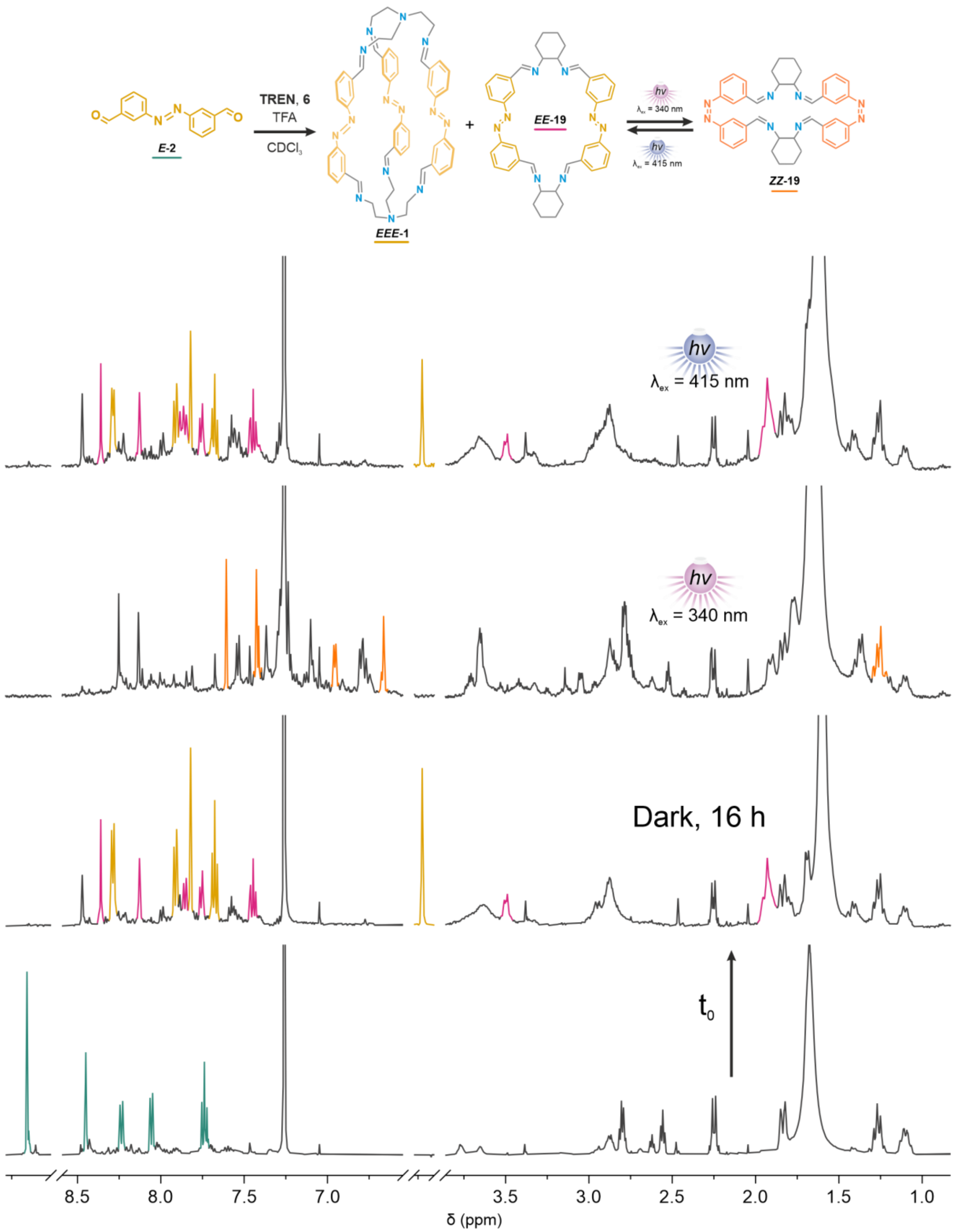


S48. [1]H NMR (500 MHz, $CDCl_3$, TFA 5 µM, 25 °C) stacked (from bottom to top) of the self-sorting and reversible light-fueled cage-to-macrocycle transformations for **10**. In the bottom, compounds ***E*-2** (3 mM) + **TREN** (2mM) + **10** (3mM) → ***EEE*-1** + ***EE*-19** immediately after mixing the components ($t_0$), followed by the self-sorting in the dark for 16 h. Next spectra show the cage-to-macrocycle transformation ***EEE*-1 + 10** → ***ZZ*-19** + **TREN** of the self-sorted system by UV irradiation ($\lambda_{irr}$ = 340 nm). On the top, the ***ZZ*-19** + **TREN** →

***EEE*-1 + 10** macrocycle to cage transformation of the self-assembled system after visible irradiation ($\lambda_{irr}$ = 415 nm) is shown.

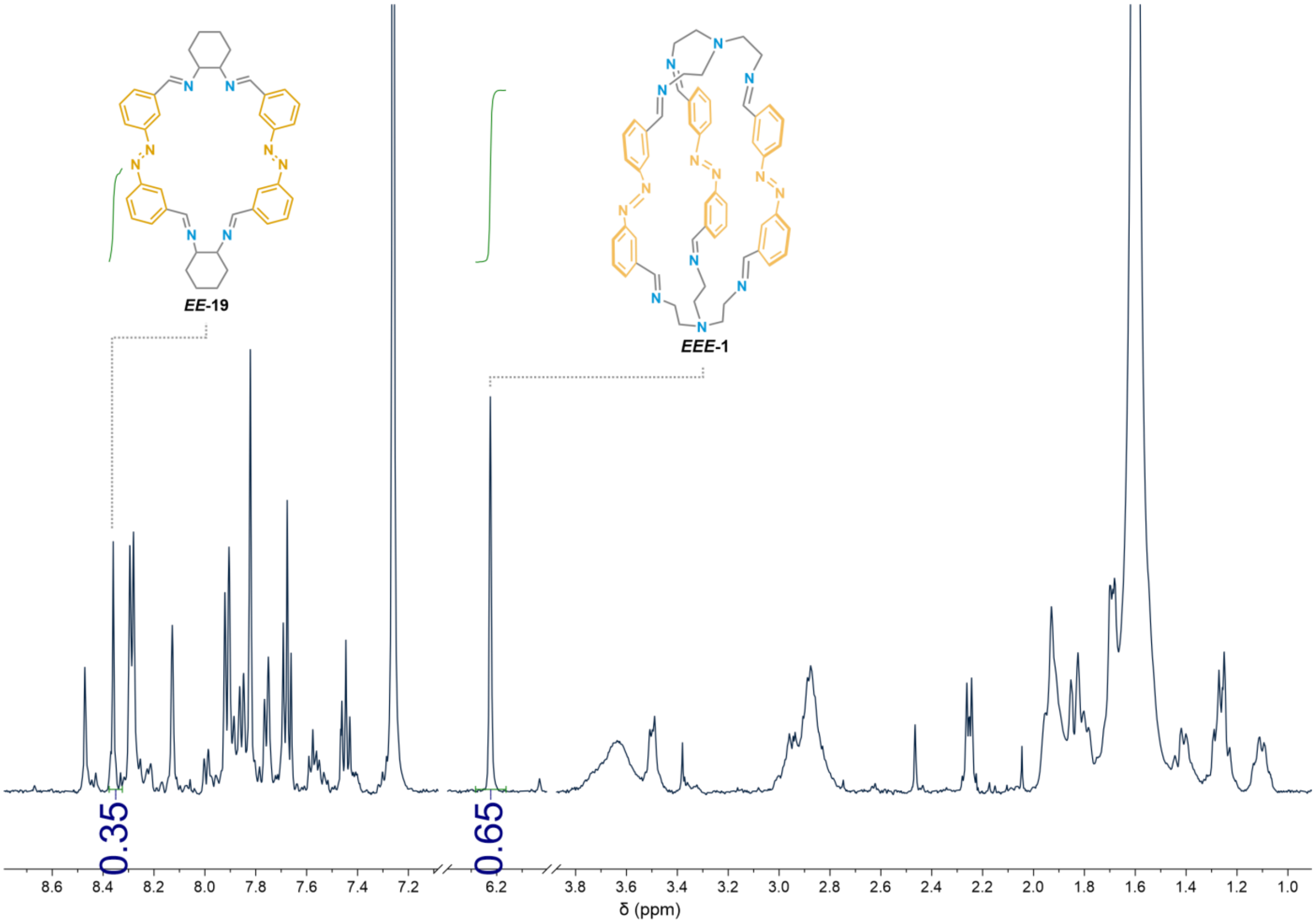


S49. $^1$H NMR (500 MHz, $CDCl_3$, TFA 5 µM, 25 °C) spectra of the self-sorted system for **10** after 16 h in the dark. The shown integration was used to determine the ratios after the self-sorting process in the dark.

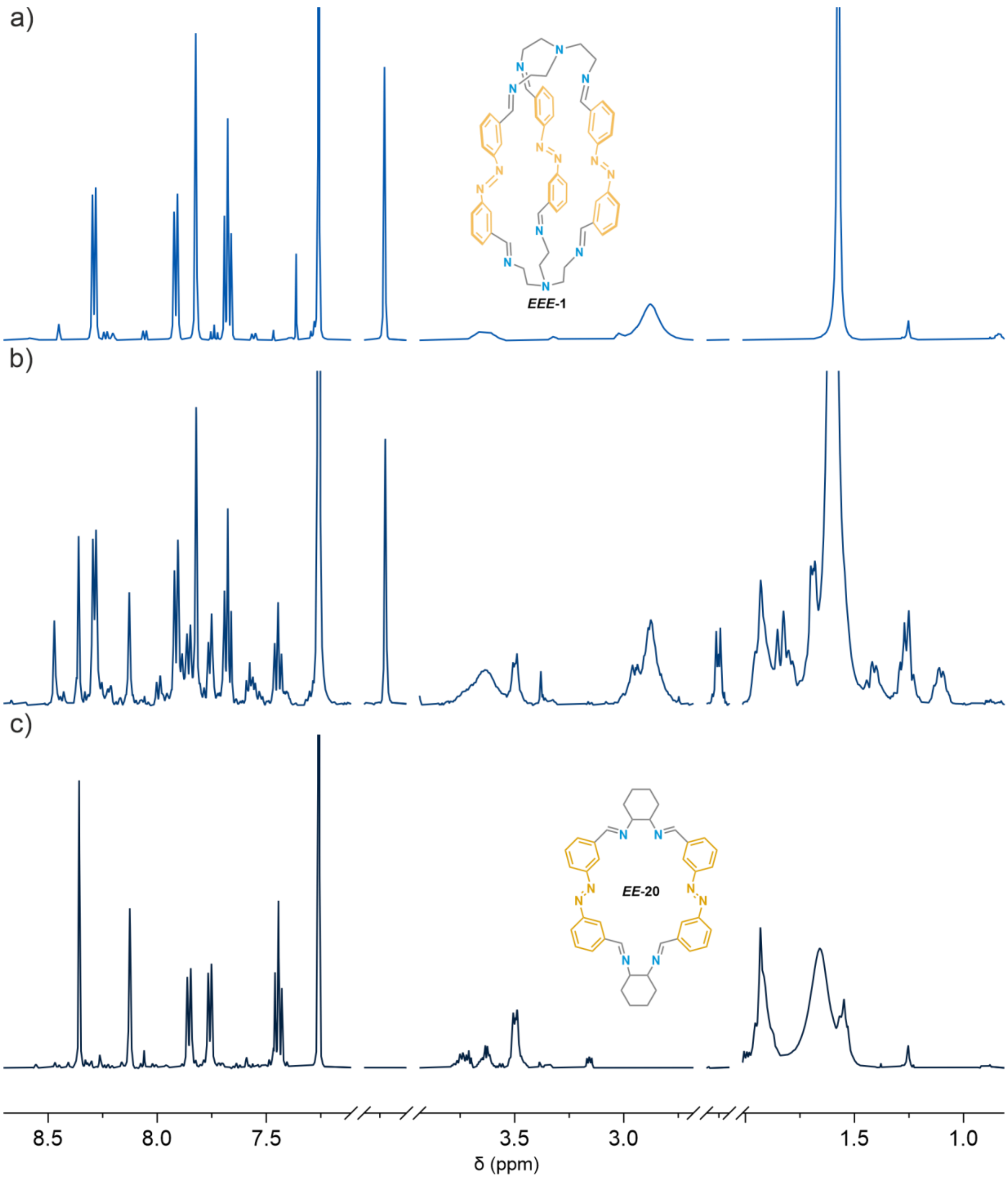


S50. the acidic solution, **b**, the self-sorted system of compounds ***E*-2** (3 mM) + **TREN** (2mM) + **10** (3mM) → ***EEE*-1** + ***EE*-19** + after 16 h in the dark and **c**, self-assembled macrocycle ***EE*-19**.

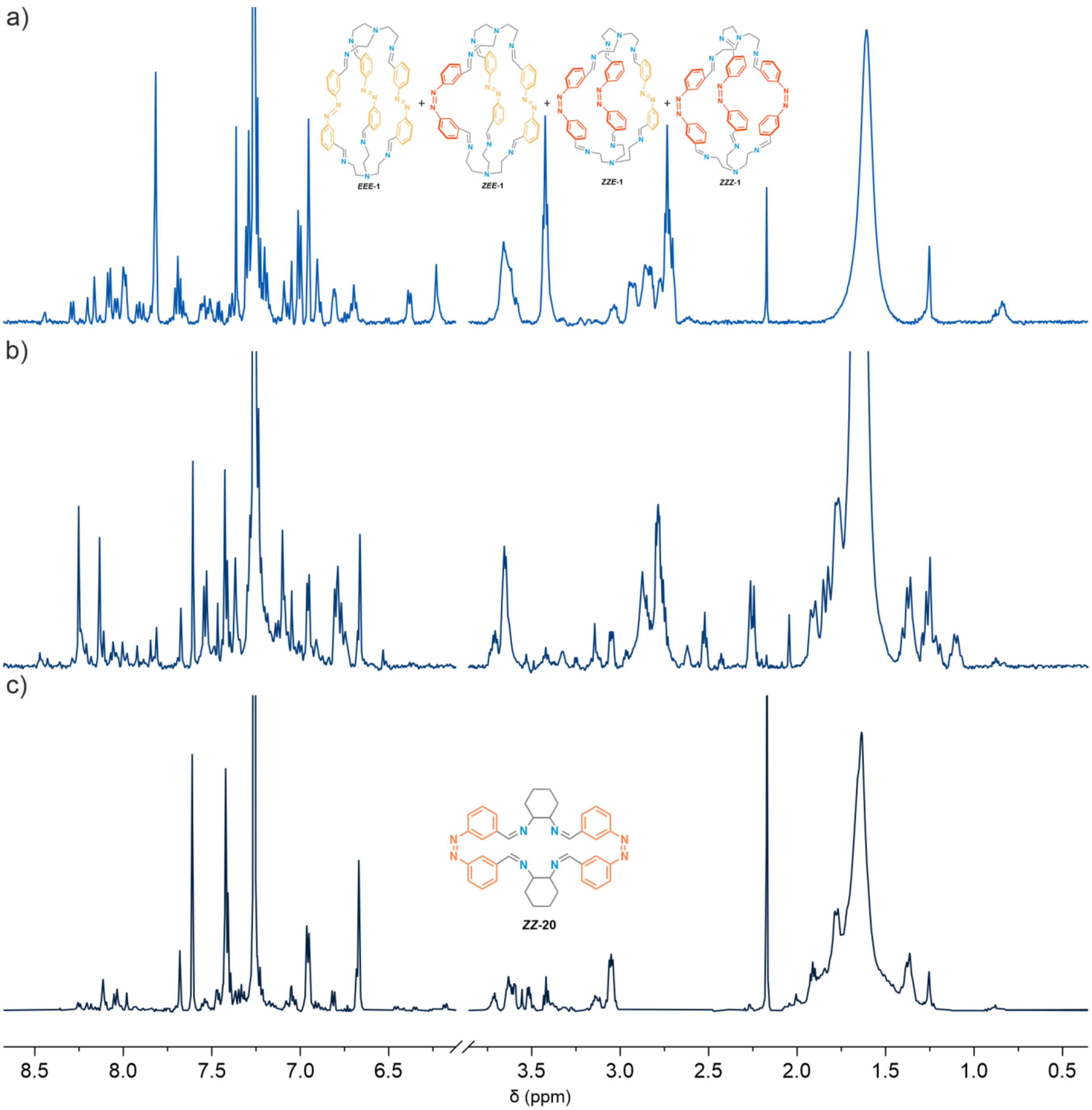


S51. [1]H NMR (500 MHz, $CDCl_3$, TFA 5 µM, 25 °C) stacked spectra comparing **a**, cage **1** irradiated with UV light ($\lambda_{irr}$ = 340 nm) generating a mixture of isomers of **1** (***EEE*-1**, ***ZEE*-1**, ***ZZE*-1** and ***ZZZ*-1**), **b**, the self-sorted system of compounds ***E*-2** (3 mM) + **TREN** (2mM) + **10** (3mM) after the light-fueled cage-to-macrocycle transformation ***EEE*-1** + ***EE*-19** → ***ZZ*-19** and **c**, irradiated sample of self-assembled system containing macrocycle ***ZZ*-19**.

## DOSY NMR spectra of macrocycles 3-4 and 11-19

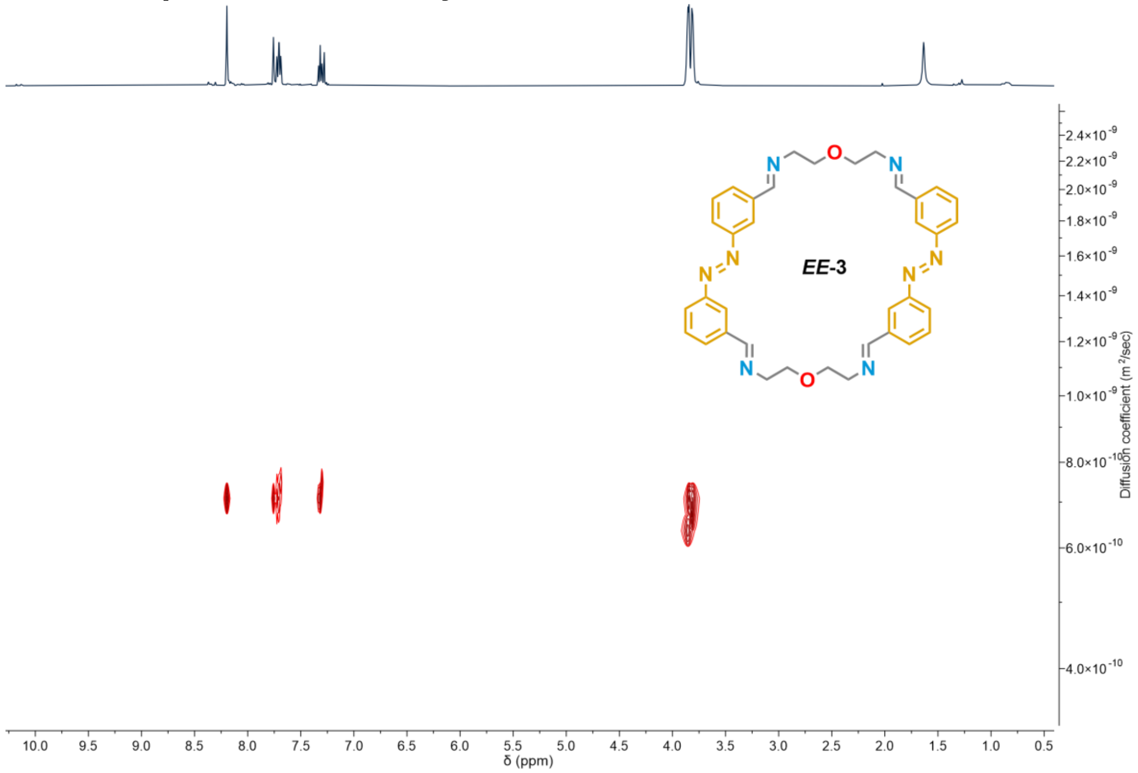


S52. DOSY [1]H NMR (500 MHz, $CDCl_3$, TFA 5 µM, 25 °C) of the self-assembled macrocycle ***EE*-3**.

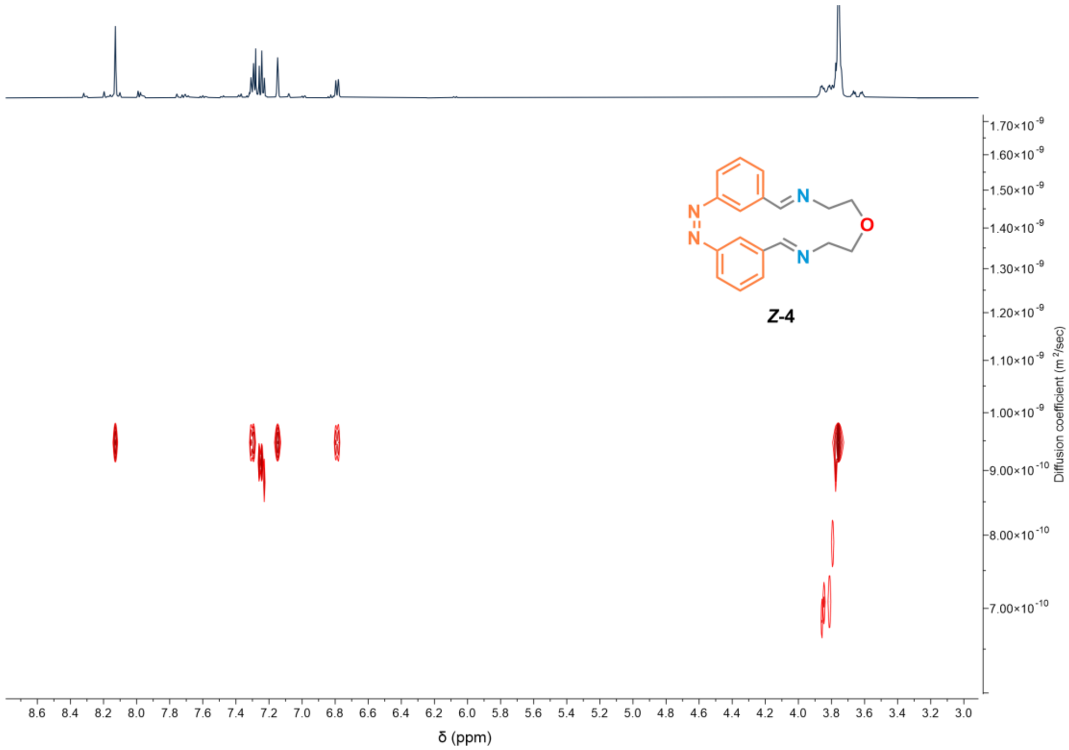


S53. DOSY [1]H NMR (500 MHz, $CDCl_3$, TFA 5 µM, 25 °C) of the macrocycle ***Z*-4** generated by irradiation ($\lambda_{irr}$ = 340 nm, 3 h) of ***EE*-3**.

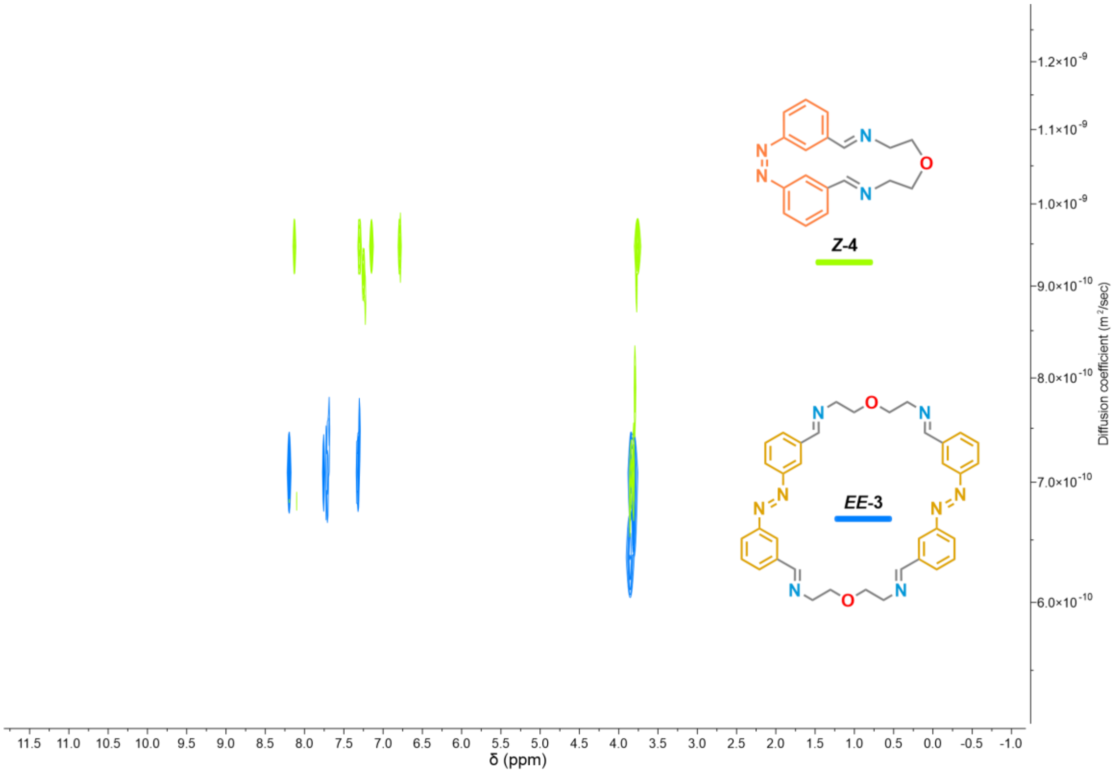


S54. DOSY [1]H NMR (500 MHz, $CDCl_3$, TFA 5 µM, 25 °C) overlay of macrocycles ***EE*-3** (blue) and ***Z*-4** (green).

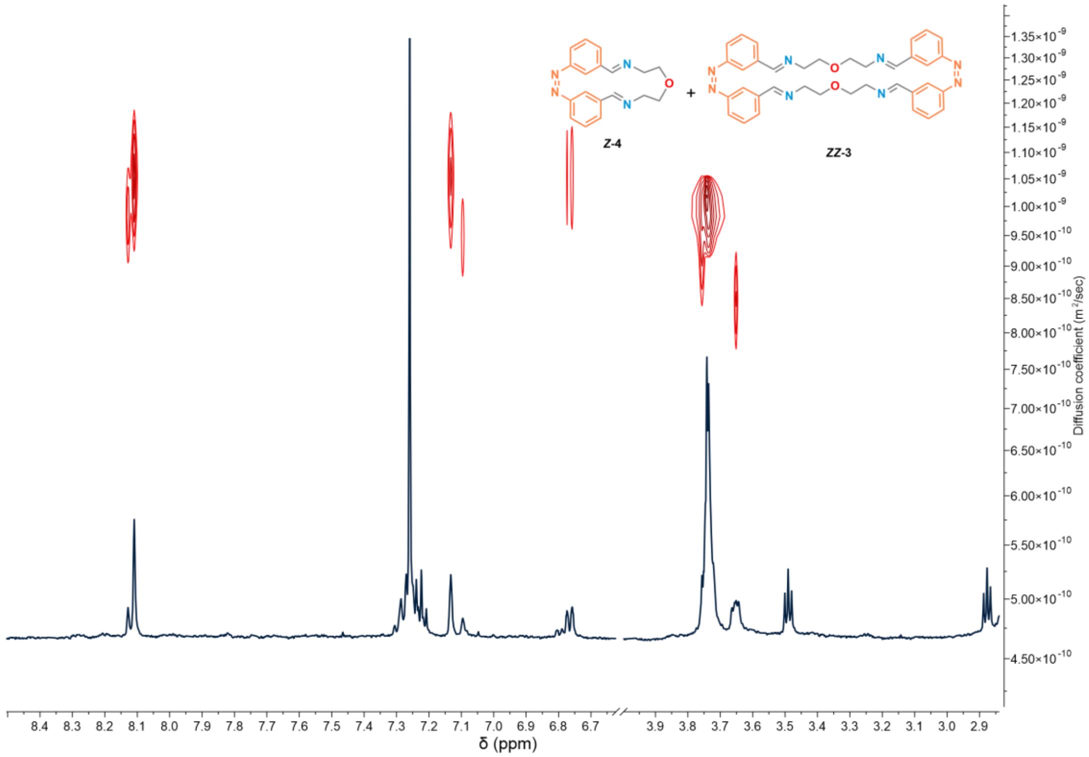


S55. DOSY [1]H NMR (500 MHz, $CDCl_3$, TFA 5 µM, 25 °C) of macrocycles generated from the light-fuelled cage-to-macrocycle transformation **1** + **NON** → ***Z*-4** + ***ZZ*-3** immediately after irradiation.

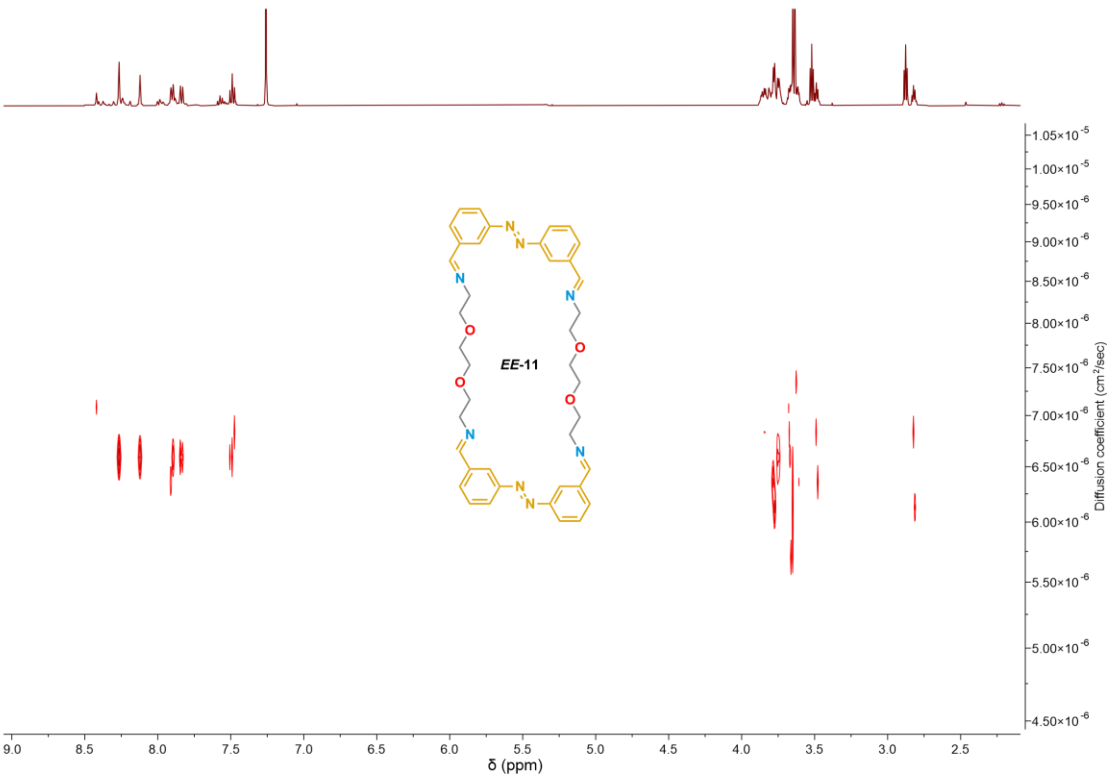


S56. DOSY [1]H NMR (500 MHz, $CDCl_3$, TFA 5 µM, 25 °C) of the self-assembled macrocycle ***EE*-11**.

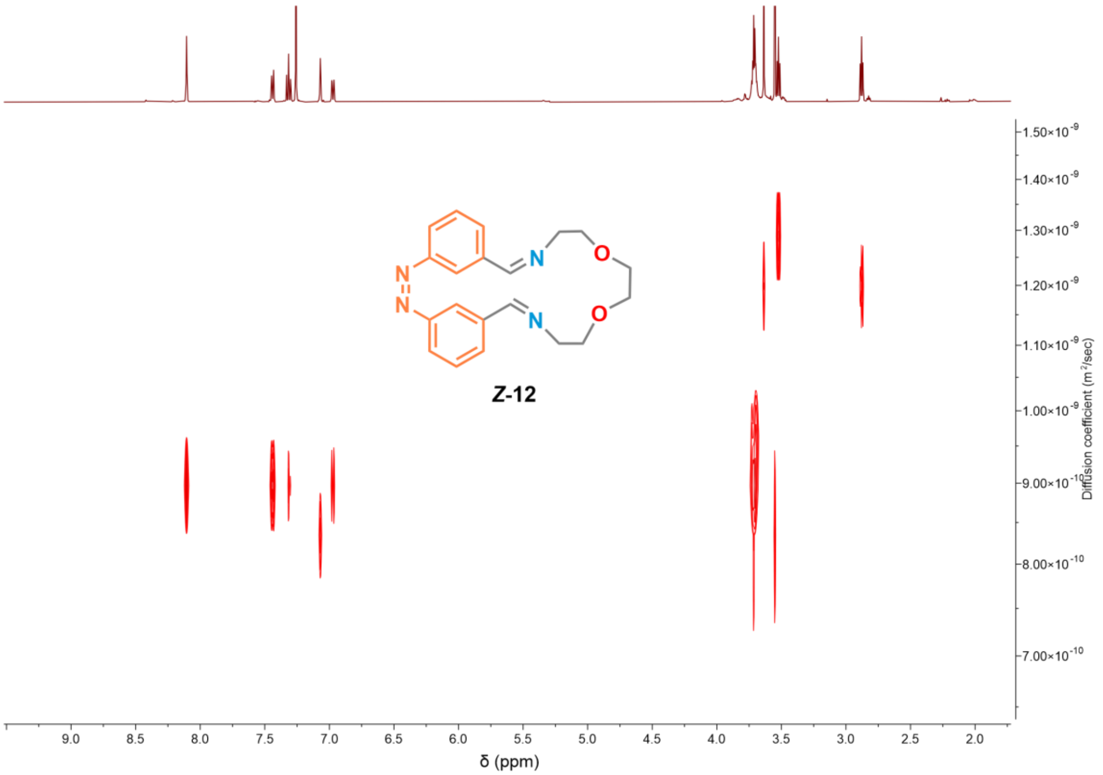


S57. DOSY [1]H NMR (500 MHz, $CDCl_3$, TFA 5 µM, 25 °C) of the macrocycle ***Z*-12** generated by irradiation ($\lambda_{irr}$ = 340 nm, 3 h) of ***EE*-3**.

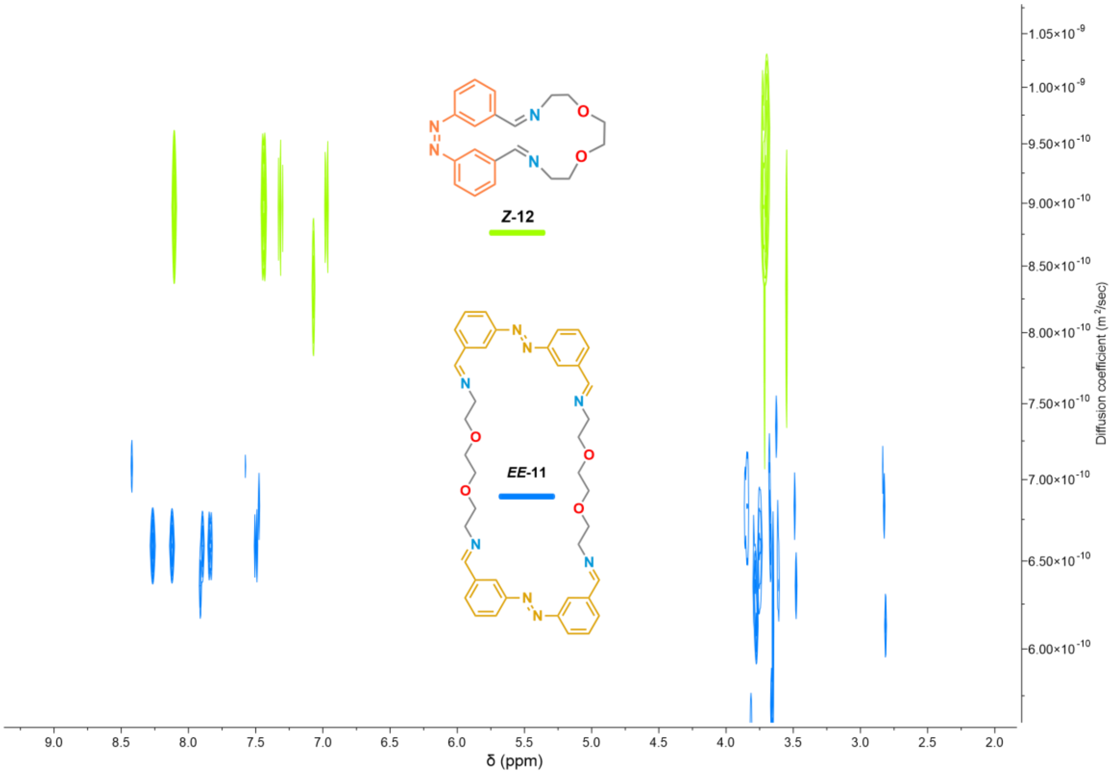


S58. DOSY $^1$H NMR (500 MHz, $CDCl_3$, TFA 5 µM, 25 °C) overlay of macrocycles ***EE*-11** (blue) and ***Z*-12** (green).

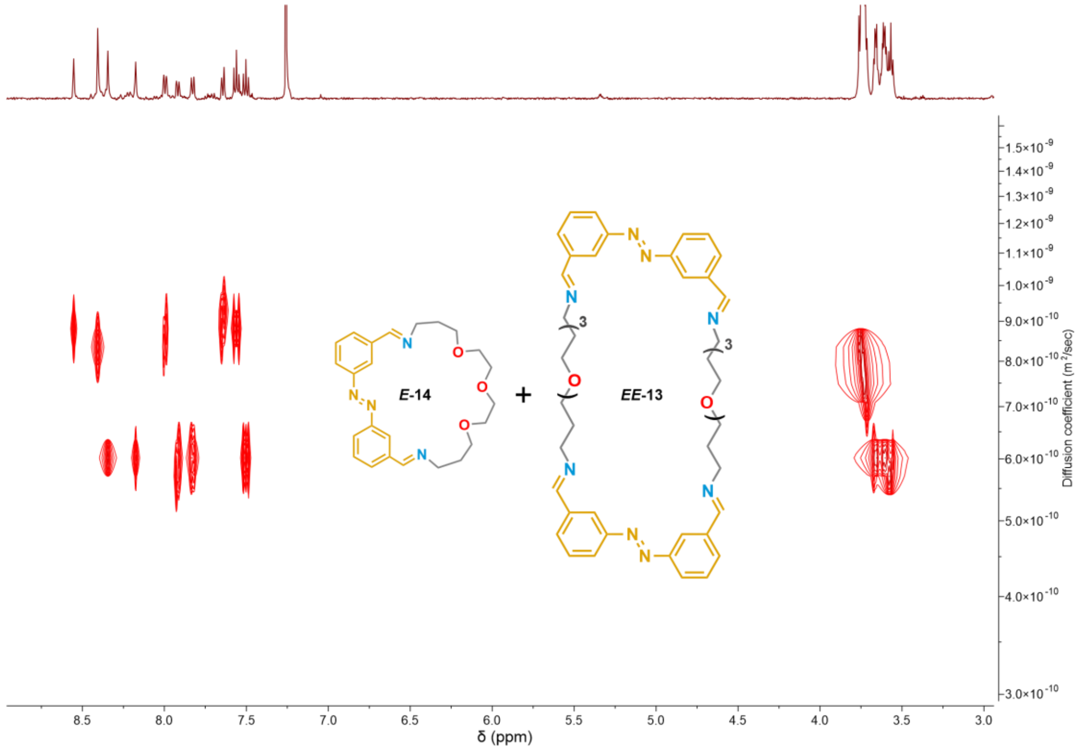


S59. DOSY $^1$H NMR (500 MHz, $CDCl_3$, TFA 5 µM, 25 °C) of the self-assembled macrocycles ***EE*-13** and ***E*-14**.

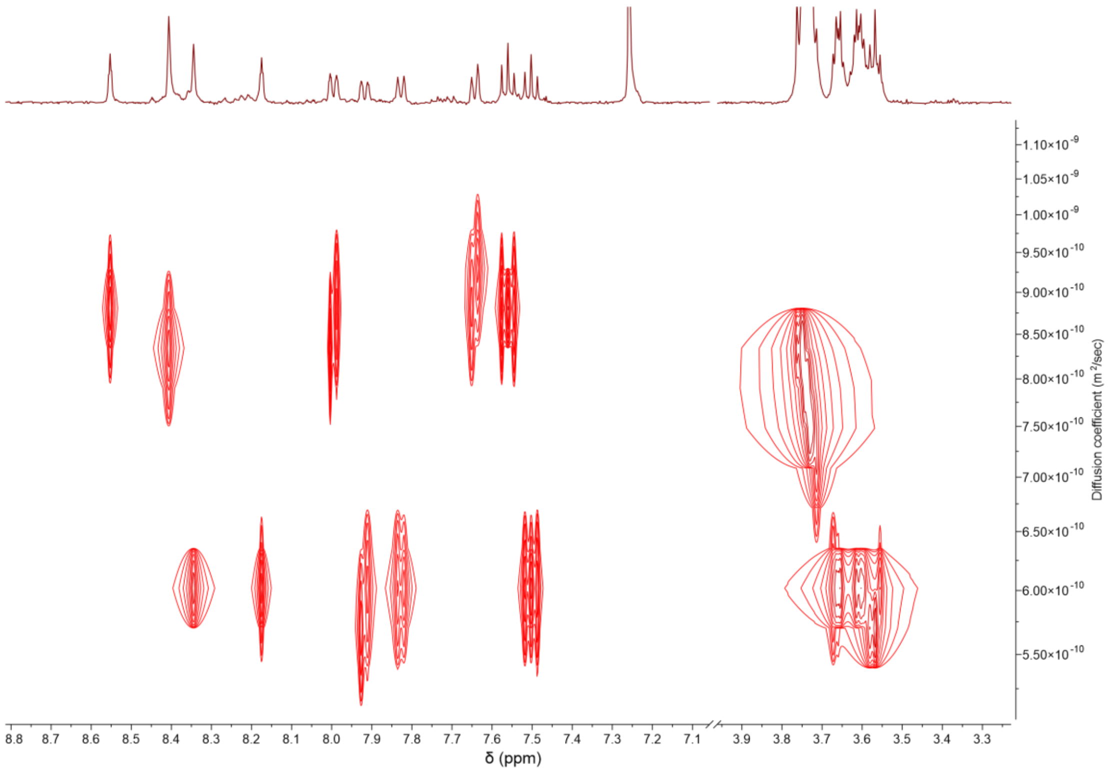


S60. DOSY $^{1}H$ NMR (500 MHz, $CDCl_3$, TFA 5 µM, 25 °C) expansion of the macrocycles ***EE*-13** and ***E*-14**.

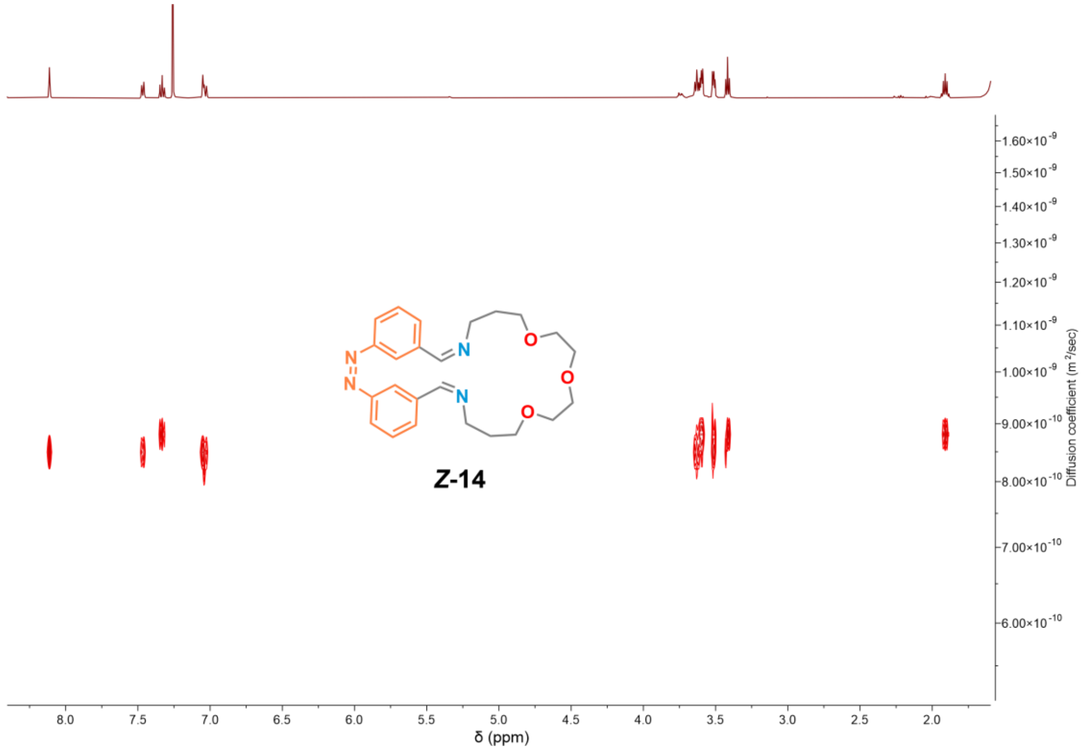


S61. DOSY $^{1}H$ NMR (500 MHz, $CDCl_3$, TFA 5 µM, 25 °C) of the macrocycle ***Z*-14** generated by irradiation ($\lambda_{irr}$ = 340 nm, 3 h) of macrocycles ***EE*-13** and ***E*-14**.

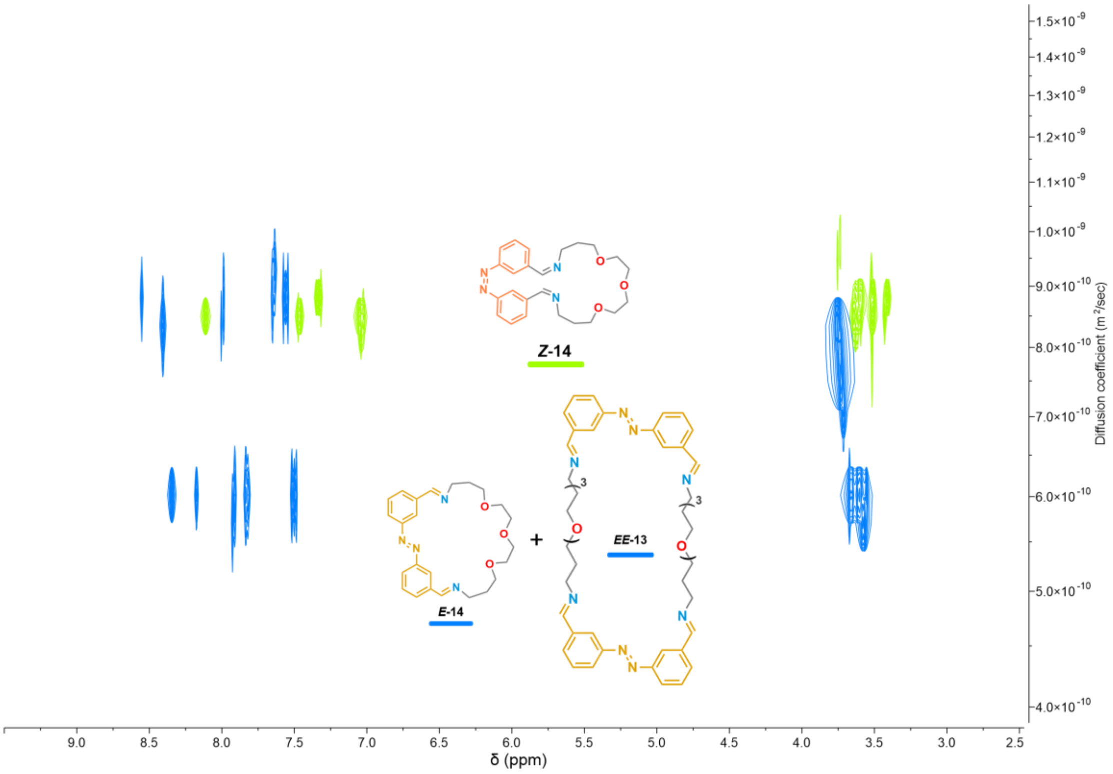


S62. DOSY $^{1}$H NMR (500 MHz, $CDCl_3$, TFA 5 µM, 25 °C) overlay of the self-assembled macrocycles ***EE*-13** and ***E*-14** (blue) and the macrocycle ***Z*-4** (green) formed after irradiation ($\lambda_{irr}$ = 340 nm, 3 h).

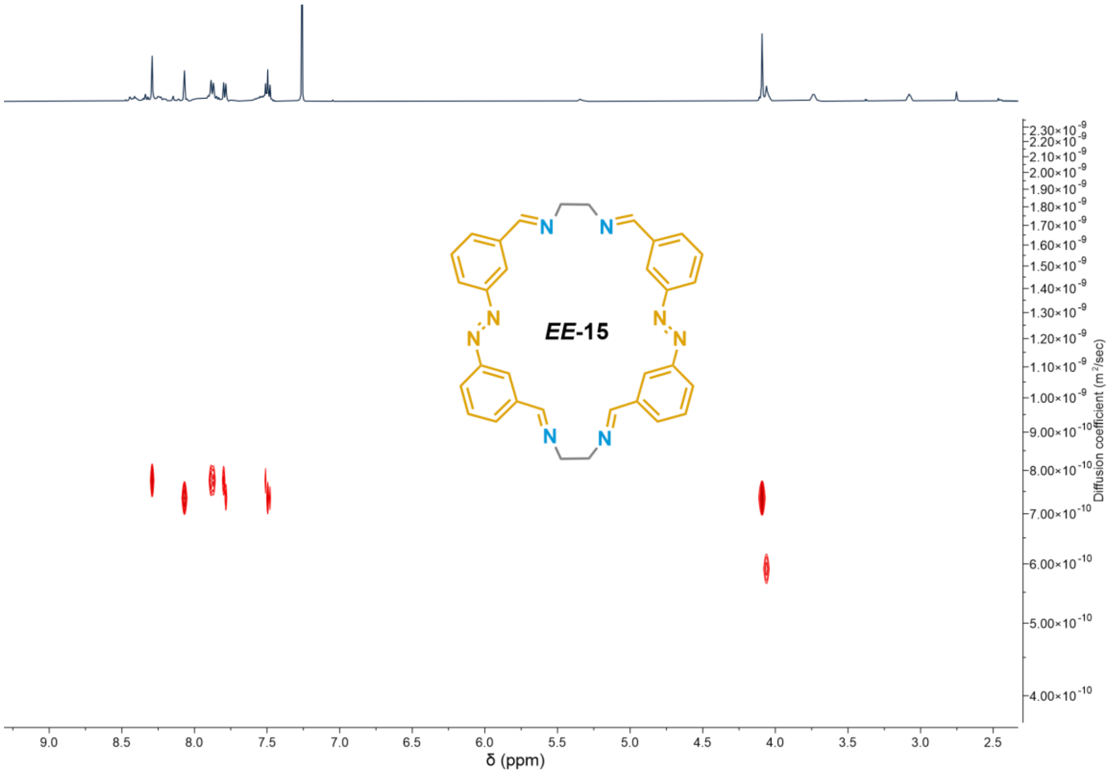


S63. DOSY $^{1}$H NMR (500 MHz, $CDCl_3$, TFA 5 µM, 25 °C) of the self-assembled macrocycle ***EE*-15**.

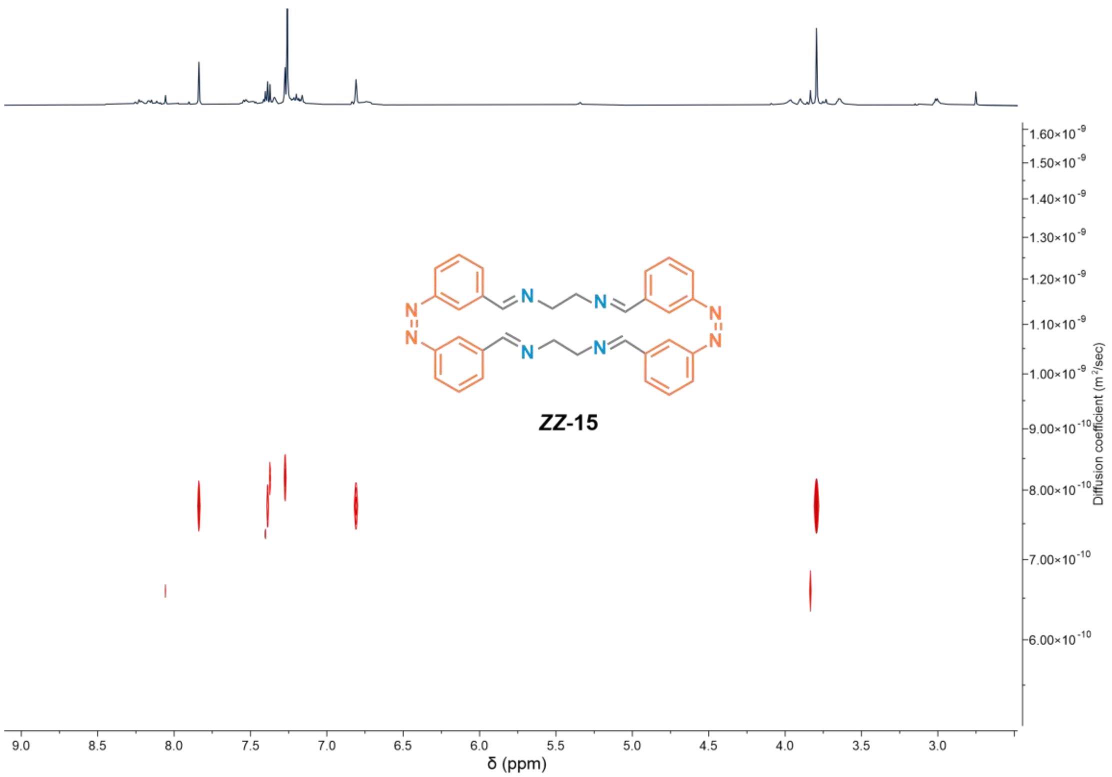


S64. DOSY $^{1}H$ NMR (500 MHz, $CDCl_3$, TFA 5 µM, 25 °C) of the macrocycle ***ZZ*-15** generated by irradiation ($\lambda_{irr}$ = 340 nm, 3 h) of ***EE*-15**.

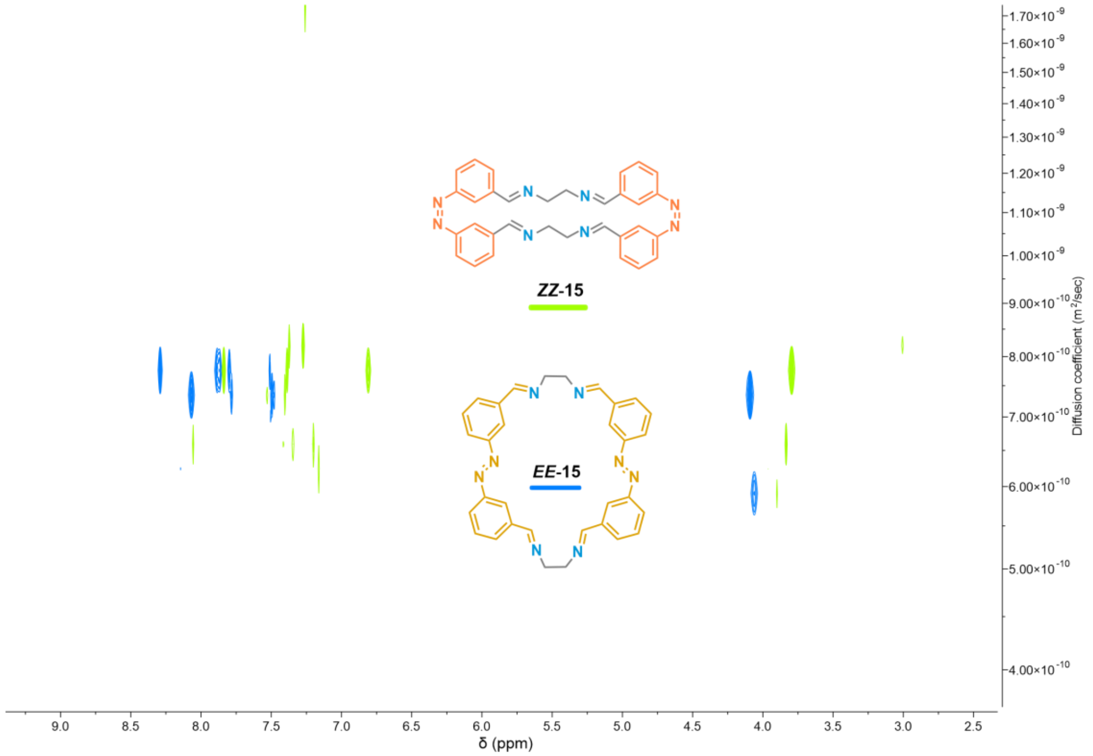


S65. DOSY $^{1}H$ NMR (500 MHz, $CDCl_3$, TFA 5 µM, 25 °C) overlay of macrocycles ***EE*-15** (blue) and ***ZZ*-15** (green).

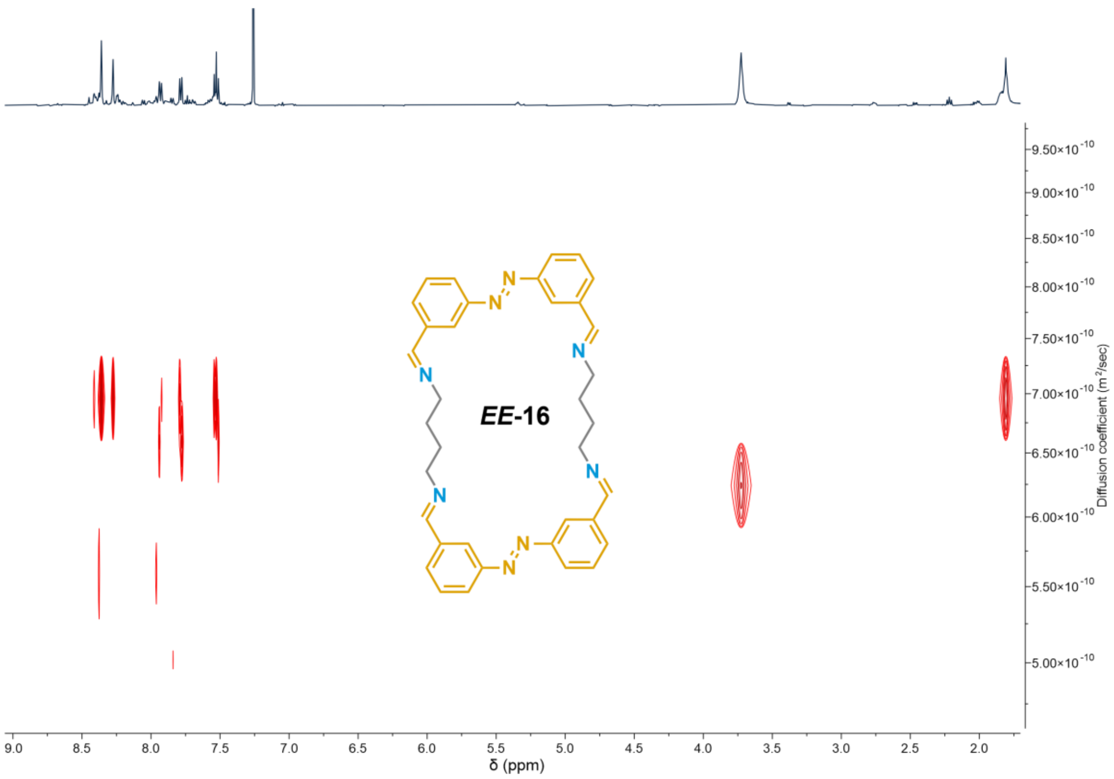


S66. DOSY [1]H NMR (500 MHz, $CDCl_3$, TFA 5 μM, 25 °C) of the self-assembled macrocycle ***EE*-16**.

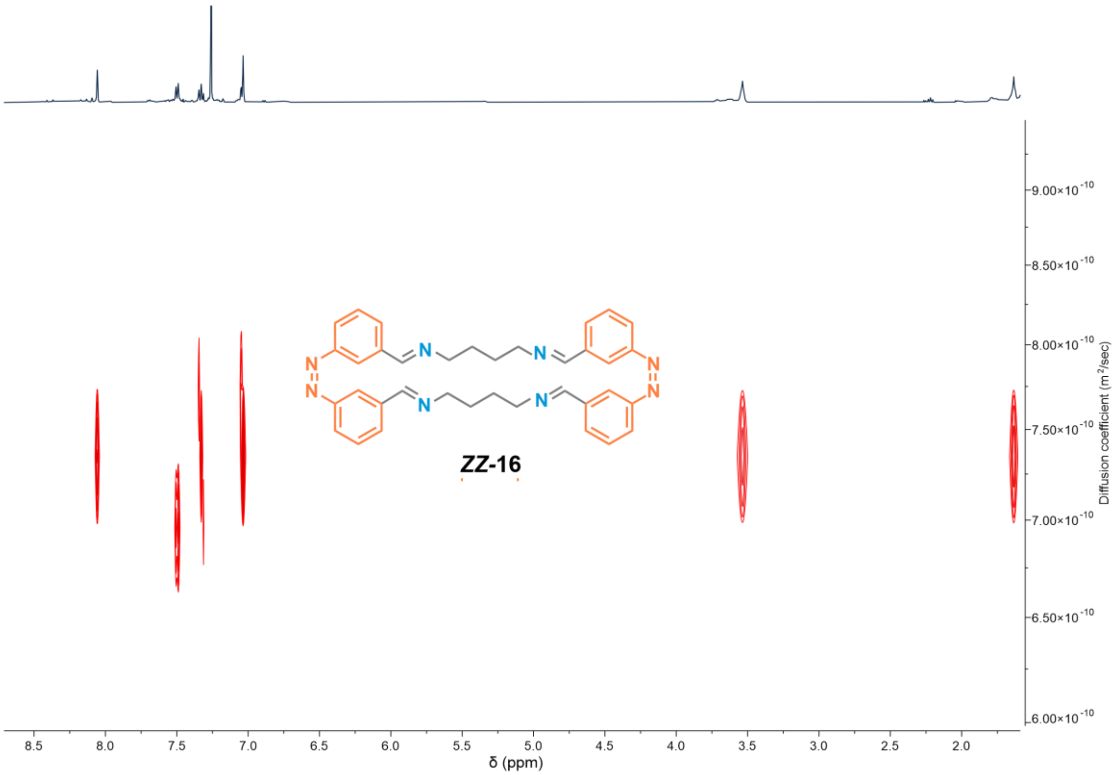


S67. DOSY [1]H NMR (500 MHz, $CDCl_3$, TFA 5 μM, 25 °C) of the macrocycle ***ZZ*-16** generated by irradiation ($\lambda_{irr}$ = 340 nm, 3 h) of ***EE*-16**.

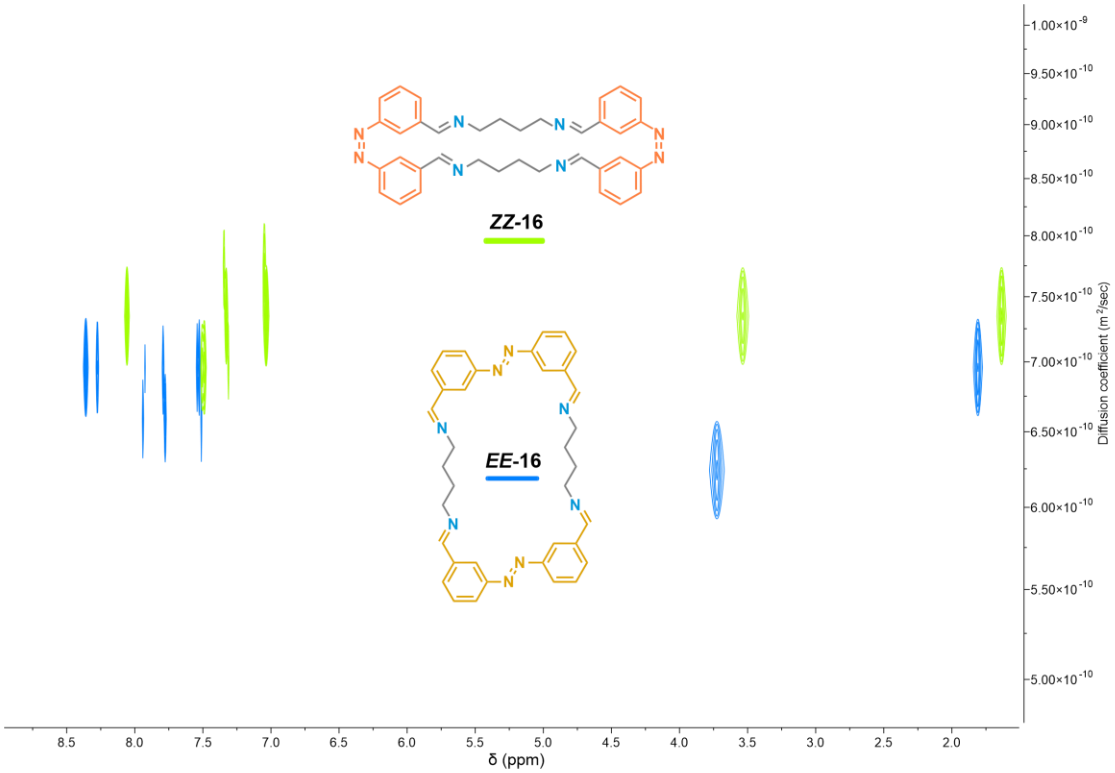


S68. DOSY ¹H NMR (500 MHz, $CDCl_3$, TFA 5 µM, 25 °C) overlay of macrocycles ***EE*-16** (blue) and ***ZZ*-16** (green).

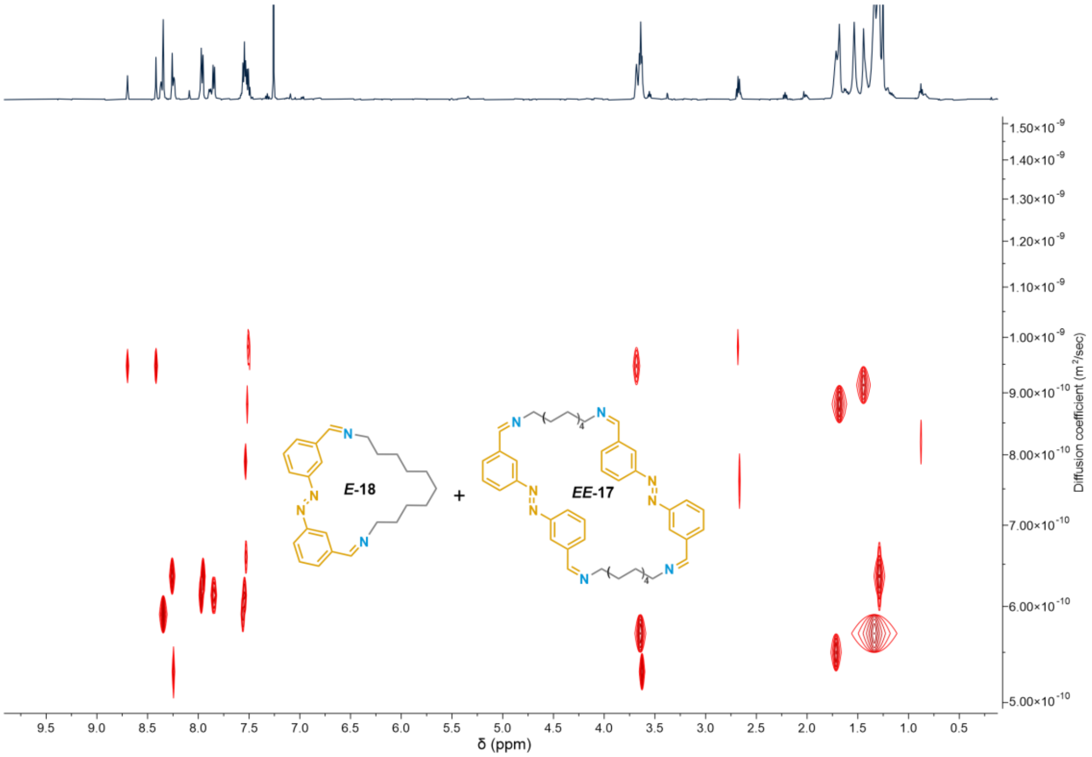


S69. DOSY ¹H NMR (500 MHz, $CDCl_3$, TFA 5 µM, 25 °C) of the self-assembled macrocycles ***EE*-17** and ***E*-18**.

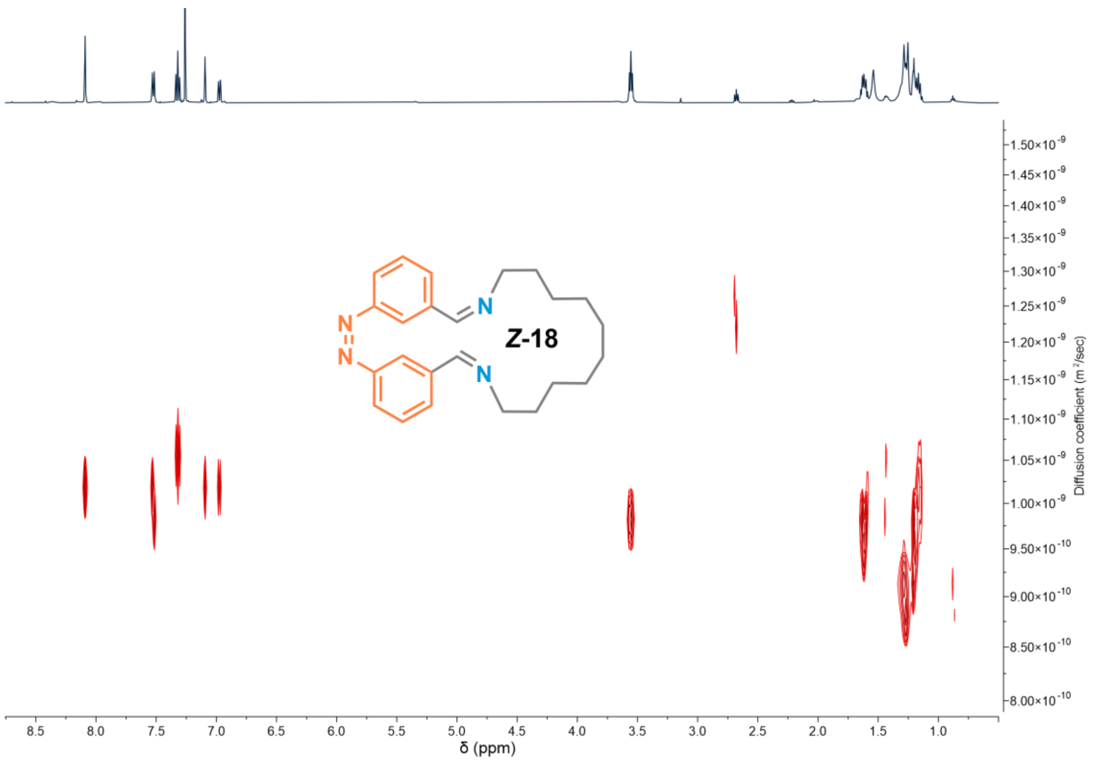


S70. DOSY $^{1}H$ NMR (500 MHz, $CDCl_3$, TFA 5 µM, 25 °C) of the macrocycle ***Z*-18** generated by irradiation ($\lambda_{irr}$ = 340 nm, 3 h) of ***EE*-17** and ***E*-18**.

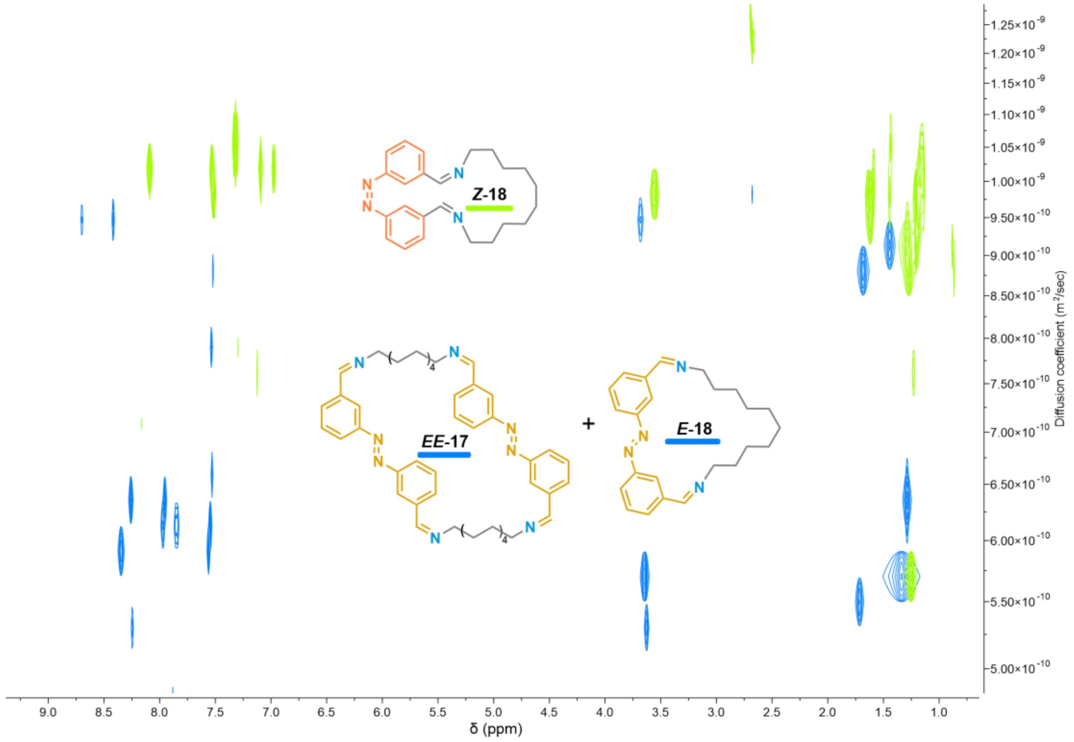


S71. DOSY $^{1}H$ NMR (500 MHz, $CDCl_3$, TFA 5 µM, 25 °C) overlay spectra of the self-assembled macrocycles ***EE*-17** and ***E*-18** (blue) and the macrocycle ***Z*-18** (green) formed after irradiation ($\lambda_{irr}$ = 340 nm, 3 h).

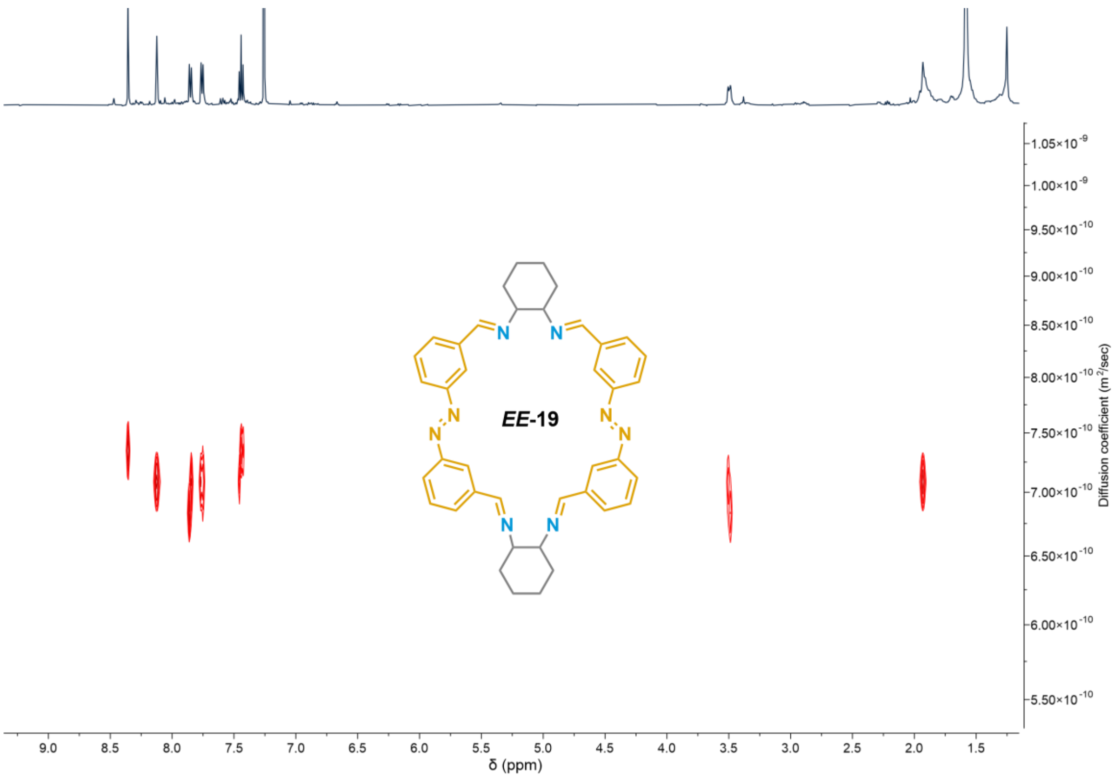


S72. DOSY $^{1}$H NMR (500 MHz, $CDCl_3$, TFA 5 µM, 25 °C) of the self-assembled macrocycle ***EE*-19**.

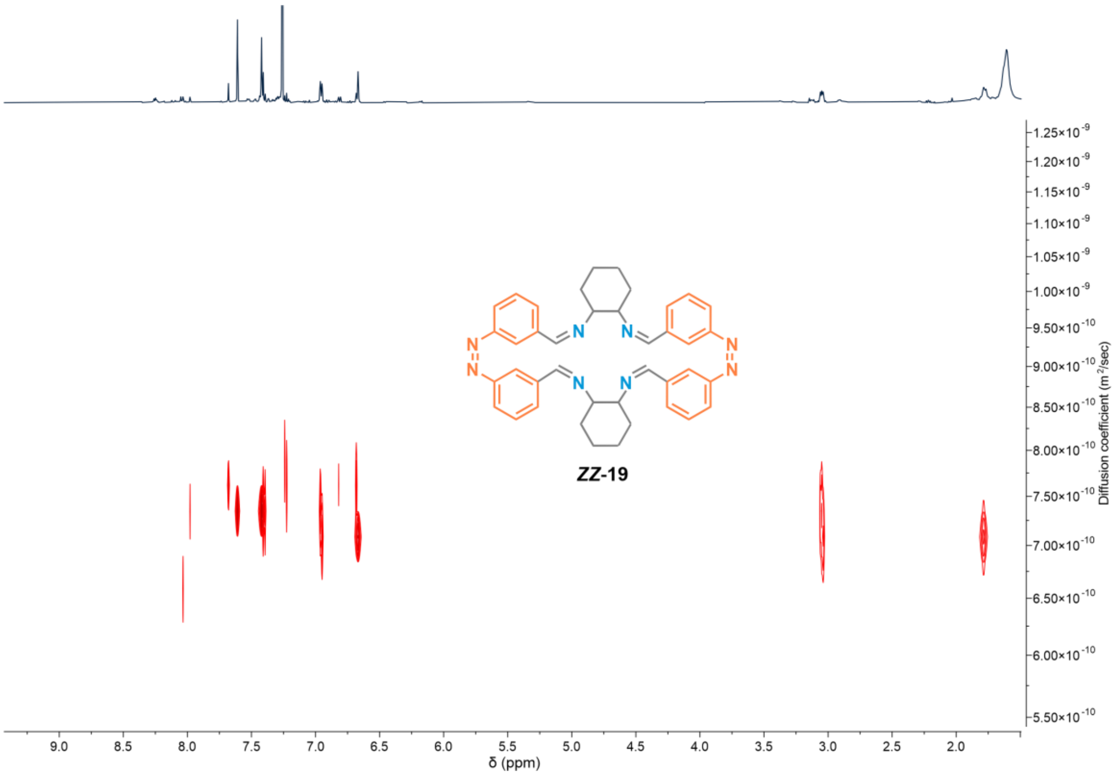


S73 DOSY $^{1}$H NMR (500 MHz, $CDCl_3$, TFA 5 µM, 25 °C) of the macrocycle ***ZZ*-19** generated by irradiation ($\lambda_{irr}$ = 340 nm, 3 h) of ***EE*-19**.

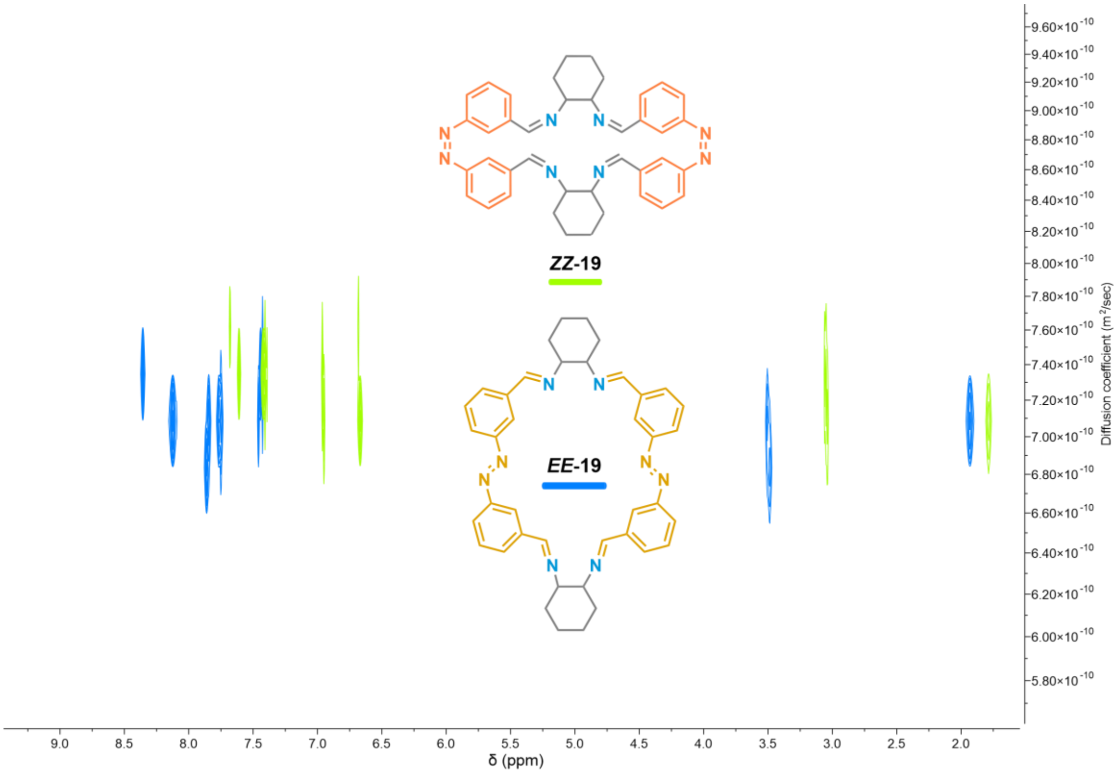


S74. DOSY $^1H$ NMR (500 MHz, $CDCl_3$, TFA 5 µM, 25 °C) overlay of macrocycles ***EE*-19** (blue) and ***ZZ*-19** (green).

# ESI-HRMS of self-assembled macrocycles

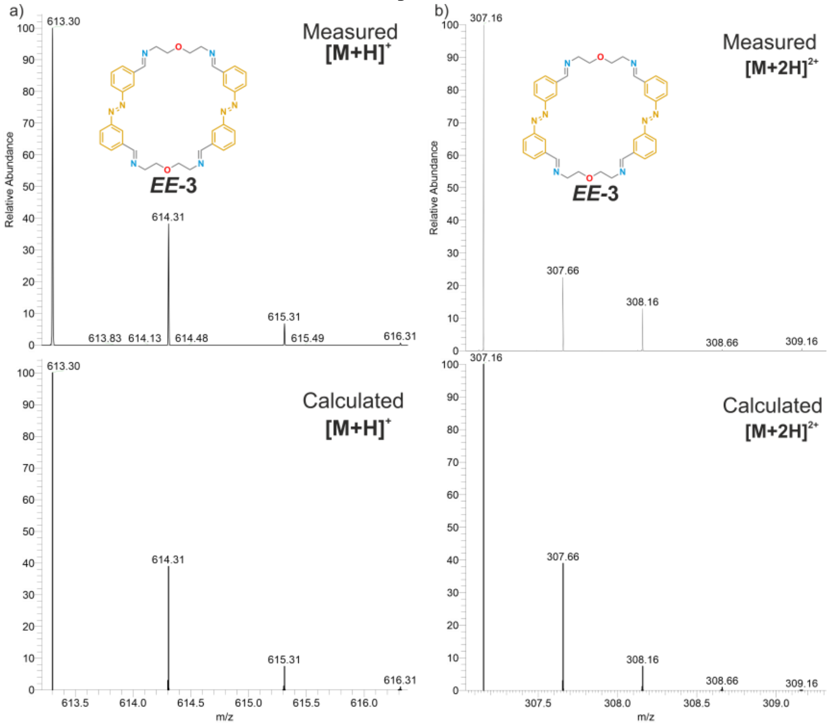


S75. Expansion of the signals for the molecular ion and comparation with the calculated spectra of **a**) singly $[M+H]^+$ and **b**) doubly ionized $[M+2H]^{2+}$ species of ***EE*-3**.

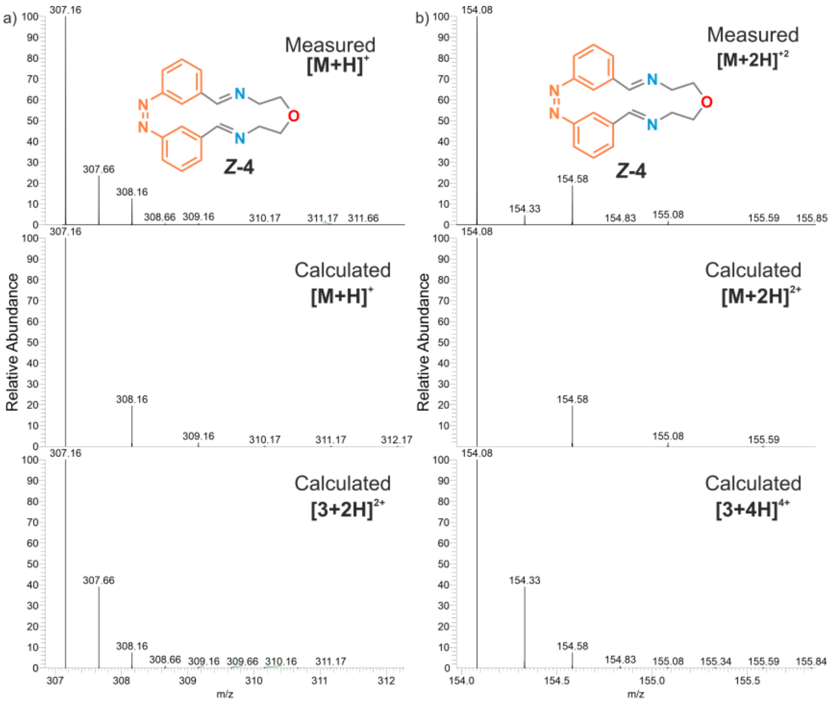


S76. HRMS-ESI spectra of macrocycle ***Z*-4** generated by irradiation ($\lambda_{irr}$ = 340 nm, 3 h) of ***EE*-3**. The signals for the molecular ion singly $[M+H]^+$ and doubly ionized $[M+2H]^{2+}$ are shown at 307.16 m/z and 154.08 m/z, respectively.

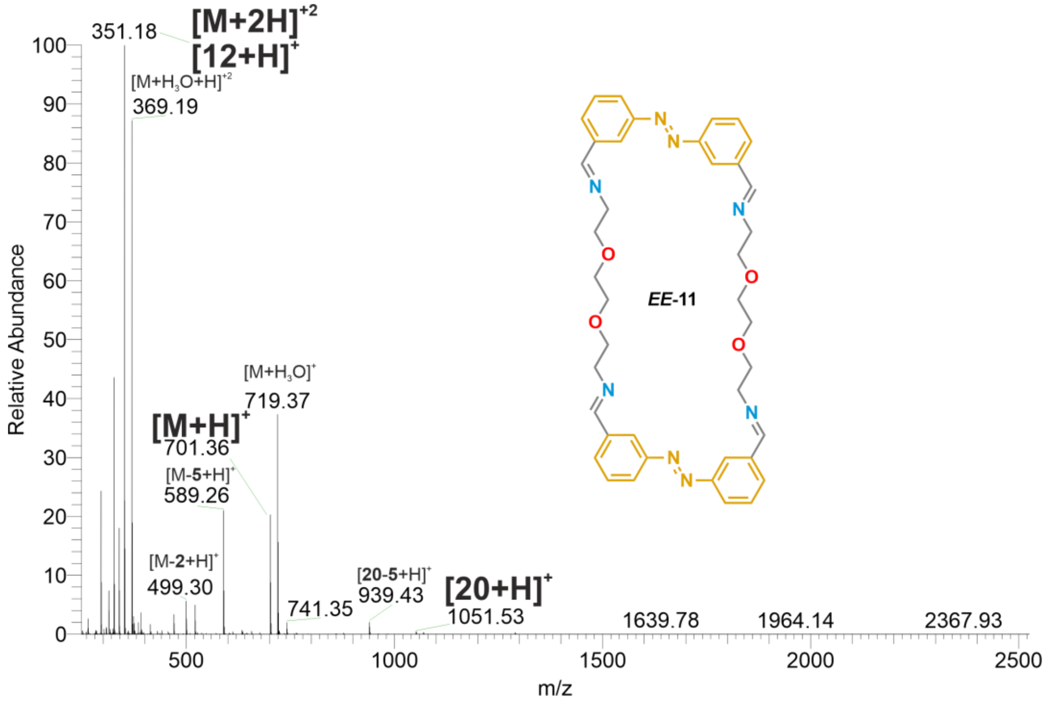


S77. HRMS-ESI spectra of self-assembled macrocycle **11** up to 2500 m/z. The signals corresponding to the singly ionized $[M+H]^+$ and doubly ionized $[M+2H]^{2+}$ are shown at 701.36 m/z and 351.18 m/z, respectively. Additionally, signals that correspond to the [1+1] and [3+3] macrocycles **12** and **20** are also shown.

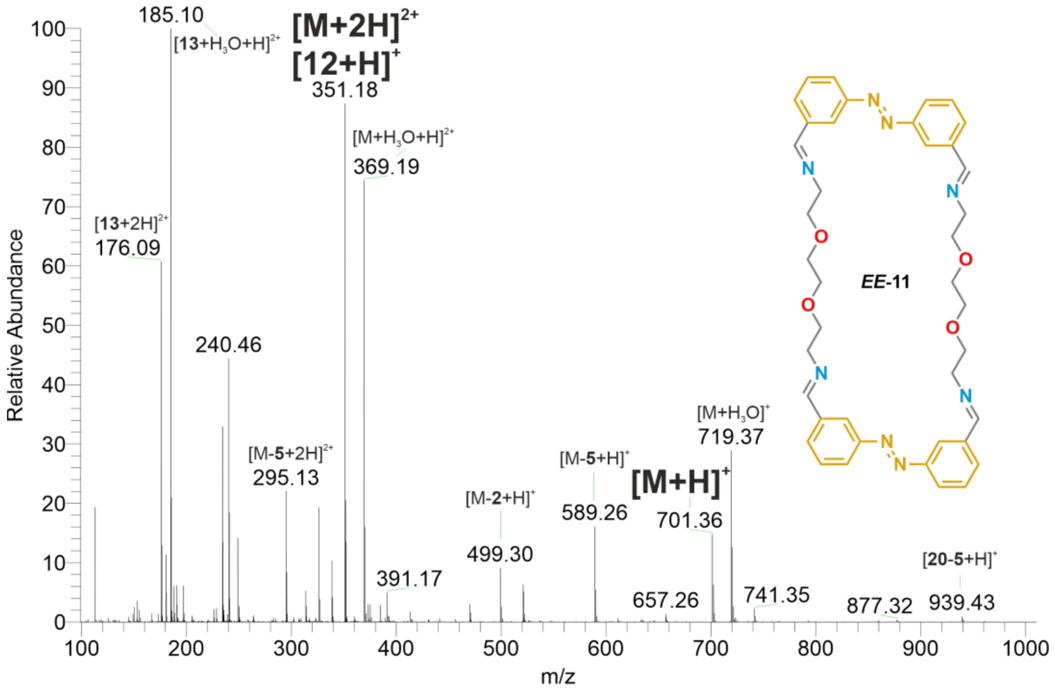


S78. HRMS-ESI spectra of self-assembled macrocycle **11** up to 1000 m/z. The signals for the singly ionized $[M+H]^+$ and doubly ionized $[M+2H]^{2+}$ are shown at 701.36 m/z and 351.18 m/z, respectively. Additionally, signals that correspond to the [1+1] and [3+3] macrocycles **12** and **20** are also shown.

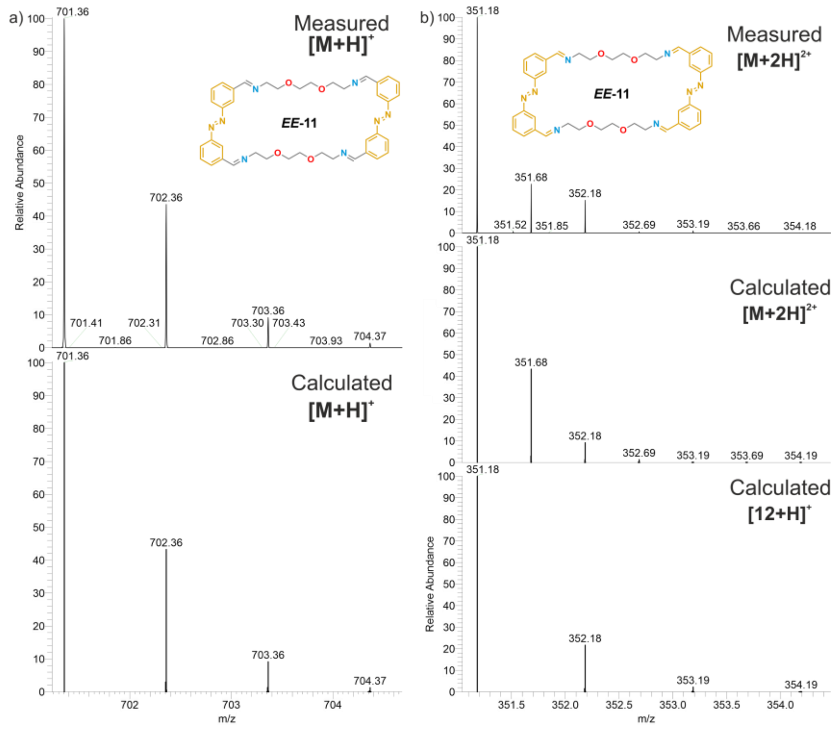


S79. Expansion of the signals for ESI-HRMS and comparation with the calculated spectra of **11**. **a**) singly charged [**M**+H]$^+$ and **b**) the isobaric species [**M**+H]$^+$ and [**12**+2H]$^{+2}$.

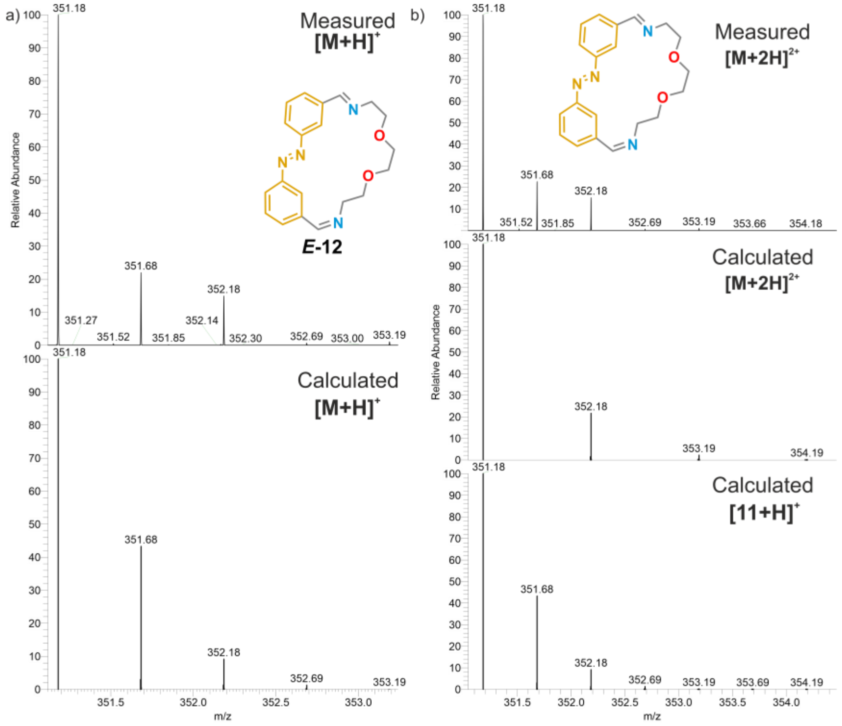


S80. Expansion of the signals for ESI-HRMS and comparation with the calculated spectra of **12**. **a**) singly charged [**M**+H]$^+$ and **b**) the isobaric species [**M**+H]$^+$ and [**11**+2H]$^{+2}$.

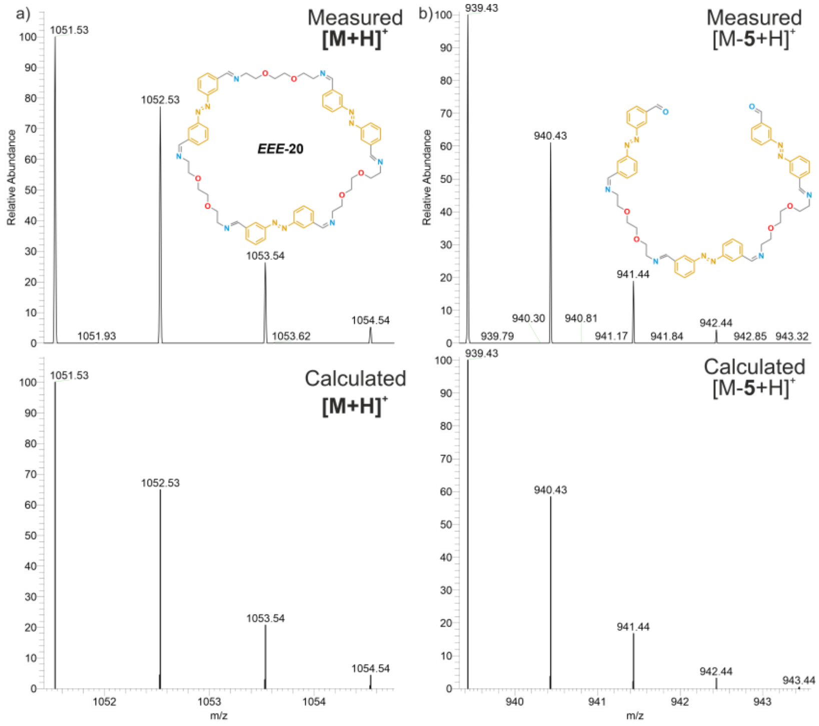


S81. Expansion of the signals for ESI-HRMS and comparation with the calculated spectra of **20**. **a**) singly charged [**M**+H]$^{+}$ and **b**) doubly charged [**M**+H2]$^{+2}$.

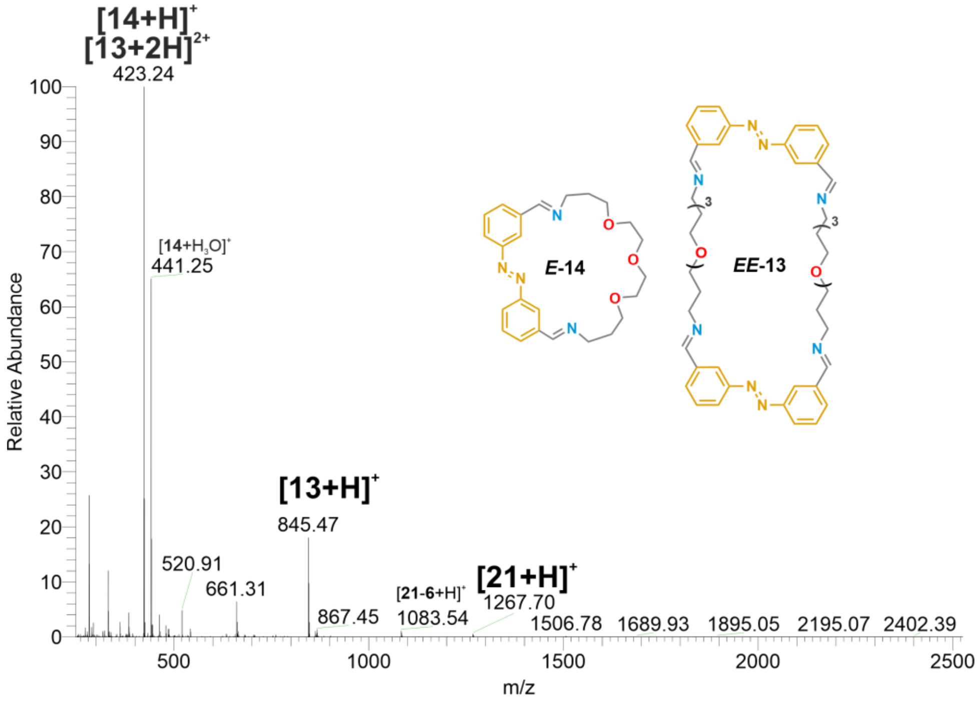


S82. HRMS-ESI spectra of self-assembled macrocycle **13** and **14** up to 2500 m/z. The signals corresponding to the molecular ion singly [**13**+H]$^{+}$ and the isobaric species [**13**+2H]$^{2+}$ and [**14**+2H]$^{2+}$ are shown at 1267. m/z and 423.24 m/z, respectively. Additionally, signals that correspond to the [3+3] macrocycle **21** are also shown at 1267.40 m/z.

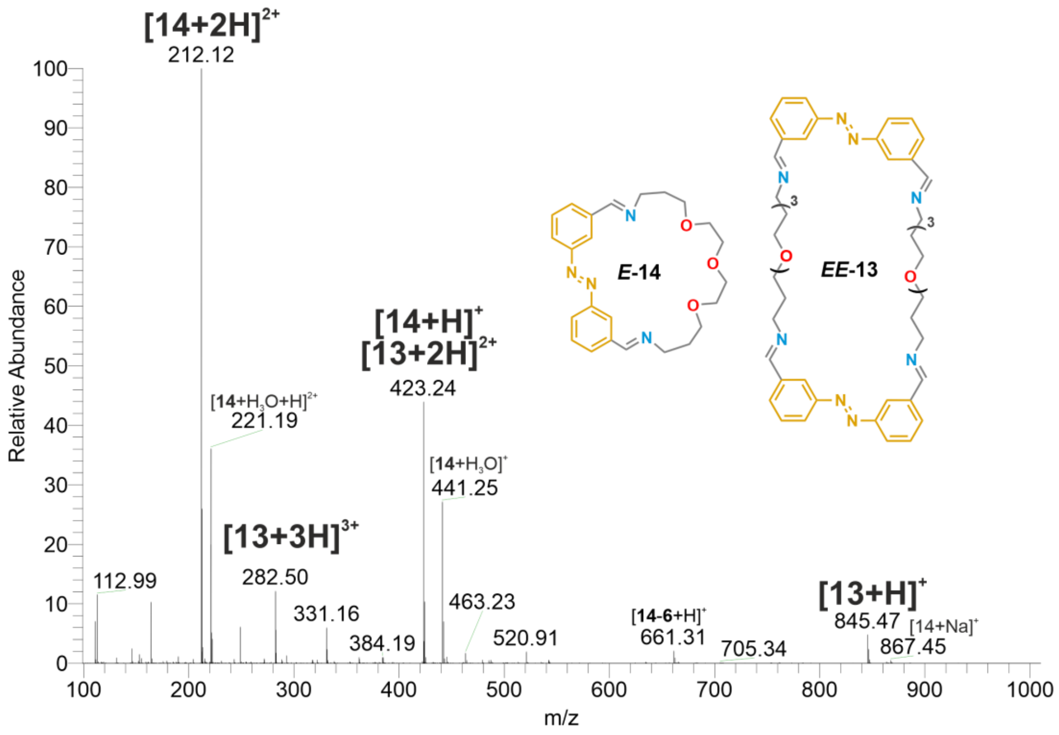


S83. HRMS-ESI spectra of self-assembled macrocycle **13** and **14** up to 1000 m/z. The signals corresponding to the molecular ion singly [**13**+H]+ and the isobaric species [**13**+2H]2+ and [**14**+2H]2+ are shown at 1267. m/z and 423.24 m/z, respectively.

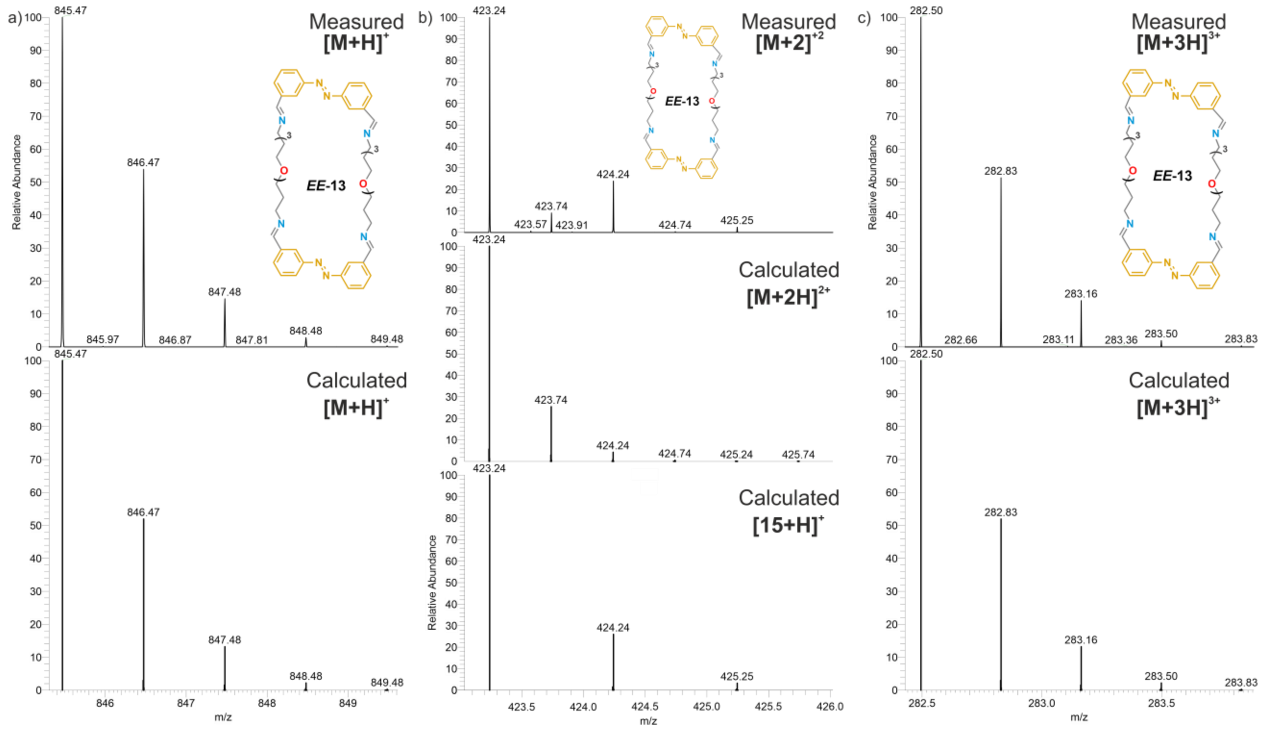


S84. Expansion of the signals for ESI-HRMS and comparation with the calculated spectra of **13**. **a**) singly charged [**M**+H]+, **b**) the isobaric species [**M**+2H]2+ and [**15**+H]+ and **c)** triply charged [**M**+3H]3+.

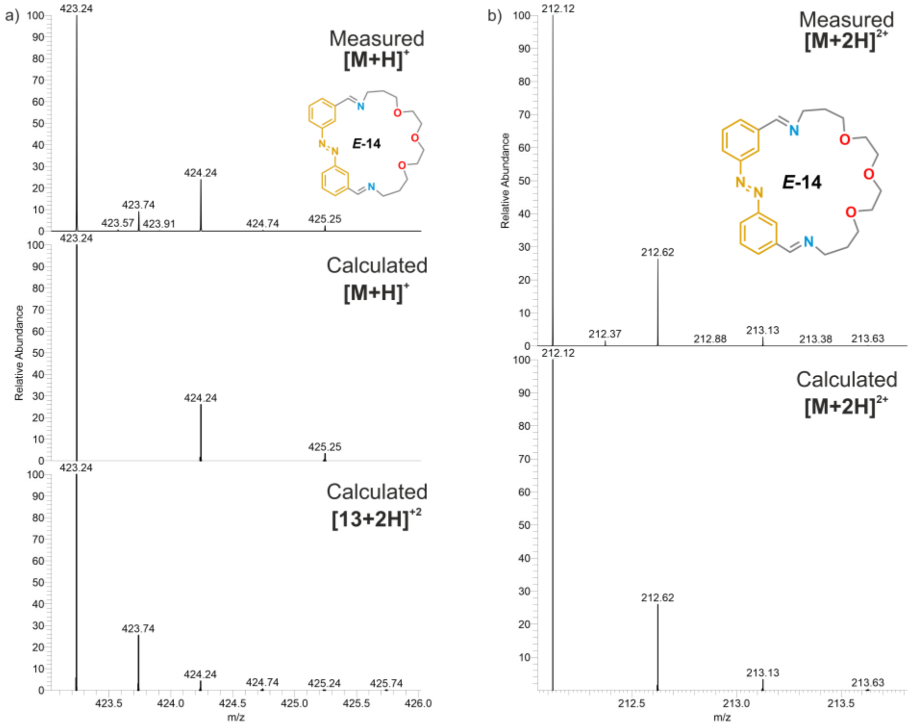


S85. Expansion of the signals for ESI-HRMS and comparation with the calculated spectra of **13**. **a**) isobaric species [**M**+H]$^{+}$ and [**13**+2H]$^{2+}$, **b)** doubly charged [**M**+2H]$^{2+}$.

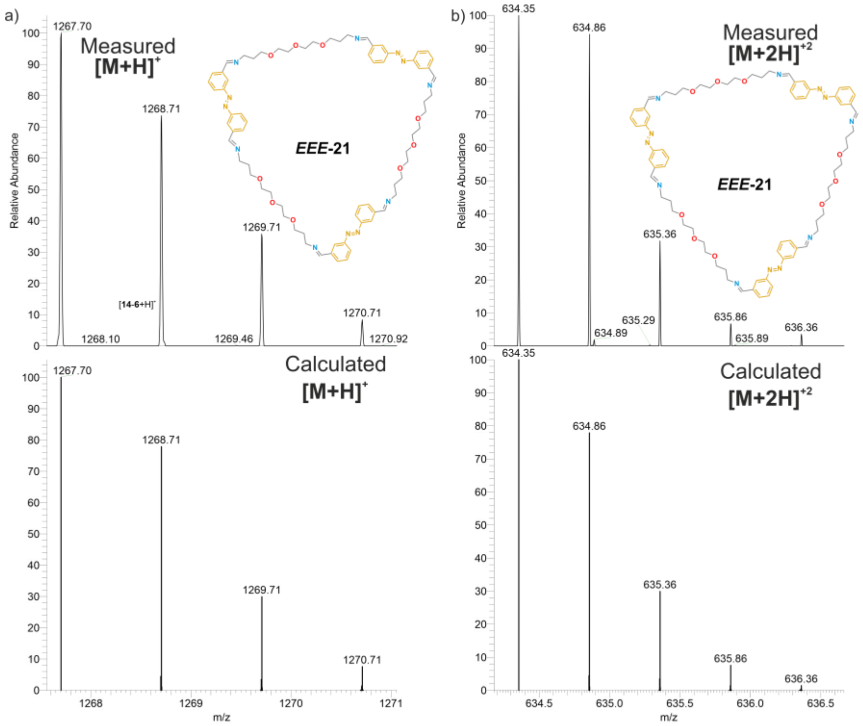


S86. Expansion of the signals for ESI-HRMS and comparation with the calculated spectra of **21. a**) singly charged [**M**+H]$^{+}$ and **b)** doubly charged [**M**+2H]$^{2+}$.

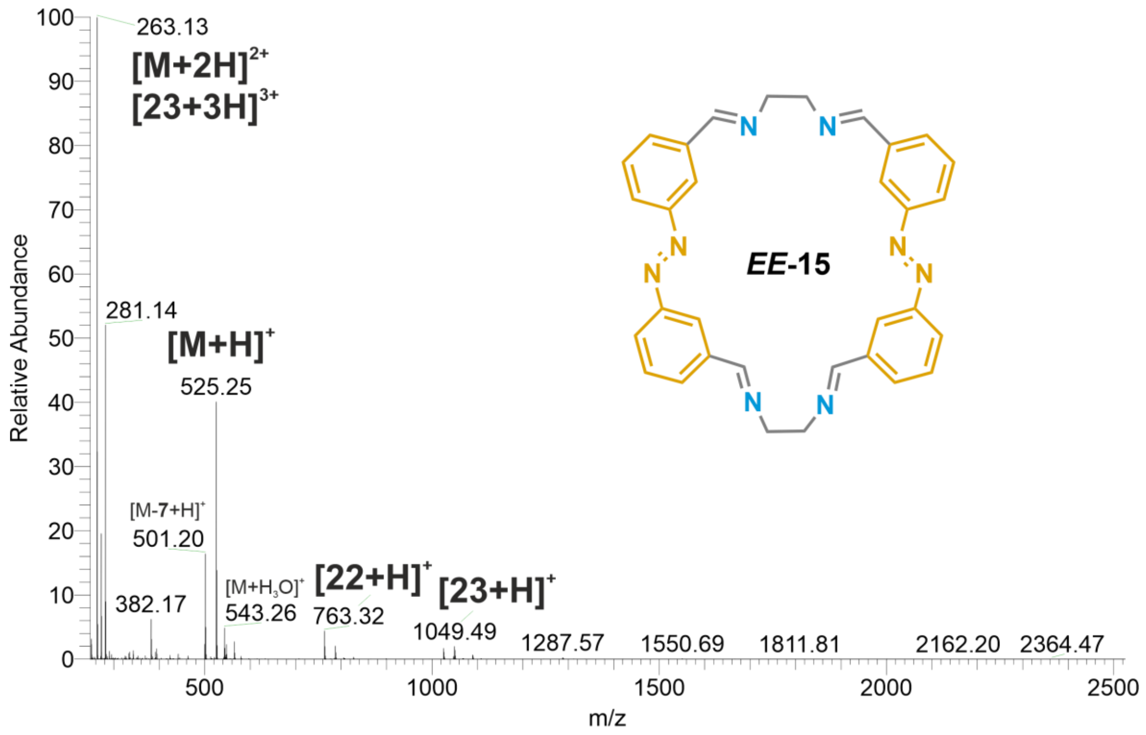


S87. HRMS-ESI spectra of self-assembled macrocycle **15** up to 2500 m/z. The signals corresponding to the singly ionized $[M+H]^+$ and doubly ionized $[M+2H]^{2+}$ are shown at 525.25 m/z and 263.13 m/z, respectively. Additionally, signals that correspond to the [3+3] and [4+4] macrocycles **22** and **23** are also shown.

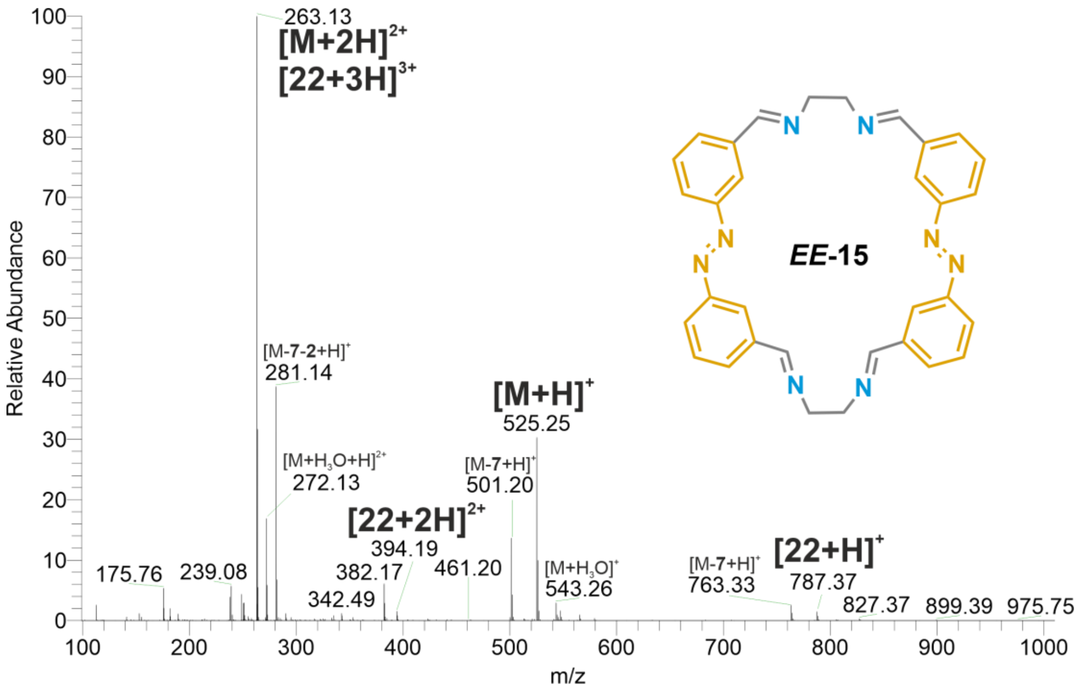


S88. HRMS-ESI spectra of self-assembled macrocycle **15** up to 1000 m/z. The signals corresponding to the singly ionized $[M+H]^+$ and doubly ionized $[M+2H]^{2+}$ are shown at 525.25 m/z and 263.13 m/z, respectively. Additionally, signals that correspond to the [3+3] macrocycle **22** are also shown.

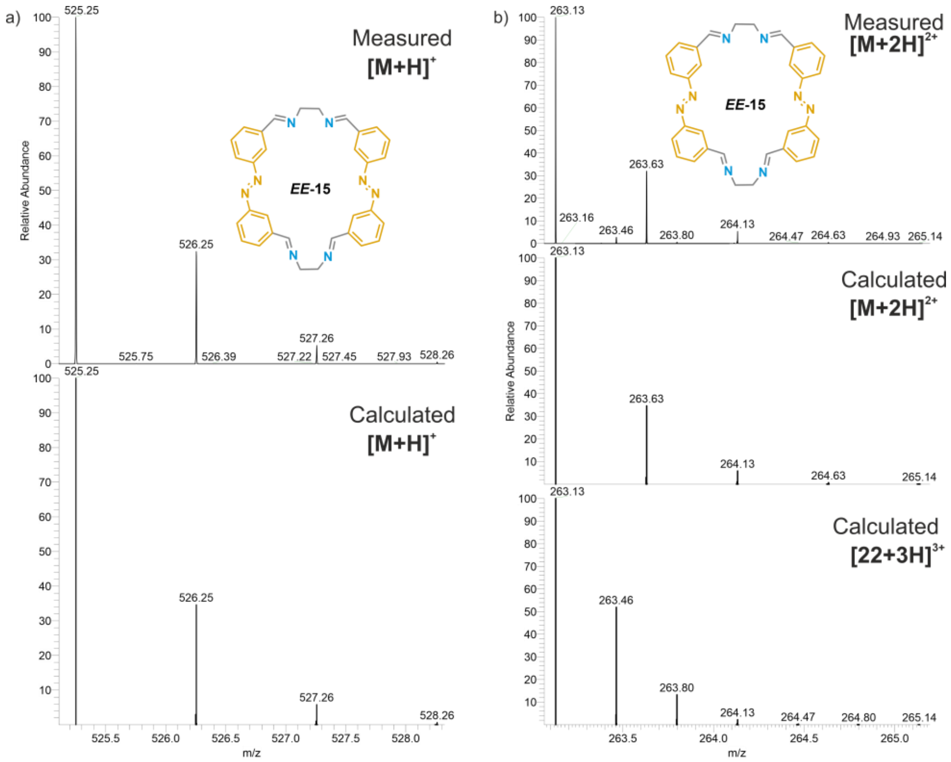


S89. Expansion of the signals for ESI-HRMS and comparation with the calculated spectra of **15**. **a**) singly charged [**M**+H]$^{+}$ and **b**) the isobaric species [**M**+H]$^{+}$ and [**22**+3H]$^{+3}$.

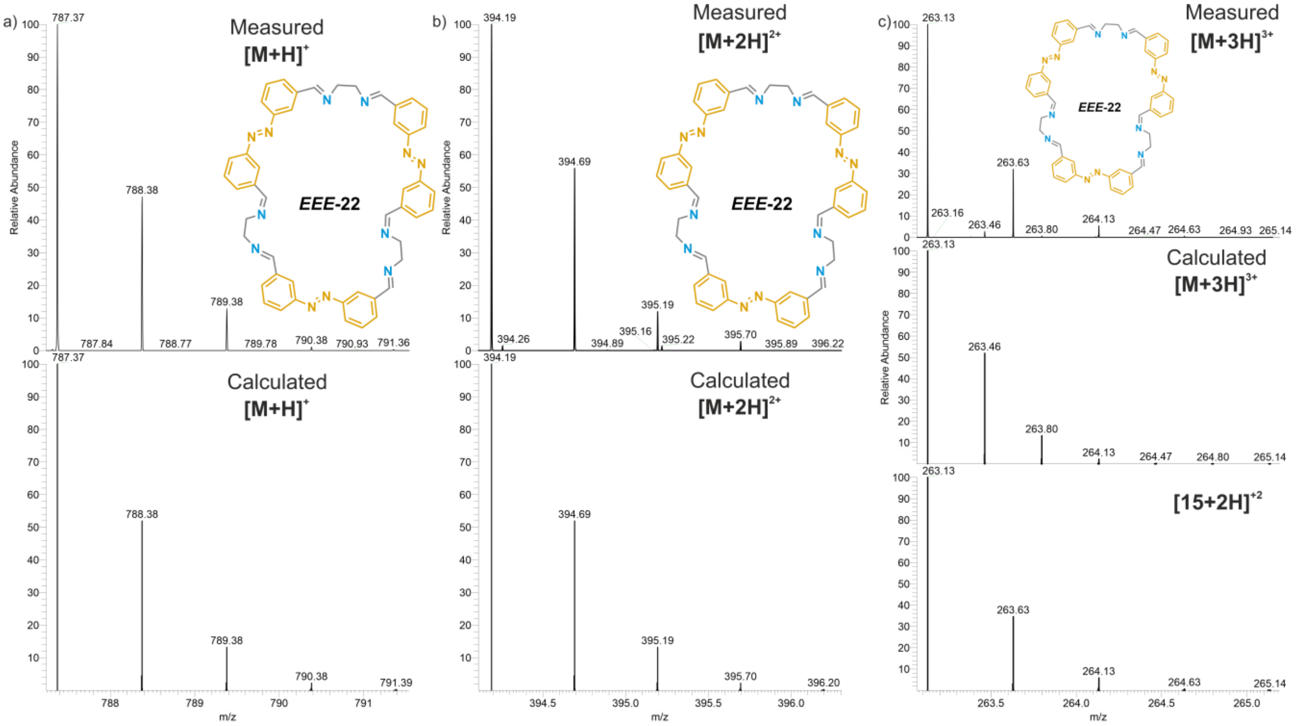


S90. Expansion of the signals for ESI-HRMS and comparation with the calculated spectra of **22**. **a**) singly charged [**M**+H]$^{+}$, **b**) doubly charged [**M**+2H]$^{2+}$, and **c)** the isobaric species [**M**+3H]$^{3+}$ and [**15**+2H]$^{+2}$.

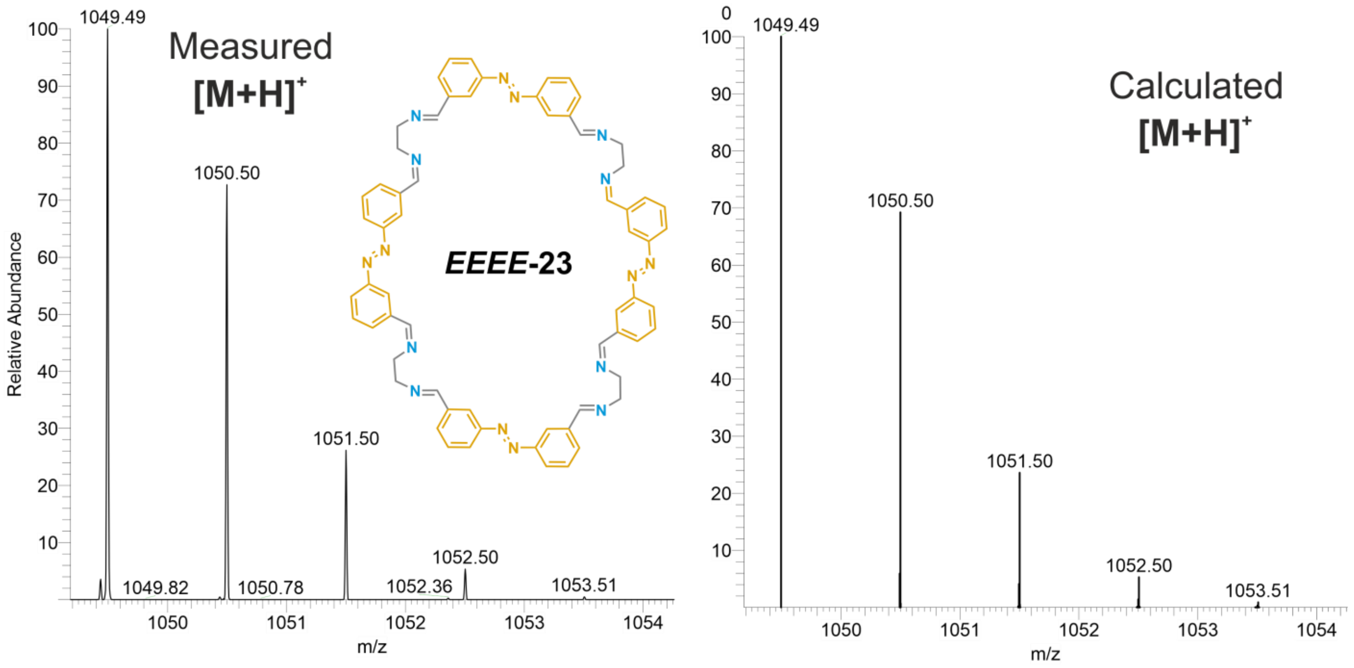


S91. Expansion of the signals for ESI-HRMS and comparation with the calculated spectra of the singly charged [**M**+H]$^{+}$ for the [4+4] macrocycle **23**.

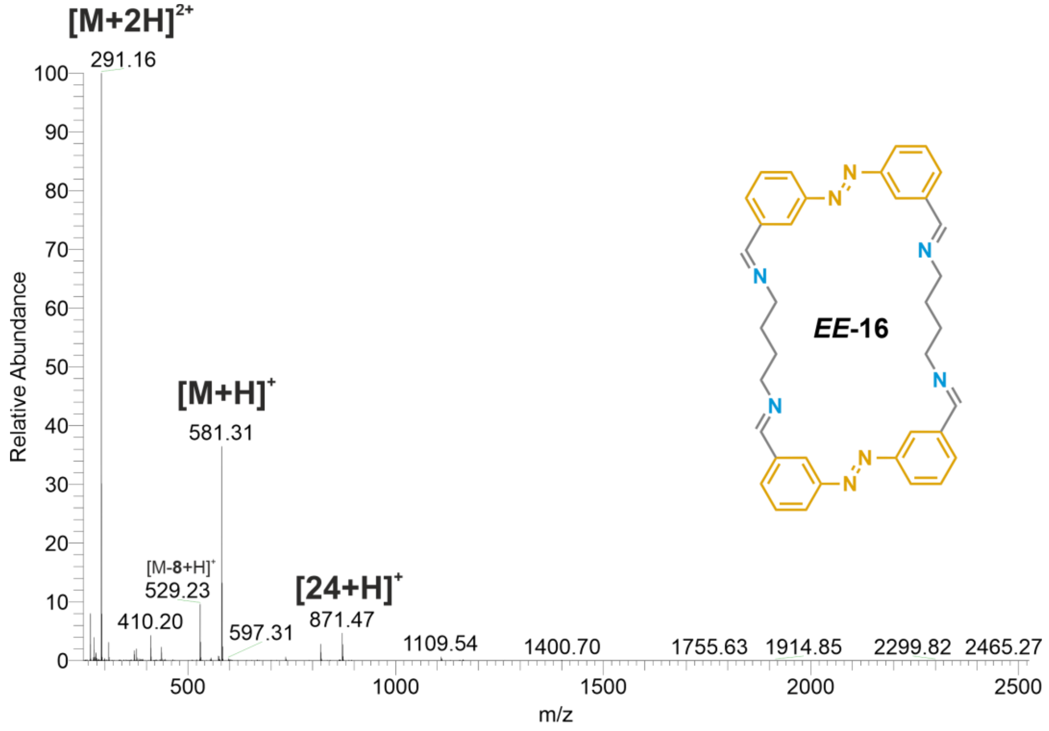


S92. HRMS-ESI spectra of self-assembled macrocycle **16** up to 2500 m/z. The signals corresponding to the singly ionized [M+H]$^{+}$ and doubly ionized [M+2H]$^{2+}$ are shown at 581.31 m/z and 291.16 m/z, respectively. Additionally, signals that correspond to the [3+3] macrocycles **24** are also shown.

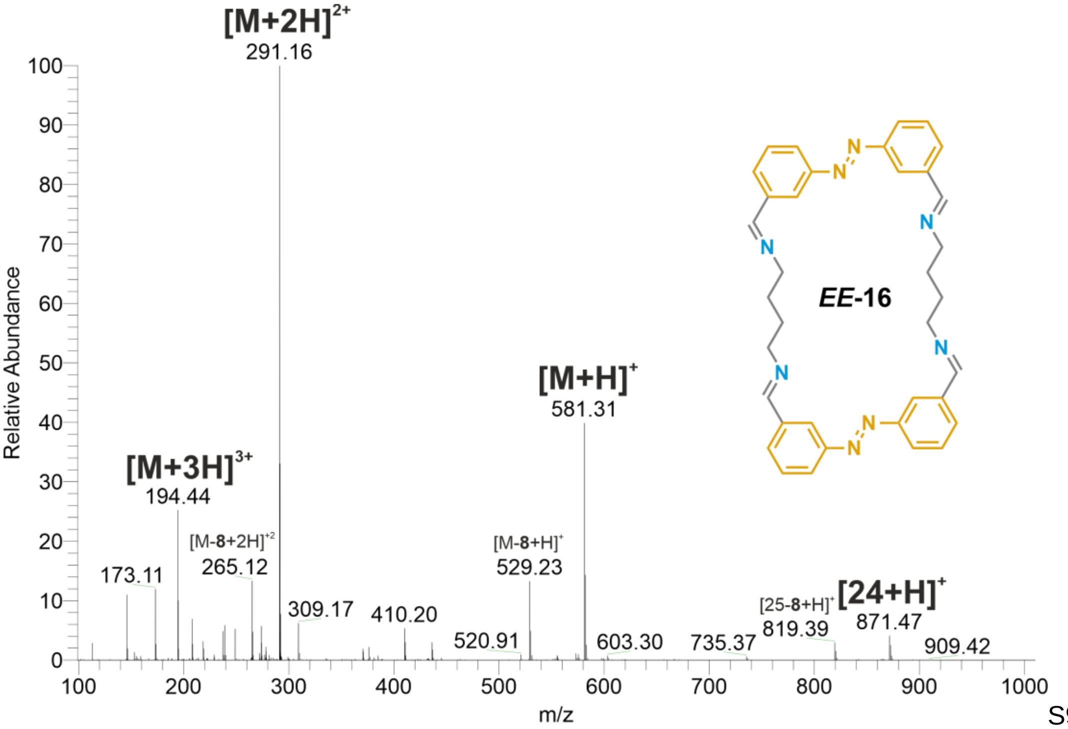


S93. HRMS-ESI spectra of self-assembled macrocycle **16** up to 1000 m/z. The signals corresponding to the singly ionized $[M+H]^+$ and doubly ionized $[M+2H]^{2+}$ are shown at 581.31 m/z and 291.16 m/z, respectively. Additionally, signals that correspond to the [3+3] macrocycles **24** are also shown.

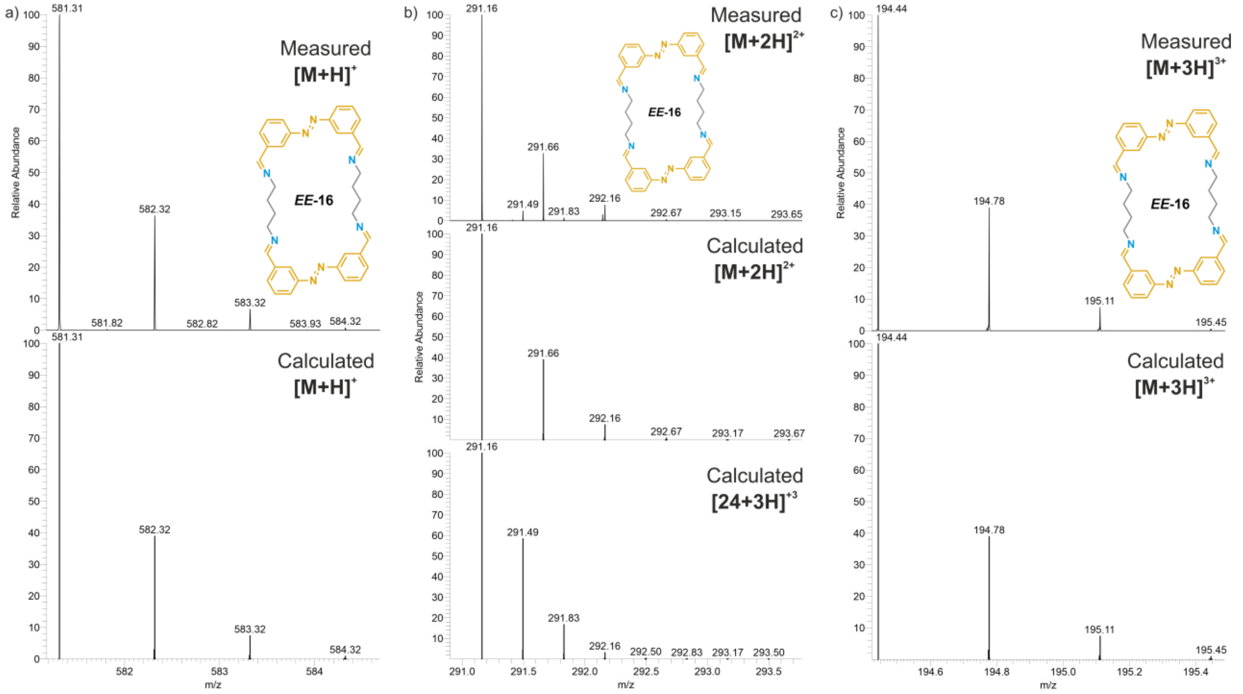


S94. Expansion of the signals for ESI-HRMS and comparation with the calculated spectra of **16**. **a**) singly charged [**M**+H]$^+$, **b**) the isobaric species [**M**+H]$^+$ and [**24**+2H]$^{+2}$ and **c**) triply charged [**M**+3H]$^{3+}$.

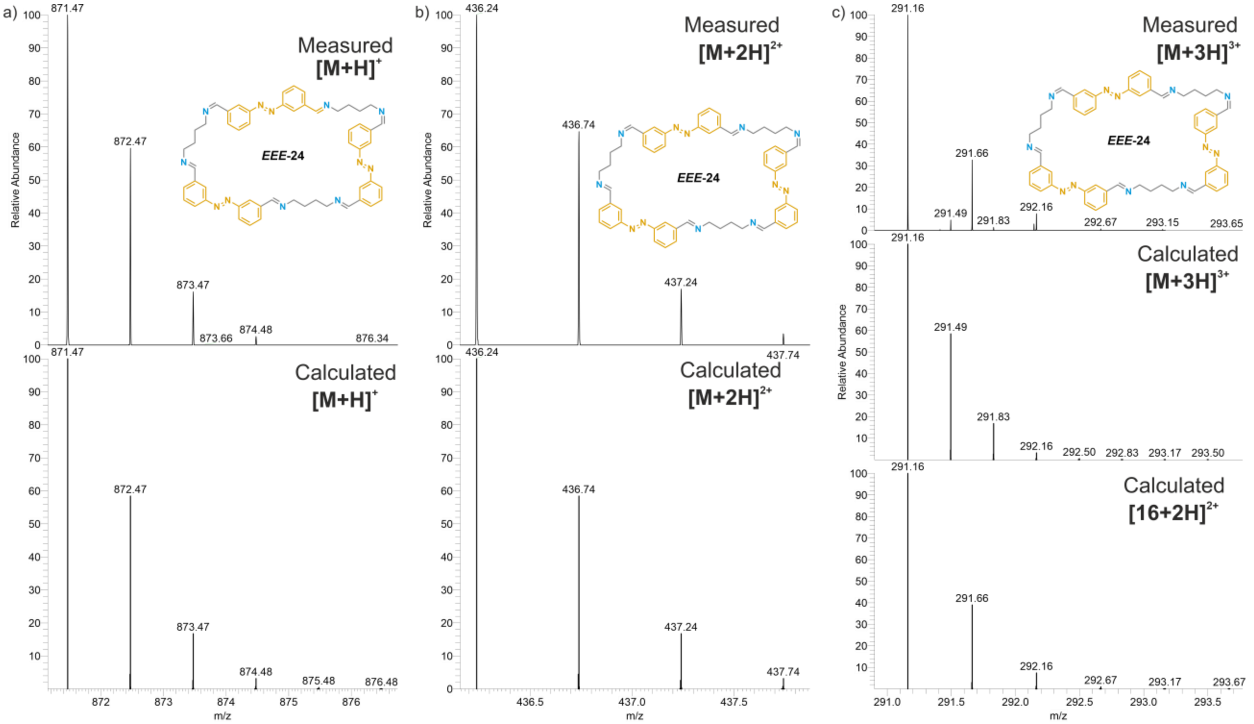

S95. Expansion of the signals for ESI-HRMS and comparation with the calculated spectra of **24**. **a**) singly charged [**M**+H]$^+$, **b**) doubly charged [**M**+H2]$^{+2}$ and **c**) the isobaric species [**M**+3H]$^{3+}$ and [16+2H]$^{+2}$.

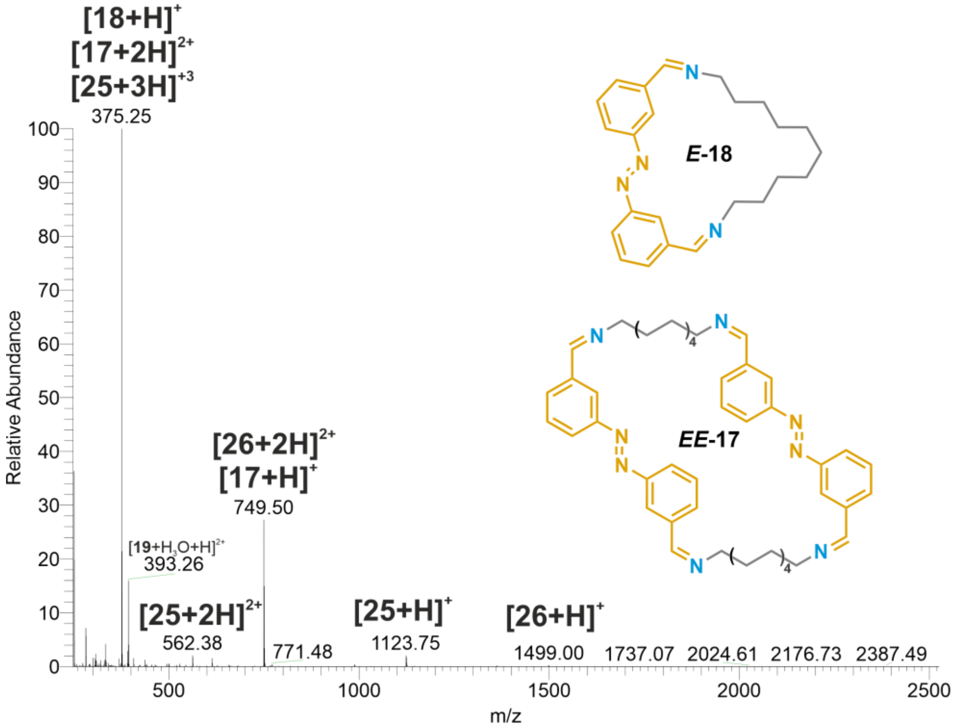

S96. HRMS-ESI spectra of self-assembled macrocycles **17** and **18** up to 2500 m/z. Additionally, signals that correspond to the [3+3] and [4+4] macrocycles **25** and **26** are also shown.

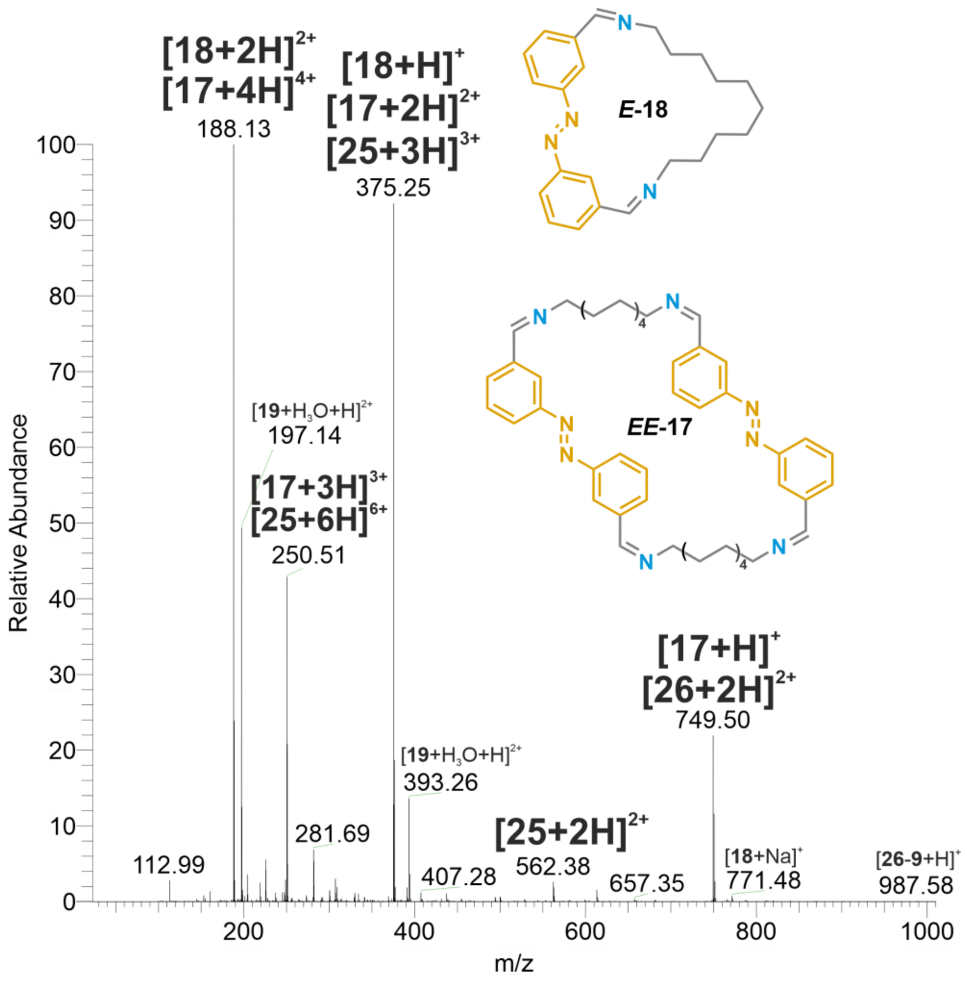


S97. HRMS-ESI spectra of self-assembled macrocycles **17** and **18** up to 1000 m/z. Additionally, signals that correspond to the [3+3] and [4+4] macrocycles **25** and **26** are also shown.

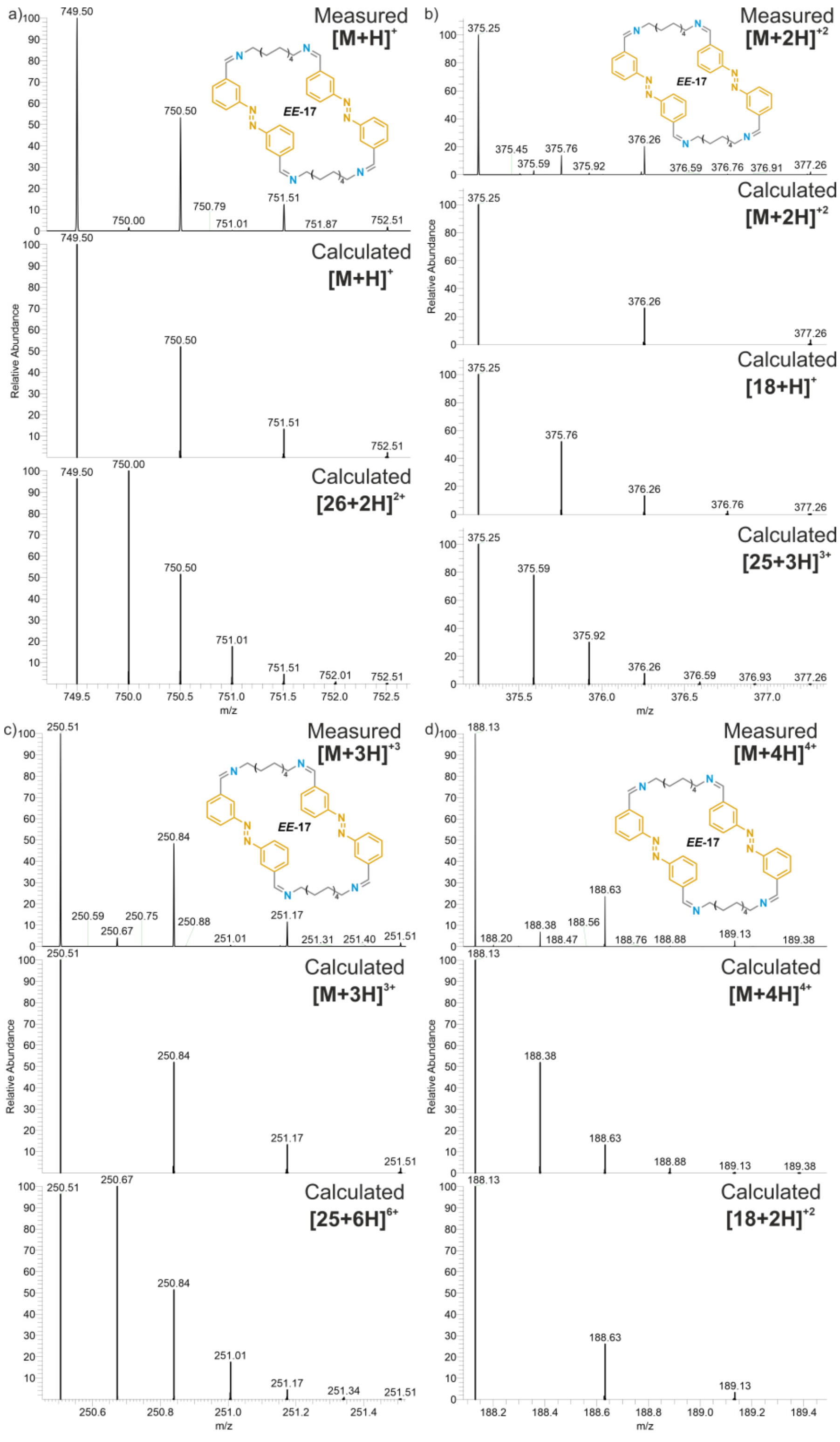


S98. Expansion of the signals for ESI-HRMS and comparation with the calculated spectra of **17**. **a**) isobaric species [**M**+H]$^{+}$ and [**26**+2H]$^{2+}$, **b**) isobaric species [**M**+2H]$^{2+}$, [**18**+H]$^{+}$ and [**25**+3H]$^{3+}$, **c**) isobaric species [**M**+3H]$^{3+}$ and [**25**+6H]$^{6+}$ and **d**) ) isobaric species [**M**+4H]$^{4+}$, and [**18**+2H]$^{2+}$.

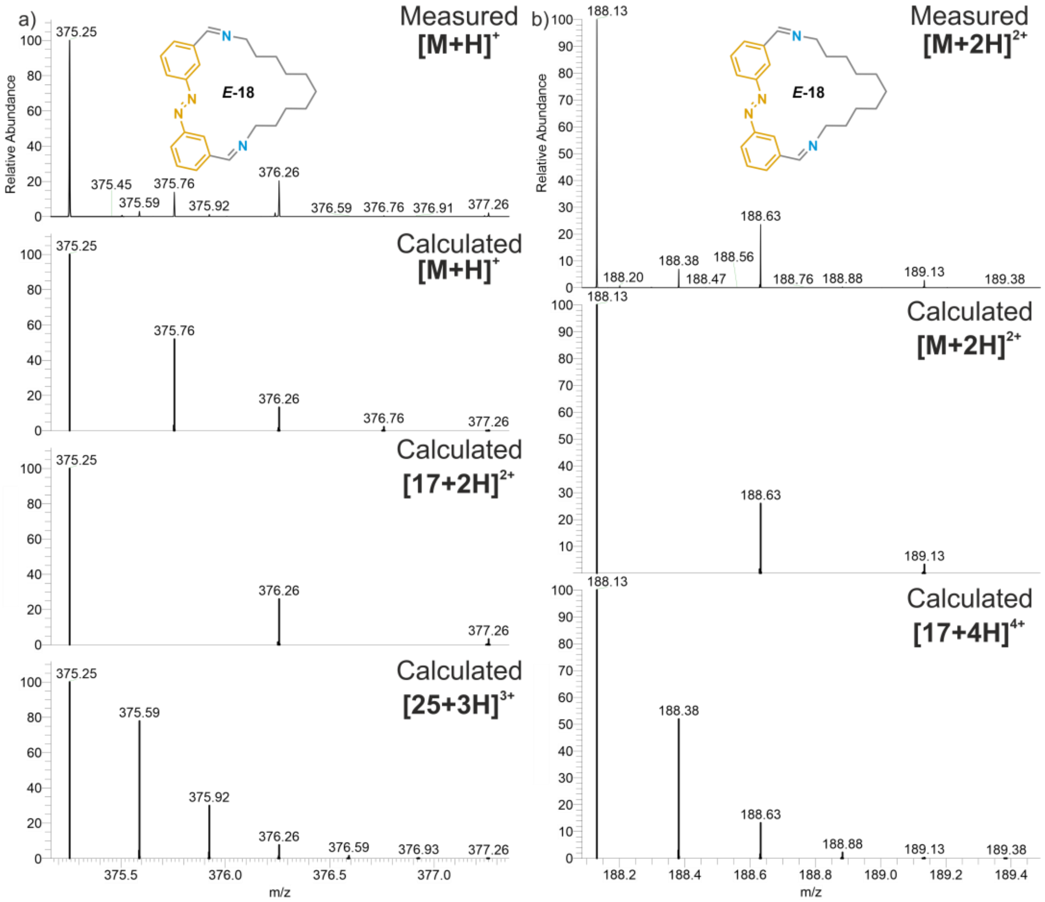


S99. Expansion of the signals for ESI-HRMS and comparation with the calculated spectra of **18**. **a**) isobaric species [**M**+H]$^{+}$, [**17**+2H]$^{2+}$ and [**25**+3H]$^{3+}$ and **b**) isobaric species [**M**+2H]$^{2+}$, [**17**+4H]$^{4+}$.

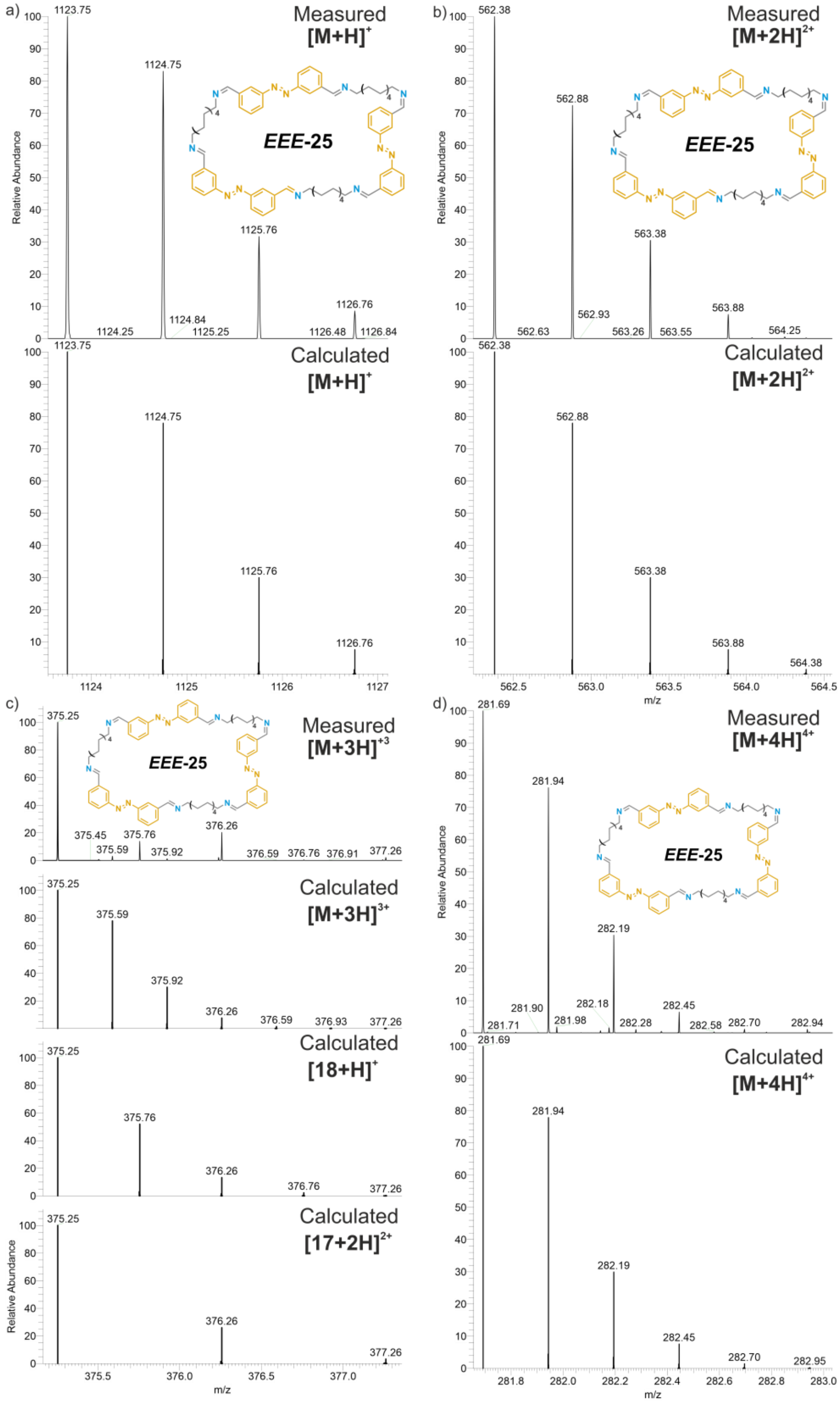


S100. Expansion of the signals for ESI-HRMS and comparation with the calculated spectra of **25**. **a**) singly charged [**M**+H]$^{+}$, **b**) doubly charged [**M**+2H]$^{2+}$ **c**) isobaric species [**M**+3H]$^{3+}$, [**18**+H]$^{+}$ and [**17**+2H]$^{2+}$ and **d**) quadruply charged [**M**+4H]$^{4+}$.

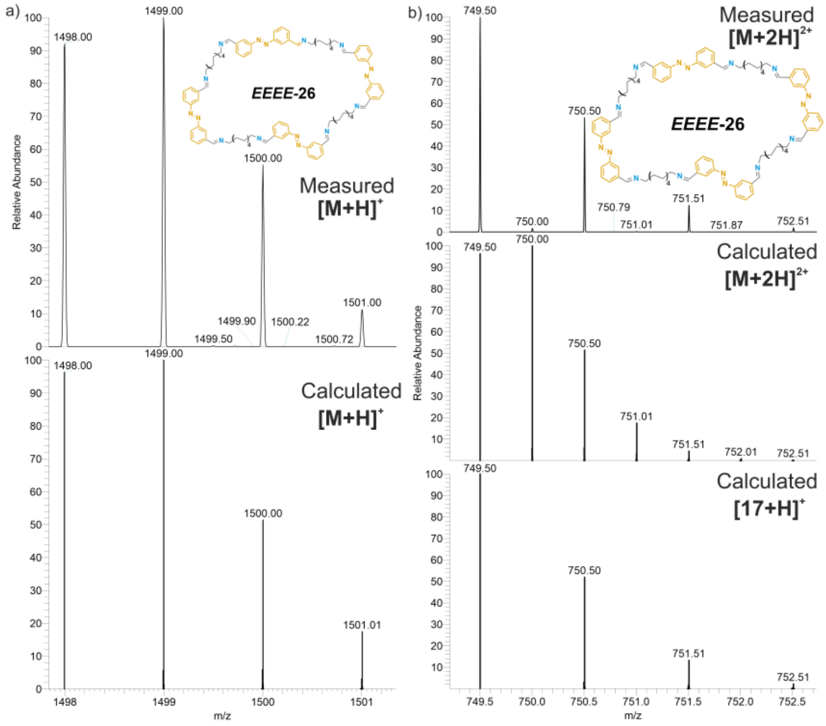


S101. Expansion of the signals for ESI-HRMS and comparation with the calculated spectra of **26**. **a**) singly charged [**M**+H]$^+$, **b**) isobaric species [**M**+2H]$_2$$^+$, and [**17**+H]$^+$

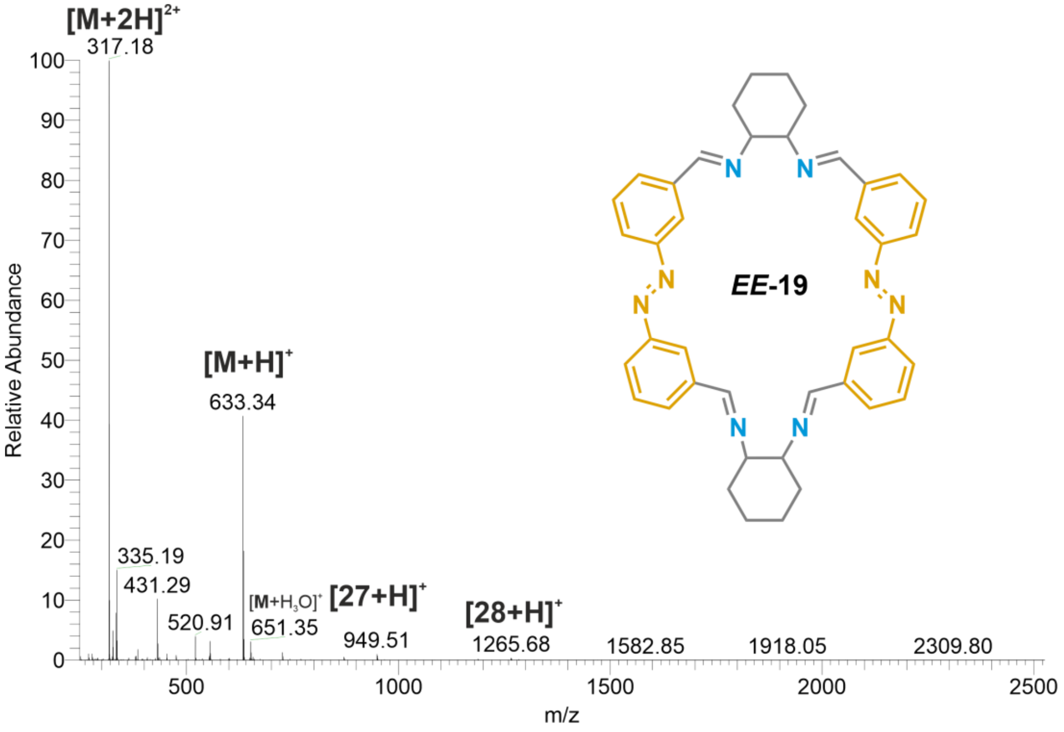


S102. HRMS-ESI spectra of self-assembled macrocycle **19** up to 2500 m/z. The signals corresponding to the singly ionized [M+H]$^+$ and doubly ionized [M+2H]$^{2+}$ are shown at 633.34 m/z and 317.18 m/z, respectively. Additionally, signals that correspond to the [3+3] and [4+4] macrocycles **27** and **28** are also shown.

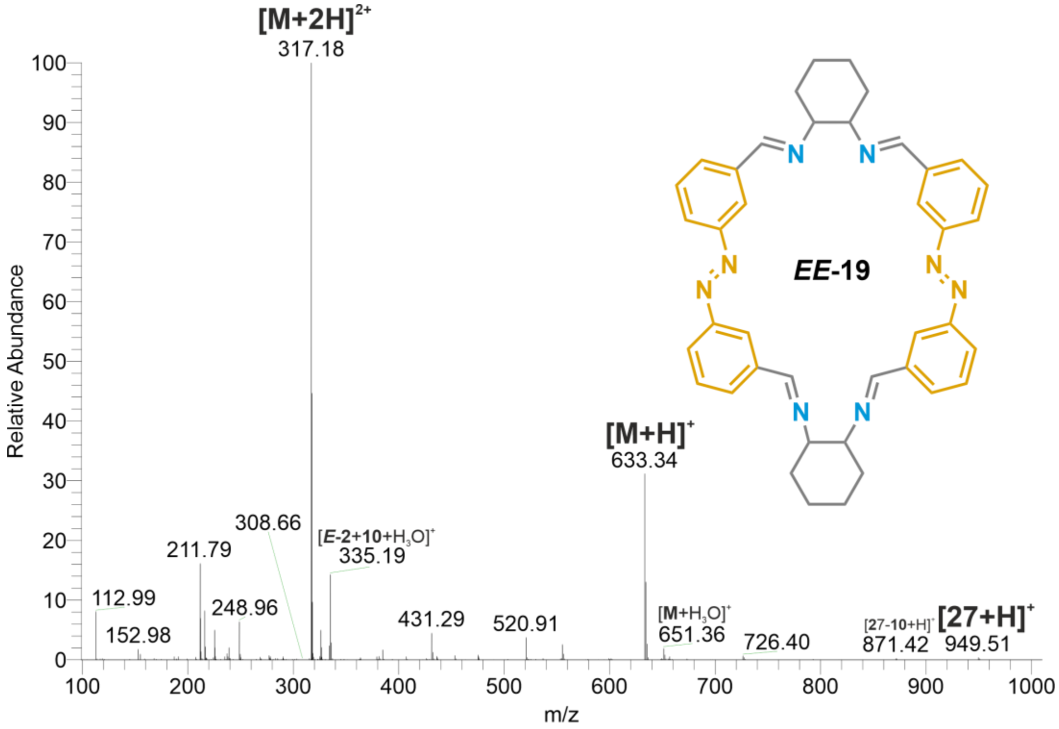


S103. HRMS-ESI spectra of self-assembled macrocycle **19** up to 1000 m/z. The signals corresponding to the singly ionized $[M+H]^{+}$ and doubly ionized $[M+2H]^{2+}$ are shown at 633.34 m/z and 317.18 m/z, respectively. Additionally, signals that correspond to the [3+3] macrocycles **27** are also shown.

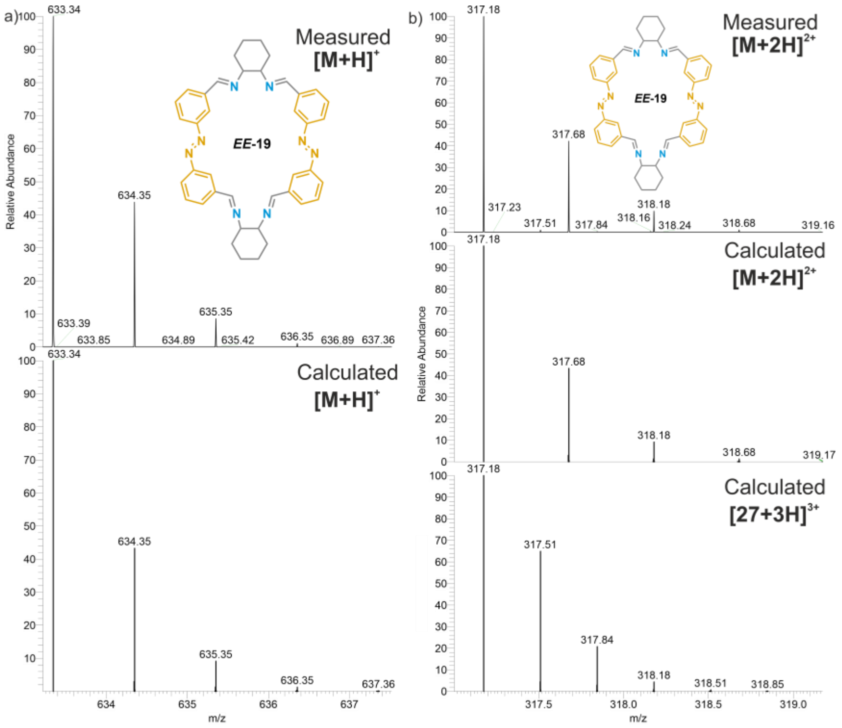


S104. Expansion of the signals for ESI-HRMS and comparation with the calculated spectra of **19**. **a**) singly charged [**M**+H]$^{+}$ and **b**) the isobaric species [**M**+2H]$^{2+}$ and [**27**+3H]$^{+3}$.

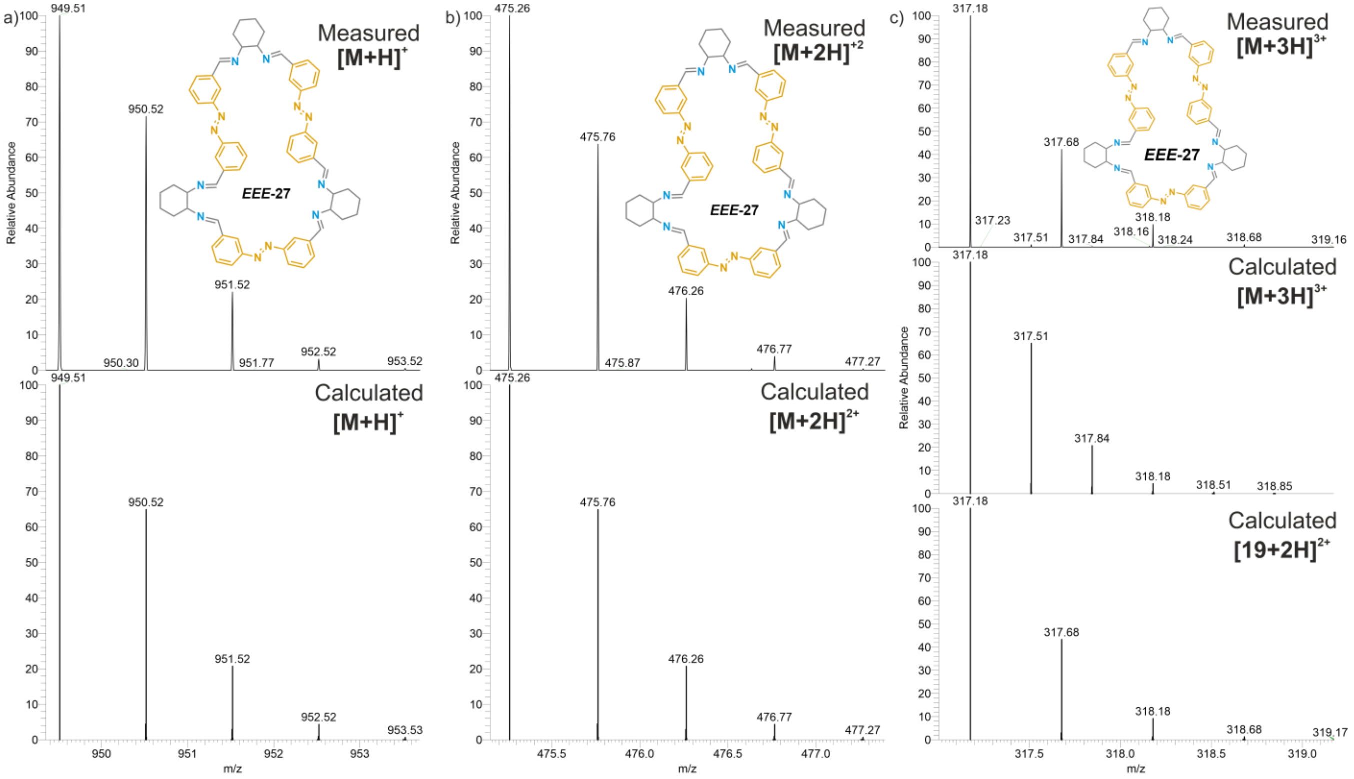


S105. Expansion of the signals for ESI-HRMS and comparation with the calculated spectra of **27**. **a** singly charged [**M**+H]$^+$, **b**) doubly charged [**M**+2H]$^{2+}$ and **c**) isobaric species [**M**+3H]$^{3+}$ and [**19**+2H]$^{2+}$

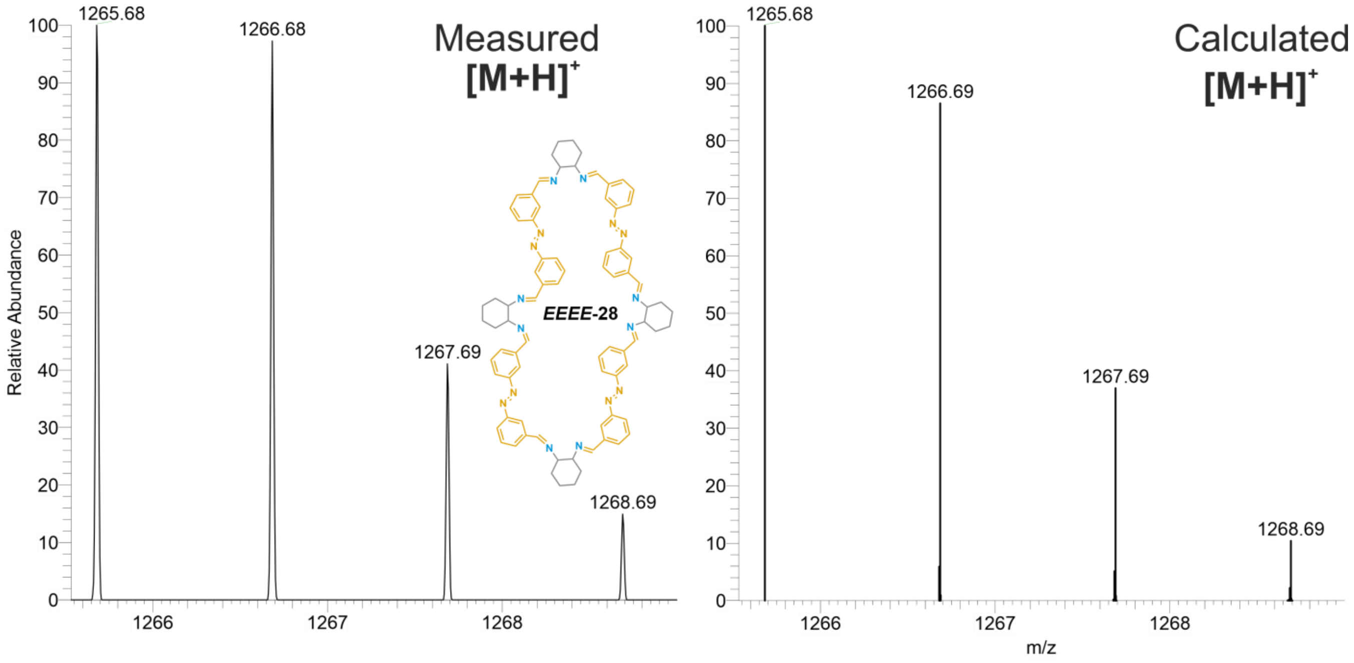


S106. Expansion of the signals for ESI-HRMS and comparation with the calculated spectra of the singly charged [**M**+H]$^+$ for the [4+4] macrocycle **28**.

## Single-Crystal X-Ray Analysis

Crystals of ***EE*-3** were obtained by vapor diffusion of pentane into a solution of ***EE*-3** in chloroform. A single crystal was mounted on a cryoloop and analyzed on a Bruker-AXS D8 Venture diffractometer, using MoKα radiation (λ = 0.71073 Å). The data collection was done at room temperature under ambient conditions. The Bruker APEX4 software suite was used for data collection and processing, and a multi-scan absorption correction was applied using SADABS (SADABS-2016/2).[4] The structure was solved using SHELXT.[5] Subsequent refinement was done using SHELXL[6] in the OLEX2 software package.[7] Hydrogen atoms were generated by geometrical considerations and refined using a riding model.

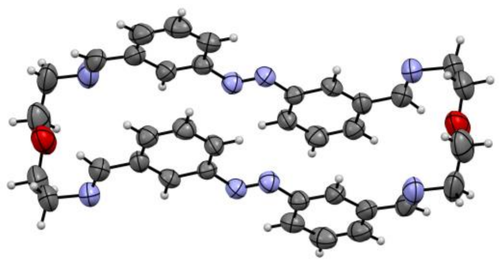

**Table S1. Crystallographic data for *EE*-3.**

| | |
|---|---|
| Empirical formula | $C_{36}H_{36}N_8O_2$ |
| Formula weight | 612.73 |
| Temperature/K | 293 |
| Crystal system | monoclinic |
| Space group | $P2_1/n$ |
| a/Å | 6.0442(16) |
| b/Å | 12.436(3) |
| c/Å | 22.127(5) |
| α/° | 90 |
| β/° | 97.737(13) |
| γ/° | 90 |
| Volume/Å$^3$ | 1648.1(7) |
| Z | 2 |
| $\rho_{calc}$g/cm$^3$ | 1.235 |
| μ/mm$^{-1}$ | 0.080 |
| F(000) | 648.0 |
| Crystal size/mm$^3$ | 0.241 × 0.069 × 0.035 |
| Radiation | MoKα (λ = 0.71073) |
| 2Θ range for data collection/° | 6.466 to 49.616 |
| Index ranges | -7 ≤ h ≤ 7, -14 ≤ k ≤ 14, -26 ≤ l ≤ 26 |
| Reflections collected | 30437 |
| Independent reflections | 2818 [$R_{int}$ = 0.1467, $R_{sigma}$ = 0.0452] |
| Data/restraints/parameters | 2818/0/208 |
| Goodness-of-fit on F$^2$ | 1.168 |
| Final R indexes [I>=2σ (I)] | $R_1$ = 0.1310, w$R_2$ = 0.1983 |
| Final R indexes [all data] | $R_1$ = 0.2143, w$R_2$ = 0.2383 |
| Largest diff. peak/hole / e Å$^{-3}$ | 0.51/-0.23 |

# Apendix

NMR spectra

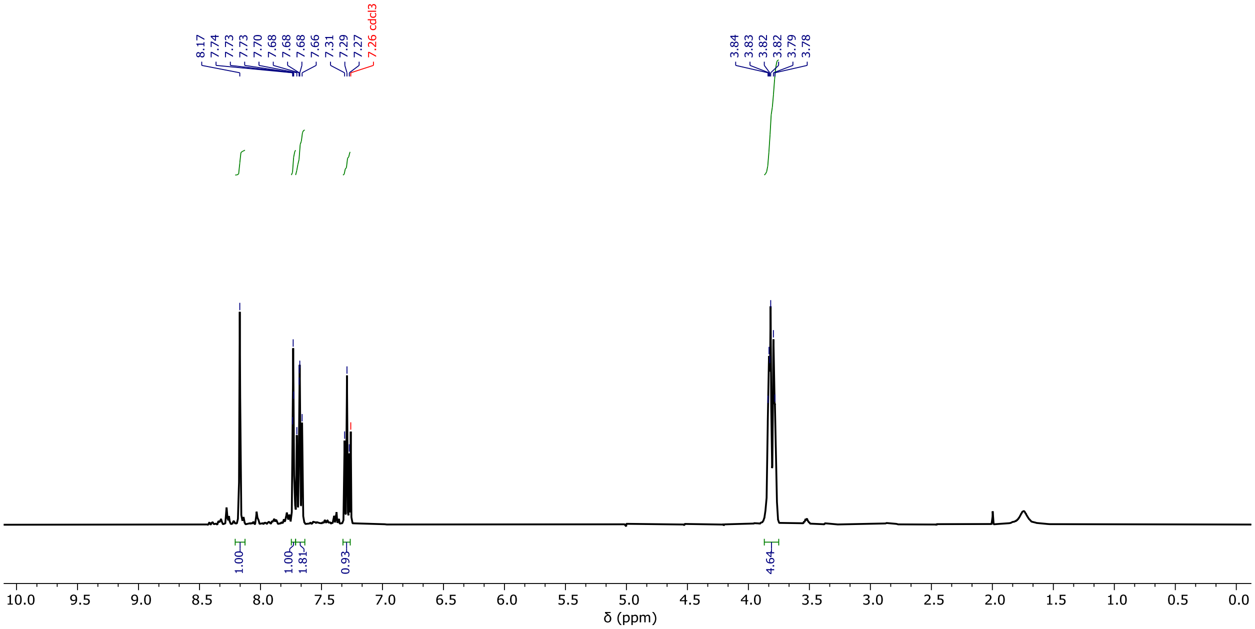


$^{1}H$ NMR (500 MHz, $CDCl_3$) spectra of macrocycle ***EE*-3**.

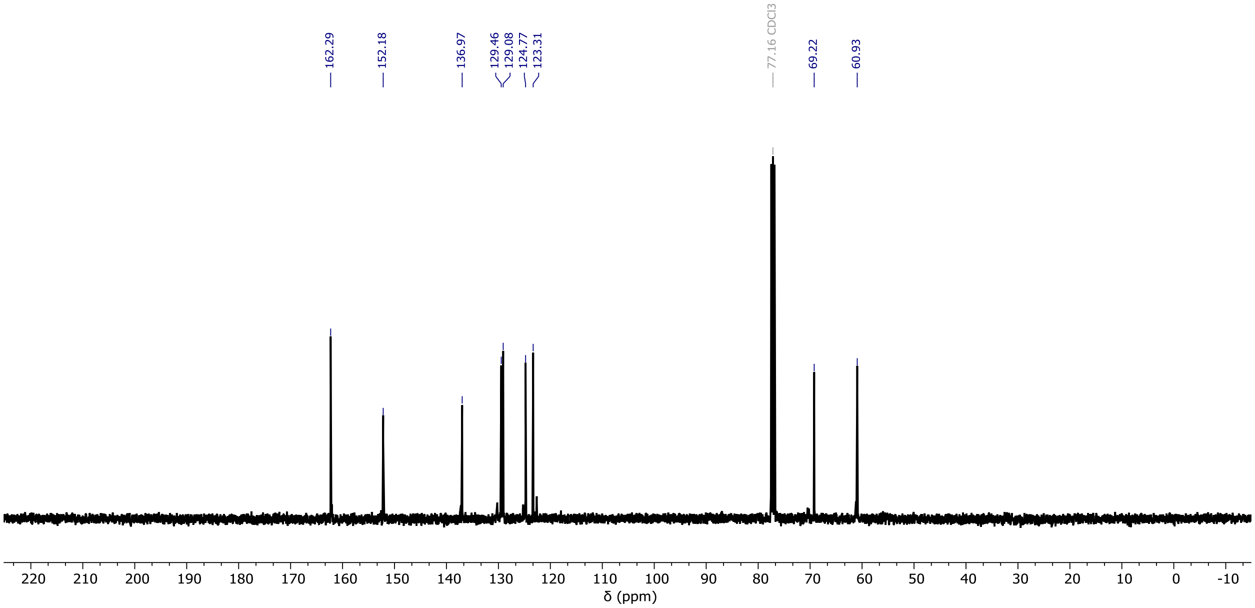


$^{13}C$ NMR (101 MHz, $CDCl_3$) spectra of macrocycle ***EE*-3**.

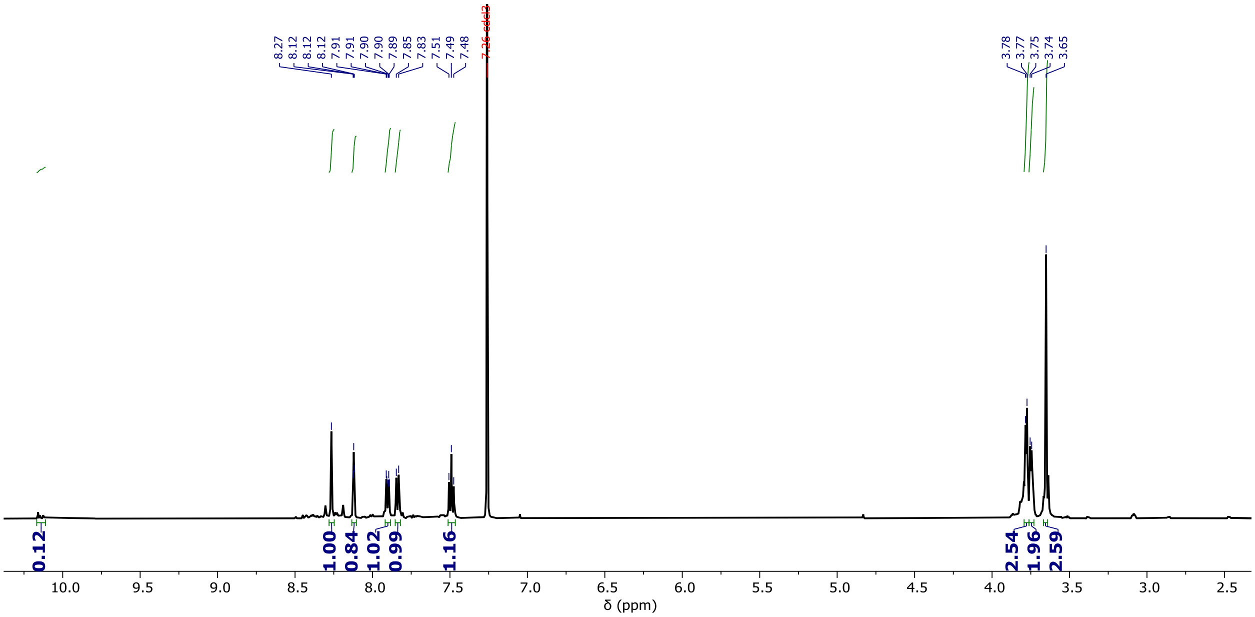

$^1H$ NMR (500 MHz, $CDCl_3$, TFA 5 µM) spectra of self-assembled macrocycle ***EE*-11**.

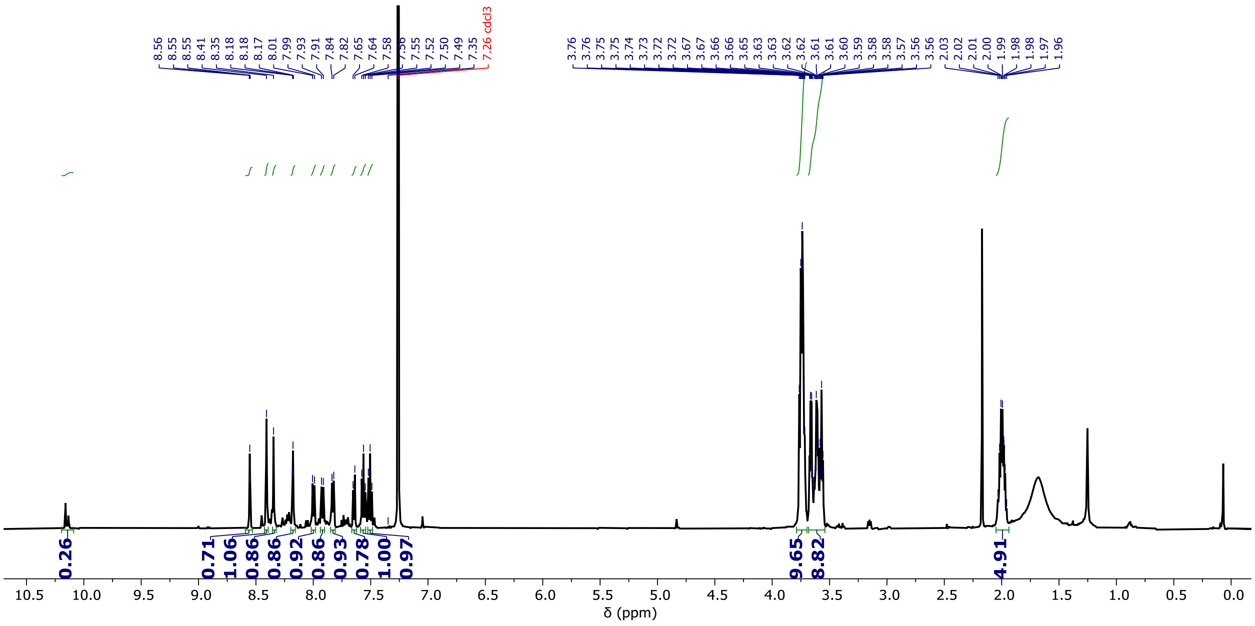

$^1H$ NMR (500 MHz, $CDCl_3$, TFA 5 µM) spectra of self-assembled macrocycles ***EE*-13** and ***E*-14**.

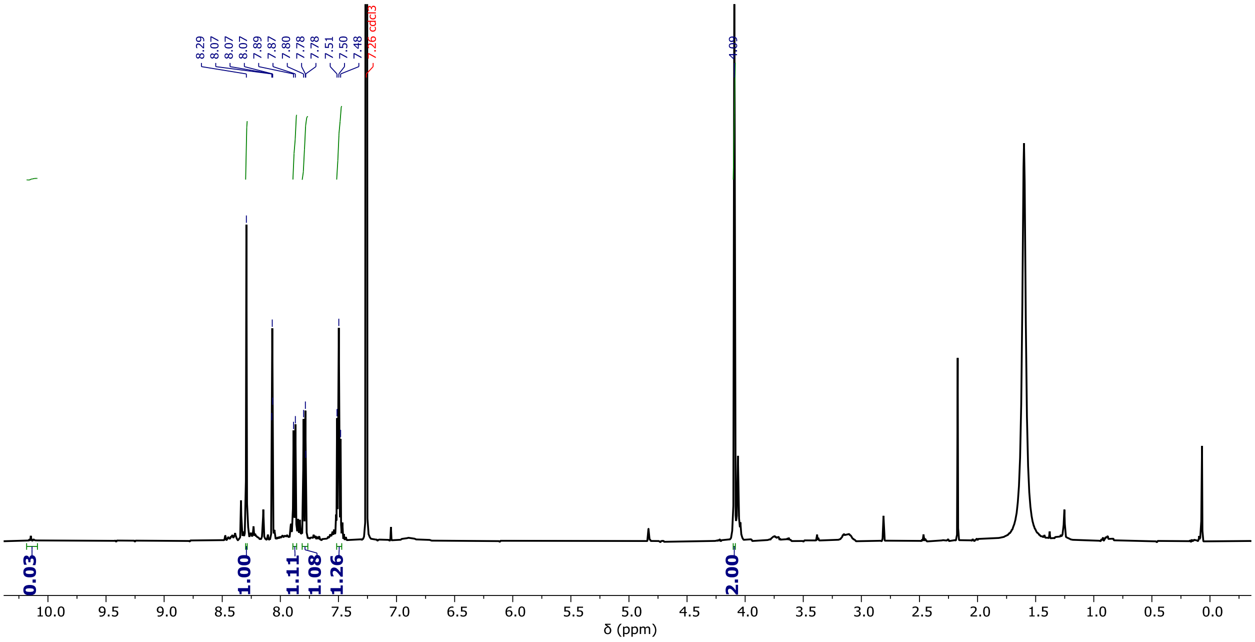

$^{1}H$ NMR (500 MHz, $CDCl_3$, TFA 5 μM) spectra of self-assembled macrocycle ***EE*-15**.

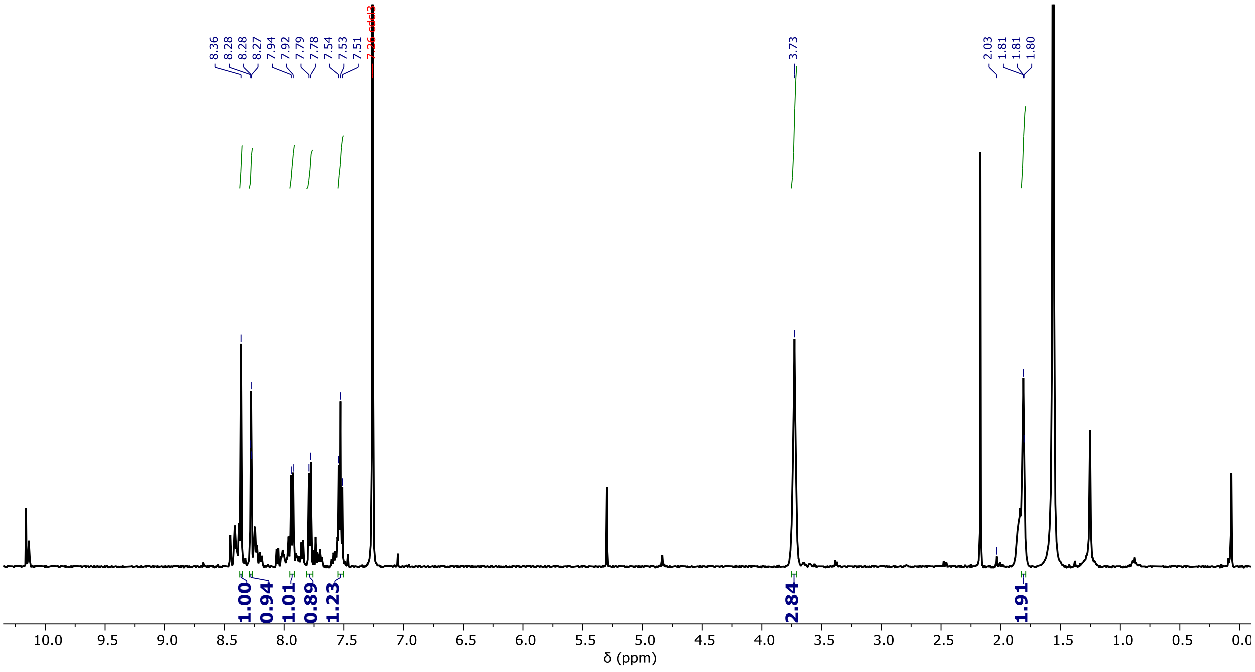

$^{1}H$ NMR (500 MHz, $CDCl_3$, TFA 5 μM) spectra of self-assembled macrocycle ***EE*-16**.

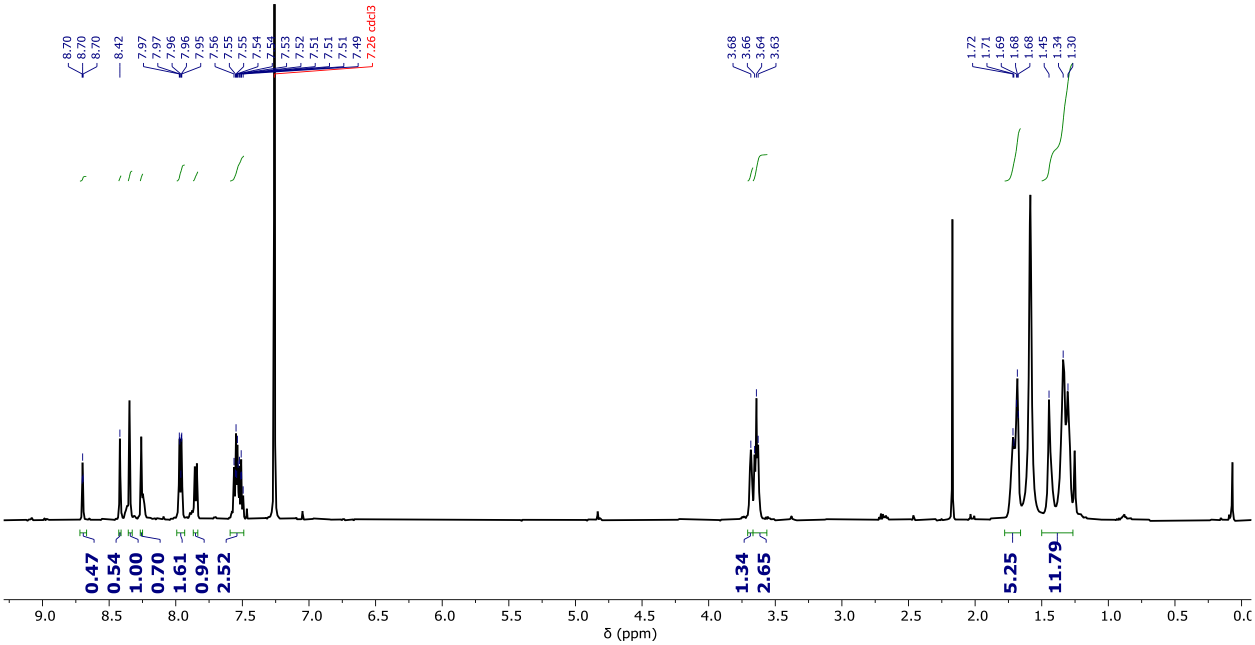

$^{1}$H NMR (500 MHz, $CDCl_3$, TFA 5 μM) spectra of self-assembled macrocycles ***EE*-17** and ***E*-18**.

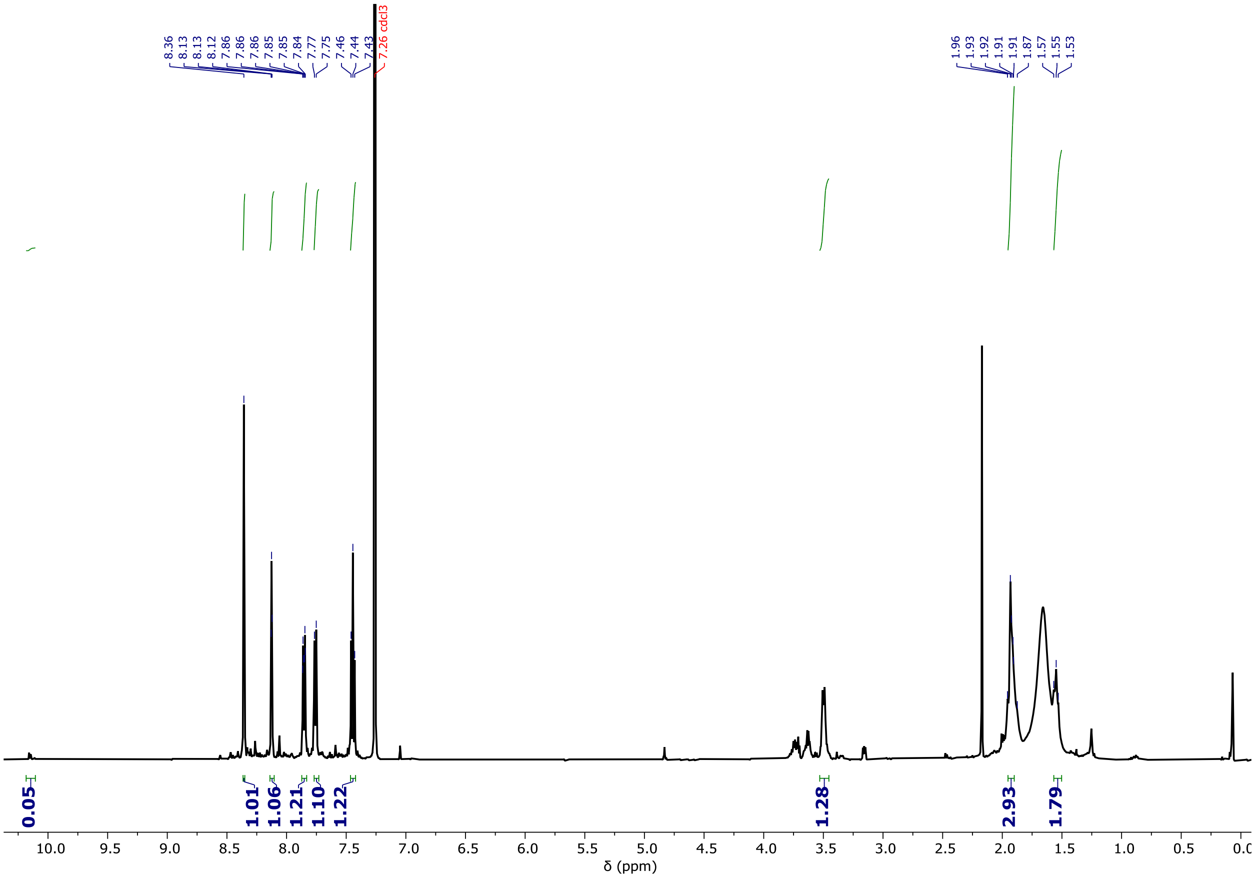

$^{1}$H NMR (500 MHz, $CDCl_3$, TFA 5 μM) spectra of self-assembled macrocycle ***EE*-19**.

## References


(1) Feldmeier, C.; Bartling, H.; Riedle, E.; Gschwind, R. M. LED Based NMR Illumination Device for Mechanistic Studies on Photochemical Reactions – Versatile and Simple, yet Surprisingly Powerful. *J. Magn. Reson.* **2013**, *232*, 39–44.

(2) Su, R.; Lü, L.; Zheng, S.; Jin, Y.; An, S. Synthesis and Characterization of Novel Azo-Containing or Azoxy-Containing Schiff Bases and Their Antiproliferative and Cytotoxic Activities. *Chem. Res. Chin. Univ.* **2015**, *31* (1), 60–64.

(3) Ovalle, M.; Kathan, M.; Toyoda, R.; Stindt, C. N.; Crespi, S.; Feringa, B. L. Light-Fueled Transformations of a Dynamic Cage-Based Molecular System. *Angew. Chem. Int. Ed.* **2023**, *62*, e202214495.

(4) Krause, L.; Herbst-Irmer, R.; Sheldrick, G. M.; Stalke, D. Comparison of Silver and Molybdenum Microfocus X-Ray Sources for Single-Crystal Structure Determination. *J. Appl. Cryst.* **2015**, *48* (1), 3–10.

(5) Sheldrick, G. M. SHELXT – Integrated Space-Group and Crystal-Structure Determination. *Acta Ctryst. A.* **2015**, *71* (1), 3–8.

(6) Sheldrick, G. M. A Short History of SHELX. *Acta Cryst. A* **2008**, *64* (1), 112–122.

(7) Dolomanov, O. V.; Bourhis, L. J.; Gildea, R. J.; Howard, J. a. K.; Puschmann, H. OLEX2: A Complete Structure Solution, Refinement and Analysis Program. *J. Appl. Cryst.* **2009**, *42* (2), 339–341.
